# Attaching Metal-Containing Moieties to β-Lactam Antibiotics: The Case of Penicillin and Cephalosporin

**DOI:** 10.1021/acs.inorgchem.4c01548

**Published:** 2024-06-26

**Authors:** María Moreno-Latorre, María C. de la Torre, Javier A. Cabeza, Pablo García-Álvarez, Miguel A. Sierra

**Affiliations:** † Instituto de Química Orgánica General, Consejo Superior de Investigaciones Científicas (IQOG-CSIC), Juan de la Cierva 3, 28006 Madrid, Spain; ‡ Departamento de Química Orgánica e Inorgánica, Facultad de Química, Universidad de Oviedo, 33071 Oviedo, Spain; § Departamento de Química Orgánica I, Facultad de Química, Universidad Complutense, 28040 Madrid, Spain; ∥ Centro de Innovación en Química Avanzada ORFEO-CINQA, https://orfeocinqa.es/

## Abstract

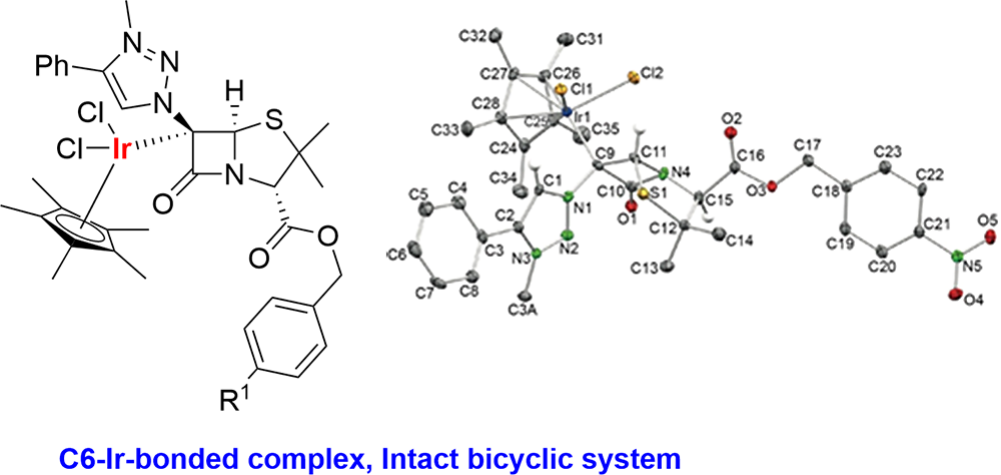

Procedures for the preparation of transition metal complexes having intact bicyclic cepham or penam systems as ligands have been developed. Starting from readily available 4-azido-2-azetidinones, a synthetic approach has been tuned using a copper-catalyzed azide–alkyne cycloaddition between 3-azido-2-azetinones and alkynes, followed by methylation and transmetalation to Au(I) and Ir(III) complexes from the mesoionic carbene Ag(I) complexes. This methodology was applied to 6-azido penam and 7-azido cepham derivatives to build 6-(1,2,3-triazolyl)penam and 7-(1,2,3-triazolyl)cepham proligands, which upon methylation and metalation with Au(I) and Ir(III) complexes yielded products derived from the coordination of the metal to the penam C^6^ and cepham C^7^ positions, preserving intact the bicyclic structure of the penicillin and cephalosporin scaffolds. The crystal structure of complex **28b**, which has an Ir atom directly bonded to the intact penicillin bicycle, was determined by X-ray diffraction. This is the first structural report of a penicillin-transition-metal complex having the bicyclic system of these antibiotics intact. The selectivity of the coordination processes was interpreted using DFT calculations.

## Abstract 2

Penicillin and cephalosporin derivatives containing C6–M and C7–M (M = Ir, Au) bonds have been synthesized, and the X-ray structure of the C6–Ir penam complex has been resolved. This research may be significant for the development of new active compounds targeting β-lactamase-producing bacteria.

## Introduction

The resistance of bacteria to current clinically used antibiotics is a worldwide sanitary emergency of the first magnitude. In 2019, the most optimistic estimation was a 4.95 million death toll caused by bacterial infections, with 1.3 million directly attributable to bacteria resistant to the currently used antibiotics.^1,2^ By 2050, it is foreseeable that the number of worldwide deaths caused by bacteria will be around 10 million, a figure considered conservative nowadays. Among other factors, the massive use of antibacterial drugs prescribed during the COVID-19 pandemic may strongly contribute to an increase in bacterial resistance.^3,4^

Bacterial resistance is the main cause of this explosive outgrowth of life-threatening bacterial infections. The appearance of bacteria resistant to antibiotics is simultaneous with the beginning of their clinical use.^5^ New strains of microorganisms producing β-lactamases (mβls) have evolved during the last 20 years, and today, those able to produce New Delhi metallo-β-lactamases (NDM-1) are, in some cases, resistant to all of the clinically used β-lactam antibiotics.^6^ So far, more than 500 different metallo-β-lactamases have been described.

**Scheme 1:**
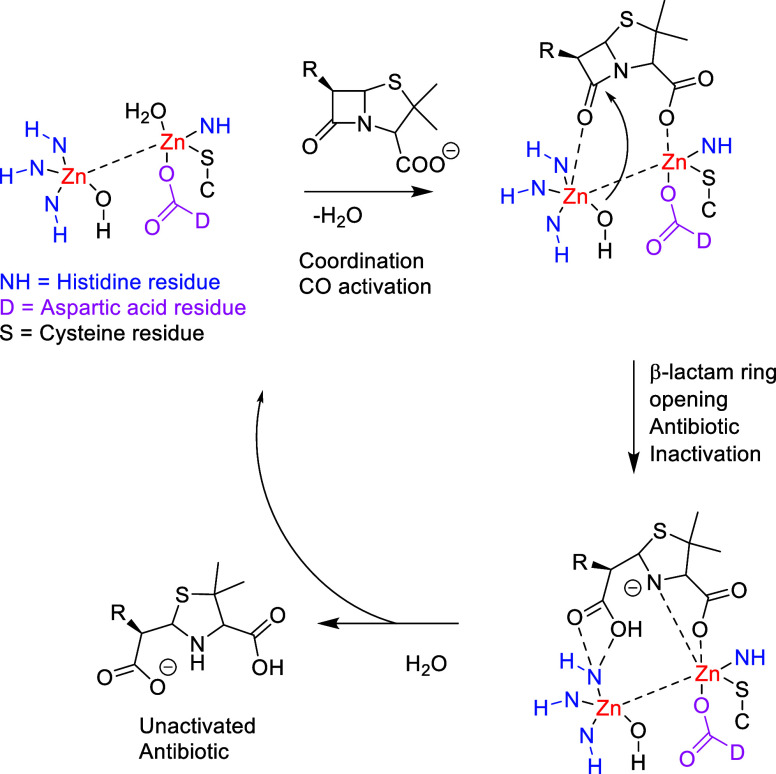
Schematic Mode of Action of β-Lactam Cleavage by a Dizinc mβl

β-Lactamases hydrolyze the four-membered ring common to all β-lactam antibiotics,^7^ therefore destroying the ability of these compounds to acylate the active serine sites of the penicillin-binding protein and hence inhibiting the building of the bacteria cell wall. mβls contain one or two Zn(II) ions that have a key role in substrate binding.^8^ It is currently assumed that the amino acid residues and the flexible loops play only minor roles in stabilizing the enzyme–substrate complex. The lack of crystal structures of a mβl and an unhydrolyzed substrate keeps this assumption at the hypothesis level (Scheme 1).^9^

Analogously, the coordination of clinically used β-lactams to several transition metals has been repeatedly studied. However, to date, no crystal diffraction data of a metal complex of 2-azetidinone have been reported. Finally, several mβls are able to exchange their Zn(II) with different metals while maintaining their mβl activity.^10^ The situation is complicated because cephalosporin, penicillin, and different clinically used antibiotics are not only multidentate ligands but also amphoteric products, with coordination points strongly dependent on the pH.^11^

Metallo-derivatives of clinically used antibacterial agents have been previously prepared, and their antibacterial activity was determined. For example, coordination complexes of quinolone antibiotics have been deeply studied,^12^ as well as silver complexes that were, arguably, the first antibacterial agents used by humanity.^13^ The importance of silver-based new materials and coatings, as well as the use of silver nanoparticles as antibacterial agents, is exponentially increasing, and these topics have been thoroughly reviewed.^14^ Using a different approach, siderophores were used as “Trojan Horses” to introduce an antibacterial agent inside the bacteria, fooling the cell membrane barrier by attaching the antibiotic to the Fe-siderophore.^15^ Metallo-peptides have also been tested as antibacterial agents.^16^ Finally, the number of reports regarding the antibiotic activity of different classes of metal complexes is exponentially growing.^17^ These complexes lack fragments related to clinically used drugs.

**Figure 1:**
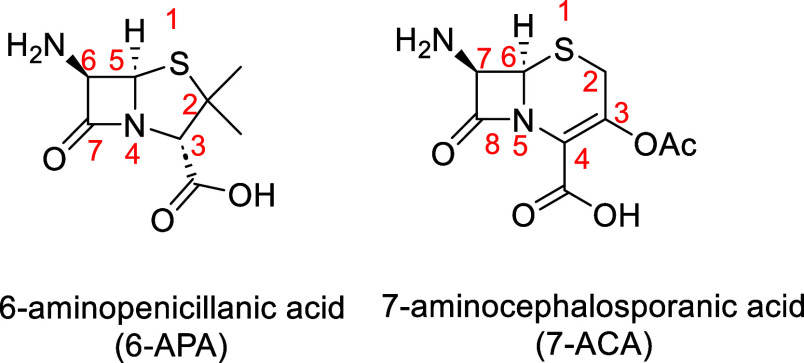
6-APA and 7-ACA antibiotics show the skeleton numbering.

In this context, we devised an alternate approach to coordinate the intact bicyclic cepham or penam system (Figure 1) to a transition metal, namely, to use C–H activation reactions reported previously by us in these systems.^18^ In contrast with our previous works, the target of the actual work was to directly coordinate the β-lactam four-membered ring to the metal and to obtain X-ray structures of these complexes. To the best of our knowledge, X-ray crystal data of such complexes are currently unknown and may be of interest for elucidating the mode of action of mβls and for designing new inhibitors of these enzymes.^19^

**Scheme 2:**
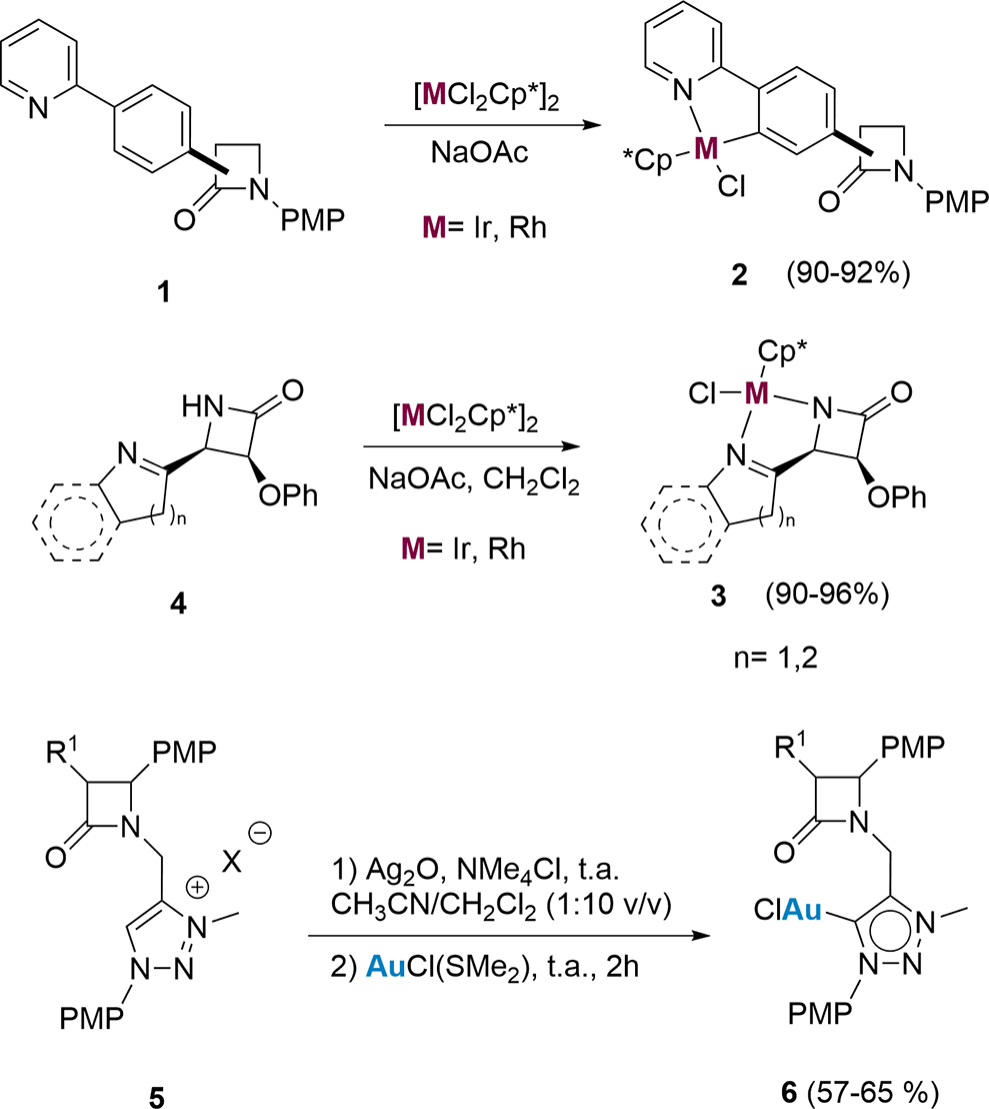
Some Examples of Syntheses of Metallo-β-Lactams

Our previous work used 2-azetidinones with 2-phenylpyridine in the four-membered ring (**1**; Scheme 2). Reactions of these substrates with [MCl_2_Cp*]_2_ (M = Ir, Rh; Cp* = η^5^-C_5_Me_5_) in the presence of NaOAc formed the corresponding metallo-2-azetidinones **2** as diastereomeric mixtures. It should be noted that the β-lactam ring survives these reaction conditions. Additionally, we reported the formation of several metallotrinems **3** through the activation of the lactam N–H bond, directed by a heteroaromatic ring attached to the C4 of the four-membered ring (Scheme 2). Compounds **3** are the bio-organometallic analogues of trinems like sanfetrinem or 6-(1-hydroxyethylcyclonorcardicine).^20^ Other work placed several chromium(0) carbene moieties in groups attached to the carboxy or amino moieties of the cepham and penam systems.^21^ Very recently, we incorporated mesoionic carbene (MIC) ligands bearing 2-azetidinone, penam, and cepham moieties into Au(I), Ni(II), and Pd(II) complexes **6** and determined their catalytic activity in several cycloisomerizations, hydrosilylations, and Suzuki couplings.^22^

## Results and Discussion

**Scheme 3:**
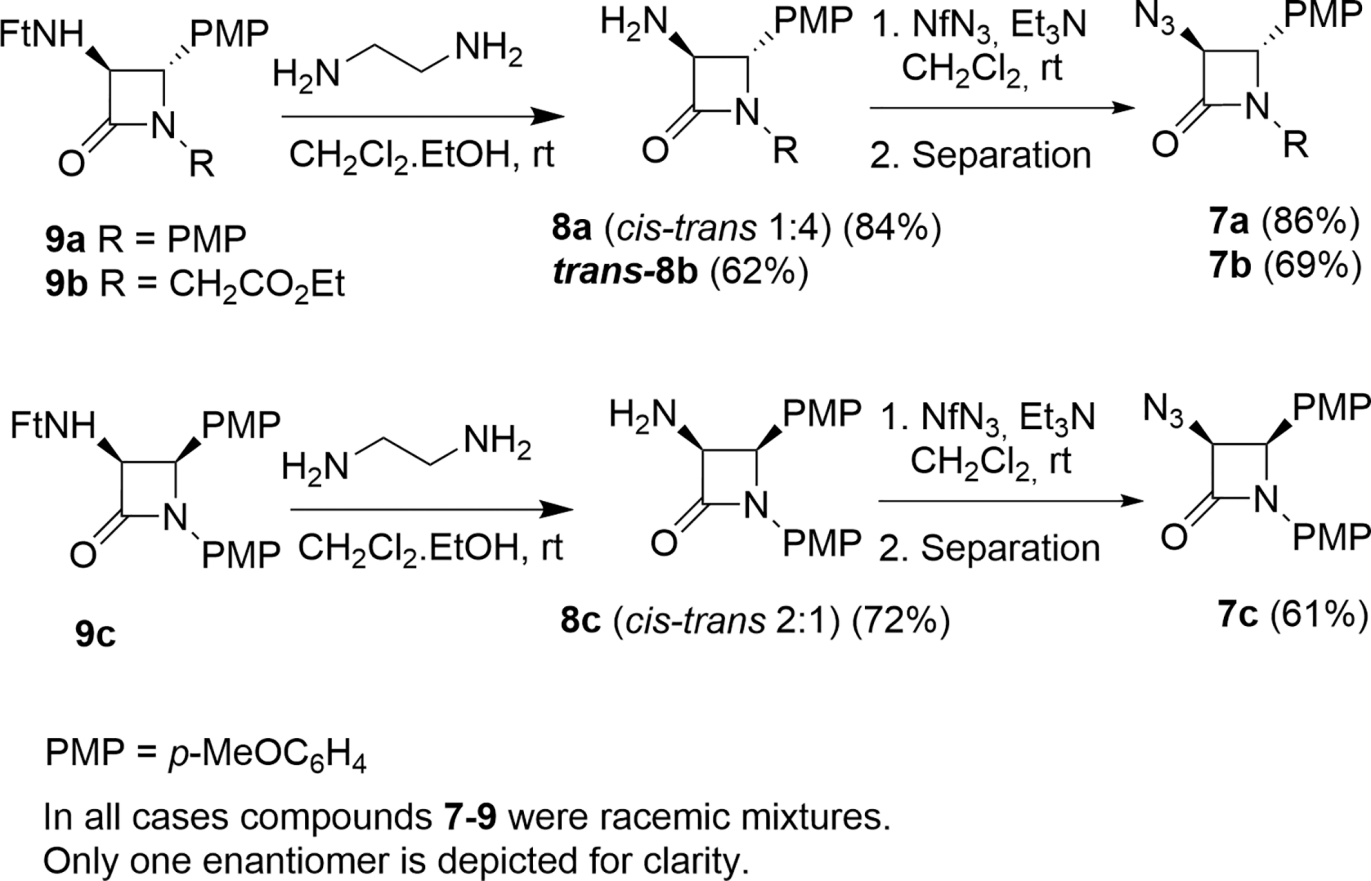
Synthesis of 3-Azido-2-azetidinones **7**

To test the possibility of incorporating a metal next to the 2-azetidinone ring, we prepared the 3-azido-β-lactams **7** from the corresponding 3-amino-2-azetidinones **8** by reacting them with nonaflyl azide in dichloromethane in the presence of Et_3_N.^23^ 2-Azetidinones **8** were obtained in good yields. Both cis- and trans-isomers were accessible from the corresponding 2-azetidinones, which in turn were prepared by deprotection of the corresponding 3-*N*-phthalimidoyl-2-azetidinones **9** with ethylendiamine (Scheme 3).^24,25^ The starting β-lactams **9** were obtained by standard reactions of phthalimidoyl chloride with the corresponding imines, making use of the appropriate reaction conditions to access exclusively the cis- or trans-isomers.^26^

**Scheme 4:**
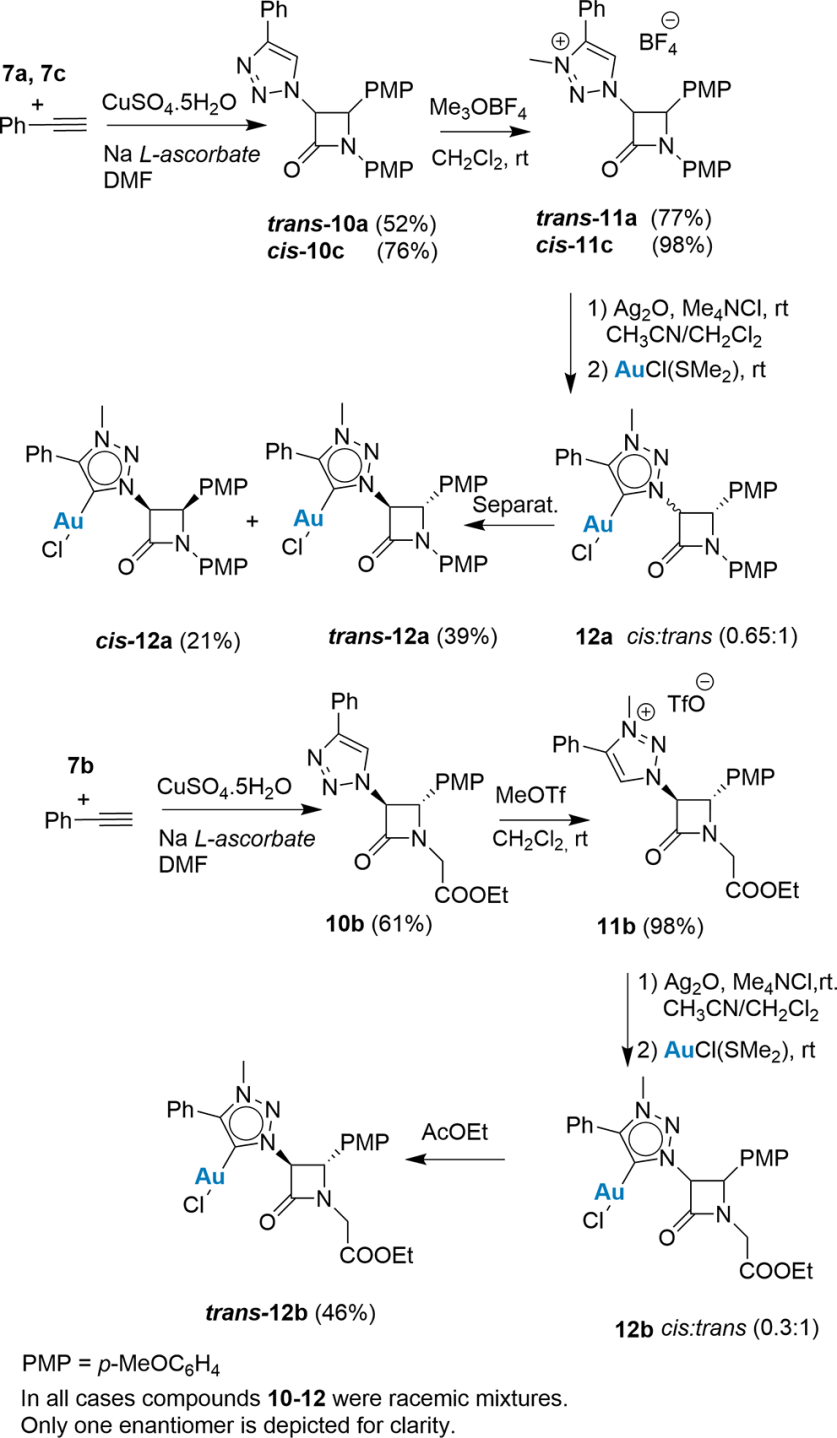
Syntheses of β-Lactam MIC–Au(I) Complexes **12**

Azides **7** were transformed into 1,2,3-triazoles **10** and the corresponding triazolium salts **11** via a Cu-catalyzed azide–alkyne cycloaddition (CuAAC),^27^ in good yields and with retention of both the integrity and the stereochemistry of the four-membered ring. The methylation of 1,2,3-triazoles **10** deserves some comments. 2-Azetidinone **10b** reacted with [Me_3_O]BF_4_ but exclusively led to degradation of the four-membered ring. However, the use of MeOTf (CH_2_Cl_2_/10 °C) rendered the desired *trans*-**11b** in 98% yield. The formation of 1,2,3-triazoles **10** and 1,2,3-triazolium salts **11** was determined by spectroscopic means. Especially relevant are the appearance of the signal attributable to the *N*-Me moiety in the salts (**11a**–**11c**) and the deshielding of the signals corresponding to the 1,2,3-triazole ring upon methylation. Finally, the stereochemical integrity of the salts **11a**–**11c** was ensured by the values of *J*_3,4_ = 2.0 Hz for compound **11a** corresponding to a trans-arrangement of the hydrogens in C^3^ and C^4^ of the four-membered ring, while *cis*-**11c** showed a *J*_3,4_ = 5.4 Hz characteristic of a cis-arrangement in the four-membered ring (Scheme 4).

Metalation of proligands **11** was next pursued. Thus, 1,2,3-triazolium salts **11** were subjected to the standard Ag(I) to Au(I) transmetalation sequence by reacting 2-azetidinones **11** with Ag_2_O in the presence of Me_4_NCl to form the corresponding MIC–Ag(I) complexes, which were reacted with AuCl(SMe_2_) to form the MIC–Au(I)Cl complexes **12**.^28^ In all cases, partial cis–trans isomerization of the β-lactam ring occurred. Thus, compounds **11a** and **11c** formed, independently of the stereochemistry of the starting material, a 0.65:1 cis–trans mixture from which both diastereomers could be separated by silica-gel column chromatography to yield pure *cis***-12a** and *trans*-**12a** in 21 and 39% yields, respectively. Similarly, *trans*-**11b** formed the corresponding MIC–AuCl complex **12b** as a 0.3:1 cis–trans mixture from which pure *trans*-**12b** could be isolated in 46% yield (Scheme 4). While the base-induced cis–trans isomerization of the 2-azetidone ring has been repeatedly reported,^29^ the inverse isomerization is less common and, in this case, is related to the mechanism of metalation of the 2-azetidinone ring (see below).

**Figure 2:**
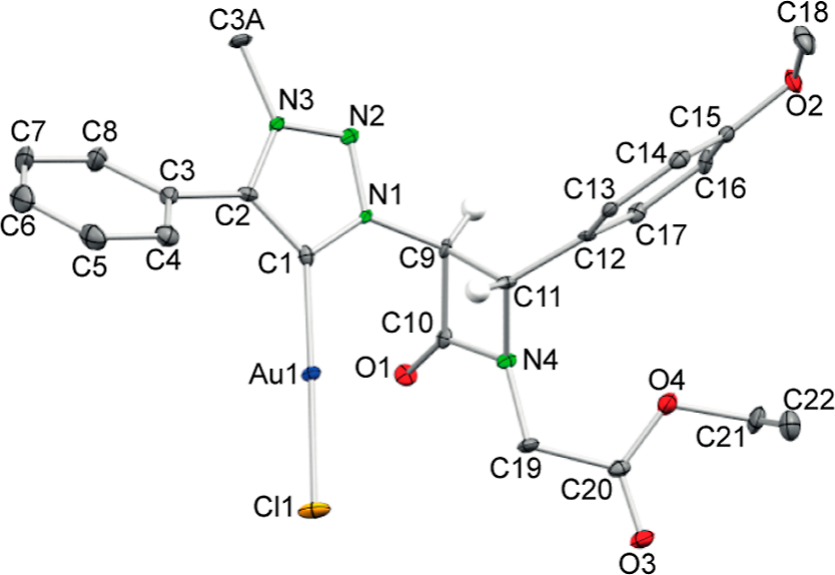
ORTEP view of complex *trans*-**12b** (ellipsoids at 40%; H atoms have been omitted for clarity except those bonded to C9 and C11). Selected interatomic distances (Å) and angles (°): C1–N1 1.367(5), C1–C2 1.380(5), C1–Au1 1.980(4), C2–N3 1.362(5), C3A–N3 1.468(4), C9–N1 1.455(4), C9–C10 1.548(5), C9–C11 1.568(5), C10–O1 1.211(4), C10–N4 1.358(5), C11–N4 1.494(4), Au1–Cl1 2.2817(9), N1–N2 1.337(4), N2–N3 1.318(4); N1–C1–C2 102.3(3), N1–C1–Au1 125.3(3), C2–C1–Au1 132.4(3), N3–C2–C1 107.4(3), N1–C9–C10 115.8(3), N1–C9–C11 116.8(3), C10–C9–C11 86.0(3), O1–C10–N4 133.2(4), O1–C10–C9 135.7(3), N4–C10–C9 91.1(3), N4–C11–C9 85.5(3), C1–Au1–Cl1 178.5(2), N2–N1–C1 114.9(3), N3–N2–N1 103.0(3), N2–N3–C2 112.4(3), C10–N4–C11 96.2(3).

Complexes **12** were spectroscopically characterized. Especially relevant is the disappearance of the signal corresponding to H^5^ of the 1,2,3-triazole ring at 8.25–8.69 ppm in the 1,2,3-triazolium precursors **11**. The stereochemistry of the MIC–Au(I) complexes was assigned as indicated above based on the values of the *J*_3,4_ couplings.^30^ Thus, complex *cis*-**12a** shows a *J*_3,4_ = 5.4 Hz, while complexes *trans*-**12a** and *trans*-**12b** show a *J*_3,4_ = 2.2 and 2.3 Hz, respectively. Moreover, crystals of MIC–Au(I) complex **12b** were grown from an EtOAc/hexane mixture, and its structure was determined by X-ray diffraction (Figure 2). In this figure, the 1,2,3-triazole C^5^ atom is labeled as C1.

**Scheme 5:**
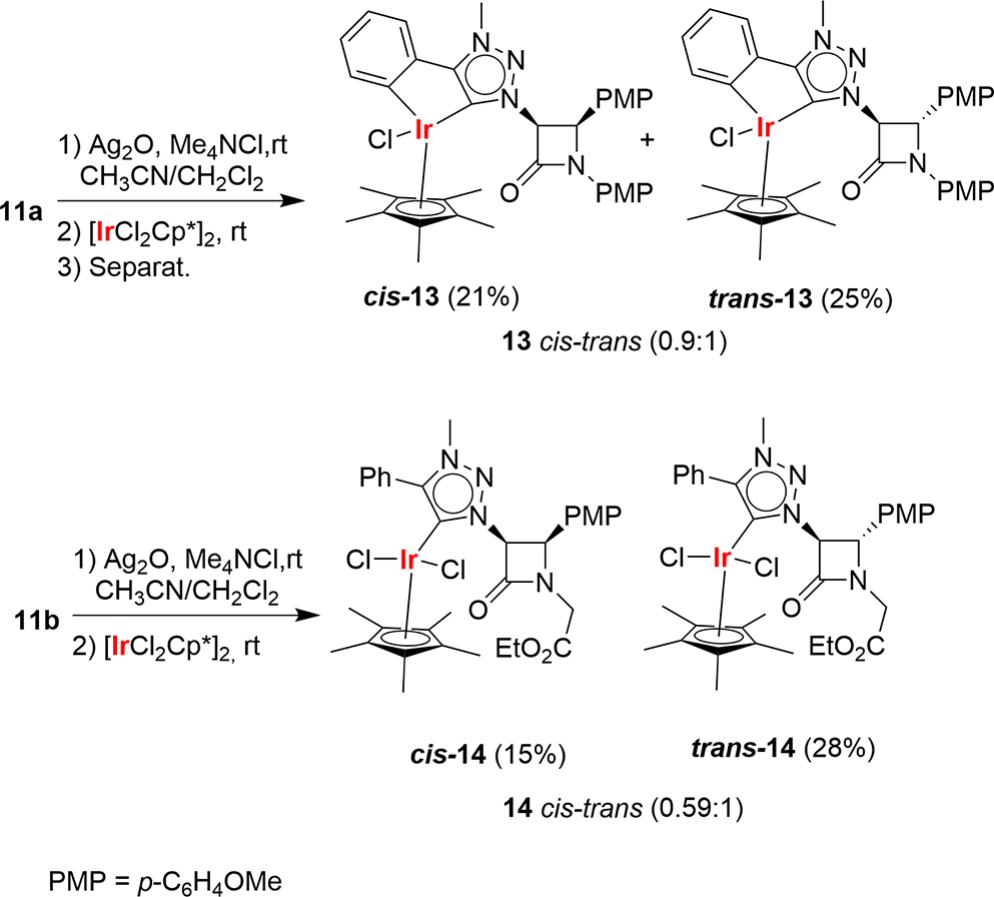
Syntheses of β-Lactam MIC–Ir(III) Complexes **13** and **14**

Aiming at promoting the C–H activation of either the aromatic ring bonded to C^4^ of the 1,2,3-triazole ring or, alternatively, the unknown C–H insertion into the C^3^–H bond of the 2-azetidinone ring, proligand **11a** was reacted with [IrCl_2_Cp*]_2_. As in the synthesis of MIC-Au(I) complexes **12**, the transmetalation of the MIC-Ag(I) intermediate complex derived from *trans*-**11a** formed a mixture of *cis*- and *trans*-β-lactams **13**. Under analogous conditions, proligand **11b** formed complex **14**, in which C–H insertion did not occur. Complexes **13** could be separated by column chromatography. Similarly, complexes **14** were also separated and characterized. It should be noted that 2-azetidinone **11a** could give a diastereomeric mixture of at least 3 compounds due to the new chiral Ir center (Scheme 5); however, only two racemic diastereomers were formed, which confirms the stereoselectivity of the formation of complexes **13**.^31^

Results in Scheme 5 show that the Ir atom either inserts into the aromatic C–H bond (complexes **13**) or coordinates the MIC without further insertion (complexes **14**), but in both cases, the H^3^ of the 2-azetidinone ring is epimerized. This is demonstrated by the four-membered-ring *J*_3,4_ coupling values. Probably, the isomerization occurs during the formation of the intermediate MIC–Ag(I) complex because Ag_2_O may act as a base.

**Scheme 6:**
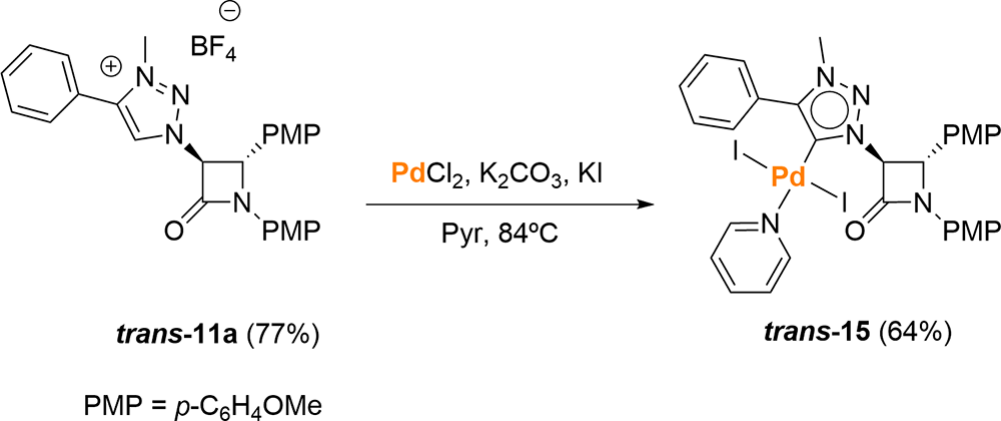
Synthesis of β-Lactam MIC–Pd(II) Complex **15**

To further support this hypothesis, the MIC–Pd(II) complex was prepared from 1,2,3-triazolium salt **11a** without the intermediacy of the silver complex. Thus, the reaction of **11a** with PdCl_2_ in the presence of K_2_CO_3_ yielded MIC complex **15**, which keeps the trans-stereochemistry of the starting material unaltered, as demonstrated by the value of *J*_3,4_ = 2.0 Hz (Scheme 6). Then, it can be safely concluded that the metalation of salt **11** with Ag_2_O is responsible for the isomerization of the 2-azetidinone ring.

The above results clearly show that the metalation of 3-(1,2,3-triazolyl)-2-azetidinones is compatible with the structural integrity of the four-membered ring. However, when a sequential transmetalation process is used, going from Ag(I) to another metal, the stereochemical arrangement of the four-membered ring changes. This change in the reactivity may be due to the basic character of Ag_2_O.

Penam and cepham derivatives were next studied. Beginning with commercial 6-aminopenicillanic acid (6-APA) derivatives, they were transformed into tosylate **16** following the reported methodology, either directly (*p*-nitrobenzyl ester **16b**)^32^ or sequentially [protection of the amino group of APA with ethyl acetoacetate (acac),^33^ followed by treatment with benzyl bromide and elimination of the acac group with TsOH].

**Figure 3:**
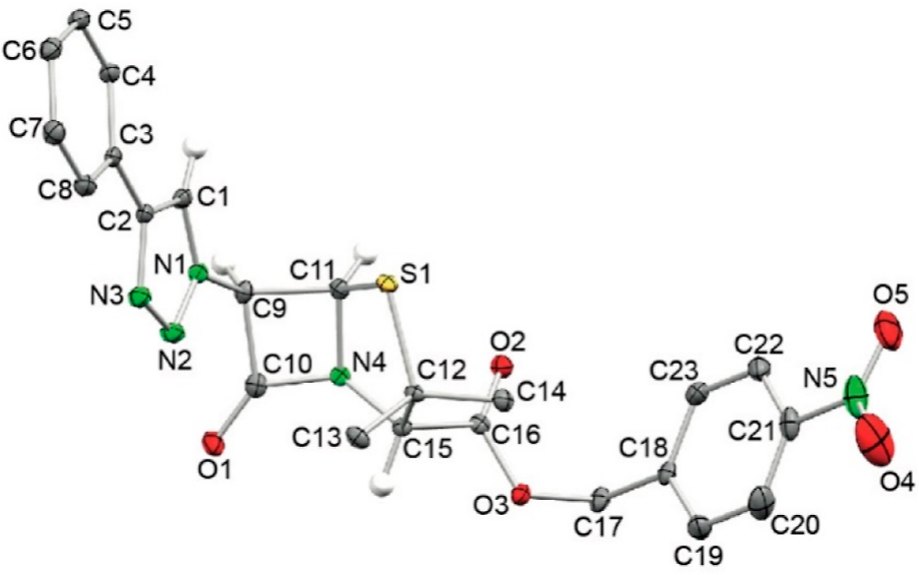
ORTEP view of compound **18b** (ellipsoids at 40%; H atoms have been omitted for clarity except those bonded to C1 C9, C11, and C15). Selected interatomic distances (Å) and angles (°): C1–N1 1.352(3), C1–C2 1.371(3), C2–N3 1.370(3), C9–N1 1.445(3), C9–C10 1.556(3), C9–C11 1.562(3), C10–O1 1.197(3), C10–N4 1.385(3), C11–N4 1.467(3), C11–S1 1.810(2), C12–C15 1.576(3), C12–S1 1.856(2), C15–N4 1.447(3), C15–C16 1.519(3), C16–O2 1.205(3), C16–O3 1.343(2), N1–N2 1.353(3), N2–N3 1.312(3); N1–C1–C2 104.6(2), N3–C2–C1 108.3(2), N1–C9–C10 113.5(2), N1–C9–C11 116.8(2), C10–C9–C11 85.0(2), O1–C10–N4 132.2(2), O1–C10–C9 136.5(2), N4–C10–C9 91.3(2), N4–C11–C9 88.0(2), N4–C11–S1 105.7(1), C9–C11–S1 120.2(1), C14–C12–C15 113.8(2), C15–C12–S1 105.7(1), N4–C15–C12 106.3(2), C1–N1–N2 111.0(2), C1–N1–C9 127.3(2), N2–N1–C9 121.3(2), N3–N2–N1 107.0(2), N2–N3–C2 109.1(2).

**Scheme 7:**
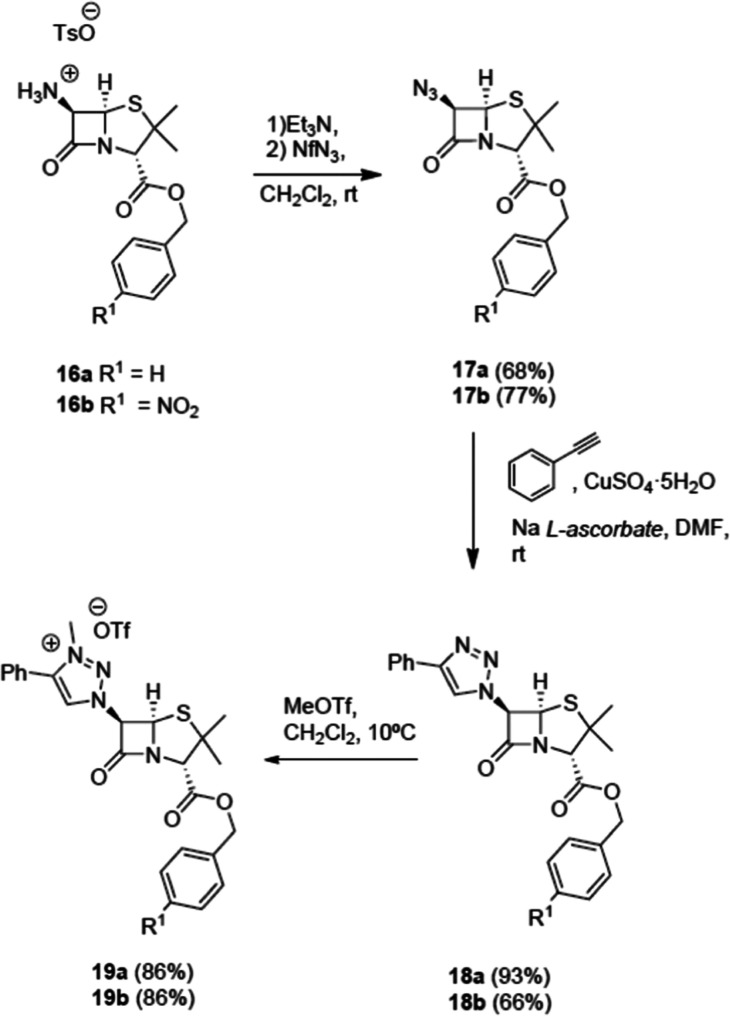
Syntheses of Proligands **19** Derived from 6-APA

Treatment of salts **16** with Et_3_N followed by the reaction of the free base with nonaflyl azide (NfN_3_) formed 6-azido derivatives **17** in good yields. Azides **17** were reacted with phenylacetylene in the presence of Cu(I) to form the corresponding 1,2,3-triazoles **18** in good yields, which upon methylation (MeOTf) formed the corresponding 1,2,3-triazolium salts **19**. The structure of the 1,2,3-triazole derivatives **19** was stablished by spectroscopic grounds and, in the case of compound **18b**, by X-ray diffraction (Figure 3), which also confirms that the cycloaddition reaction has selectively occurred in such a way that the terminal alkyne C^1^ atom has ended attached to the internal N atom of the azide precursor (Scheme 7).

**Scheme 8:**
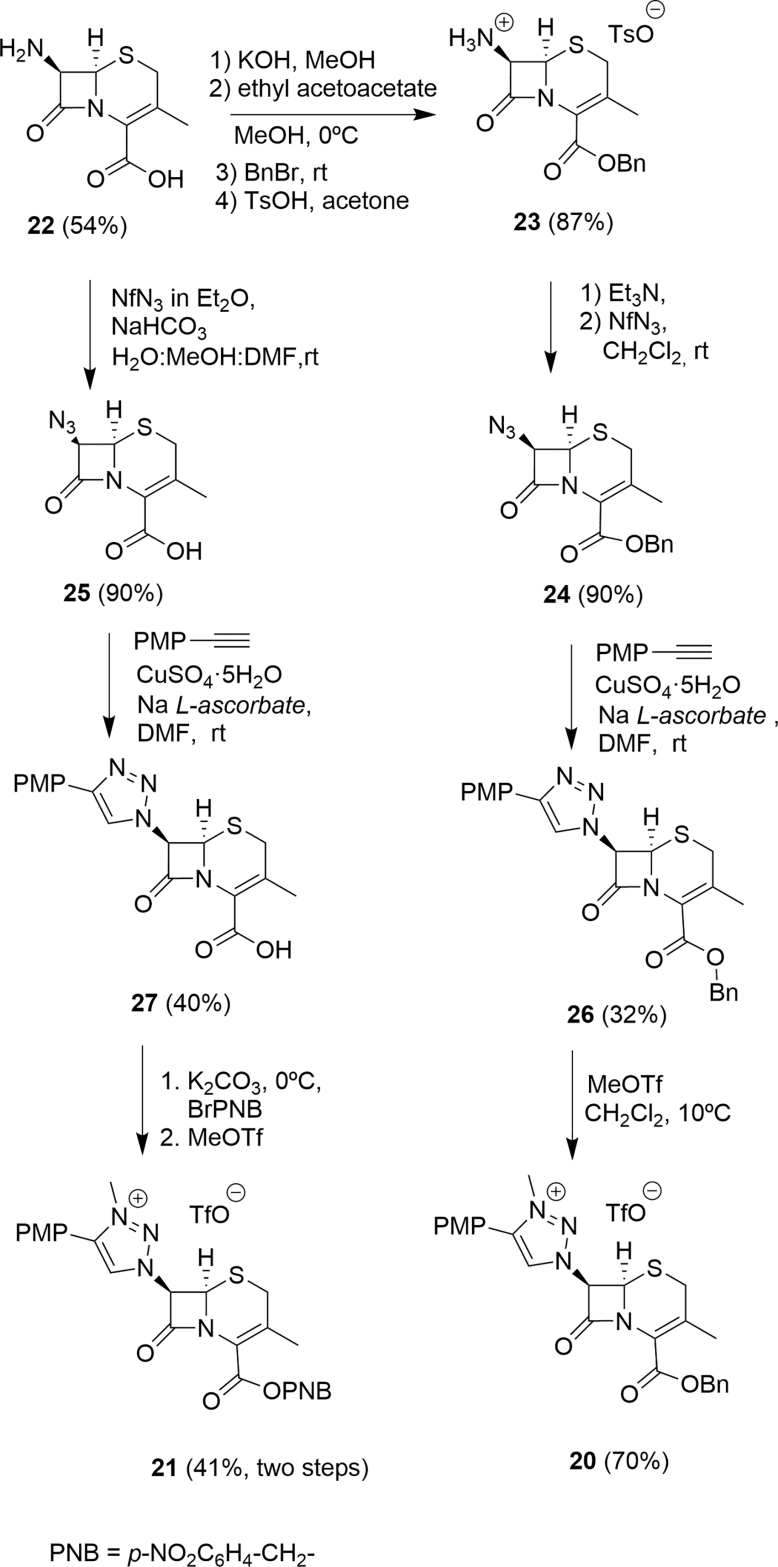
Syntheses of Proligands **20** and **21** Derived from 7-ACA

Cephalosporin-based proligands **20** and **21** were prepared from 7-aminocephalosporanic acid (7-ACA). The syntheses of these derivatives were achieved by following two approaches. Reduction of the 3-AcO group of 7-ACA was carried out by reaction with Et_3_SiH in the presence of BF_3_·Et_2_O in a TFA solution to yield compound **22**.^34^ Compound **22** was then protected with ethyl acetoacetate and alkylated with benzyl bromide. Elimination of the amino protecting group with TsOH formed tosylate **23**, which upon treatment with Et_3_N and NfN_3_ gave the 7-azido derivative **24** (Scheme 8). In parallel, a solution of compound **22** in MeOH/H_2_O/DMF was treated with NfN_3_ in Et_2_O solution to afford 7-azidocephalosporanic acid **25** in 90% yield. Both azides, **24** and **25**, were reacted with *p*-MeOC_6_H_4_CCH under the standard CuAAC conditions, forming 7-1,2,3-triazole-cepham derivatives **26** and **27**. To avoid complications with the free COOH group during the methylation process, the free acid of triazole **27** was esterified with *p*-nitrobenzyl bromide. Ester derivatives were finally methylated with MeOTf to render 1,2,3-triazolium salts **20** and **21** (Scheme 8).

Metalation of penam-based proligands **19** in the presence of Ag_2_O followed by transmetalation with [IrCl_2_Cp*]_2_ led to decomposition mixtures. However, reactions of salts **19** with [IrCl_2_Cp*]_2_ in the presence of Cs_2_CO_3_ at room temperature formed the new metal complexes **28a** and **28b**, which were isolated in 44 and 54% yields, respectively. Both were single enantiomers.

**Scheme 9:**
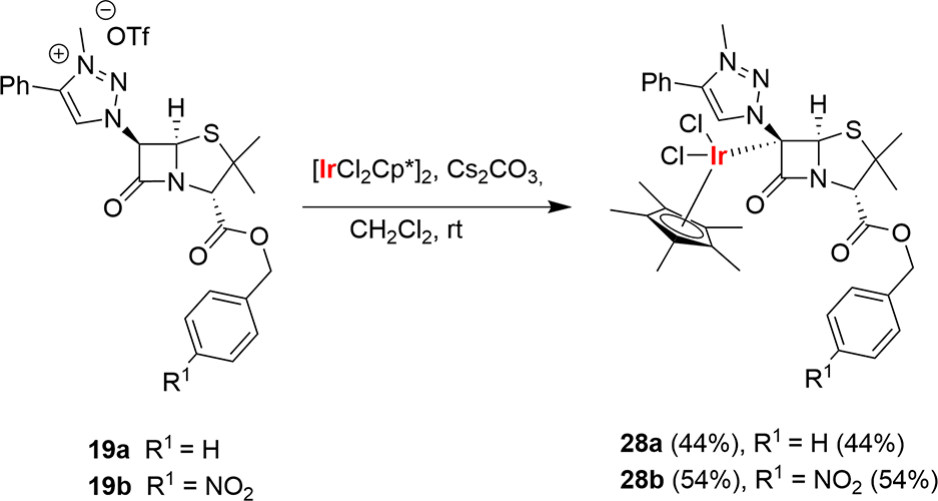
Synthesis of C^6^–Ir(III) Complexes with a Penam Bicyclic System

The structures of these new complexes were determined by spectroscopic and spectrometric means. Thus, both complexes show a singlet in their ^1^H NMR spectra at around 9 ppm that can be assigned to the 1,2,3-triazole ring proton. Moreover, only two signals were observed for the 2-azetidinone ring (instead of the three signals of the starting material). These signals are singlets (δ = 5.94 and 4.52 ppm for **28a** and δ = 6.06 and 4.56 ppm for **28b**). HRMS demonstrated that both complexes lack one hydrogen with respect to the starting material as well as the presence of the Ir moiety in the molecules. These findings are congruent with the coordination of the Ir atom to the C^6^ atom of the penicillin bicyclic system. Both the bicyclic [4,5]-system of the penicillin and the 1,2,3-triazolium moiety remained unaltered (Scheme 9).

**Figure 4:**
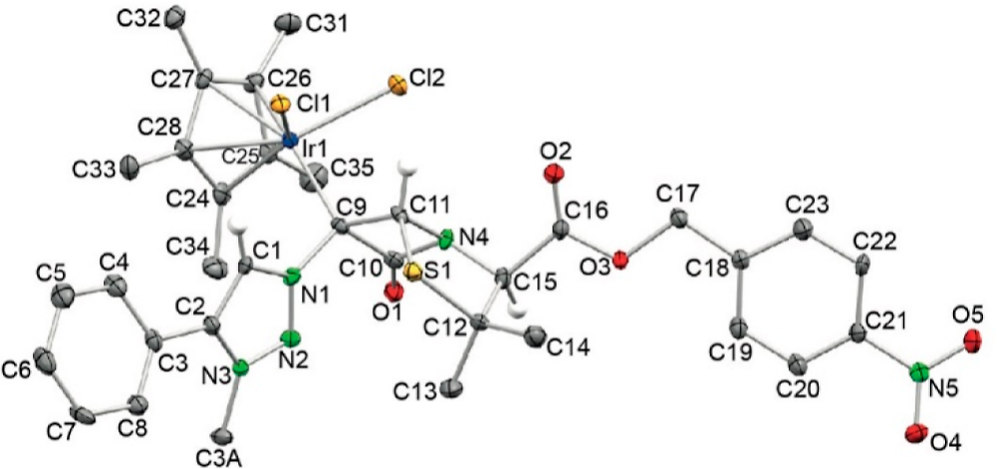
ORTEP view of complex **28b** (ellipsoids at 40%; H-atoms have been omitted for clarity, except those bonded to C1, C11, and C15). Selected interatomic distances (Å) and angles (°): C1–N1 1.35(1), C1–C2 1.38(1), C2–N3 1.37(1), C3A–N3 1.47(1), C9–N1 1.47(1), C9–C10 1.54(1), C9–C11 1.57(1), C9–Ir1 2.122(9), C10–O1 1.21(1), C10–N4 1.39(1), C11–N4 1.47(1), C11–S1 1.835(9), C12–C15 1.57(1), C12–S1 1.861(8), C15–N4 1.44(1), C15–C16 1.53(1), C16–O2 1.19(1), C16–O3 1.35(1), C24–C28 1.44(1), C24–Ir1 2.159(8), C25–Ir1 2.136(9), C26–Ir1 2.223(9), C27–Ir1 2.224(8), C28–Ir1 2.146(9), Cl1–Ir1 2.437(2), Cl2–Ir1 2.406(2), N1–N2 1.31(1), N2–N3 1.33(1); N1–C1–C2 105.6(8), N3–C2–C1 104.4(8), N1–C9–C10 110.9(7), N1–C9–C11 111.9(7), C10–C9–C11 84.1(6), N4–C10–C9 92.9(7), N4–C11–C9 88.4(6), N4–C11–S1 104.8(5), C14–C12–S1 108.4(7), C15–C12–S1 104.5(6), N4–C15–C12 107.0(7), N2–N1–C1 113.1(7), C9–N1–C1 125.1(7), N2–N1–C9 121.8(7), N3–N2–N1 104.3(7), N2–N3–C2 112.5(7), C10–N4–C15 127.8(7), C10–N4–C11 93.4(6), C15–N4–C11 117.5(6).

Crystals of complex **28b** were grown from CH_2_Cl_2_ and analyzed by X-ray diffraction. The corresponding crystallographic data (Figure 4) confirm that the Ir atom is attached to the C^6^ atom (C^9^ in Figure 4) of the penicillin bicycle and not to the 1,2,3-triazole carbene-like atom of the analogous monolactams. In this complex, the four-membered ring acts as a two-electron donor. It is important to remark that a penicillin derivative having a metal directly bonded to the intact [4,5] bicycle is unprecedented and that this crystal structure (Figure 4) is also the first one to show a metal complex having the metal atom bonded to the penicillin bicycle.

**Scheme 10:**
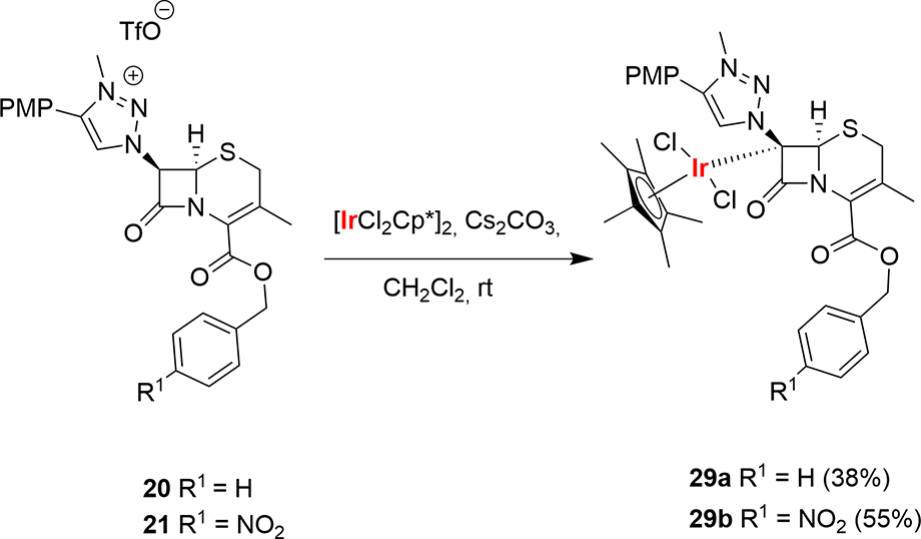
Syntheses of C7–Ir(III) Complexes with a Cepham Bicyclic System

7-(1,2,3-Triazolium)-cephalosporin derivatives **20** and **21** were next reacted with [IrCl_2_Cp*]_2_ under conditions analogous to those used for the synthesis of complexes **28**. New complexes **29a** and **29b** were obtained in 38 and 55% isolated yields, respectively, after column chromatography. The spectroscopic data (NMR) of complexes **29** were analogous to those of penam-derived complexes **28**. Thus, the H^5^ of the 1,2,3-triazolium moiety appears at 9.46 ppm, and complexes **29** show one single signal attributable to the four-membered ring of the [4,6] bicyclic system. The ^13^C NMR signal at 129 ppm confirms the integrity of the 1,2,3-triazole C–H bond. Finally, HRMS of complexes **29** shows the presence of the Ir-center and the loss of one hydrogen. Thus, we can conclude that complexes **28** and **29** share a common structure (Scheme 10). It should be noted again that complexes **29** are the first M-cephalosporin complexes with a metal atom bonded to the intact bicyclic system.^35^

**Scheme 11:**
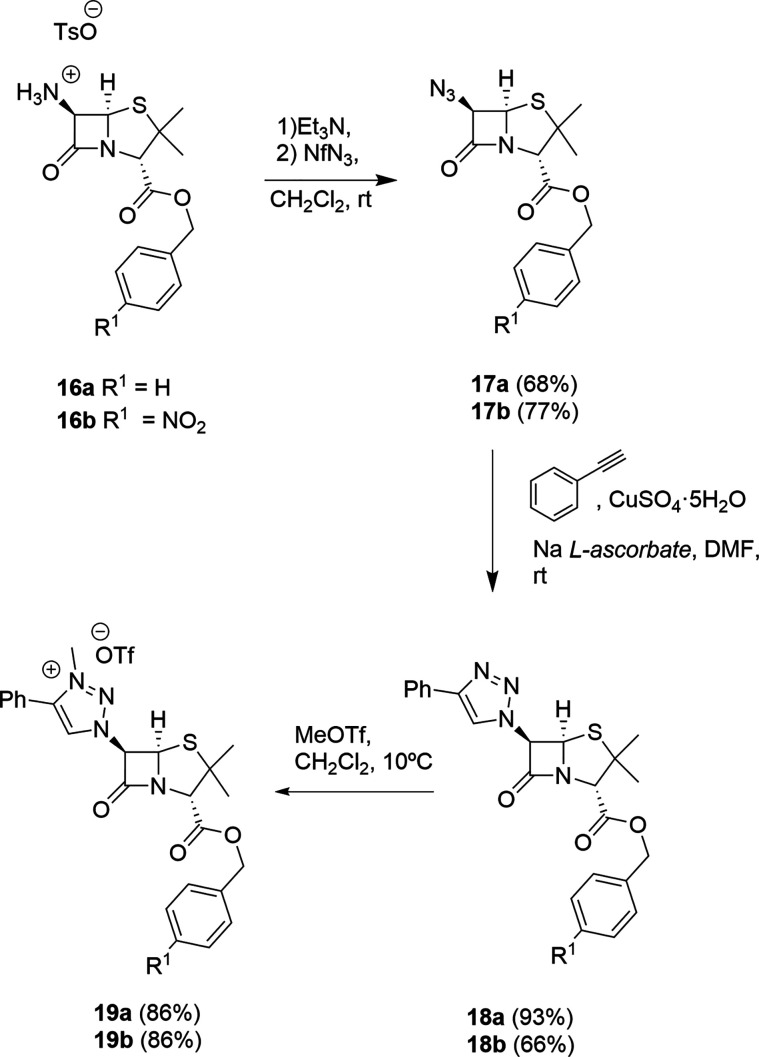
Synthesis of Complex **30**

Finally, we tested the possibility of extending the coordination of proligand **19a** to Au(I). Reaction of 1,2,3-triazolium salt **19a** with [AuCl(SMe_2_)] in the presence of K_2_CO_3_ afforded complex **30** in 51% yield after column chromatography. Complex **30** shows a distinctive singlet signal in its ^1^H NMR spectrum at 8.71 ppm, corresponding to the H^5^ of the 1,2,3-triazolium ring. Similar to the behavior observed for complexes **28**, penam complex **30** also lacks the H^6^ signal of the penam system. HRMS confirmed the coordination of the Au atom to the system as well as the absence of one hydrogen. Therefore, proligand **19a** is able to coordinate Au(I) at the C^6^ of the four-membered ring, similar to Ir in complexes **28** and **29** (Scheme 11).

The reactions involving penams and cephams having a 1,2,3-triazolyl moiety attached to, respectively, the 6- and 7-positions of their bicyclic structures revealed interesting features. Contrary to expectations, deprotonation of the 1,2,3-triazolium salt did not lead to the MIC (metal-bound intermediate carbene) complex. Instead, the metal preferred to bind the four-membered ring of the compound. This behavior stands in stark contrast to mono-β-lactams **11**, which form MIC complexes, with or without C–H insertion.

In this regard, the penam and cepham ligands in complexes **28** and **29** are new classes of two-electron-donor ligands, with almost no contribution of the amide system to the coordination of the metal. Thus, in the X-ray structures, the amide CO bond in complex **28b**, 1.21(1) Å, is only slightly longer than that of proligand **18b**, 1.197(3) Å.

**Scheme 12:**
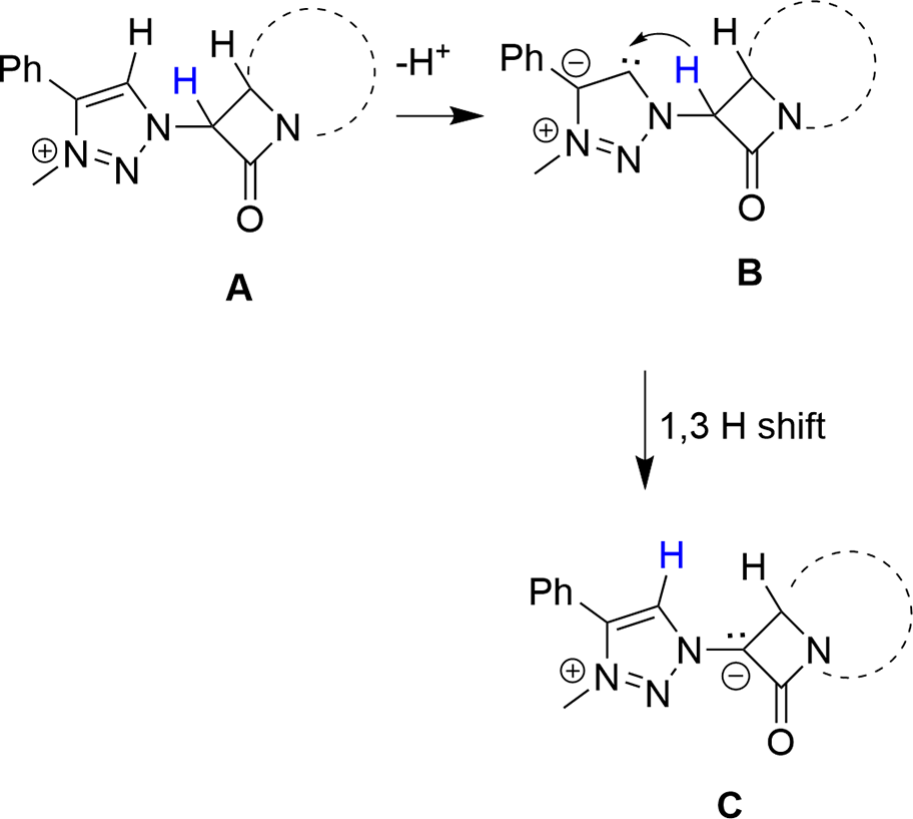
1,3-H Shift to Form the Carbene Proligand in the Four-Membered Ring from the Initial Deprotonation of the 1,2,3-Triazolium Salt

The above results may be rationalized by assuming that an initial deprotonation of the more acidic H^5^ of the 1,2,3-triazole moiety is followed by a 1,3-prototropy of the penam system H^6^ atom or the cepham system H^7^ atom (Scheme 12).

**Scheme 13:**
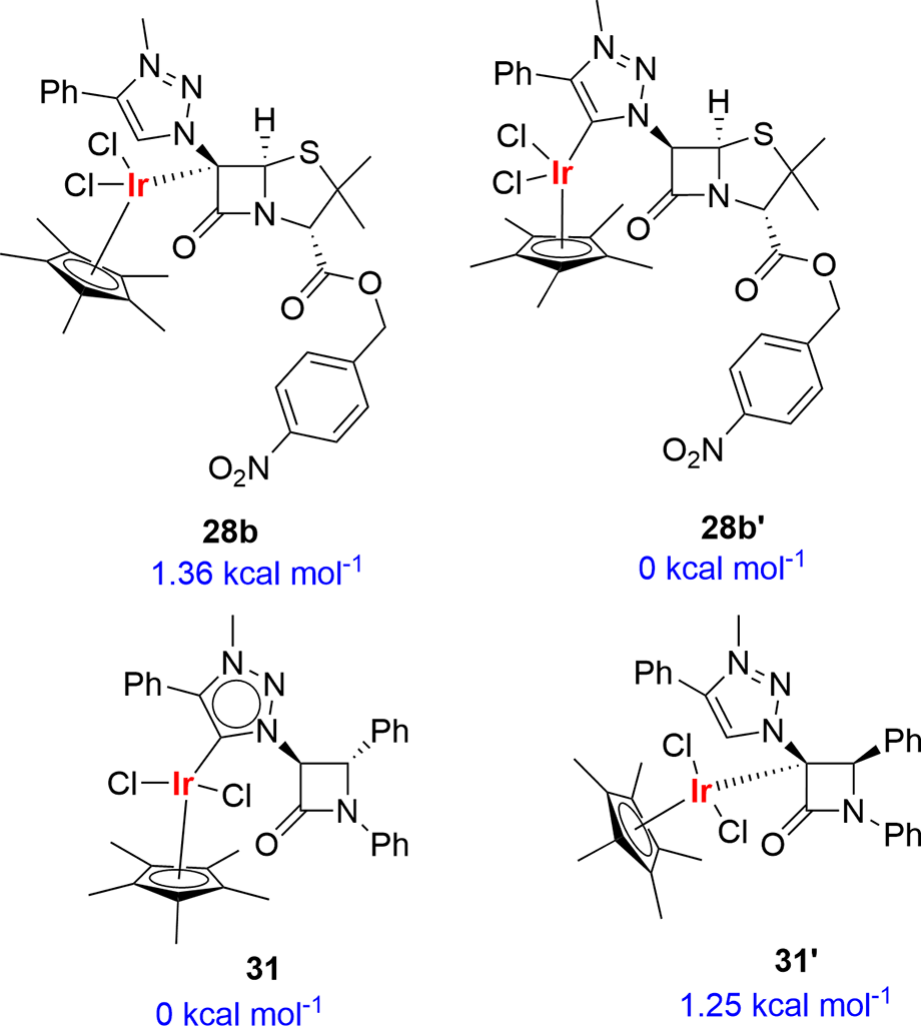
Computed Relative Gibbs Energies^27^ for the Pairs of Carbene Complexes **28b–28b′** and **31**–**31′**

**Scheme 14:**
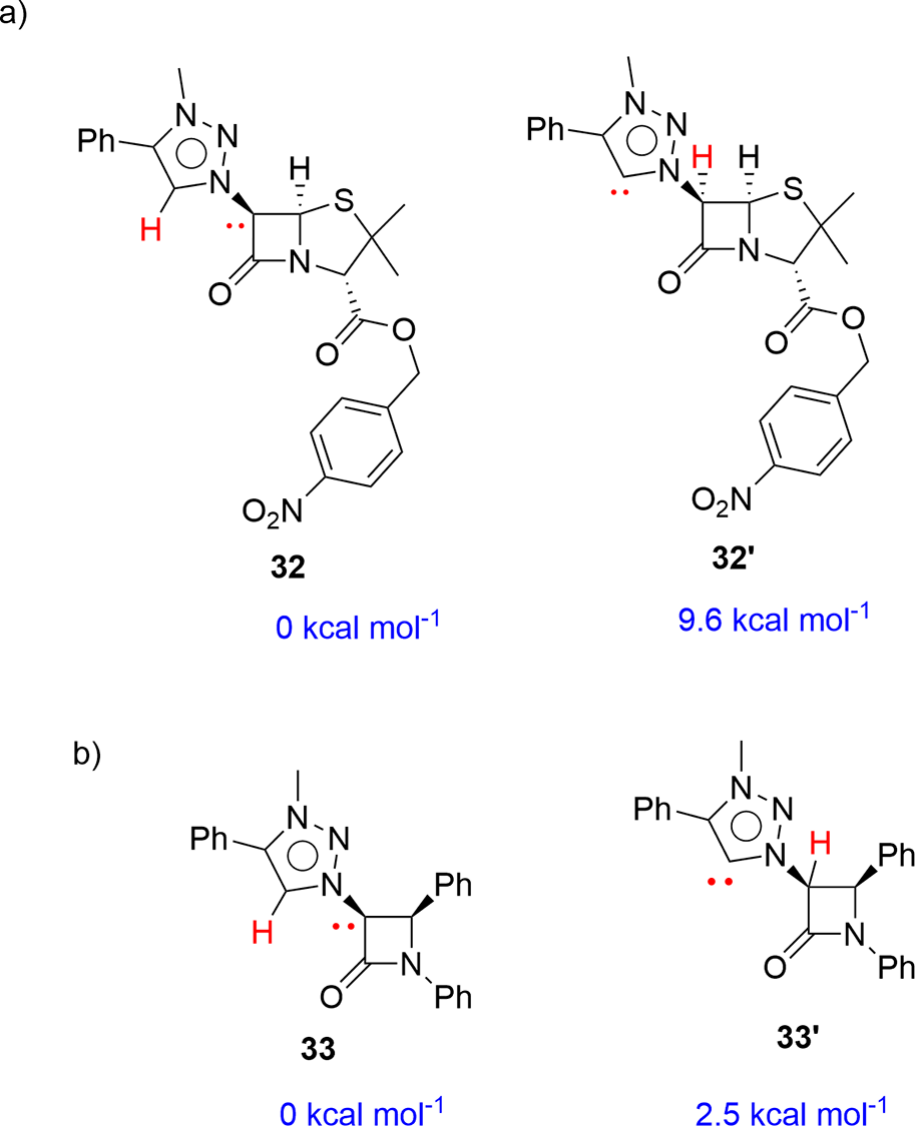
Computed Relative Gibbs Energies^27^ for (a) Carbenes Derived from 6-(1,2,3-Triazolyl)penam **32** and **32′** and (b) Carbenes Derived from 6-(1,2,3-Triazolyl)-2-azetidinone **33** and **33′**

To understand the origin of the different coordination observed for mono-β-lactams (coordination at the C^5^ of the 1,2,3-triazole moiety instead of the C^3^ of the four-membered ring) and for the bicyclic penicillin and cephalosporin derivatives (coordination at C^6^ and C^7^ of the bicyclic ring, respectively), we computed^36^ the Gibbs energies of the complexes derived from both coordination modes, both in 2-azetidinones **31** and **31′** and in penam derivatives **28b** and **28b′**. The obtained results (Scheme 13) show that for monocyclic 2-azetidinones, complexes **31** and **31**′ are nearly isoenergetic, with complex **31**′ (coordination to the C^4^ position) being 1.25 kcal mol^–1^ less stable than **31**, which results from coordination to the C^5^ of the 1,2,3-triazole ring. Complexes **28b** and **28b′** are also nearly isoenergetic, with complex **28b′** being 1.95 kcal mol^–1^ more stable than complex **28b**. Therefore, the selectivity of the metalation reactions cannot be attributed to the stability of the final complexes. Subsequently, we computed the energies of carbene **32** and MIC **32′**. The carbene compound having the lone pair located at the C^7^ position of the bicyclic penam system **32** is 9.16 kcal mol^–1^ more stable than its counterpart with the lone pair at the C^5^ position of 1,2,3-triazole **32**′ (Scheme 14). Therefore, the formation of complexes **28** occurs through the more stable carbene ligand **32**. In addition, monolactam carbene **33** is 2.5 kcal mol^–1^ more stable than C^5^-1,3,3-triazole MIC **33**′. Apparently, the use of Ag_2_O for the transmetalation from the initially formed Ag-MIC to Au and Ir centers favors the formation of the kinetic complexes **12**–**15** (Schemes 5 and 6). It is worth noting that for Pd-complex **15**, the use of similar conditions to those used for the synthesis of complexes **28**–**30** resulted in extensive decomposition. Unfortunately, this decomposition prevents a conclusive determination of whether the C^3^-complex would form under these conditions.

## Conclusions

We developed synthetic procedures to prepare transition metal complexes containing intact bicyclic cepham or penam systems as ligands. Our synthetic approach used 3-azido-2-azetidinones, which are readily available from reactions of phthalimidoyl chloride and imines as ligand precursors. Removal of their amino protecting group and reaction of the free amine with NfN_3_ was followed by a CuAAC between the 3-azido-2-azetinones and alkynes, which yielded the corresponding 3-(1,2,3-triazolyl)-2-azetidinones. Upon methylation, the latter led to proligands **11**. Their treatment with Ag_2_O followed by transmetalation to Au(I) and Ir(III) complexes formed MIC complexes **12**–**14** in good to acceptable yields. The direct reaction of the proligand with PdCl_2_ in the presence of pyridine and K_2_CO_3_ afforded Pd(II) complex **15** in lower yield due to extensive decomposition. Interestingly, epimerization of the 2-azetidinone ring occurred during the transmetalation process as a consequence of the isoenergetic character of the cis- and trans-isomers, which was unexpected. The epimerization process also occurred during deprotection of the C^3^-phthalimido group for analogous reasons.

Extending this methodology to 6-azido penam and 7-azido cepham derivatives rendered the corresponding 6-(1,2,3-triazolyl) penam and 7-(1,2,3-triazolyl) cepham proligands. Metalation of these proligands [with Au(I) and Ir(III)] yielded complexes, resulting from the coordination of the metal to the C^6^-penam and C^7^-cepham positions. Notably, the bicyclic structure of the penicillin and cephalosporin derivatives remained intact. The crystal structure of complex **28b**, with the Ir atom directly bonded to the intact penicillin bicycle, was determined. It should be noted that this is the first report of the structure of a transition metal complex having the bicyclic system of these antibiotics intact. The current lack of crystal structures of metallo β-lactams with unhydrolyzed substrates confers our finding relevance for understanding enzyme–substrate interactions and designing new mβl inhibitors.

DFT calculations were carried out to understand the differences observed between mono- and bicyclic 2-azetidinones in the metalation reactions. The selectivity of the metalation processes is not attributable to significant differences in the stability of the final complexes since these are essentially isoenergetic. Accordingly, we performed computations on their precursors, carbenes **32** and **32′**. We found that the molecule with the carbene located at the C^6^ position of the bicyclic penam system is 9.16 kcal mol^–1^ more stable than its counterpart at the C^5^ position of the 1,2,3-triazole. Consequently, the formation of complexes primarily takes place through the more stable carbene ligand. Monocyclic 2-azetidinone carbenes **33** and **33′** were nearly isoenergetic. In this case, the selectivity of the reaction may be attributable to the more favorable kinetics of the coordination with less stable carbene **33**′.

To summarize, unprecedented coordination of the four-membered ring of β-lactam antibiotics to the metal atom of metal complexes has been achieved, and the structures of two of these new complexes have been determined by X-ray diffraction. The crystallographic data of the Ir(III)-penicillin complex **28b** may be a significant contribution to the development of new active compounds against mβl-producing bacteria. Future work with these complexes will focus on their activity against resistant bacteria strains, for which the stability and deprotection of the carboxylate group have to be determined.

## Supporting Information Available

General procedures, experimental details, characterization data, copies of NMR spectra for all the compounds prepared in this work, and copies of the variable-time ^1^H NMR spectra experiments (PDF)
Atomic coordinates of the DFT-optimized structures (TXT)
