# Supplementary material for: Attaching Metal-Containing Moieties to β-Lactam Antibiotics: The Case of Penicillin and Cephalosporin
Source: Inorg Chem. 2024 Jun 26;63(27):12593–603. doi: 10.1021/acs.inorgchem.4c01548 (PMC11234371; doi:10.1021/acs.inorgchem.4c01548)
Supplement: Supplementary file 1 — ic4c01548_si_001.pdf [file ic4c01548_si_001.pdf]

Supporting Information

Attaching Metal-Containing Moieties to β-Lactam Antibiotics: The case of Penicillin and Cephalosporin

María Moreno-Latorre,[a,d] María C. de la Torre,*[,a,d] Javier A. Cabeza,[b,d] Pablo García-Álvarez,[b,d] Miguel A. Sierra*[c,d]

[a] Instituto de Química Orgánica General. Consejo Superior de Investigaciones Científicas (IQOG-CSIC). Juan de la Cierva 3, 28006 Madrid (Spain).

[b] Departamento de Química Orgánica e Inorgánica, Facultad de Química, Universidad de Oviedo, 33071 Oviedo (Spain).

[c] Departamento de Química Orgánica I, Facultad de Química. Universidad Complutense, 28040 Madrid (Spain).

[d] Centro de Innovación en Química Avanzada (ORFEO-CINQA).

e-mails:
MCT: mc.delatorre@csic.es
MAS: sierraor@ucm.es

Index:

General Methods	pag. S2
Experimental Section	pag. S2
Single Crystal X-Ray Diffraction Analyses	pag. S27
Computational details	pag. S29
References	pag. S29
Copies of NMR spectra	pag. S30-S102
Variable time 1H NMR experiments (Figure S1)	pag. S103-S108

General methods.

Unless noted otherwise, all manipulations were carried out under argon atmosphere using standard Schlenk techniques. DMF, toluene, CH_2Cl_2 and CH_3CN were dried by passage through solvent purification columns containing activated alumina. Other solvents were HPLC grade and were used without purification. All reagents were obtained from commercial sources and used without additional purification, unless noted otherwise. Flash column chromatography was performed using silica gel 60 (Merck, n° 1.09385, 230-400 mesh). ^1H and ^{13}C NMR spectra were recorded at 400 or 500 MHz (^1H NMR) and at 101 or 126 MHz (^{13}C NMR) using $CDCl_3$, MeCN-d_3 or DMSO-d_6 as solvents with the residual solvent signal as internal reference ($CHCl_3$ 7.26 and 77.2 ppm), (Acetonitrile 1.94, and 118.26 and 1.32 ppm), (DMSO, 3.33, 2.5 y 39.52 ppm). The following abbreviations are used to describe peak patterns when appropriate: s (singlet), d (doublet), t (triplet), q (quadruplet), m (multiplet), and br (broad). The NMR peak assignments were based on the analysis of ^1H–^{13}C HMBC and HSQC recorded spectra along with previously reported data for related compounds. High-resolution mass spectrometry (HRMS) by the ESI technique was performed with an Agilent 6500 accurate mass apparatus with a Q-TOF analyser. IR spectra were recorded on a Perkin- Elmer 681 spectrophotometer. Melting points were determined on a Koffler block.

Compounds ***trans*-9a**,[1] ***cis*-9c**[2], ***trans*-9b**[3], ***cis:trans*-III.8a**[4], ***cis:trans*-8c**[4], **16a**[5] and **16b**[6] were prepared according to previously described procedures. The nonafluorobutanesulfonyl azide (NfN_3) and the $[IrCl_2Cp^*]_2$ dimer were synthesized following the procedures described in the literature.[7]

Experimental section

Caution! Even if we have not experienced any explosive or otherwise highly exothermic decomposition event, organic azides are explosive. They can decompose to release $N_2(g)$ upon the input of energy, such as heat, pressure, or impact. All reactions with azides were done behind a safety screen of polymetacrylate of 2.5 cm thickness.

Amine *trans*-8b

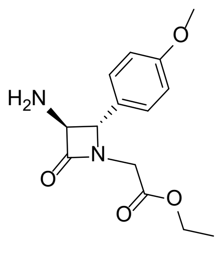

To a solution of the ***trans*-9b** (150 mg, 0.367 mmol, 1.0 eq) in a mixture of anhydrous CH_2Cl_2 (3.0 mL) and EtOH (8.0 mL), under argon atmosphere, was added dropwise ethylenediamine (44 mg, 0.735 mmol, 2.0 eq). The resulting mixture was stirred at rt for 4 h until disappearance of the starting material (TLC analysis). The volatiles were removed under *vacuum* to obtain ***trans*-8b** as a yellowish oil (63 mg, 62 %).

^{1}H NMR (400 MHz, $CDCl_3$) δ 7.21 (d, *J* = 8.3 Hz, 2H, *p*-OMe-C_6H_4), 6.90 (d, *J* = 8.3 Hz, 2H, *p*-OMe-C_6H_4), 4.47 (d, *J* = 2.1 Hz, 1H, β-lactam), 4.30 (d, *J* = 18.0 Hz, 1H, O=C-CH_2), 4.15 (dddd, *J* = 17.8, 10.7, 7.1, 3.5 Hz, 2H, CH_2CH_3), 3.96 (d, *J* = 2.1 Hz, 1H, β-lactam), 3.80 (s, 3H, CH_3, *p*-OMe-C_6H_4), 3.42 (d, *J* = 18.0 Hz, 1H, O=C-CH_2), 1.24 (t, *J* = 7.2 Hz, 3H, CH_2CH_3). ^{13}C NMR (101 MHz, $CDCl_3$) δ 171.5 (C), 168.2 (C), 160.1 (C), 128.27 (C), 127.9 (2CH, *p*-OMe-C_6H_4), 114.5 (2CH, *p*-OMe-C_6H_4), 70.7 (CH, β-lactam), 66.6 (CH, β-lactam), 61.7 (CH_2), 55.5 (CH_3), 41.3 (CH_2), 14.2 (CH_3). IR: ν_{max} 1736, 1513, 1245, 1200, 1175, 1028, 1175, 1028, 833 cm^{-1}. HRMS (ESI) m/z calculated for $C_{14}H_{19}N_2O_4$: 279.1339 [M + H]$^+$; found 279.1357.

Preparation of azides

Azide *trans*-7a

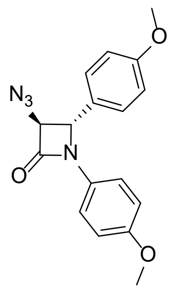

To a solution of amine mixture ***cis:trans*-8a** (1.28 g, 4.29 mmol, 1.0 eq) in anhydrous CH_2Cl_2 (128 mL), under argon atmosphere, was added dropwise Et_3N (0.59 mL, 4.29 mmol, 1.0 eq) and NfN_3 (4.18 g, 12.9mmol, 3.0 eq) successively. The resulting mixture was stirred for 18 h at room temperature (TLC analysis) until disappearance of the starting material. The volatiles were removed under *vacuum* and the residue was submitted to SiO_2 chromatography [(hexanes/EtOAc) (7:3)] to yield pure ***trans*-7a** as a white solid (1.20 g, 86 %).[2]

^{1}H NMR (400 MHz, $CDCl_3$) δ 7.26 (d, *J* = 8.8 Hz, 2H, *p*-OMe-C_6H_4), 7.20 (d, *J* = 9.1 Hz, 2H, *p*-OMe-C_6H_4), 6.92 (d, *J* = 8.8 Hz, 2H, *p*-OMe-C_6H_4), 6.78 (d, *J* = 9.1 Hz, 2H, *p*-OMe-C_6H_4), 4.79 (d, *J* = 2.0 Hz, 1H, β-lactam), 4.48 (d, *J* = 2.0 Hz, 1H, β-lactam), 3.81 (s, 3H, CH_3, *p*-OMe-C_6H_4), 3.74 (s, 3H, CH_3, *p*-OMe-C_6H_4).IR: ν_{max} 2119, 1514, 1370, 1355, 1232, 1182, 1139, 1041, 834, 801, 738, 592, 490 cm^{-1}.

Azide *cis*-7c

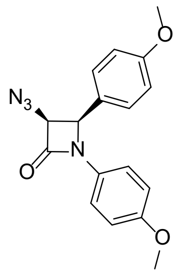

To a solution of the mixture of amines ***cis:trans*-8c** (215 mg, 0.72 mmol, 1.0 eq) in anhydrous CH_2Cl_2 (20 mL), under argon atmosphere, was successively added dropwise Et_3N (100 µL, 0.72 mmol, 1.0 eq) and NfN_3 (703 mg, 2.16 mmol, 3.0 eq). The resulting mixture was stirred for 18 h at room temperature (TLC analysis) until disappearance of the starting material. The volatiles were removed under *vacuum* and the residue was submitted to SiO_2 chromatography [(Hexanes/EtOAc)(7:3)] to yield pure ***cis*-7c** as a white solid (200 mg, 61 %).[8]

^1H NMR (300 MHz, $CDCl_3$) δ 7.28 (m, 4H, *p*-OMe-C_6H_4), 6.96 (d, *J* = 8.7 Hz, 2H, *p*-OMe-C_6H_4), 6.82 (d, *J* = 9.0 Hz, 2H, *p*-OMe-C_6H_4), 5.26 (d, *J* = 5.2 Hz, 1H, β-lactam), 5.01 (d, *J* = 5.2 Hz, 1H, β-lactam), 3.84 (s, 3H, CH_3, *p*-OMe-C_6H_4), 3.77 (s, 3H, CH_3, *p*-OMe-C_6H_4).

Azide *trans*-7b

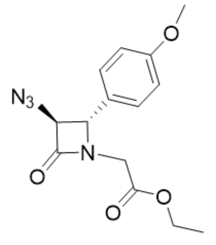

To a solution of amine ***trans*-8b** (0.475 g, 1.71 mmol, 1.0 eq) in anhydrous CH_2Cl_2 (48 mL), under argon atmosphere, was successively added dropwise Et_3N (0.24 mL, 1.71 mmol, 1.0 eq) and NfN_3 (1.66 g, 5.12 mmol, 3.0 eq). The resulting mixture was stirred for 5 h at room temperature (TLC analysis) until disappearance of the starting material. The volatiles were removed under *vacuum* and the residue was submitted to SiO_2 chromatography [(Hexanes/EtOAc)(8:2)] to yield pure ***trans*-7b** as an oil (0.36 g, 69 %).

^1H NMR (400 MHz, $CDCl_3$) δ 7.23 (d, *J* = 8.4 Hz, 2H, *p*-OMe-C_6H_4), 6.94 (d, *J* = 8.4 Hz, 2H, *p*-OMe-C_6H_4), 4.72 (d, *J* = 2.1 Hz, 1H, β-lactam), 4.37 (d, *J* = 2.1 Hz, 1H, β-lactam), 4.31 (d, *J* = 18.0 Hz, 1H, O=C-CH_2), 4.19 (m, 2H, CH_2CH_3), 3.82 (s, 3H, CH_3, *p*-OMe-C_6H_4), 3.46 (d, *J* = 18.0 Hz, 1H, O=C-CH_2), 1.26 (t, *J* = 7.6 Hz, 3H, CH_2CH_3). ^{13}C NMR (101 MHz, $CDCl_3$) δ 167.6 (C), 165.1 (C), 160.6 (C), 128.2 (2CH, *p*-OMe-C_6H_4), 126.3 (C), 114.8 (2CH, *p*-OMe-C_6H_4), 73.0 (CH, β-lactam), 63.1 (CH, β-lactam), 62.0 (CH_2), 55.5 (CH_3, *p*-OMe-C_6H_4), 41.6 (CH_2), 14.2 (CH_3, CH_2CH_3). IR: ν_{max} 2109, 1761, 1741, 1248, 1236, 1201, 1194 cm^{-1}. HRMS (ESI) m/z calculated for $C_{14}H_{16}N_4O_4Na$: 327.1064 $[M + Na]^+$; found 327.1063.

General procedure for the synthesis of triazoles, 10.

A mixture of organic azide (1.0 eq), alkyne (1.2 eq), sodium (*l*)-ascorbate (0.5 eq) and $CuSO_4 \cdot 5H_2O$ (0.25 eq) in DMF was stirred under argon at rt until completion of the reaction (TLC analysis). The solvent was evaporated under *vacuum* and the product was purified by column chromatography (SiO_2) to afford the pure triazoles ***trans*-10a**, ***trans*-10b** and ***cis*-10c**.

Triazole *trans*-10a

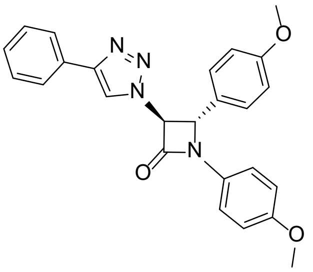

Following the general procedure, a mixture of azide ***trans*-7a** (576 mg, 1.78 mmol, 1.0 eq), phenylacetylene (250 mg, 2.13 mmol, 1.2 eq), sodium (L)-ascorbate (176 mg, 0.89 mmol, 0.5 eq) and $CuSO_4 \cdot 5H_2O$ (133 mg, 0.53 mmol, 0.3 eq) in DMF (20 mL) was stirred under argon at rt for 3 h. The solvents were removed under *vacuum* and the crude was purified to SiO_2 chromatography [(Hex/EtOAc) (7:3)] to yield ***trans*-10a** as a white solid (192 mg, 52%).

^1H NMR (400 MHz, $CDCl_3$) δ 8.00 (s, 1H, N_3C=CH), 7.83 (d, *J* = 7.3 Hz, 2H, Ph), 7.43 (t, *J* = 7.3 Hz, 2H, Ph), 7.35 (d, *J* = 8.7 Hz, 2H, *p*-OMe-C_6H_4), 7.34 (1H, overlapped, Ph), 7.29 (d, *J* = 9.0 Hz, 2H, *p*-OMe-C_6H_4), 6.94 (d, *J* = 8.7 Hz, 2H, *p*-OMe-C_6H_4), 6.82 (d, *J* = 9.0 Hz, 2H, *p*-OMe-C_6H_4), 5.59 (d, *J* = 2.2 Hz, 1H, β-lactam), 5.41 (d, *J* = 2.2 Hz, 1H, β-lactam), 3.82 (s, 3H, CH_3, *p*-OMe-C_6H_4), 3.76 (s, 3H, CH_3, *p*-OMe-C_6H_4). ^{13}C NMR (101 MHz, $CDCl_3$) δ 160.7 (C, *p*-OMe-C_6H_4), 158.9 (C, C=O β-lactam), 157.1 (C, *p*-OMe-C_6H_4), 148.4 (C, N_3C=CH), 130.1 (C, *p*-OMe-C_6H_4), 129.9 (C, *p*-OMe-C_6H_4), 129.0 (2CH, Ph), 128.6 (CH, Ph), 127.8 (2CH, *p*-OMe-C_6H_4), 126.5 (C, Ph), 126.0 (2CH, Ph), 119.5 (CH, N_3C=CH), 119.4 (2CH, *p*-OMe-C_6H_4), 115.1 (2CH, *p*-OMe-C_6H_4), 114.6 (2CH, *p*-OMe-C_6H_4), 72.3 (CH, β-lactam), 63.35 (CH, β-lactam), 55.58 (CH_3, *p*-OMe-C_6H_4), 55.52 (CH_3, *p*-OMe-C_6H_4). IR: ν_{max} 1752, 1510, 1246, 1027 cm^{-1}. HRMS (ESI) *m/z* calculated for $C_{25}H_{23}N_4O_3$: 427.1765 $[M + H]^+$; found 427.1754. Mp: 65 °C.

Triazole *cis*-10c

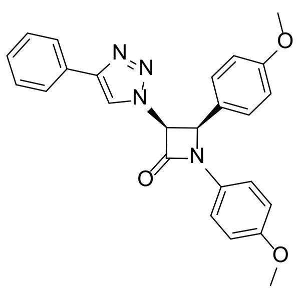

Following the general procedure, a mixture of azide ***cis*-7c** (200 mg, 0.62 mmol, 1.0 eq), phenylacetylene (76 mg, 0.74 mmol, 1.2 eq), sodium (L)-ascorbate (61 mg, 0.31 mmol, 0.5 eq) and $CuSO_4{\cdot}5H_2O$ (46 mg, 0.18 mmol, 0.3 eq) in DMF (10 mL) was stirred under argon at rt for 3 h. The solvents were removed under *vacuum* and the crude was purified to SiO_2 chromatography [(Hexanes/EtOAc (7:3)] to yield ***cis*-10c** as a white solid (201 mg, 76%). ^{1}H NMR (400 MHz,$CDCl_3$) δ 7.66-7.60 (m, 3H, 2H Ph + 1H N_3C=CH), 7.41-7.32 (m, 4H, 2H *p*-OMe-C_6H_4 + 2H Ph), 7.32-7.28 (m, 1H, Ph)., 7.10 (d, *J* = 8.6 Hz, 2H, *p*-OMe-C_6H_4), 6.87 (d, *J* = 8.1 Hz, 2H, *p*-OMe-C_6H_4), 6.70 (d, *J* = 8.6 Hz, 2H, *p*-OMe-C_6H_4), 6.38 (d, *J* = 5.4 Hz, 1H, β-lactam), 5.62 (d, *J* = 5.4 Hz, 1H, β-lactam), 3.79 (s, 3H, CH_3, *p*-OMe-C_6H_4), 3.65 (s, 3H,CH_3, *p*-OMe-C_6H_4). ^{13}C NMR (101 MHz,$CDCl_3$) δ 160.2 (C, *p*-OMe-C_6H_4), 158.8 (C, C=O β-lactam), 157.1 (C, *p*-OMe-C_6H_4), 147.7 (C, N_3C=CH), 130.2 (2C, *p*-OMe-C_6H_4 + Ph), 128.9 (2CH, Ph), 128.4 (1CH,Ph), 128.1 (2CH, *p*-OMe-C_6H_4), 125.9 (2CH, Ph), 123.0 (C, *p*-OMe-C_6H_4), 119.9 (CH, N_3C=CH), 119.1 (2CH, *p*-OMe-C_6H_4), 114.7 (2CH, *p*-OMe-C_6H_4), 114.4 (2CH, *p*-OMe-C_6H_4), 67.9 (CH, β-lactam), 61.1 (CH, β-lactam), 55.6 (CH_3, *p*-OMe-C_6H_4), 55.3 (CH_3, *p*-OMe-C_6H_4). IR: ν_{max} 1756, 1511, 1248, 768 cm^{-1}. HRMS (ESI) *m/z* calculated for $C_{25}H_{23}N_4O_3$: 427.1765 [M + H]$^+$; found 427.1754. Mp: 230 °C.

Triazole *trans*-10b

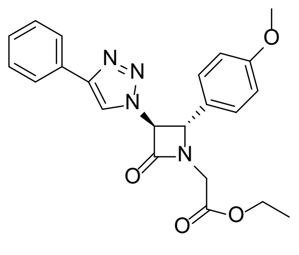

Following the general procedure, a mixture of azide ***trans*-7b** (218 mg, 0.72 mmol, 1.0 eq), phenylacetylene (87.8 mg, 0.86 mmol, 1.2 eq), sodium (L)-ascorbate (70.9 mg, 0.36 mmol, 0.5 eq) and $CuSO_4{\cdot}5H_2O$ (53.7 mg, 0.22 mmol, 0.3 eq) in DMF (10 mL) was stirred under argon at rt for 1 h. The solvents were removed under *vacuum* and the crude was purified to SiO_2 chromatography [(Hexanes/EtOAc (7:3)] to yield ***trans*-10b** as a white solid (179 mg, 61 %).

^{1}H NMR (400 MHz, $CDCl_3$) δ 8.19 (s, 1H, N_3C=CH), 7.91-7.83 (m, 2H, Ph), 7.44 (t, *J* = 7.5 Hz, 2H, Ph), 7.39-7.33 (m, 1H, Ph), 7.30 (d, *J* = 8.7 Hz, 2H, *p*-OMe-C_6H_4), 6.96 (d, *J* = 8.7 Hz, 2H, *p*-OMe-C_6H_4), 5.73 (d, *J* = 2.2 Hz, 1H, β-lactam), 5.06 (d, *J* = 2.2 Hz, 1H, β-lactam), 4.48 (d, *J* = 18.1 Hz, 1H, O=C-CH_2), 4.24 (m, 2H, CH_2CH_3), 3.83 (s, 3H, CH_3, *p*-OMe-C_6H_4),

3.58 (d, *J* = 18.1 Hz, 1H, O=C-CH_2), 1.29 (t, *J* = 7.1 Hz, 3H, CH_2CH_3). ^{13}C NMR (101 MHz, $CDCl_3$) δ 167.7 (C), 163.5 (C), 160.9 (C), 148.7 (C), 130.3 (C), 129.0 (2CH), 128.5 (CH), 128.2 (2CH), 126.0 (2CH), 125.8 (C), 118.8 (CH), 115.0 (2CH), 73.0 (CH, β-lactam), 64.5 (CH, β-lactam), 62.3 (CH_2), 55.6 (CH_3, *p*-OMe-C_6H_4), 42.0 (CH_2), 14.2 (CH_3, CH_2CH_3). IR: ν_{max} 1788, 1719, 1256, 1224, 1033, 1019 cm^{-1}. HRMS (ESI) m/z calculated for $C_{22}H_{23}N_4O_4$: 407.1714 $[M + H]^+$; found 407.1718. Mp: 115 °C.

General procedure for the synthesis of triazolium salts.

Method A. A mixture of triazole (1.0 equiv) and Meerwein's salt (1.5 equiv) in CH_2Cl_2 was stirred under argon at rt until reaction completion (TLC analysis). The reaction was quenched with some drops of methanol. The solvent was removed under *vacuum* and the resulting residue was dissolved in the minimum amount of CH_2Cl_2 and precipitated with Et_2O. The solvents were decanted, and the solid was washed with Et_2O (x3) and *vacuum*-dried to yield pure triazolium salts.

Method B. MeOTf (1.5 equiv) was added to a CH_2Cl_2 solution of triazole (1.0 equiv) at 0 ˚C. The mixture was stirred at 10 ˚C (cold room laboratory) for 4h, until no evolution of the reaction was observed. The solvent was removed under *vacuum* and the resulting residue was dissolved in the minimum amount of CH_2Cl_2 and precipitated with Et_2O. The solvents were decanted and the solid was washed with Et_2O (x3) and *vacuum*-dried to yield the pure triazolium salts.

Triazolium salt *trans*-11a

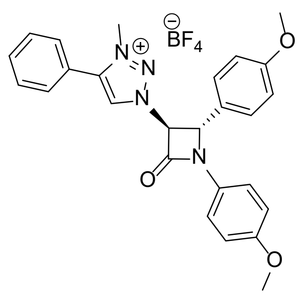

Following method A, treatment of ***trans*-10a** (350 mg, 0.82 mmol, 1.0 eq) with Me_3OBF_4 (182 mg, 1.23 mmol, 1.5 eq) in CH_2Cl_2 (40 mL) afforded pure ***trans*-11a** as a white solid (332 mg, 77%).

^1H NMR (400 MHz, $CDCl_3$) δ 8.29 (s, 1H, N_3C=CH), 7.66-7.50 (m, 5H, Ph), 7.46 (d, *J* = 8.6 Hz, 2H, *p*-OMe-C_6H_4), 7.27 (d, *J* = 8.9 Hz, 2H, *p*-OMe-C_6H_4), 6.89 (d, *J* = 8.6 Hz, 2H, *p*-OMe-C_6H_4), 6.80 (d, *J* = 8.9 Hz, 2H, *p*-OMe-C_6H_4), 5.80 (d, *J* = 2.0 Hz, 1H, β-lactam), 5.71 (d, *J* = 2.0 Hz, 1H, β-lactam), 4.22 (s, 3H, CH_3, N-CH_3), 3.76 (s, 3H, CH_3, *p*-OMe-C_6H_4), 3.74 (s, 3H, CH_3, *p*-OMe-C_6H_4). ^{13}C NMR (101

MHz, $CDCl_3$) δ 160.8 (C, *p*-OMe-C_6H_4), 157.5 (C, *p*-OMe-C_6H_4), 156.2 (C, C=O, β-lactam), 144.4 (C, N_3C=CH), 132.2 (CH, Ph), 129.8 (4CH, Ph), 129.0 (C, *p*-OMe-C_6H_4), 128.5 (2CH, *p*-OMe-C_6H_4), 128.1 (CH, N_3C=CH), 125.3 (C, *p*-OMe-C_6H_4), 121.6 (C, Ph), 120.0 (2CH, *p*-OMe-C_6H_4), 115.0 (2CH, *p*-OMe-C_6H_4), 114.7 (2CH, *p*-OMe-C_6H_4), 73.7 (CH, β-lactam), 63.0 (CH, β-lactam), 55.6 (CH_3, *p*-OMe-C_6H_4), 55.5 (CH_3, *p*-OMe-C_6H_4), 38.9 (CH_3, N-CH_3). IR: ν_{max} 1763, 1512, 1248, 1027 cm^{-1}. HRMS (ESI) *m/z* calculated for $C_{26}H_{25}N_4O_3$: 441.1921 $[M]^+$; found 441.1918. Mp: 97 °C.

Triazolium salt *cis*-11c

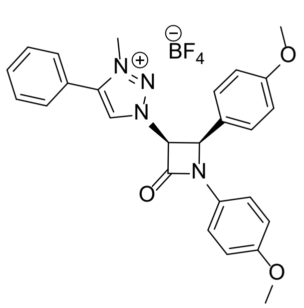

Following method A, treatment of ***cis*-10c** (86 mg, 0.20 mmol, 1.0 eq) with Me_3OBF_4 (45 mg, 0.30 mmol, 1.5 eq) in CH_2Cl_2 (9 mL) afforded pure ***cis*-11c** as a white solid (90 mg, 85 %).

^1H NMR (400 MHz, $CDCl_3$) δ 8.85 (s, 1H, N_3C=CH), 7.65-7.59 (m, 1H, Ph), 7.58-7.52 (m, 2H, Ph), 7.37-7.31 (m, 3H, 1H Ph + 2H *p*-OMe-C_6H_4), 7.32-7.25 (m, 3H, 1H Ph + 2H *p*-OMe-C_6H_4), 6.86 (d, *J* = 9.0 Hz, 2H, *p*-OMe-C_6H_4), 6.83 (d, *J* = 5.4 Hz, 1H, β-lactam), 6.77 (d, *J* = 9.0 Hz, 2H, *p*-OMe-C_6H_4), 5.98 (d, *J* = 5.4 Hz, 1H, β-lactam), 4.06 (s, 3H, CH_3, N-CH_3), 3.79 (s, 3H, CH_3, *p*-OMe-C_6H_4), 3.72 (s, 3H, CH_3, *p*-OMe-C_6H_4). ^{13}C NMR (101 MHz, $CDCl_3$) δ 160.6 (C, *p*-OMe-C_6H_4), 157.4 (C, *p*-OMe-C_6H_4), 155.5 (C, C=O, β-lactam), 143.5 (C, N_3C=CH), 132.5 (CH), 130.1 (2CH), 129.9 (CH, N_3C=CH), 129.6 (C, *p*-OMe-C_6H_4), 129.1 (4CH), 122.3 (C, *p*-OMe-C_6H_4), 121.1 (C, Ph), 119.4 (2CH), 114.7 (2CH, *p*-OMe-C_6H_4), 114.1 (2CH, *p*-OMe-C_6H_4), 70.3 (CH, β-lactam), 60.6 (CH, β-lactam), 55.6 (CH_3, *p*-OMe-C_6H_4), 55.5 (CH_3, *p*-OMe-C_6H_4), 38.6 (CH_3, N-CH_3). IR: ν_{max} 1781, 1514, 1252, 1065, 1018, 841 cm^{-1}. HRMS (ESI) *m/z* calculated for $C_{26}H_{25}N_4O_3$: 441.1921 $[M]^+$; found 441.1915. Mp: 75 °C

Triazolium salt *trans*-11b

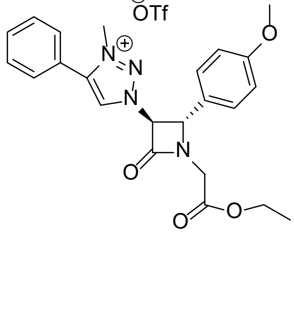

Following method B, treatment of ***trans*-10b** (177 mg, 0.44 mmol, 1.0 eq) with MeOTf (73.8 µL, 0.65 mmol, 1.5 eq) in CH_2Cl_2 (20 mL) afforded pure ***trans*-11b** as a white solid (243 mg, 98 %).

^{1}H NMR (400 MHz, $CDCl_3$) δ 8.69 (s, 1H, N_3C=CH), 7.71-7.64 (m, 2H, Ph), 7.63-7.49 (m, 3H, Ph), 7.48 (d, *J* = 8.8 Hz, 2H, *p*-OMe-C_6H_4), 6.94 (d, *J* = 8.8 Hz, 2H, *p*-OMe-C_6H_4), 5.78 (d, *J* = 2.0 Hz, 1H, β-lactam), 5.48 (d, *J* = 2.0 Hz, 1H, β-lactam), 4.32 (d, *J* = 18.2 Hz, 1H, O=C-CH_2), 4.27 (s, 3H, N-CH_3), 4.21 (q, *J* = 7.2 Hz, 2H, CH_2CH_3), 3.80 (s, 3H, *p*-OMe-C_6H_4), 3.70 (d, *J* = 18.2 Hz, 1H, O=C-CH_2), 1.26 (t, *J* = 7.2 Hz, 3H, CH_2CH_3). ^{13}C NMR (101 MHz, $CDCl_3$) δ 167.2 (C), 161.0 (C), 160.7 (C), 144.6 (C), 132.2 (CH), 129.9 (2CH), 129.8 (2CH), 128.9 (2CH), 127.6 (CH), 124.6 (C), 121.7 (C), 114.9 (2CH), 74.4 (CH, β-lactam), 63.4 (CH, β-lactam), 62.5 (CH_2), 55.5 (CH_3), 42.8 (CH_2), 39.0 (CH_3), 14.1 (CH_3). IR: ν_{max} 1780, 1746, 1252, 1200, 1156, 1029, 637 cm^{-1}. HRMS (ESI) m/z calculated for $C_{23}H_{25}N_4O_4$: 421.1870 $[M]^+$; found 421.1868. Mp: 145 °C.

General procedure for the synthesis of Au complexes

In a Schlenk flask charged with 4 Å molecular sieves, a mixture of the corresponding triazolium salt (1.0 eq), NMe_4Cl (1.5 eq) and Ag_2O (0.8 eq) in a 1:10 CH_3CN/CH_2Cl_2 mixture was stirred at rt in the dark until formation of the corresponding silver carbene (^{1}H NMR analysis). [AuCl(SMe_2)] complex (1.1 eq) was then added and the mixture was stirred at rt until the reaction was completed (^{1}H NMR analysis). The mixture was filtered through a short pad of celite and the volatiles were removed under *vacuum* to afford the corresponding carbene complexes, which were purified through a short pad of SiO_2.

Au(I) complexes ,12a

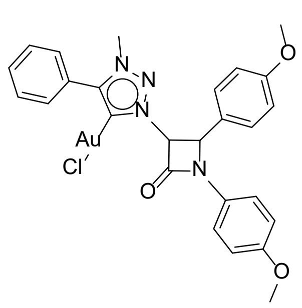

A mixture of the triazolium salt ***trans*-11a** (60 mg, 0.114 mmol, 1.0 eq), NMe_4Cl (18.7 mg, 0.170 mmol, 1.5 eq), and Ag_2O (21.1 mg, 0.091 mmol, 0.8 eq) in 10:1 CH_2Cl_2:CH_3CN (16.5 mL) was stirred, under argon, at room temperature overnight. Then, [AuCl(SMe_2)] (66.9 mg, 0.277 mmol, 1.1 eq) was added and the mixture stirred at room temperature for 2 h. The crude was purified (SiO_2, Hexane/AcOEt 3:2) yielding ***cis*-12a** (16 mg, 21%) and ***trans*-12a** (30 mg, 39%) as white solids. The metallation of the triazolium salt ***cis*-11c** under the above conditions leads to the same mixture of reaction products and in the same proportion.

Au(I) complex *cis*-12a

^{1}H NMR (400 MHz, $CDCl_3$) δ 7.51-7.42 (m, 3H, Ph), 7.37 (d, *J* = 9.0 Hz, 2H, *p*-OMe-C_6H_4), 7.33-7.28 (m, 2H, Ph), 7.26 (d, *J* = 8.7 Hz, 2H, *p*-OMe-C_6H_4), 6.86 (d, *J* = 9.0 Hz, 2H, *p*-OMe-C_6H_4), 6.73 (d, *J* = 8.7 Hz, 2H, *p*-OMe-C_6H_4), 6.59 (d, *J* = 5.4 Hz, 1H, β-lactam), 5.73 (d, *J* = 5.4 Hz, 1H, β-lactam), 3.88 (s, 3H, CH_3), 3.78 (s, 3H, CH_3), 3.72 (s, 3H, CH_3). ^{13}C NMR (101 MHz, $CDCl_3$) δ 160.4 (C), 159.1 (C), 157.2 (C), 156.4 (C), 147.3 (C), 130.7 (CH), 130.0 (C), 129.3 (5CH), 129.3 (CH), 125.6 (C), 122.6 (C), 119.2 (2CH), 114.7 (2CH), 113.6 (2CH), 71.6 (CH, β-lactam), 60.5 (CH, β-lactam), 55.6 (CH_3), 55.5 (CH_3), 37.9 (CH_3). IR: ν_{max} 1755, 1511, 1250, 1176, 1025, 823, 698, 552 cm^{-1}. HRMS (ESI) m/z calculated for $C_{28}H_{27}AuN_5O_3$: 678.1774 [M – Cl + MeCN]$^+$; found 678.1778. Mp: 230 °C.

Au(I) complex *trans*-12a

^{1}H NMR (400 MHz, $CDCl_3$) δ 7.65-7.59 (m, 2H, Ph), 7.56-7.47 (m, 3H, Ph), 7.37 (d, *J* = 8.7 Hz, 2H, *p*-OMe-C_6H_4), 7.31 (d, *J* = 9.2 Hz, 2H, *p*-OMe-C_6H_4), 6.94 (d, *J* = 8.7 Hz, 2H, *p*-OMe-C_6H_4), 6.83 (d, *J* = 9.2 Hz, 2H, *p*-OMe-C_6H_4), 5.91 (d, *J* = 2.2 Hz, 1H, β-lactam), 5.55 (d, *J* = 2.2 Hz, 1H, β-lactam), 4.11 (s, 3H, CH_3), 3.82 (s, 3H, CH_3), 3.75 (s, 3H, CH_3). ^{13}C NMR (101 MHz, $CDCl_3$) δ 160.8 (C), 159.1 (C), 157.7 (C), 157.1 (C), 148.0 (C), 130.8 (CH), 129.7 (C), 129.7 (2CH), 129.4 (2CH), 128.1 (2CH), 125.8 (C), 119.8 (2CH), 115.2 (2CH), 114.6 (2CH), 76.0 (CH, β-lactam), 63.7 (CH, β-lactam), 55.6 (CH_3), 55.5 (CH_3), 38.3 (CH_3). IR: ν_{max} 1759, 1511, 1247, 1175, 1145, 1074, 1026, 829 cm^{-1}. HRMS (ESI) m/z calculated for $C_{28}H_{27}AuN_5O_3$: 678.1774 [M - Cl + MeCN]$^+$; found 678.1778. Mp: 148 °C

Au(I) complexes, 12b

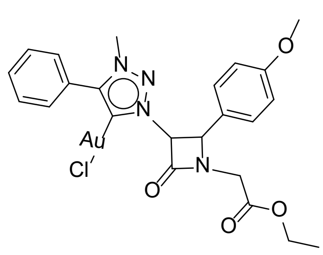

A mixture of the triazolium salt ***trans*-11b** (100 mg, 0.18 mmol, 1.0 eq), NMe_4Cl (29 mg, 0.26 mmol, 1.5 eq), and Ag_2O (33 mg, 0.14 mmol, 0.8 eq) in 10:1 CH_2Cl_2:CH_3CN (16.5 mL) was stirred, under argon, at room temperature overnight. Then, [AuCl(SMe_2)] (62 mg, 0.21 mmol, 1.2 eq) was added and the mixture was stirred at room temperature for 2 h. A *cis:trans* (0.3:1) mixture of Au-complexes **12b** was obtained. From this mixture pure ***trans*-12b** (46%) isomer was precipitated using AcOEt. The ***cis*-12b** isomer could not be isolated in pure form.

Au(I) complex *trans*-12b

^{1}H NMR (400 MHz, $CDCl_3$) δ 7.65-7.58 (m, 2H, Ph), 7.57-7.47 (m, 3H, Ph), 7.37 (d, *J* = 8.7 Hz, 2H, *p*-OMe-C_6H_4), 6.97 (d, *J* = 8.7 Hz, 2H, *p*-OMe-C_6H_4), 5.93 (d, *J* = 2.3 Hz, 1H, β-lactam), 5.45 (d, *J* = 2.3 Hz, 1H, β-lactam), 4.30 (d, *J* = 18.0 Hz, 1H), 4.19 (qd, *J* = 7.1, 1.8 Hz, 2H), 4.14 (s, 3H), 3.86 (d, *J* = 18.0 Hz, 1H), 3.84 (s, 3H) 1.26 (t, *J* = 7.1 Hz, 3H). ^{13}C NMR (101 MHz, $CDCl_3$) δ 167.5 (C), 161.4 (C), 161.1 (C), 158.7 (C), 148.0 (C), 130.8 (CH), 129.6 (2CH), 129.4 (2CH), 128.8 (2CH), 125.8 (C), 125.1 (C), 115.1 (2CH), 76.0 (CH), 64.4 (CH), 62.0 (CH_2), 55.6 (CH_3), 42.2 (CH_2), 38.3 (CH_3), 14.2 (CH_3). IR: ν_{max} 1766, 1722, 1266, 1242, 1176, 1031, 816, 699, 564 cm^{-1}. HRMS (ESI) m/z calculated for $C_{23}H_{24}AuN_4O_4$: 617.1458 $[M]^+$; found 617.1452 Mp: 205 °C.

General procedure for the synthesis of Ir complexes, 13 and **14.**

In a Schlenk flask charged with 4 Å molecular sieves, a mixture of triazolium salt (1.0 eq), NMe_4Cl (1.5 eq) and Ag_2O (0.8 eq) was stirred at rt in the dark in a 1:10 CH_3CN/CH_2Cl_2 mixture until formation of the corresponding silver carbene (^{1}H NMR analysis). $[IrCl_2Cp^*]_2$ complex (0.5 eq) was then added, and the mixture was stirred at rt until the reaction was completed (^{1}H NMR analysis). The mixture was filtered through a short pad of Celite and the volatiles were removed under *vacuum* to afford the corresponding carbene complexes, which were purified through a short pad of SiO_2.

Ir(III) complexes, 13

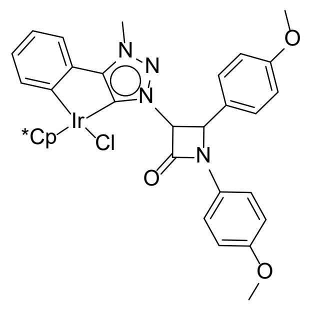

A mixture of the triazolium salt ***trans*-11a** (80 mg, 0.15 mmol, 1.0 eq), NMe_4Cl (25 mg, 0.23 mmol, 1.5 eq) and Ag_2O (28 mg, 0.12 mmol, 0.8 eq) in 10:1 CH_2Cl_2:CH_3CN was stirred, under argon, at room temperature overnight. Then, $[IrCl_2Cp^*]_2$ (60 mg, 0.075 mmol, 0.5 eq) was added and the mixture stirred at room temperature for 3 h. The crude is purified (SiO2, Hexane/AcOEt (3:7)) to yield ***cis*-13** (26 mg, 21%) and ***trans*-13** (30.3 mg 25%) as orange solids.

Ir(III) complex *cis*-13

^{1}H NMR (400 MHz, $CDCl_3$) δ 7.59 (dd, *J* = 1.7, 7.3 Hz, 2H, Ph), 7.48 (d, *J* = 8.7 Hz, 2H, *p*-OMe-C_6H_4), 7.45-7.35 (m, 4H, 2H *p*-OMe-C_6H_4 + 2H Ph), 7.21(d, *J* = 5.7 Hz, 1H, β-lactam), 6.87 (d, *J* = 9.2 Hz, 2H, *p*-OMe-C_6H_4), 6.74 (d, *J* = 9.0 Hz, 2H, *p*-OMe-C_6H_4), 5.97 (d, *J* = 5.7 Hz, 1H, β-lactam), 3.78 (s, 3H, CH_3, *p*-OMe-C_6H_4), 3.71 (s, 3H, CH_3, *p*-OMe-C_6H_4), 3.48 (s, 3H, CH_3, N-CH_3), 1.40 (s, 15H, 5CH_3, Cp*). ^{13}C NMR (101 MHz, $CDCl_3$) δ 161.0 (C, C=O, β-lactam), 159.6 (C, *p*-OMe-C_6H_4), 156.7 (C, *p*-OMe-C_6H_4), 148.6 (C, N_3C=CIr), 148.4 (C, N_3C=CIr), 132.5 (2CH, Ph), 130.7 (C, *p*-OMe-C_6H_4), 130.1 (CH, Ph), 130.0 (CH, Ph), 128.0 (2CH, *p*-OMe-C_6H_4), 126.9 (C, Ph), 123.8 (C, *p*-OMe-C_6H_4), 119.0 (2CH, *p*-OMe-C_6H_4), 114.6 (2CH, *p*-OMe-C_6H_4), 113.4 (2CH, *p*-OMe-C_6H_4), 88.7 (5C, Cp*), 71.2 (CH, β-lactam), 63.6 (CH, β-lactam), 55.6 (CH_3, *p*-OMe-C_6H_4), 55.3 (CH_3, *p*-OMe-C_6H_4), 37.3 (CH_3, N-CH_3), 8.81 (5CH_3, Cp*). IR: ν_{max} 1754, 1511, 1247, 1176, 1025, 828, 717, 702 cm^{-1}. HRMS (ESI) m/z calculated for $C_{36}H_{39}ClIrN_4O_3$: 803.2328 $[M + H]^+$; found 803.2327. Mp: 230 °C.

Ir(III) complex *trans*-13

^{1}H NMR (400 MHz, $CDCl_3$) δ 7.64 (d, *J* = 5.8 Hz, 2H, Ph), 7.49-7.40 (m, 2H, Ph), 7.35-7.29 (m, 4H, *p*-OMe-C_6H_4), 6.93 (d, *J* = 2.0 Hz, 1H, β-lactam), 6.87 (d, *J* = 8.7 Hz, 2H, *p*-OMe-C_6H_4), 6.82 (d, *J* = 9.0 Hz, 2H, *p*-OMe-C_6H_4), 5.51 (d, *J* = 2.0 Hz, 1H, β-lactam), 3.79 (s, 3H, CH_3, *p*-OMe-C_6H_4), 3.75 (s, 6H, 2CH_3, *p*-OMe-C_6H_4 + N-CH_3), 1.44 (s, 15H, 5CH_3, Cp*).^{13}C NMR (101 MHz, $CDCl_3$) δ 161.7 (C, C=O, β-lactam), 160.2 (C, *p*-OMe-C_6H_4), 156.7 (C, *p*-OMe-C_6H_4), 149.8 (C, N_3C=CIr), 149.1 (C, N_3C=CIr), 132.6 (2CH, Ph), 130.4 (C, *p*-OMe-C_6H_4), 130.1 (CH, Ph), 128.7 (2CH, *p*-OMe-C_6H_4), 128.0 (CH, Ph), 127.4 (C, Ph), 126.5 (C, *p*-OMe-C_6H_4), 119.4 (2CH, *p*-OMe-C_6H_4), 114.5 (2CH, *p*-OMe-C_6H_4), 114.4 (2CH, *p*-OMe-C_6H_4), 88.5 (5C, Cp*), 74.6 (CH, β-lactam), 64.9 (CH, β-lactam), 55.6 (CH_3, *p*-OMe-C_6H_4), 55.3 (CH_3, *p*-OMe-C_6H_4), 37.5 (CH_3, N-CH_3), 8.78 (5CH_3, Cp*). IR: ν_{max} 1755, 1511, 1246, 1024, 829, 702 cm^{-1}. HRMS (ESI) m/z calculated for $C_{36}H_{39}ClIrN_4O_3$: 803.2328 $[M + H]^+$; found 803.2283. Mp: 194 °C.

Ir(III) complexes, 14

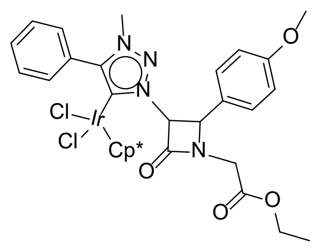

A mixture of the triazolium salt ***trans*-11b** (50 mg, 0.088 mmol, 1.0 eq), NMe_4Cl (14 mg, 0.18 mmol, 1.5 eq) and Ag_2O (16 mg, 0.070 mmol, 0.8 eq) in 10:1 CH_2Cl_2:CH_3CN was stirred, under argon, at room temperature overnight. Then, $[IrCl_2Cp^*]_2$ (35 mg, 0.044 mmol, 0.5 eq) was added and the mixture stirred at room temperature for 3 h. The crude was purified (SiO2, Hexane/AcOEt (3:7)) to yield ***cis*-14** (11 mg, 15%) and ***trans*-14** (20 mg, 28%) as orange solids.

Ir(III) complex *cis*-14

^{1}H NMR (500 MHz, $CDCl_3$) δ 7.56-7.50 (m, 4H, 2H Ph + 2H *p*-OMe-C_6H_4), 7.44-7.35 (m, 3H, Ph), 7.16 (d, *J* = 5.6 Hz, 1H, β-lactam), 6.81 (d, *J* = 8.7 Hz, 2H, *p*-OMe-C_6H_4), 5.70 (d, *J* = 5.6 Hz, 1H, β-lactam), 4.52 (d, *J* = 17.9 Hz, 1H, O-CH_2), 4.23 (qd, *J* = 7.1, 3.2 Hz, 2H, CH_2CH_3), 3.76 (d, *J* = 17.9 Hz, 1H, O-CH_2), 3.76 (s, 3H, CH_3, *p*-OMe-C_6H_4), 3.53 (s, 3H, N-CH_3), 1.45 (s, 15H, 5CH_3, Cp*), 1.30 (t, *J* = 7.1 Hz, 3H, CH_3, CH_2CH_3). ^{13}C NMR (126 MHz, $CDCl_3$) δ 167.3 (C, O=C-OCH_2), 165.6 (C, C=O, β-lactam), 159.8 (C, *p*-OMe-C_6H_4), 149.5 (C, N_3C=CIr), 148.4 (C, N_3C=CIr), 132.5 (2CH, Ph), 130.4 (2CH, *p*-OMe-C_6H_4), 130.0 (CH, Ph), 128.0 (2CH, Ph), 127.0 (C, Ph), 123.9 (C, *p*-OMe-C_6H_4), 113.7 (2CH, *p*-OMe-C_6H_4), 88.7 (5C, Cp*), 72.7 (CH, β-lactam), 64.8 (CH, β-lactam), 62.0 (CH_2, CH_2CH_3), 55.4 (CH_3, *p*-OMe-C_6H_4), 42.5 (CH_2, O=C-CH_2N), 37.3 (CH_3, N-CH_3), 14.3 (CH_3, CH_2CH_3), 8.87 (5CH_3, Cp*). IR: ν_{max} 2922, 1773, 1754, 1247, 1190, 1179, 1029, 1019, 700 cm^{-1}. HRMS (ESI) m/z calculated for $C_{33}H_{39}ClIrN_4O_4$: 783.2277 $[M - Cl]^+$; found 783.2281 Mp: 225 °C.

Ir(III) complex *trans*-14

^{1}H NMR (400 MHz, $CDCl_3$) δ 7.65 (d, *J* = 8.2 Hz, 2H, Ph), 7.49-7.37 (m, 3H, Ph), 7.28 (d, *J* = 8.7 Hz, 2H, *p*-OMe-C_6H_4), 7.08 (d, *J* = 2.2 Hz, 1H, β-lactam), 6.91 (d, *J* = 8.7 Hz, 2H, *p*-OMe-C_6H_4), 5.45 (d, *J* = 2.2 Hz, 1H, β-lactam), 4.34 (d, *J* = 17.9 Hz, 1H, O-CH_2), 4.20 (qd, *J* = 7.1, 5.4 Hz, 2H, CH_2CH_3), 3.80 (s, 6H, CH_3 *p*-OMe-C_6H_4 + CH_3 N-CH_3), 3.61 (d, *J* = 17.9 Hz, 1H, O-CH_2), 1.43 (s, 15H, 5CH_3, Cp*), 1.28 (t, *J* = 7.1 Hz, 3H, CH_2CH_3). ^{13}C NMR (101 MHz, $CDCl_3$) δ 167.7 (C, O=C-OCH_2), 165.0 (C, C=O, β-lactam), 160.4 (C, *p*-OMe-C_6H_4),

149.8 (C, N_3C=CIr), 148.6 (C, N_3C=CIr), 132.7 (2CH, Ph), 130.1 (CH, Ph), 129.4 (2CH, *p*-OMe-C_6H_4), 128.0 (2CH, Ph), 127.4 (C, Ph), 125.5 (C, *p*-OMe-C_6H_4), 114.4 (2CH, *p*-OMe-C_6H_4), 88.5 (5C, Cp*), 74.4 (CH, β-lactam), 65.2 (CH, β-lactam), 61.8 (CH_2, CH_2CH_3), 55.4 (CH_3, *p*-OMe-C_6H_4), 41.3 (CH_2, O=C-CH_2N), 37.6 (CH_3, N-CH_3), 14.3 (CH_3, CH_2CH_3), 8.76 (5CH_3, Cp*). IR: ν_{max} 2925, 1772, 1733, 1248, 1023, 702 cm^{-1}. HRMS (ESI) m/z calculated for $C_{33}H_{39}ClIrN_4O_4$: 783.2277 $[M-Cl]^+$; found 783.2278. Mp: 225 °C.

Pd(II) complex *trans*-15

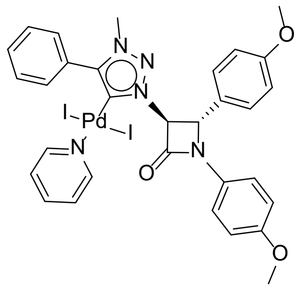

3.0 mL of pyridine were added to a mixture of triazolium salt ***trans*-11a** (60 mg, 0.114 mmol, 1.0 eq), K_2CO_3 (17.3 mg, 0.125 mmol, 1.1 eq), $PdCl_2$ (22.2 mg, 0.125 mmol, 1.1 eq) and KI (94.6 mg, 0.568 mmol, 5.0 eq). The resulting mixture was stirred under argon at 84 °C for 18 h. Pyridine was evaporated under vacuum and the crude product was purified by SiO_2 chromatography [(Hexanes/EtOAc (7:3)] to yield pure ***trans*-15** as an orange solid (64 mg, 64 %).

^{1}H NMR (400 MHz, $CDCl_3$) δ 8.22 (dd, *J* = 1.5, 6.5Hz, 2H), 7.88 (dd, *J* = 7.9, 1.6 Hz, 2H), 7.77 (d, *J* = 8.8 Hz, 2H, *p*-OMe-C_6H_4), 7.63-7.52 (m, 4H), 7.35 (d, *J* = 9.1 Hz, 2H, *p*-OMe-C_6H_4), 7.06 (dd, *J* = 7.8, 6.3 Hz, 2H), 6.94 (d, *J* = 8.8 Hz, 2H, *p*-OMe-C_6H_4), 6.80 (d, *J* = 9.1 Hz, 2H, *p*-OMe-C_6H_4), 6.71 (d, *J* = 2.0 Hz, 1H, β-lactam), 5.60 (d, *J* = 2.0 Hz, 1H, β-lactam), 4.03 (s, 3H, CH_3, N–CH_3), 3.81 (s, 3H, CH_3, *p*-OMe-C_6H_4), 3.74 (s, 3H, CH_3, *p*-OMe-C_6H_4). ^{13}C NMR (101 MHz, $CDCl_3$) δ 160.4 (C, *p*-OMe-C_6H_4), 157.4 (C, C=O, β-lactam), 156.9 (C, *p*-OMe-C_6H_4), 153.5 (2CH), 145.3 (C), 137.4 (C), 137.3 (CH), 130.6 (2CH), 130.3 (CH), 130.2 (2CH, *p*-OMe-C_6H_4), 130.1 (C), 128.9 (2CH), 127.1 (C), 126.7 (C), 124.1 (2CH), 119.5 (2CH, *p*-OMe-C_6H_4), 114.6 (2CH, *p*-OMe-C_6H_4), 114.5 (2CH, *p*-OMe-C_6H_4), 76.2 (CH, β-lactam), 62.6 (CH, β-lactam), 55.6 (CH_3, *p*-OMe-C_6H_4), 55.5 (CH_3, *p*-OMe-C_6H_4), 38.0 (CH_3, N-CH_3). IR: ν_{max} 1758, 1509, 1245, 1176 cm^{-1}. HRMS (ESI) *m/z* calculated for $C_{28}H_{27}IN_5O_3Pd$: 714.0199 $[M - I - py + MeCN]^+$; found 714.0185. Mp: (dec.)

Azide 17a

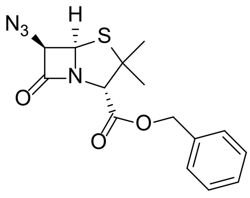

The synthesis of azide **17a**,[9] has been carried out under the following conditions: freshly distilled NEt_3 (1.40 mL, 9.82 mmol, 2.0 eq) was added dropwise to a suspension of the tosylate salt **16a** (2.35 g, 4.91 mmol, 1.0 eq) in anhydrous CH_2Cl_2 (90 mL), under an argon atmosphere, and stirred at room temperature for 45 min. Next, NfN_3 (3.19 g, 9.82 mmol, 2.0 eq) was added and the mixture was stirred for 6 h until the disappearance of starting material (TLC analysis). Solvents are removed in vacuo and the residue was submitted to SiO_2 chromatography [(Hexane/AcOEt) (9:1)] to yield pure **17a** (1.11 g, 68%).

^{1}H NMR (300 MHz, $CDCl_3$) δ 7.37 (s, 5H, Ph), 5.48 (d, *J* = 4.0 Hz, 1H, β-lactam), 5.19 (s, 2H, CH_2, CH_2Ph), 4.92 (d, *J* = 4.0 Hz, 1H, β-lactam), 4.50 (s, 1H, β-lactam), 1.65 (s, 3H, CH_3), 1.42 (s, 3H, CH_3).

Azide 17b

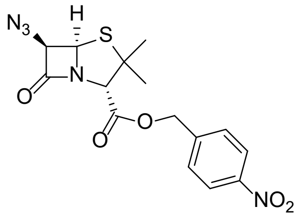

The synthesis of azide **17b**,[10] has been carried out under the following conditions: freshly distilled NEt_3 (0.62 ml, 4.46 mmol, 2.0 eq) was added dropwise to a suspension of the tosylate salt **16b** (1.17 g, 2.23 mmol, 1.0 eq) in anhydrous CH_2Cl_2 (40 mL), under an argon atmosphere, and stirred at room temperature for 45 min. Next, NfN_3 (1.45 g, 4.46 mmol, 2.0 eq) was added and the mixture was stirred for 6 h until the disappearance of starting material (TLC analysis). Solvents were removed *in vacuo* and the residue was submitted to SiO_2 chromatography [(Hexane/AcOEt) (9:1)] to yield pure **17b** (650 mg, 77%).

^{1}H NMR (300 MHz, $CDCl_3$) δ 8.25 (d, *J* = 8.9 Hz, 2H, *p*-NO_2-C_6H_4), 7.55 (d, *J* = 8.9 Hz, 2H, *p*-NO_2-C_6H_4), 5.48 (d, *J* = 4.1 Hz, 1H, β-lactam), 5.36-5.20 (m, 2H, CH_2, CH_2Ph), 4.95 (d, *J* = 4.1 Hz, 1H, β-lactam), 4.54 (s, 1H, β-lactam), 1.67 (s, 3H, CH_3), 1.43 (s, 3H, CH_3).

Triazole 18a

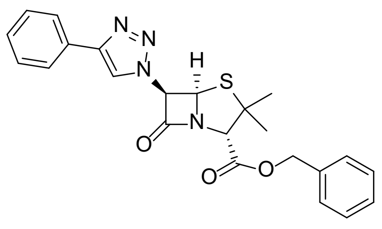

A mixture of azide **17a** (170 mg, 0.51 mmol, 1.0 eq), phenylacetylene (67.4 µl, 0.61 mmol, 1.2 eq), sodium (*L*)-ascorbate (50.7 mg, 0.26 mmol, 0.5 eq) and $CuSO_4·5H_2O$ (38.3 mg, 0.15 mmol, 0.3 eq) in DMF (6.0 mL) was stirred under argon at rt for 1 h. The solvents were removed under *vacuum* and the crude was purified to SiO_2 chromatography [(Hexanes/EtOAc (1:1)] to yield triazole **18a** as a white solid (204 mg, 93%).

^{1}H NMR (400 MHz, $CDCl_3$) δ 8.03 (s, 1H, N_3C=CH), 7.85 (br d, *J* = 7.0 Hz, 2H, Ph), 7.40 (m, 8H, Ph), 6.40 (d, *J* = 4.2 Hz, 1H, β-lactam), 5.80 (d, *J* = 4.2 Hz, 1H, β-lactam), 5.29-5.17 (m, 2H, O-CH_2), 4.60 (s, 1H, β-lactam), 1.70 (s, 3H, CH_3), 1.43 (s, 3H, CH_3). ^{13}C NMR (101 MHz, $CDCl_3$) δ 168.4 (C, C=O, β-lactam), 167.3 (C, C=O, $PhCH_2O$-C=O), 147.7 (C, N_3C=CH), 134.6 (C, Ph), 130.2 (C, Ph), 129.1 (CH, Ph), 129.0 (2CH, Ph), 128.9 (4CH, Ph), 128.6 (CH, Ph), 126.0 (2CH, Ph), 120.6 (CH, N_3C=CH), 71.0 (CH, β-lactam), 68.0 (CH_2, CH_2-Ph), 67.4 (CH, β-lactam), 66.4 (CH, β-lactam), 65.6 (C, $C(CH_3)_2$), 31.0 (CH_3), 27.2 (CH_3). IR: ν_{max} 1783, 1742, 1314, 1303, 1196, 1174, 1150, 1025, 964, 776, 753, 746, 699, 496 cm^{-1}. HRMS (ESI) m/z calculated for $C_{23}H_{23}N_4O_3S$: 435.1485 [M + H]$^+$; found 435.1477. Mp: 148 °C. $[\alpha]^{25}_D$ = + 170.5430 (*c* 0.86, $CHCl_3$).

Triazole 18b

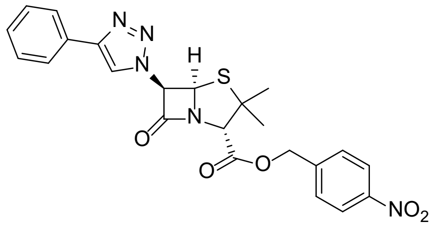

A mixture of azide **17b** (438 mg, 1.16 mmol, 1.0 eq), phenylacetylene (142 mg, 1.39 mmol, 1.2 eq), sodium (L)-ascorbate (115 mg, 0.58 mmol, 0.5 eq) and $CuSO_4·5H_2O$ (87 mg, 0.35 mmol, 0.3 eq) in DMF (15 mL) was stirred under argon at rt for 1 h. The solvents were removed under *vacuum* and the crude was purified to SiO_2 chromatography [(Hexanes/EtOAc (1:1)] to yield triazole **18b** as a white solid (375 mg, 66 %).

^{1}H NMR (500 MHz, $CDCl_3$) δ 8.27 (d, *J* = 8.8 Hz, 2H, *p*-NO_2-C_6H_4), 8.02 (s, 1H, N_3C=CH), 7.84 (m, 2H, Ph), 7.57 (d, *J* = 8.8 Hz, 2H, *p*-NO_2-C_6H_4), 7.44 (t, *J* = 7.5 Hz, 2H, Ph), 7.36 (m, 1H, Ph), 6.42 (d, *J* = 4.2 Hz, 1H, β-lactam), 5.81 (d, *J* = 4.2 Hz, 1H, β-lactam), 5.31 (m, 2H, O-CH_2), 4.64 (s, 1H, β-lactam), 1.73 (s, 3H, CH_3), 1.46 (s, 3H, CH_3).^{13}C NMR (126 MHz,

$CDCl_3$) δ 168.4 (C), 167.1 (C), 148.3 (C), 147.8 (C), 141.6 (C), 130.1 (C), 129.3 (2CH), 129.0 (2CH), 128.7 (CH), 126.0 (2CH), 124.2 (2CH), 120.5 (CH), 70.9 (CH, β-lactam), 67.5 (CH, β-lactam), 66.4 (CH, β-lactam), 66.3 (CH_2, CH_2-*p*-NO_2-C_6H_4), 65.5 (C), 31.0 (CH_3), 27.3 (CH_3). IR: ν_{max} 1787, 1741, 1517, 1347, 1315 cm^{-1}. HRMS (ESI) m/z calculated for $C_{23}H_{22}N_5O_5S$: 480.1336 $[M + H]^+$; found 480.1357. Mp: 165 °C. $[\alpha]^{25}_D$ = + 182.402 (*c* 0.63, $CHCl_3$).

Triazolium salt 19a

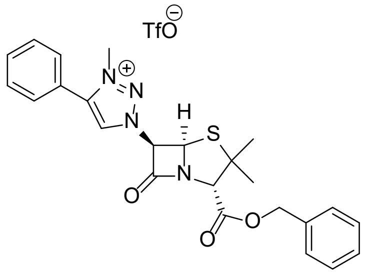

MeOTf (120 µL, 1.07 mmol, 1.5 eq) was added to a solution of the triazole **18a** (309 mg, 0.71 mmol, 1.0 eq) in CH_2Cl_2 (13 mL) at 10 ˚C. The mixture was stirred at 10˚C overnight. The solvent was removed under reduced pressure and the resulting residue was dissolved in the minimum amount of CH_2Cl_2 and precipitated with Et_2O. The solvent was decanted and the solid was washed with Et_2O (x3). The solvent was removed under *vacuum* to give pure triazolium salt **19a** (364 mg, 86%) as a white solid.

^1H NMR (400 MHz, $CDCl_3$) δ 8.98 (s, 1H, N_3C=CH), 7.64-7.52 (m, 5H, Ph), 7.38 (s, 5H, Ph), 6.80 (d, *J* = 4.3 Hz, 1H, β-lactam), 5.99 (d, *J* = 4.3 Hz, 1H, β-lactam), 5.21 (s, 2H, CH_2Ph), 4.62 (s, 1H, β-lactam), 4.32 (s, 3H, CH_3, N-CH_3), 1.67 (s, 3H, CH_3), 1.40 (s, 3H, CH_3). ^{13}C NMR (101 MHz, $CDCl_3$) δ 166.6 (C, C=O, $PhCH_2O$-C=O), 163.6 (C, C=O, β-lactam), 143.8 (C, N_3C=CH), 134.4 (C, Ph), 132.3 (CH, Ph), 129.9 (2CH, Ph), 129.5 (CH, Ph), 129.4 (2CH, Ph), 128.9 (CH, N_3C=CH), 128.8 (4CH, Ph), 121.2 (C, Ph), 70.5 (CH, β-lactam), 69.5 (CH, β-lactam), 67.8 (CH_2, CH_2-Ph), 67.7 (CH, β-lactam), 65.8 (C, $C(CH_3)_2$), 39.1 (CH_3, N-CH_3), 32.5 (CH_3), 26.5 (CH_3). IR: ν_{max} 1795, 1741, 1257, 1153, 1029, 636 cm^{-1}. HRMS (ESI) m/z calculated for $C_{24}H_{25}N_4O_3S$: 449.1642 $[M]^+$; found 449.1633. Mp: 65 °C. $[\alpha]^{25}_D$ = + 122.711 (*c* 0.86, $CHCl_3$).

Triazolium salt 19b

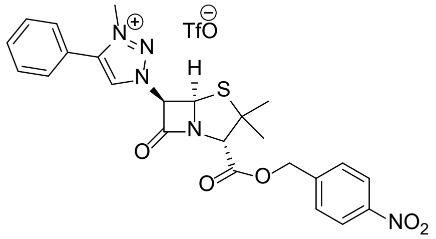

MeOTf (94 µL, 0.86 mmol, 1.5 eq) was added to a solution of triazole **18b** (275 mg, 0.57 mmol, 1.0 eq) in CH_2Cl_2 (15 ml) at 10 ˚C. The mixture was stirred at 10˚C in a cold room laboratory overnight. The solvent was removed under reduced pressure and

the resulting residue was dissolved in the minimum amount of CH_2Cl_2 and precipitated with Et_2O. The solvent was decanted and the solid is washed with Et_2O (x3). The solvent was removed under *vacuum* to give pure triazolium salt **19b** (320 mg, 86%) as a white solid.

^{1}H NMR (500 MHz, $CDCl_3$) δ 9.03 (s, 1H, $N_3C{=}CH$), 8.26 (d, *J* = 8.7 Hz, 2H, *p*-NO_2-C_6H_4), 7.67-7.54 (m, 7H, *p*-NO_2-C_6H_4 + Ph), 6.89 (d, *J* = 4.2 Hz, 1H, β-lactam), 6.06 (d, *J* = 4.2 Hz, 1H, β-lactam), 5.38-5.28 (m, 2H, CH_2), 4.68 (s, 1H, β-lactam), 4.33 (s, 3H, N-CH_3), 1.70 (s, 3H, CH_3), 1.43 (s, 3H, CH_3).^{13}C NMR (126 MHz, $CDCl_3$) δ 166.4 (C), 163.7 (C), 148.2 (C), 143.8 (C), 141.6 (C), 132.6 (CH), 130.1 (2CH), 129.8 (CH), 129.6 (2CH), 129.3 (2CH), 124.2 (2CH), 121.3 (C), 70.6 (CH), 69.8 (CH, β-lactam), 68.1 (CH, β-lactam), 66.3 (CH_2), 65.9 (C), 39.3 (CH_3), 32.8 (CH_3), 26.7 (CH_3). IR: ν_{max} 1795, 1746, 1256, 1153, 1029, 636 cm^{-1}. HRMS (ESI) m/z calculated for $C_{24}H_{24}N_5O_5S$: 494.1493 $[M]^+$; found 494.1492. Mp: 70 °C. $[\alpha]^{25}_D$ = + 115.119 (*c* 0.52, $CHCl_3$).

Compound 22

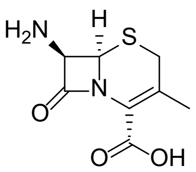

To a solution of 7-ACA (3.0 g, 11.0 mmol, 1 eq) in freshly distilled trifluoroacetic acid (21 mL) is added Et_3SiH (21 mL) followed by BF_3OEt_3 (13 mL). The reaction is exothermic, so the addition is carried out with an ice bath. After 5 min, the bath is removed and the mixture was cooled to room temperature and diluted with Et_2O, the precipitate was filtered and dried. The solid was dissolved in water and the pH was adjusted to pH = 4 with 10 M NaOH solution to induce the precipitation of the product in its zwitterionic form. The solid was dried to obtain **22** as a white solid (1.28 g, 54%).

^{1}H NMR (400 MHz, DMSO-D_6) δ 4.89 (d, *J* = 4.8 Hz, 1H, β-lactam), 4.68 (d, *J* = 4.8 Hz, 1H, β-lactam), 3.52 (d, *J* = 18.1 Hz, 1H, S-CH_2), 3.26 (d, *J* = 18.1 Hz, 1H, S-CH_2), 1.94 (s, 3H, CH_3). ^{13}C NMR (101 MHz, DMSO-D_6) δ 169.3 (C, C=O, β-lactam), 163.9 (C, C=O, COOH), 128.3 (C, $CH_3C{=}CCOOH$), 122.8 (C, $CH_3C{=}CCOOH$), 63.1 (CH, β-lactam), 58.4 (CH, β-lactam), 28.5 (CH_2, S-CH_2), 19.5 (CH_3,). IR: ν_{max} 3148, 1786, 1532, 1516, 1408, 1347, 795, 786 cm^{-1}. HRMS (ESI) m/z calculated for $C_8H_{10}N_2O_3SNa$: 237.0304 $[M + Na]^+$; found 237.0299. Mp: (dec.).

Compound 24

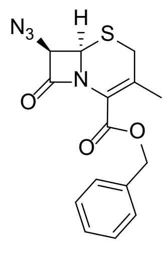

Et_3N (0.10 ml, 0.724 mmol, 1.5 eq) was added dropwise to a suspension of the tosylate salt **23** (230 mg, 0.483 mmol, 1 eq) in anhydrous CH_2Cl_2 (10 ml), under argon atmosphere, and the mixture was stirred at room temperature for 45 min. Then, NfN_3 (313 mg, 0.966 mmol, 3.0 eq) was added and the mixture was stirred until tlc analysis show the disappearance of the cephalosporine azide (5 h). The volatiles were removed under *vacuum* and the residue was submitted to SiO_2 chromatography [(Hexanes/EtOAc)(8:2)] to yield pure azide **24** as an oil (160 mg, 90 %).

^1H NMR (400 MHz, $CDCl_3$) δ 7.46-7.30 (m, 5H, Ph), 5.26 (s, 2H, CH_2, CH_2Ph), 5.03 (d, J = 4.7 Hz, 1H, β-lactam), 4.93 (d, J = 4.7 Hz, 1H, β-lactam), 3.46 (d, J = 18.1 Hz, 1H, S-CH_2), 3.24 (d, J = 18.1 Hz, 1H, S-CH_2), 2.16 (s, 3H, CH_3). ^{13}C NMR (101 MHz, $CDCl_3$) δ 162.1 (C, C, C=O, O=C-CH_2Ph), 161.6 (C, C=O, β-lactam), 135.1 (C, Ph), 134.2 (C, CH_3C=CCOOPh), 128.9 (2CH, Ph), 128.8 (2CH, Ph), 128.7 (CH, Ph), 122.6 (C, CH_3C=CCOOPh), 67.9 (CH_2, CH_2Ph), 67.2 (CH, β-lactam), 57.5 (CH, β-lactam), 30.7 (CH_2, S-CH_2), 20.4 (CH_3). IR: ν_{max} 2117, 1766, 1718, 1216, 1190, 1130 cm^{-1}. HRMS (ESI) m/z calculated for $C_{15}H_{15}N_4O_3S$: 331.0859 $[M + H]^+$; found 331.0859. $[\alpha]^{25}_D$ = + 8.041 (*c* 0.88, $CHCl_3$).

Compound 25

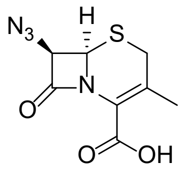

The synthesis of azide **25**[11] was carried out under the following conditions: $NaHCO_3$ (314 mg, 3.73mmol, 4.0 eq) and a solution of NfN_3 (455 mg, 1.40 mmol, 1.5 eq) in Et_2O (1.9 mL) were added to a solution of β-lactam **22** (200 mg, 0.93 mmol, 1.0 eq) in water (2.5 mL), MeOH (6.8 mL), and DMF (5 mL). The resulting mixture was stirred at room temperature for 6h. After that time, the solution was diluted in water, acidified with a 1M HCl solution and then extracted with AcOEt. Compound **25** was obtained after drying (Na_2SO_4) and solvent removal under *vacuum* (201 mg, 90%).

^1H NMR (300 MHz, DMSO-D_6) δ 13.26 (s, 1H, OH), 5.59 (d, J = 4.8 Hz, 1H, β-lactam), 5.10 (d, J = 4.8 Hz, 1H, β-lactam), 3.61 (d, J = 17.8 Hz, 1H, S-CH_2), 3.35 (d, J = 17.8 Hz, 1H, S-CH_2), 2.01 (s, 3H, CH_3).

Compound 26

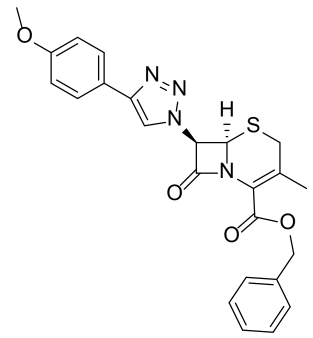

A mixture of azide **24** (200 mg, 0.605 mmol, 1.0 eq), 4-ethynylanisole (96.0 mg, 0.726 mmol, 1.2 eq), sodium (*L*)-ascorbate (60.0 mg, 0.303 mmol, 0.5 eq) and $CuSO_4·5H_2O$ (45.4 mg, 0.182 mmol, 0.3 eq) in DMF (10 mL) was stirred under argon at rt for 1 h. The solvents were removed under *vacuum* and the crude was purified to SiO_2 chromatography [(Hexanes/EtOAc (7:3)] to yield triazole **26** as a white solid (90 mg, 32 %).

^{1}H NMR (400 MHz, $CDCl_3$) δ 7.91 (s, 1H, N_3C=CH), 7.76 (d, *J* = 8.9 Hz, 2H, *p*-OMe-C_6H_4), 7.48-7.31 (m, 5H, Ph), 6.96 (d, *J* = 8.9 Hz, 2H, *p*-OMe-C_6H_4), 6.43 (d, *J* = 4.8 Hz, 1H, β-lactam), 5.31 (s, 2H, O-CH_2), 5.24 (d, *J* = 4.8 Hz, 1H, β-lactam), 3.84 (s, 3H, CH_3, *p*-OMe-C_6H_4), 3.49 (d, *J* = 18.2 Hz, 1H, S-CH_2), 3.28 (d, *J* = 18.2 Hz, 1H, S-CH_2), 2.19 (s, 3H, CH_3). ^{13}C NMR (101 MHz, $CDCl_3$) δ 161.7 (C, C=O, O=C-OCH_2Ph), 160.0 (C, *p*-OMe-C_6H_4), 159.5 (C, C=O, β-lactam), 147.6 (C, N_3C=CH), 135.1 (C, Ph), 133.9 (C, CH_3C=CCOOPh), 128.9 (2CH, Ph), 128.8 (2CH, Ph), 128.8 (CH, Ph), 127.3 (2CH, *p*-OMe-C_6H_4), 122.8 (C, *p*-OMe-C_6H_4), 122.6 (C, CH_3C=CCOOPh), 120.0 (CH, N_3C=CH), 114.4 (2CH, *p*-OMe-C_6H_4), 68.0 (CH_2 O-CH_2), 67.2 (CH, β-lactam), 57.1 (CH, β-lactam), 55.5 (CH_3, *p*-OMe-C_6H_4), 30.8 (CH_2, S-CH_2), 20.3 (CH_3). IR: ν_{max} 1777, 1718, 1247, 1217, 1148, 1029, 834, 806, 728 cm^{-1}. HRMS (ESI) m/z calculated for $C_{24}H_{23}N_4O_4S$: 463.1435 $[M + H]^+$; found 463.1443. Mp: 155-160 °C. $[\alpha]^{25}_D$ = + 12.763 (*c* 0.68, $CHCl_3$).

Compound 27

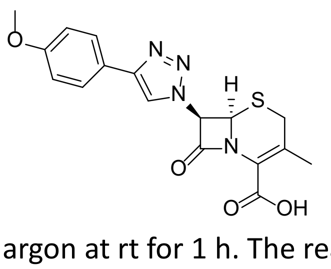

A mixture of azide **25** (310 mg, 1.29 mmol, 1.0 eq), 4-ethynylanisole (205 mg, 1.55 mmol, 1.2 eq), sodium (*L*)-ascorbate (128 mg, 0.65 mmol, 0.5 eq) and $CuSO_4·5H_2O$ (97 mg, 0.39 mmol, 0.3 eq) in DMF (15.0 mL) was stirred under argon at rt for 1 h. The reaction mixture was diluted with water and adjusted to acid pH with 1M HCl solution, then extracted with AcOEt. The organic layer was dried over anhydrous Na_2SO_4 and solvents were removed under *vacuum*. The resulting residue was dissolved in the minimum amount of CH_2Cl_2 and precipitated with Et_2O. The solvents were decanted, and the solid was washed with Et_2O (×3) and vacuum-dried to yield pure triazole **27** (194 mg, 40 %).

^{1}H NMR (400 MHz, DMSO-D_6) δ 8.55 (s, 1H, N_3C=CH), 7.86 (d, *J* = 8.9 Hz, 2H, *p*-OMe-C_6H_4), 7.01 (d, *J* = 8.9 Hz, 2H, *p*-OMe-C_6H_4), 6.84 (d, *J* = 4.8 Hz, 1H, β-lactam), 5.43 (d, *J* = 4.8 Hz, 1H, β-lactam), 3.79 (s, 3H, CH_3, *p*-OMe-C_6H_4), 3.59 (d, *J* = 17.7 Hz, 1H, S-CH_2), 3.45 (d, *J* = 17.7 Hz, 1H, S-CH_2), 2.10 (s, 3H, CH_3). ^{13}C NMR (101 MHz, DMSO-D_6) δ 163.3 (C, C=O, COOH), 159.6 (C, C=O, β-lactam), 159.3 (C, *p*-OMe-C_6H_4), 146.3 (C, N_3C=CH), 133.7 (C, CH_3C=CCOOH), 126.8 (2CH, *p*-OMe-C_6H_4), 122.9 (C, CH_3C=CCOOH), 122.7 (C, *p*-OMe-C_6H_4), 121.3 (CH, N_3C=CH), 114.4 (2CH, *p*-OMe-C_6H_4), 66.7 (CH, β-lactam), 56.9 (CH, β-lactam), 55.2 (CH_3, *p*-OMe-C_6H_4), 29.5 (CH_2, S-CH_2), 19.5 (CH_3). IR: ν_{max} 3139, 1766, 1727, 1500, 1400, 1139, 825 cm^{-1}. HRMS (ESI) m/z calculated for $C_{17}H_{17}N_4O_4S$: 373.0965 [M + H]$^+$; found 373.0962. Mp: 205 °C. $[\alpha]^{25}_D$ = + 24.8082 (*c* 0.57, DMF).

Compound 20

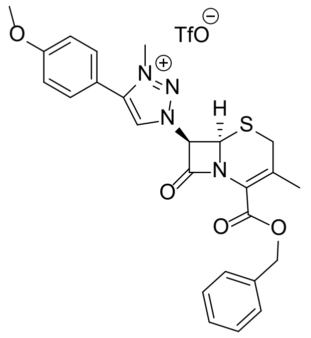

MeOTf (20 µl, 0.18 mmol, 1.5 eq) was added To a solution of triazole **26** (55 mg, 0.12 mmol, 1 eq) in CH_2Cl_2 (12.0 ml) at 10 ˚C. The mixture was stirred at 10˚C in a cold room laboratory overnight. The solvent was removed under reduced pressure and the resulting residue was dissolved in the minimum amount of CH_2Cl_2 and precipitated with Et_2O. The solvent was decanted and the solid washed with Et_2O (x3). The solvent was removed to dryness giving pure triazolium salt **20** (50 mg, 70%) as a white solid.

^{1}H NMR (500 MHz, $CDCl_3$) δ 8.97 (s, 1H, N_3C=CH), 7.54 (d, *J* = 8.4 Hz, 2H, *p*-OMe-C_6H_4), 7.43-7.29 (m, 5H, Ph), 7.07 (d, *J* = 8.9 Hz, 2H, *p*-OMe-C_6H_4), 6.79 (d, *J* = 4.4 Hz, 1H, β-lactam), 5.48 (d, *J* = 4.4 Hz, 1H, β-lactam), 5.27 (s, 2H, O-CH_2), 4.29 (s, 3H, N-CH_3), 3.87 (s, 3H, O-CH_3), 3.32 (q, *J* = 16.8 Hz, 2H, S-CH_2), 2.26 (s, 3H, CH_3). ^{13}C NMR (126 MHz, $CDCl_3$) δ 162.8 (C, *p*-OMe-C_6H_4), 161.4 (C, C=O, O=C-OCH_2Ph), 156.6 (C, C=O, β-lactam), 143.9 (C, N_3C=CH), 142.66 (C, CH_3C=CCOOPh), 135.2 (C, Ph), 131.1 (2CH, *p*-OMe-C_6H_4), 129.9 (CH, N_3C=CH), 128.8 (2CH, Ph), 128.7 (CH, Ph), 128.6 (2CH, Ph), 123.3 (C, CH_3C=CCOOPh), 115.6 (2CH, *p*-OMe-C_6H_4), 113.0 (C, *p*-OMe-C_6H_4), 70.3 (CH, β-lactam), 67.7 (CH_2, O-CH_2), 58.6 (CH, β-lactam), 55.8 (CH_3, O-CH_3), 39.3 (CH_3, N-CH_3), 31.7 (CH_2, S-CH_2), 20.3 (CH_3). IR: ν_{max} 1785, 1718, 1254, 1222, 1150,1028, 636 cm^{-1}. HRMS (ESI) m/z calculated for $C_{25}H_{25}N_4O_4S$: 477.1591 [M]$^+$; found 477.1590. Mp: 70 °C. $[\alpha]^{25}_D$ = + 55.289 (*c* 0.44, $CHCl_3$).

Compound 21.

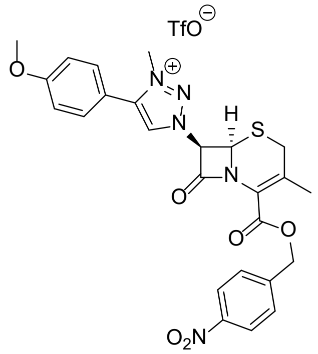

K_2CO_3 (58 mg, 0.42 mmol, 1 eq) was added to a solution of triazole **26** (157 mg, 0.42 mmol, 1 eq) in anhydrous DMF (5.0 mL), and the mixture was stirred at 0°C for 30 min. Then 4-nitrobenzyl bromide (109 mg, 0.51 mmol, 1.2 eq) was added, and the mixture stirred at room temperature for 5 h. The volatiles were removed under vacuum. The crude product was purified by SiO_2 chromatography [(Hexanes:EtOAc (7:3)] to yield the corresponding *p*-nitrobenzyl ester as a white solid (94.5 g, 44 %).

^{1}H NMR (400 MHz, $CDCl_3$) δ 8.24 (d, *J* = 8.7 Hz, 2H, *p*-NO_2-C_6H_4), 7.90 (s, 1H, N_3C=CH), 7.76 (d, *J* = 8.8 Hz, 2H, *p*-OMe-C_6H_4), 7.62 (d, *J* = 8.7 Hz, 2H, *p*-NO_2-C_6H_4), 6.96 (d, *J* = 8.8 Hz, 2H, *p*-OMe-C_6H_4), 6.46 (d, *J* = 4.8 Hz, 1H, β-lactam),5.46-5.31 (m, 2H, O-CH_2), 5.28 (d, *J* = 4.8 Hz, 1H, β-lactam), 3.84 (s, 3H, CH_3, *p*-OMe-C_6H_4), 3.53 (d, *J* = 18.3 Hz, 1H, S-CH_2), 3.34 (d, *J* = 18.3 Hz, 1H, S-CH_2), 2.23 (s, 3H, CH_3). ^{13}C NMR (101 MHz, $CDCl_3$) δ 161.4 (C, C=O, O=C-OCH_2), 160.0 (C, *p*-OMe-C_6H_4), 159.8 (C, C=O, β-lactam), 148.0 (C, *p*-NO_2-C_6H_4), 147.7 (C, N_3C=CH), 142.3 (C, *p*-NO_2-C_6H_4), 135.6 (C, CH_3C=CCOO), 129.2 (2CH, *p*-NO_2-C_6H_4), 127.3 (2CH, *p*-OMe-C_6H_4), 124.0 (2CH, *p*-NO_2-C_6H_4), 122.7 (C, *p*-OMe-C_6H_4), 122.1 (C, CH_3C=CCOO), 120.0 (CH, N_3C=CH), 114.4 (2CH, *p*-OMe-C_6H_4), 67.7 (CH, β-lactam), 66.3 (CH_2, O-CH_2), 57.2 (CH, β-lactam), 55.5 (CH_3, *p*-OMe-C_6H_4), 30.9 (CH_2,S-CH_2), 20.4 (CH_3). IR: ν_{max} 1776, 1722, 1345, 1247, 1218, 836, 803 cm^{-1}. HRMS (ESI) m/z calculated for $C_{24}H_{22}N_5O_6S$: 508.1285 [M + H]$^+$; found 508.1284. Mp: 185 °C. $[\alpha]^{25}_D$ = + 59.633 (*c* 0.48, $CHCl_3$).

MeOTf (31.6 µL, 0.28 mmol, 1.5 eq) was added to a solution of the triazole p-nitrobenzyl ester (94.5 mg, 0.19 mmol, 1.0 eq) in CH_2Cl_2 (20.0 ml) at 10 Ȼ. The mixture was stirred at º10 C in a cold room laboratory overnight. The solvent was removed under reduced pressure and the resulting residue is dissolved in the minimum amount of CH_2Cl_2 and precipitated with Et_2O. The solvent was decanted and the solid is washed with Et_2O (x3). The solvent was removed to dryness giving pure triazolium salt **21** (117 mg, 94%) as a white solid.

^{1}H NMR (400 MHz, $CDCl_3$) δ 9.00 (s, 1H, N_3C=CH), 8.21 (d, *J* = 8.5 Hz, 2H, *p*-NO_2-C_6H_4), 7.59 (d, *J* = 8.5 Hz, 2H, *p*-NO_2-C_6H_4), 7.53 (d, *J* = 8.7 Hz, 2H, *p*-OMe-C_6H_4), 7.06 (d, *J* = 8.7

Hz, 2H, *p*-OMe-C_6H_4), 6.89 (d, *J* = 4.6 Hz, 1H, β-lactam), 5.53 (d, *J* = 4.6 Hz, 1H, β-lactam), 5.41 (d, *J* = 13.3 Hz, 1H, O-CH_2), 5.29 (d, *J* = 13.3 Hz, 1H, O-CH_2), 4.33 (s, 3H, N-CH_3), 3.87 (s, 3H, O-CH_3), 3.35 (q, *J* = 16.7 Hz, 2H, S-CH_2), 2.31 (s, 3H, CH_3). ^{13}C NMR (101 MHz, $CDCl_3$) δ 162.8 (C, *p*-OMe-C_6H_4), 161.0 (C, C=O, O=C-OCH_2), 156.8 (C, C=O, β-lactam), 147.9 (C, *p*-NO_2-C_6H_4), 144.7 (C, CH_3C=CCOO), 144.0 (C, N_3C=CH), 142.5 (C, *p*-NO_2-C_6H_4), 131.1 (2CH, *p*-OMe-C_6H_4), 130.0 (CH, N_3C=CH), 128.9 (2CH, *p*-NO_2-C_6H_4), 124.0 (2CH, *p*-NO_2-C_6H_4), 122.8 (C, CH_3C=CCOO), 115.6 (2CH, *p*-OMe-C_6H_4), 112.9 (C, *p*-OMe-C_6H_4), 70.4 (CH, β-lactam), 66.1 (CH_2, O-CH_2), 58.8 (CH, β-lactam), 55.8 (CH_3, O-CH_3), 39.4 (CH_3, N-CH_3), 31.8 (CH_2, S-CH_2), 20.3 (CH_3). IR: ν_{max} 1786, 1725, 1284, 1222, 1029, 637 cm^{-1}. HRMS (ESI) m/z calculated for $C_{25}H_{24}N_5O_6S$: 522.1442 [M]$^+$; found 522.1443. Mp: 95 °C. $[\alpha]^{25}_D$ = + 58.690 (*c* 0.63, $CHCl_3$).

General procedure for the synthesis of Ir complexes derived from penicillins and cephalosporins

A mixture of the triazolium salt (1.0 eq), Cs_2CO_3 (2.0 eq), $[IrCl_2Cp^*]_2$ (0.5 eq), in anhydrous CH_2Cl_2 was stirred, under argon, at room temperature until the reaction was complete (analysis by ^{1}H-NMR). The reaction mixture was filtered through a pad of celite, the solvent was then removed under reduced pressure and the crude product was purified by column chromatography (SiO_2) to yield the corresponding complexes metal complexes.

Compound 28a

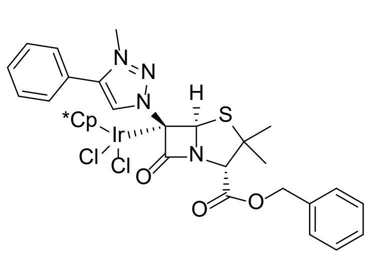

A mixture of the triazolium salt **19a** (250 mg, 0.42 mmol, 1.0 eq) Cs_2CO_3 (272 mg, 0.84 mmol, 2.0 eq) and $[IrCl_2Cp^*]_2$ (167 mg, 0.21 mmol, 0.5 eq) in CH_2Cl_2 (35 mL) was stirred, under argon, at room temperature for 2 h. The crude was purified (SiO_2, Hexane/AcOEt (1:4)) to yield **28a** as an orange solid (156 mg, 44%).

^{1}H NMR (400 MHz, $CDCl_3$) δ 8.85 (br s, 1H, N_3C=CH), 7.58-7.43 (m, 5H, Ph), 7.43-7.30 (m, 5H, Ph), 5.94 (s, 1H, β-lactam), 5.23 (d, *J* = 11.9 Hz, 1H, CH_2Ph), 5.27 (d, *J* = 11.9 Hz, 1H, CH_2Ph), 4.52 (s, 1H, β-lactam), 4.10 (s, 3H, N-CH_3), 1.53 (s, 18H, 5CH_3 Cp* + CH_3), 1.37(s, 3H, CH_3). ^{13}C NMR (101 MHz, $CDCl_3$) δ 176.4 (C, C=O, β-lactam) 168.4 (C, C=O, O=C-

OCH_2Ph), 139.8 (C), 135.0 (C, Ph), 131.3 (CH), 129.5 (2CH), 129.4 (3CH), 129.1 (2CH), 128.8 (3CH), 128.6 (C), 123.5 (C), 87.2 (5C, Cp*), 75.6 (CH, β-lactam), 70.4 (CH, β-lactam), 67.5 (CH_2), 64.5 (C, $C(CH_3)_2$), 37.7 (CH_3, N-CH_3), 29.8 (CH_3), 27.2 (CH_3), 8.17 (5CH_3, Cp*). IR: ν_{max} 1748, 1454, 1295, 1262, 1156, 1151, 1077, 1028, 766, 748, 696 cm^{-1}. HRMS (ESI) m/z calculated for $C_{34}H_{39}ClIrN_4O_3S$: 811.2047 $[M - Cl]^+$; found 811.2046. Mp: 120 °C. $[\alpha]^{25}_D$ = + 51.873 (*c* 0.51, $CHCl_3$).

Compound 28b

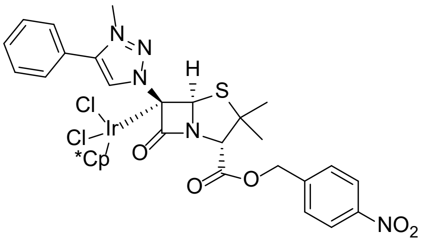

A mixture of the triazolium salt **19b** (200 mg, 0.31 mmol, 1.0 eq) Cs_2CO_3 (202 mg, 0.62 mmol, 2.0 eq) and $[IrCl_2Cp^*]_2$ (124 mg, 0.16 mmol, 0.5 eq) in CH_2Cl_2 (24 mL) was stirred, under argon, at room temperature for 2 h. The crude was purified (SiO_2, Hexane/AcOEt (1:4)) to yield **28b** as an orange solid (148 mg, 54%).

^1H NMR (500 MHz, $CDCl_3$) δ 9.09 (s, 1H, N_3C=CH), 8.24 (d, J = 8.4 Hz, 2H, *p*-NO_2-C_6H_4), 7.63 (d, J = 8.4 Hz, 2H, *p*-NO_2-C_6H_4), 7.55 (m, 3H, Ph), 7.49 (m, 2H, Ph), 6.06 (s, 1H, β-lactam), 5.29 (s, 2H, CH_2), 4.56 (s, 1H, β-lactam), 4.16 (s, 3H, N-CH_3), 1.55 (s, 3H, CH_3), 1.53 (s, 15H, 5CH_3, Cp*), 1.36 (s, 3H, CH_3). ^{13}C NMR (126 MHz, $CDCl_3$) δ 175.2 (C, C=O, β-lactam), 168.0 (C, C=O, O=C-OCH_2), 148.0 (C, *p*-NO_2-C_6H_4), 142.3 (C, *p*-NO_2-C_6H_4), 140.5 (C), 131.6 (CH, Ph), 129.7 (2CH, Ph), 129.2 (2CH, Ph), 129.2 (2CH, *p*-NO_2-C_6H_4), 128.6 (CH, N_3C=CH), 124.1 (2CH, *p*-NO_2-C_6H_4), 123.2 (C, Ph), 87.2 (5C, Cp*), 76.6 (CH, β-lactam), 70.3 (CH, β-lactam), 65.8 (CH_2), 64.6 (C, $C(CH_3)_2$), 37.8 (CH_3, N-CH_3), 31.3 (CH_3), 27.2 (CH_3), 8.30 (5CH_3, Cp*). IR: ν_{max} 2922, 2853, 1748, 1519, 1344, 734, 697 cm^{-1}. HRMS (ESI) m/z calculated for $C_{34}H_{38}ClIrN_5O_5S$: 856.1898 $[M - Cl]^+$; found 856.1901. Mp: 125 °C. $[\alpha]^{25}_D$ = + 139.626 (c 0.38, $CHCl_3$).

Compound 29a

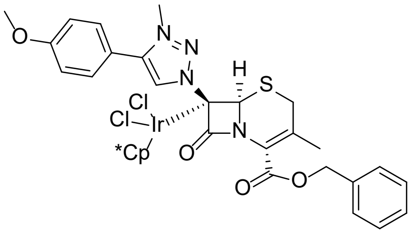

A mixture of the triazolium salt **20** (50 mg, 0.080 mmol, 1.0 eq) Cs_2CO_3 (52 mg, 0.16 mmol, 2.0 eq) and $[IrCl_2Cp^*]_2$ (32 mg, 0.040 mmol, 0.5 eq) in CH_2Cl_2 (7 mL) was stirred, under argon, at room temperature for

2 h. The crude was the purified (SiO_2, DCM/2% MeOH) to yield **29a** as an orange solid (26 mg, 38%).

^{1}H NMR (400 MHz, $CDCl_3$) δ 9.46 (s, 1H, N_3C=CH), 7.53 (d, *J* = 7.2 Hz, 2H, Ph), 7.41 (d, *J* = 8.8 Hz, 2H, *p*-OMe-C_6H_4), 7.35 (t, *J* = 7.3 Hz, 2H, Ph), 7.29 (d, *J* = 7.4 Hz, 1H, Ph), 7.05 (d, *J* = 8.8 Hz, 2H, *p*-OMe-C_6H_4), 5.60 (s, 1H, β-lactam), 5.30 (q, *J* = 12.6 Hz, 2H, O-CH_2), 4.17 (s, 3H, N-CH_3), 3.87 (s, 3H, *p*-OMe-C_6H_4), 3.47 (d, *J* = 18.0 Hz, 1H, S-CH_2), 3.05 (d, *J* = 18.0 Hz, 1H, S-CH_2), 1.99 (s, 3H, CH_3), 1.45 (s, 15H, 5CH_3 Cp*). ^{13}C NMR (101 MHz, $CDCl_3$) δ 164.5 (C, C=O, β-lactam), 163.0 (C, C=O, O=C-OCH_2Ph), 162.2 (C, *p*-OMe-C_6H_4), 140.6 (C), 135.6 (C, Ph), 130.5 (2CH, *p*-OMe-C_6H_4), 129.5 (CH, N_3C=CH), 128.6 (2CH, Ph), 128.5 (2CH, Ph), 128.0 (CH, Ph), 127.0 (C, CH_3C=CCOO), 123.3 (C, CH_3C=CCOO), 115.4 (2CH, *p*-OMe-C_6H_4), 114.6 (C, *p*-OMe-C_6H_4), 87.1 (5C, Cp*), 67.3 (CH_2, CH_2Ph), 64.6 (C), 64.2 (CH, β-lactam), 55.8 (CH_3, *p*-OMe-C_6H_4), 38.0 (CH_3, N-CH_3), 31.1 (CH_2, S-CH_2), 20.0 (CH_3), 8.55 (5CH_3, Cp*). IR: $ν_{max}$ 1752, 1721, 1612, 1503, 1256 cm^{-1}. HRMS (ESI) m/z calculated for $C_{35}H_{39}ClIrN_4O_4S$: 839.1997 $[M - Cl]^+$; found 839.2007. Mp: 125 °C. $[α]^{25}_D$ = + 126,630 (*c* 0.54, $CHCl_3$).

Compound 29b

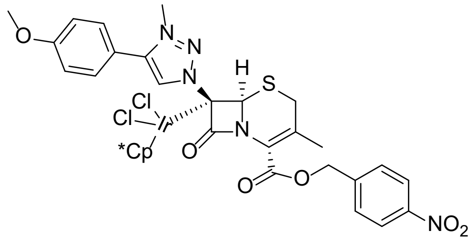

A mixture of the triazolium salt **21** (60 mg, 0.089 mmol, 1.0 eq) Cs_2CO_3 (58 mg, 0.18 mmol, 2.0 eq) and $[IrCl_2Cp^*]_2$ (36 mg, 0.045 mmol, 0.5 eq) in CH_2Cl_2 (7 mL) was stirred, under argon, at room temperature for 2 h. The crude was purified (SiO_2, Hexano/AcOEt (1:4)) to yield **29b** as an orange solid (39 mg, 55%).

^{1}H NMR (400 MHz, $CDCl_3$) δ 9.55 (s, 1H, N_3C=CH), 8.23 (d, *J* = 8.0 Hz, 2H, *p*-NO_2-C_6H_4), 7.80 (d, *J* = 8.0 Hz, 2H, *p*-NO_2-C_6H_4), 7.40 (d, *J* = 7.3 Hz, 2H, *p*-OMe-C_6H_4), 7.07 (d, *J* = 7.3 Hz, 2H, *p*-OMe-C_6H_4), 5.70 (s, 1H, β-lactam), 5.37 (s, 2H, CH_2), 4.21 (s, 3H, N-CH_3), 3.89 (s, 3H, *p*-OMe-C_6H_4), 3.51 (d, *J* = 17.6 Hz, 1H, S-CH_2), 3.08 (d, *J* = 17.6 Hz, 1H, S-CH_2), 2.03 (s, 3H, CH_3), 1.42 (s, 15H, 5CH_3 Cp*). ^{13}C NMR (101 MHz, $CDCl_3$) δ 164.3 (C, C=O, β-lactam), 162.6 (C, C=O, O=C-OCH_2), 162.4 (C, *p*-OMe-C_6H_4), 147.5 (C, *p*-NO_2-C_6H_4), 143.2 (C, *p*-NO_2-C_6H_4), 141.1 (C), 130.4 (2CH, *p*-OMe-C_6H_4), 129.3 (CH, N_3C=CH), 129.0 (C, CH_3C=CCOO), 128.6 (2CH, *p*-NO_2-C_6H_4), 123.8 (2CH, *p*-NO_2-C_6H_4), 122.8 (C, CH_3C=CCOO), 115.5 (2CH, *p*-OMe-C_6H_4), 114.3 (C), 87.1 (5C, Cp*), 65.6 (CH_2), 63.8 (CH, β-lactam), 63.7

(C), 55.8 (CH_3, *p*-OMe-C_6H_4), 38.1 (CH_3, N-CH_3), 31.2 (CH_2, S-CH_2), 19.9 (CH_3), 8.59. (5CH_3, Cp*). IR: ν_{max} 1751, 1725, 1520, 1504, 1345, 1258 cm^{-1}. HRMS (ESI) m/z calculated for $C_{35}H_{38}ClIrN_5O_6S$: 884.1848 $[M - Cl]^+$; found 884.1840. Mp: 140 °C. $[\alpha]^{25}_D$ = + 118.461 (*c* 0.36, $CHCl_3$).

Au(I) complex 30

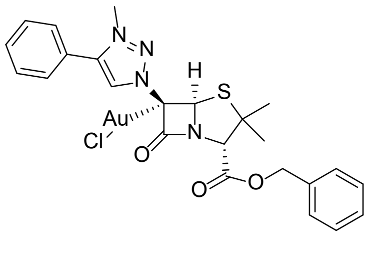

A mixture of the triazolium salt **19a** (50 mg, 0.084 mmol, 1.0 eq) K_2CO_3 (23 mg, 0.17 mmol, 2.0 eq) and $[AuCl(SMe_2)]$ (30 mg, 0.10mmol, 1.2 eq) in CH_2Cl_2 anhydrous (7.0 mL) was stirred, under an argon atmosphere, at room temperature for 3 h. The reaction mixture was filtered over celite and the solvent removed under reduced pressure. The crude oil was purified by column chromatography (SiO_2, Hexane/AcOEt (1:2)) giving rise to pure complex **30** as a white solid (29 mg, 51%).

^1H NMR (400 MHz, CD_3CN) δ 8.71 (s, 1H, N_3C=CH), 7.70-7.57 (m, 5H, Ph), 7.47-7.32 (m, 5H, Ph), 5.55 (s, 1H, β-lactam), 5.20 (s, 2H, CH_2), 4.62 (s, 1H, β-lactam), 4.13 (s, 3H, N-CH_3), 1.79 (s, 3H, CH_3), 1.37 (s, 3H, CH_3). ^{13}C NMR (101 MHz, CD_3CN) δ 170.3 (C), 168.7 (C), 143.2 (C), 136.5 (C), 132.5 (CH), 131.6 (CH), 130.4 (2CH), 130.3 (2CH), 129.5 (4CH), 129.4 (CH), 123.6 (C), 85.8 (C), 78.7 (CH, β-lactam), 69.9 (CH, β-lactam), 67.9 (CH_2), 65.3 (C), 39.3 (CH_3), 33.8 (CH_3), 26.4 (CH_3). IR: ν_{max} 1738, 1291, 1183, 1176, 1155, 765, 732, 696 cm^{-1}. HRMS (ESI) m/z calculated for $C_{26}H_{27}AuN_5O_3S$: 686.1495 $[M - Cl + MeCN]^+$; found 686.1482. Mp: 127 °C. $[\alpha]^{25}_D$ = + 109.341 (*c* 1.1, $CHCl_3$).

Single Crystal X-Ray Diffraction Analyses

Experimental

Crystals of ***trans*-12b**, **18b** and **28b** 0.25(C_6H_{14}) were analyzed by X-ray diffraction. A selection of crystal, measurement and refinement data is given in Table S1. Diffraction data were collected on a Bruker D8 Venture Photon III-14 single crystal diffractometer with Mo$K\alpha$ radiation. Empirical absorption corrections were applied using SADABS-2016/2[12] The structures were solved using SIR-97.[13] Isotropic and full matrix anisotropic least square refinements were carried out using SHELXL.[14] Non-H atoms, except the solvent atoms of **28b**·0.25(C_6H_{14}) were anisotropically refined. H atoms were placed riding on their parent atoms and were isotropically refined. The low resolution reflections 0 1 2 (of **18b**) and 1 1 0, 0 2 0, 0 2 2 and 0 1 3 (of **28b** 0.25(C_6H_{14}) were left out from their corresponding refinement because their intensity, likely affected by the beamstop, showed high S values. Hexane solvent molecules were found in the unit cell of **28b**·0.25(C_6H_{14}), which are possibly disordered, but no rational modelling of that disorder could be found, being the inclusion of a molecule of hexane, with occupancy 25 % per asymmetric unit, the best solution achieved. This solution required restraints on the geometrical and thermal parameters of the C atoms of the hexane molecule, which were isotropically refined due to their tendency to give nonpositive definite ellipsoids. The WINGX program system[15] was used throughout the structure determinations. The molecular plots were made with MERCURY.[16]

Table S1. Selected crystal and refinement data for the compounds studied by X-ray diffraction.

	***trans*-12b**	**18b**	**28b**·0.25(C_6H_{14})
formula	$C_{23}H_{24}AuClN_4O_4$	$C_{23}H_{21}N_5O_5S$	($C_{34}H_{37}Cl_2IrN_5O_5S$)·0.25($C_6H_{14}$)
fw	652.88	479.51	912.39
cryst syst	Monoclinic	Orthorhombic	Orthorhombic
space group	$P2_1/c$	$P2_12_12_1$	$P2_12_12_1$
a, Å	14.9033(7)	5.7343(2)	8.8455(2)
b, Å	11.0709(4)	11.8799(5)	20.8162(5)
c, Å	14.2963(6)	32.1308(15)	21.6127(15)
α, deg	90	90	90
β, deg	101.528(2)	90	90
γ, deg	90	90	90
V, Å^3	2311.20(17)	2188.84(16)	3979.5(3)
Z	4	4	4
F(000)	1272	1000	1822
D_{calcd}, g cm^{-3}	1.876	1.455	1.523
μ, mm^{-1} (Mo Kα)	6.519	0.196	3.587
cryst size, mm	0.10 x 0.09 x 0.01	0.24 x 0.12 x 0.11	0.09 x 0.07 x 0.03
T, K	100(2)	100(1)	100(1)
θ range, deg	2.31 a 26.37	2.54 a 27.87	1.89 a 27.99
min./max. h, k, l	–18/18, –13/13, –17/17	–7/7, –15/15, –42/42	–12/12, –28/29, –21/30
no. collected reflns	52441	34052	52638
no. unique reflns	4721	5204	9600
no. reflns with $I > 2\sigma(I)$	4165	4975	8576
no. params/restraints	301/0	309/0	460/7
GOF (on F^2)	1.054	1.061	1.068
R_1 (on F, $I > 2\sigma(I)$)	0.024	0.034	0.044
wR_2 (on F^2, all data)	0.055	0.083	0.099
min./max. $\Delta\rho$, e Å^{-3}	–1.206/0.5570	–0.380/0.528	–0.583/1.605
CCDC dep. no.	2313520	2313521	2313522

Computational Details

Calculations were performed at the DFT level using the M06 functional[17,18] with an ultrafine integration grid[18] as implemented in Gaussian 16.[19] Ir, Cl and S atoms were described using the scalarrelativistic Stuttgart-Dresden SDD pseudopotential[20] and its associated double-ζ basis set complemented with a set of f-polarization functions.[21] The 6-31G** basis set was used for the H, C, N, and O atoms.[22]All structures of the reactants, intermediates, transition states, and products were fully optimized in dichloromethane solvent (ε = 8.93) using the SMD continuum model.[23] All energies collected in the text are Gibbs energies in dichloromethane at 298 K.

References

1. Zarei, M., A straightforward approach to 2-azetidinones from imines and carboxylic acids using dimethyl sulfoxide and acetic anhydride. *Tetrahedron Lett.* **2014**, *55*, 5354-5357.
2. Asahina, Y.; Wurtz, N. R.; Arakawa, K.; Carson, N.; Fujii, K.; Fukuchi, K.; Garcia, R.; Hsu, M.-Y.; Ishiyama, J.; Ito, B.; Kick, E.; Lupisella, J.; Matsushima, S.; Ohata, K.; Ostrowski, J.; Saito, Y.; Tsuda, K.; Villarreal, F.; Yamada, H.; Yamaoka, T.; Wexler, R.; Gordon, D.; Kohno, Y., Discovery of BMS-986235/LAR-1219: A Potent Formyl Peptide Receptor 2 (FPR2) Selective Agonist for the Prevention of Heart Failure. *J. Med. Chem*. **2020**, *63*, 9003-9019.
3. Kametani, T.; Yokohama, S.; Shiratori, Y.; Aihara, S.; Fukumoto, K.; Satoh, F., Studies on the synthesis of heterocyclic compounds. 775. synthesis of β-lactams by acid chloride- and phosphate anhydride-imine methods. *Heterocycles* **1979**, *12*, 405-414.
4. Bose, A. K.; Jayaraman, M.; Okawa, A.; Bari, S. S.; Robb, E. W.; Manhas, M. S., Microwave-assisted rapid synthesis of α-amino-β-lactams. *Tetrahedron Lett.* **1996**, *37*, 6989-6992.
5. De Rosa, M.; Vigliotta, G.; Palma, G.; Saturnino, C.; Soriente, A., Novel Penicillin-Type Analogues Bearing a Variable Substituted 2-Azetidinone Ring at Position 6: Synthesis and Biological Evaluation. *Molecules* **2015**, *20*, 22044-22057.
6. Josephine, H. R.; Kumar, I.; Pratt, R. F., The Perfect Penicillin? Inhibition of a Bacterial DD-Peptidase by Peptidoglycan-Mimetic β-Lactams. *J. Am. Chem. Soc.* **2004**, *126*, 8122-8123.

7. a) NfN_3: Suárez, J. R.; Trastoy, B.; Pérez-Ojeda, M. E.; Marín-Barrios, R.; Chiara, J. L., Nonafluorobutanesulfonyl Azide: A Shelf-Stable Diazo Transfer Reagent for the Synthesis of Azides from Primary Amines. *Adv. Synth. Catal.* **2010**, *352*, 2515-2520. b) $[IrCl_2Cp^*]_2$: Ball, R.G.; Graham, W.A.G.; Heinekey, D.M., Synthesis and structure of dicarbonylbis(.eta.-pentamethylcyclopentadienyl)diiridium. *Inorg. Chem.*, **1990**, *29*, 2023-2025.
8. Zarei, M., An Easy and Convenient Synthesis of β-Lactams via a One-Pot Staudinger Reaction with 4-(4,6-Dimethoxy-1,3,5-triazin-2-yl)-4-methylmorpholinium Chloride Starting from Substituted Carboxylic Acids. *Lett. Org. Chem.* **2015**, *12,* 44-49.
9. Sahu, A.; Sahu, P.; Agrawal, R., Synthesis, Pharmacological and Toxicological Screening of Penicillin–Triazole Conjugates (PNTCs). *ACS Omega* **2019**, *4*, 17230-17235.
10. Chen, P. C.; Wharton, R. E.; Patel, P. A.; Oyelere, A. K., Direct diazo-transfer reaction on β-lactam: Synthesis and preliminary biological activities of 6-triazolylpenicillanic acids. *Bioorg. Med. Chem.* **2007**, *15*, 7288-7300.
11. Meng, G.; Guo, T.; Ma, T.; Zhang, J.; Shen, Y.; Sharpless, K. B.; Dong, J., Modular click chemistry libraries for functional screens using a diazotizing reagent. *Nature* **2019**, *574*, 86-89.
12. *SADABS-2016/2*: Krause, L.; Herbst-Irmer, R.; Sheldrick, G. M.; Stalke, D., Comparison of silver and molybdenum microfocus X-ray sources for single-crystal structure determination. *J. Appl. Crystallogr.* **2015**, *48*, 3.
13. *SIR-97*: Altomare, A.; Burla, M. C.; Camalli, M.; Cascarano, G. L.; Giacovazzo, C.; Guagliardi, A.; Moliterni, A. G. C.; Polidori, G.; Spagna, R., SIR97: a new tool for crystal structure determination and refinement. *J. Appl. Crystallogr.* **1999**, *32*, 115-119.
14. *SHELXL-2014*: Sheldrick, G. M., Crystal structure refinement with SHELXL. *Acta Cryst.* **2008**, *A64*, 112.
15. *WINGX*, version 2021.3: Farrugia, L., WinGX and ORTEP for Windows: an update. *J. Appl. Crystallogr.* **2012**, *45*, 849–854.
16. *MERCURY*, version 2022.2.0 (build 353591): Cambridge Crystallographic Data Centre, Cambridge, UK, **2022**.

17. Zhao, Y.; Truhlar, D. G., The M06 suite of density functionals for main group thermochemistry, thermochemical kinetics, noncovalent interactions, excited states, and transition elements: two new functionals and systematic testing of four M06-class functionals and 12 other functionals. *Theor. Chem. Acc.* **2008**, *120*, 215-241.

18. (a) Zhao, Y.; Truhlar, D. G., Density Functionals with Broad Applicability in Chemistry. *Res Acc. Chem*. **2008**, *41*, 157-167. (b) Zhao, Y.; Truhlar, D. G., Applications and validations of the Minnesota density functionals. *Chem. Phys. Lett.* **2011**, *502*, 1-13.

19. Gaussian 16, Revision C.01, Frisch, M. J.; Trucks, G. W.; Schlegel, H. B.; Scuseria, G. E.; Robb, M. A.; Cheeseman, J. R.; Scalmani, G.; Barone, V.; Petersson, G. A.; Nakatsuji, H.; Li, X.; Caricato, M.; Marenich, A. V.; Bloino, J.; Janesko, B. G.; Gomperts, R.; Mennucci, B.; Hratchian, H. P.; Ortiz, J. V.; Izmaylov, A. F.; Sonnenberg, J. L.; Williams-Young, D.; Ding, F.; Lipparini, F.; Egidi, F.; Goings, J.; Peng, B.; Petrone, A.; Henderson, T.; Ranasinghe, D.; Zakrzewski, V. G.; Gao, J.; Rega, N.; Zheng, G.; Liang, W.; Hada, M.; Ehara, M.; Toyota, K.; Fukuda, R.; Hasegawa, J.; Ishida, M.; Nakajima, T.; Honda, Y.; Kitao, O.; Nakai, H.; Vreven, T.; Throssell, K.; Montgomery, J. A., Jr.; Peralta, J. E.; Ogliaro, F.; Bearpark, M. J.; Heyd, J. J.; Brothers, E. N.; Kudin, K. N.; Staroverov, V. N.; Keith, T. A.; Kobayashi, R.; Normand, J.; Raghavachari, K.; Rendell, A. P.; Burant, J. C.; Iyengar, S. S.; Tomasi, J.; Cossi, M.; Millam, J. M.; Klene, M.; Adamo, C.; Cammi, R.; Ochterski, J. W.; Martin, R. L.; Morokuma, K.; Farkas, O.; Foresman, J. B.; Fox, D. J. Gaussian, Inc., Wallingford CT, 2016.

20. Andrae, U.; Häußermann, M.; Dolg, H.; Stoll, H.; Preuß, H., Energy-adjustedab initio pseudopotentials for the second and third row transition elements. *Theor. Chim. Acta* **1990**, *77,* 123-141.

21 Ehlers, A. W.; Böhme, M.; Dapprich, S.; Gobbi, A.; Höllwarth, A.; Jonas, V.; Köhler, K. F.; Stegmann, R.; Veldkamp, A.; Frenking, G., A set of f-polarization functions for pseudo-potential basis sets of the transition metals Sc Cu, Y Ag and La Au. *Chem. Phys. Lett.* **1993**, *208*, 111-114.

22 a) Hehre, W. J.; Ditchfield, R. J.; Pople, A., Self—Consistent Molecular Orbital Methods. XII. Further Extensions of Gaussian—Type Basis Sets for Use in

Molecular Orbital Studies of Organic Molecules. *J. Chem. Phys*. **1972**, *56*, 2257-2261. (b) Francl, M. M.; Pietro, W. J.; Hehre, W. J.; Binkley, J. S.; Gordon, M. S.; DeFrees, D. J. J.; Pople, A., Self-consistent molecular orbital methods. XXIII. A polarization-type basis set for second-row elements. *J. Chem. Phys.* **1982**, *77*, 3654-3665.

23. Marenich, A. V.; Cramer, C. J.; Truhlar, D. G., Universal solvation model based on solute electron density and on a continuum model of the solvent defined by the bulk dielectric constant and atomic surface tensions. *J. Phys. Chem. B* **2009**, *113*, 6378-6396.

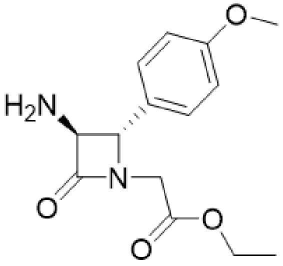

***trans*-8c**

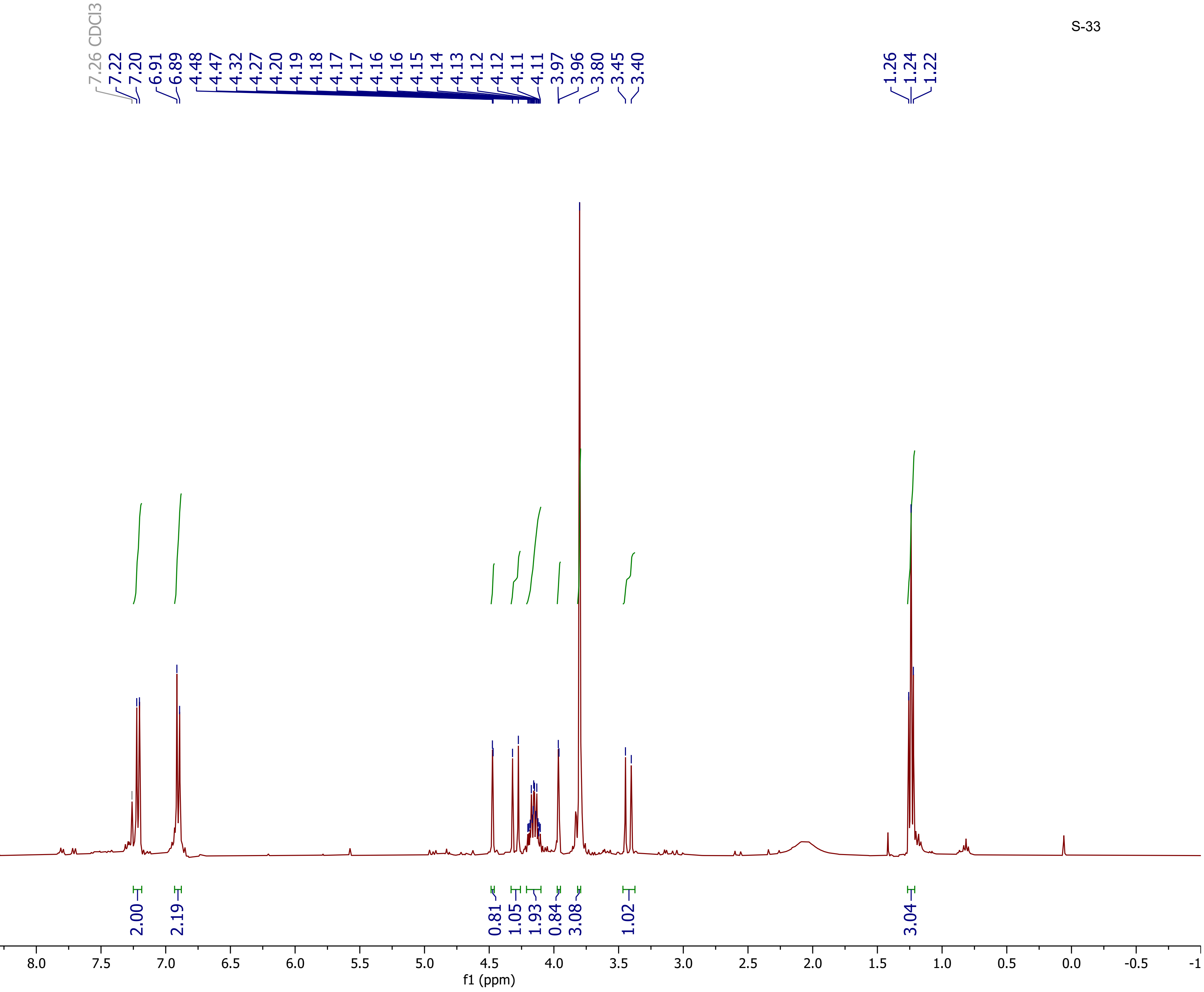
7.26 CDCl3
7.22
7.20
6.91
6.89
4.48
4.47
4.32
4.27
4.20
4.19
4.18
4.17
4.17
4.16
4.16
4.15
4.14
4.13
4.12
4.12
4.11
4.11
3.97
3.96
3.80
3.45
3.40
1.26
1.24
1.22
2.00
2.19
0.81
1.05
1.93
0.84
3.08
1.02
3.04
9.5 9.0 8.5 8.0 7.5 7.0 6.5 6.0 5.5 5.0 4.5 4.0 3.5 3.0 2.5 2.0 1.5 1.0 0.5 0.0 -0.5 -1
f1 (ppm)

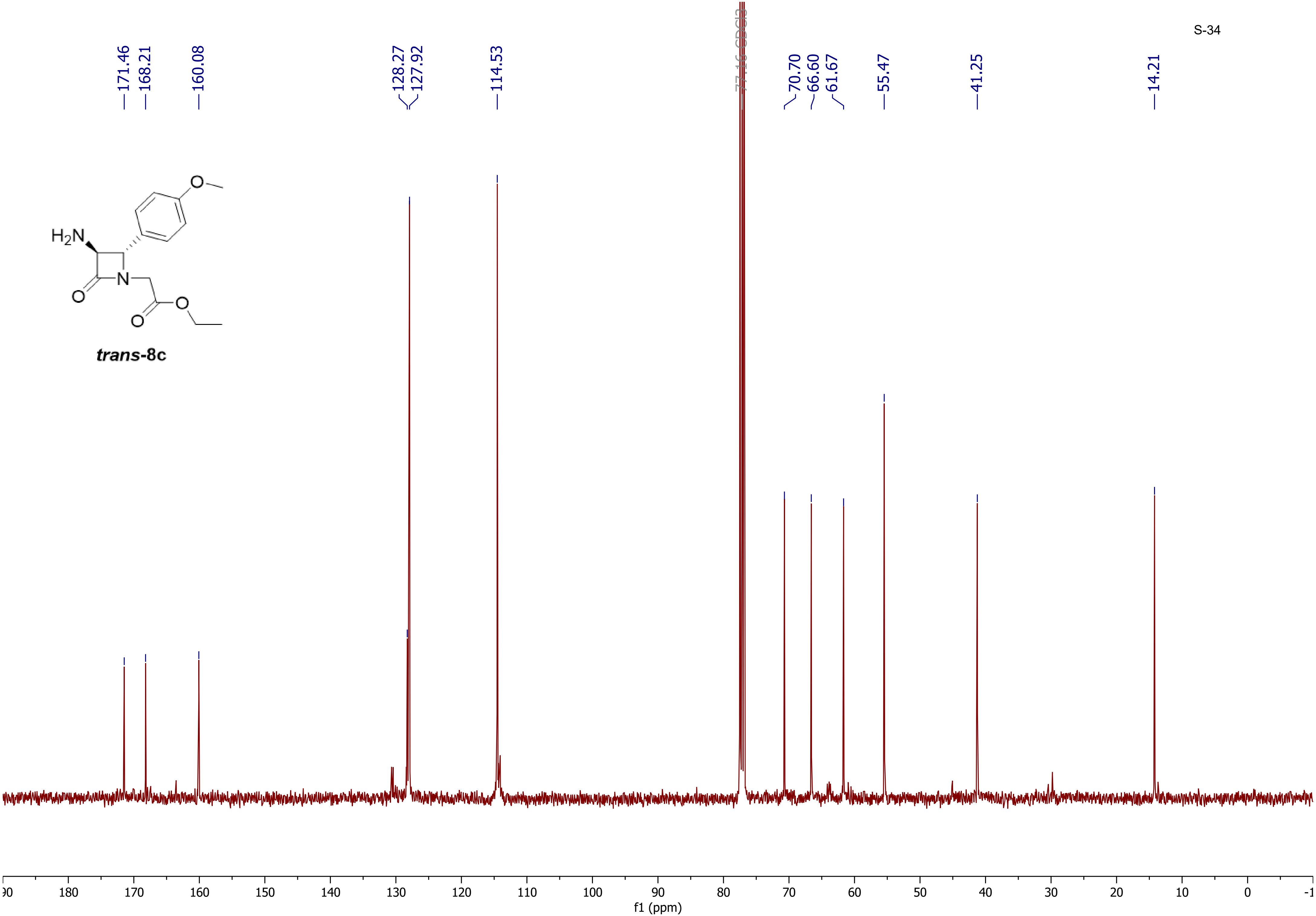
171.46
168.21
160.08
128.27
127.92
114.53
77.16 CDCl3
70.70
66.60
61.67
55.47
41.25
14.21
H2N
N
O
O
O
O
trans-8c
180
170
160
150
140
130
120
110
100
90
80
70
60
50
40
30
20
10
0
f1 (ppm)

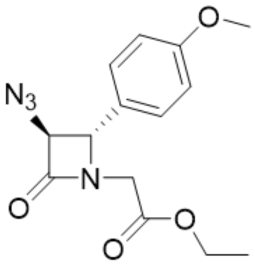
N3
O
N
O
O
O

***trans*-7c**

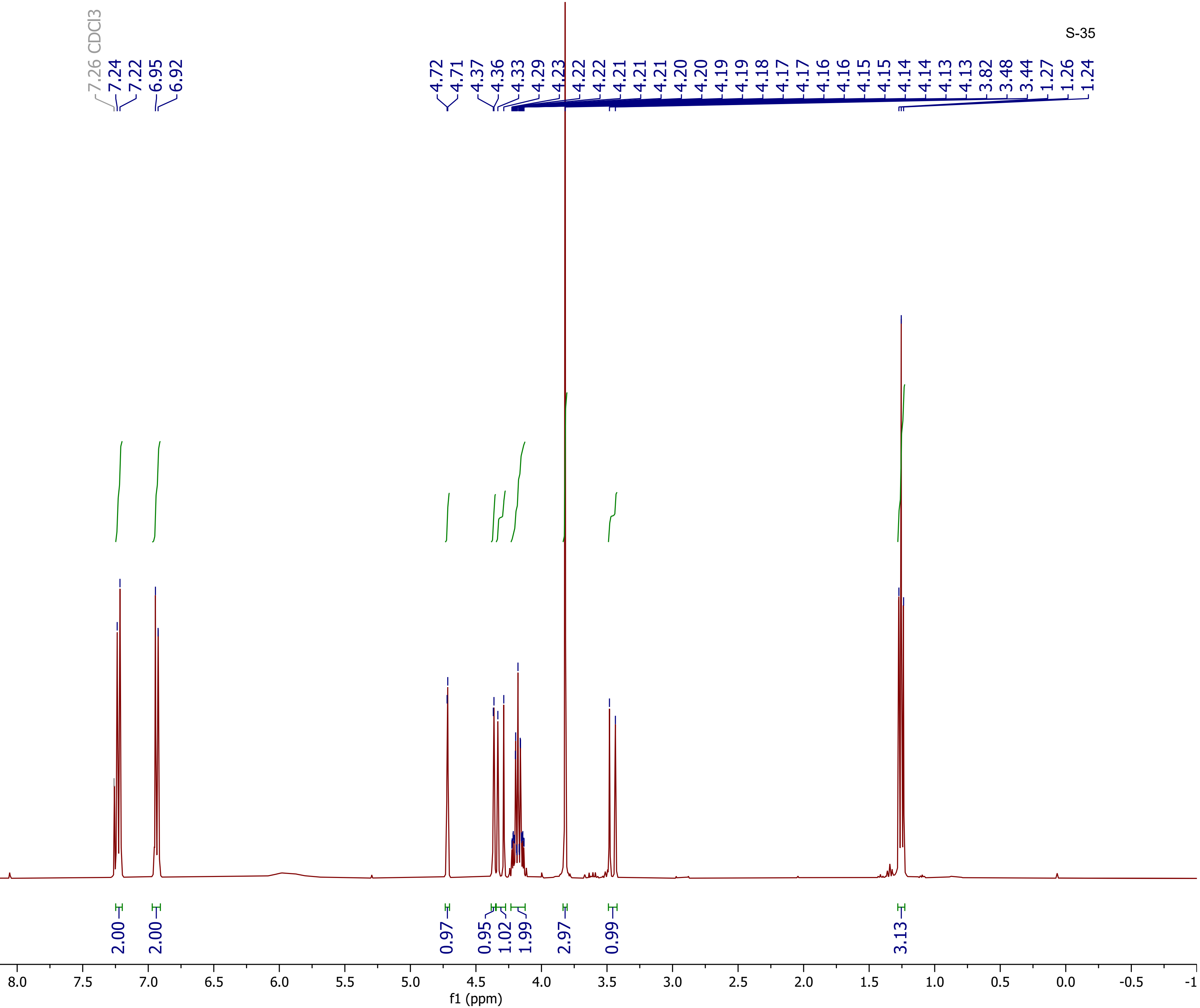
7.26 CDCl3
7.24
7.22
6.95
6.92
4.72
4.71
4.37
4.36
4.33
4.29
4.23
4.22
4.22
4.21
4.21
4.21
4.20
4.20
4.19
4.19
4.18
4.17
4.17
4.16
4.16
4.15
4.15
4.14
4.14
4.13
4.13
3.82
3.48
3.44
1.27
1.26
1.24
2.00
2.00
0.97
0.95
1.02
1.99
2.97
0.99
3.13
9.5
9.0
8.5
8.0
7.5
7.0
6.5
6.0
5.5
5.0
4.5
4.0
3.5
3.0
2.5
2.0
1.5
1.0
0.5
0.0
-0.5
f1 (ppm)

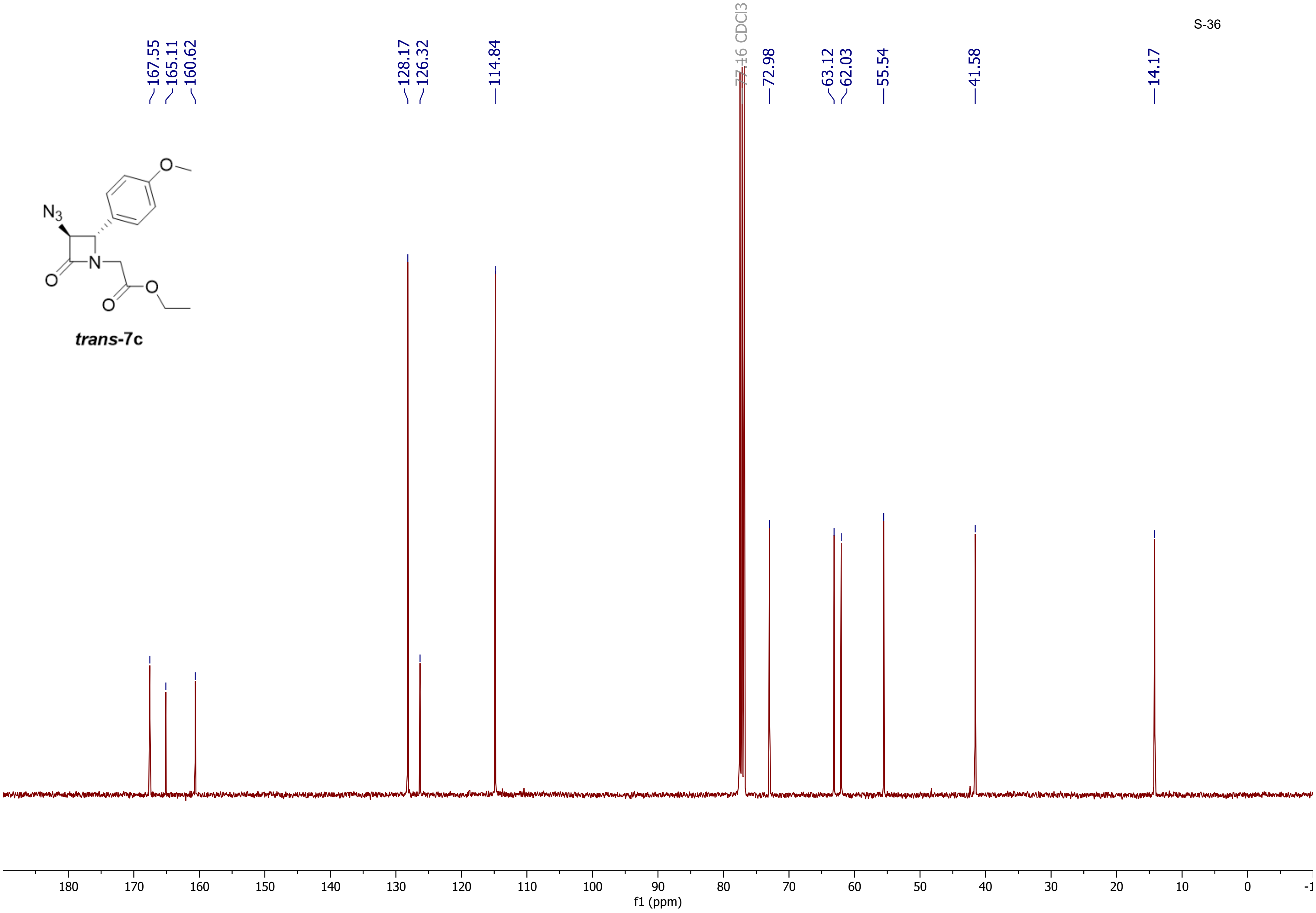
167.55
165.11
160.62
128.17
126.32
114.84
77.16 CDCl3
72.98
63.12
62.03
55.54
41.58
14.17
N3
O
N
O
O
O
trans-7c
180 170 160 150 140 130 120 110 100 90 80 70 60 50 40 30 20 10 0 -1
f1 (ppm)

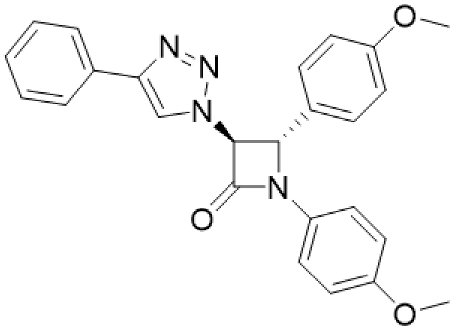

trans-**10a**

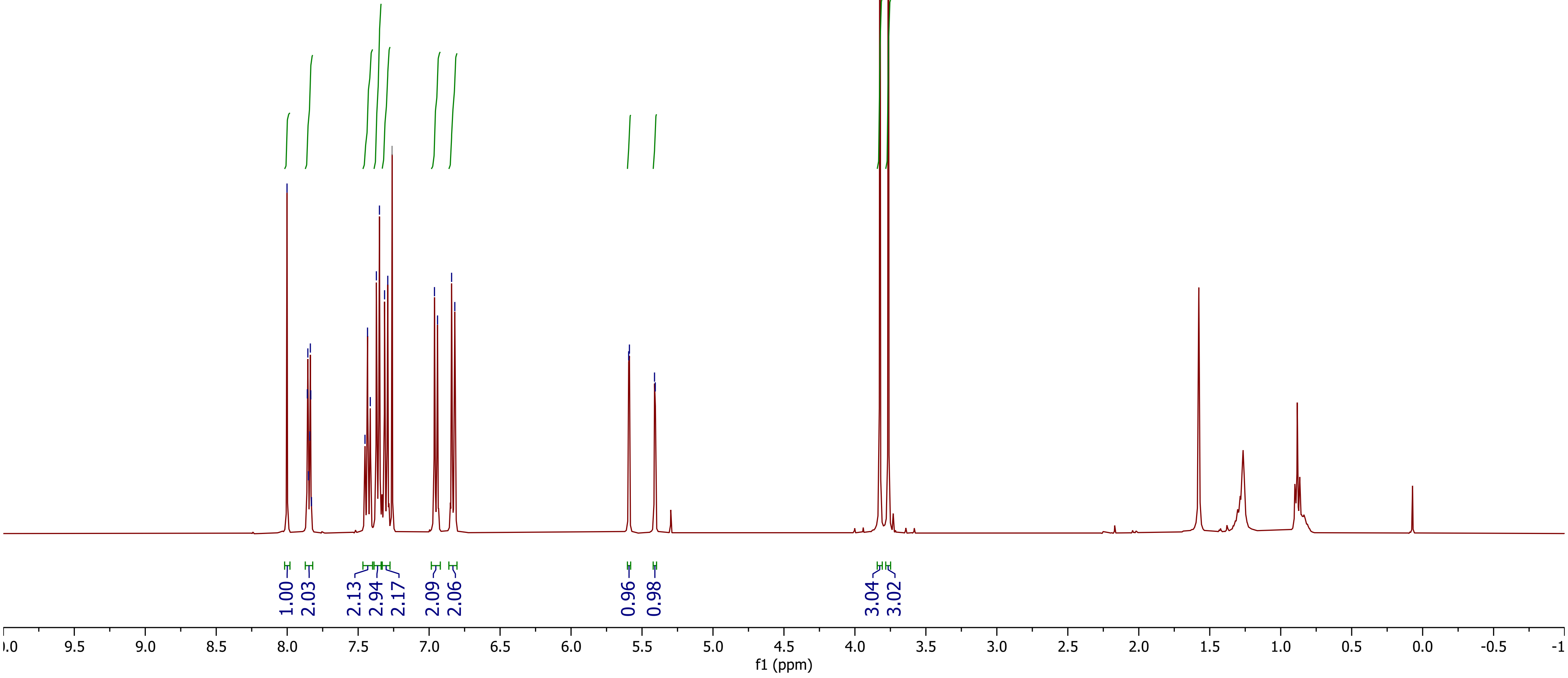
8.00
7.86
7.85
7.85
7.84
7.84
7.83
7.83
7.45
7.43
7.41
7.37
7.35
7.31
7.29
7.26 CDCl3
6.96
6.94
6.84
6.82
5.59
5.59
5.41
5.41
3.82
3.76
1.00
2.03
2.13
2.94
2.17
2.09
2.06
0.96
0.98
3.04
3.02
f1 (ppm)

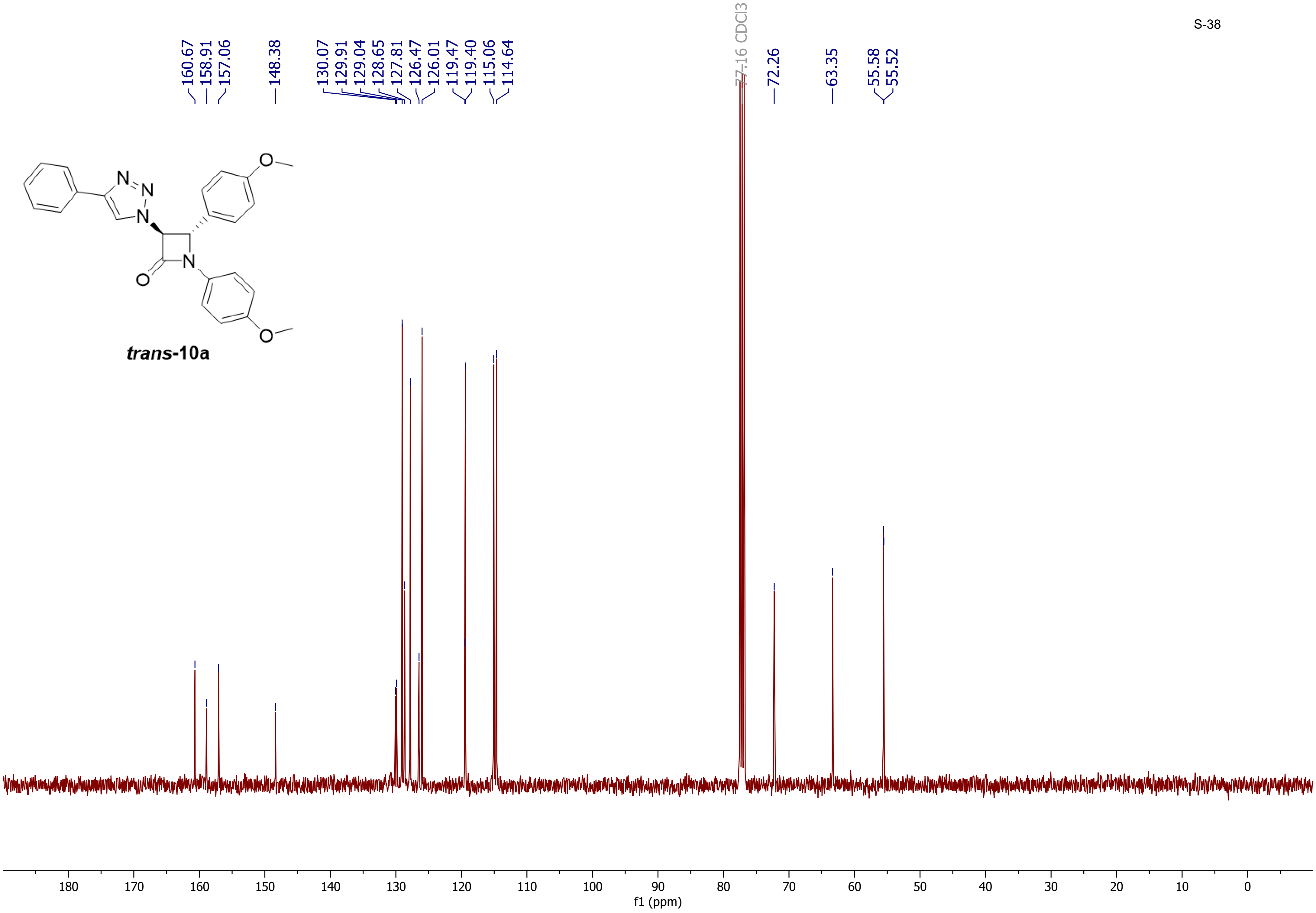

160.67
158.91
157.06
148.38
130.07
129.91
129.04
128.65
127.81
126.47
126.01
119.47
119.40
115.06
114.64
77.16 CDCl3
72.26
63.35
55.58
55.52
O
N
N
N
N
O
O
trans-10a
180
170
160
150
140
130
120
110
100
90
80
70
60
50
40
30
20
10
0
f1 (ppm)

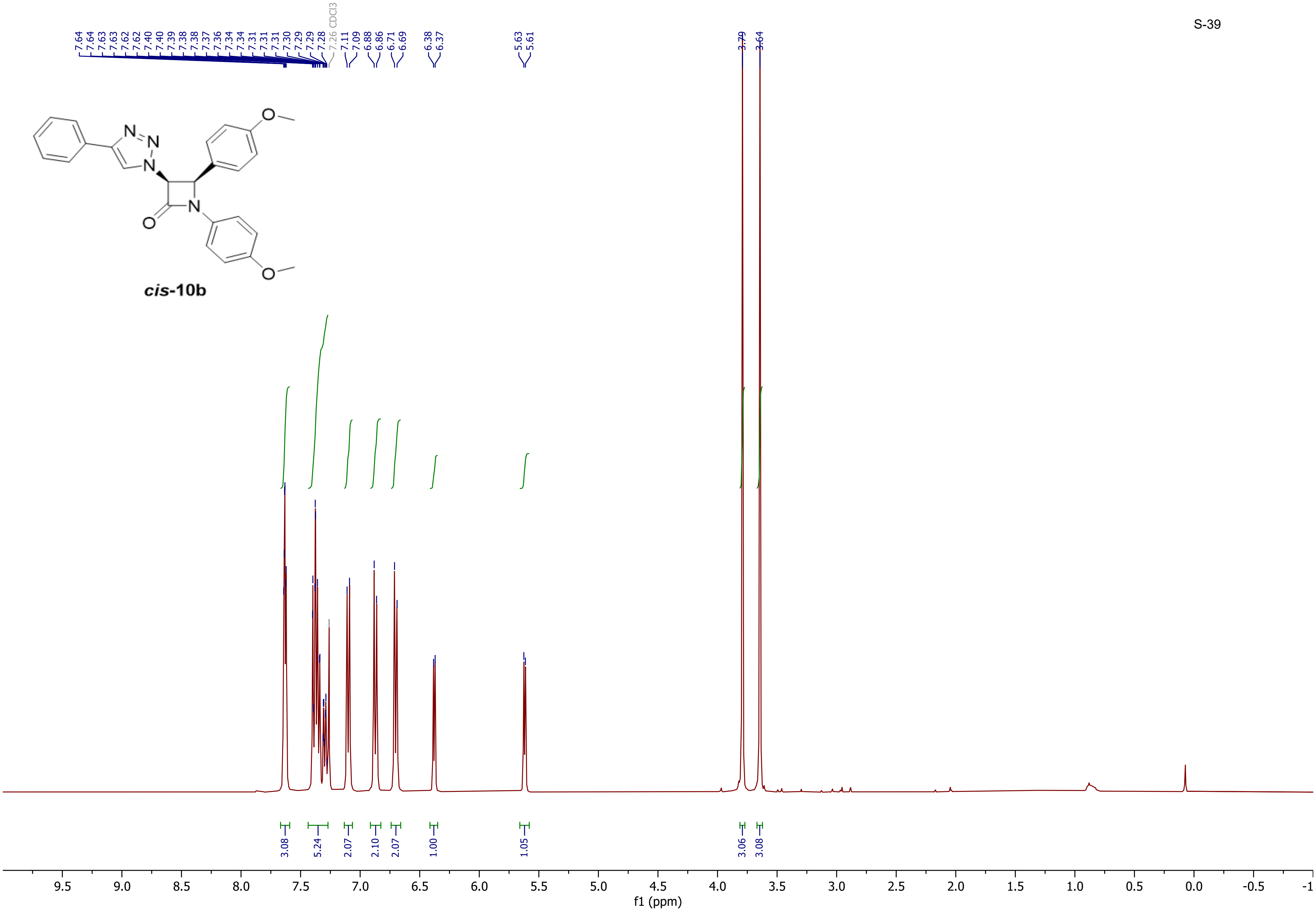
7.64
7.64
7.63
7.63
7.62
7.62
7.40
7.40
7.39
7.38
7.38
7.37
7.36
7.34
7.34
7.31
7.31
7.30
7.29
7.29
7.28
7.26 CDCl3
7.11
7.09
6.88
6.86
6.71
6.69
6.38
6.37
5.63
5.61
3.79
3.64
cis-10b
3.08
5.24
2.07
2.10
2.07
1.00
1.05
3.06
3.08
9.5
9.0
8.5
8.0
7.5
7.0
6.5
6.0
5.5
5.0
4.5
4.0
3.5
3.0
2.5
2.0
1.5
1.0
0.5
0.0
-0.5
-1
f1 (ppm)

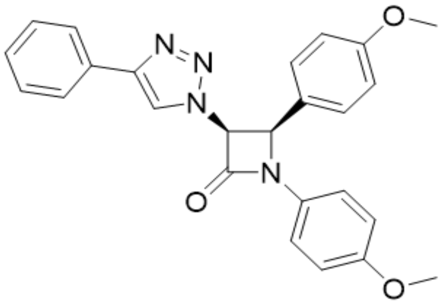
N
N
N
O
N
O
O

***cis*-10b**

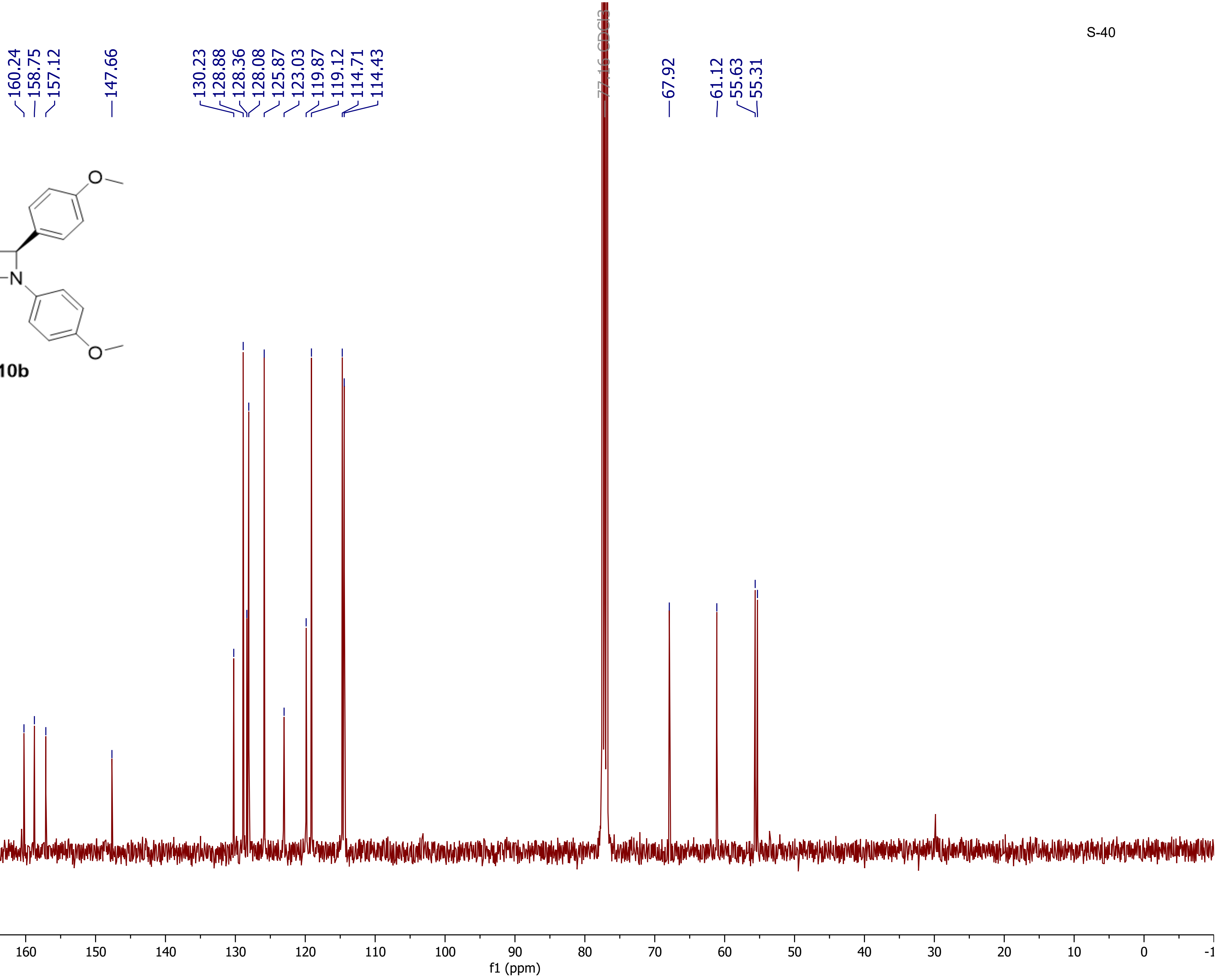
160.24
158.75
157.12
147.66
130.23
128.88
128.36
128.08
125.87
123.03
119.87
119.12
114.71
114.43
77.16 CDCl3
67.92
61.12
55.63
55.31
f1 (ppm)

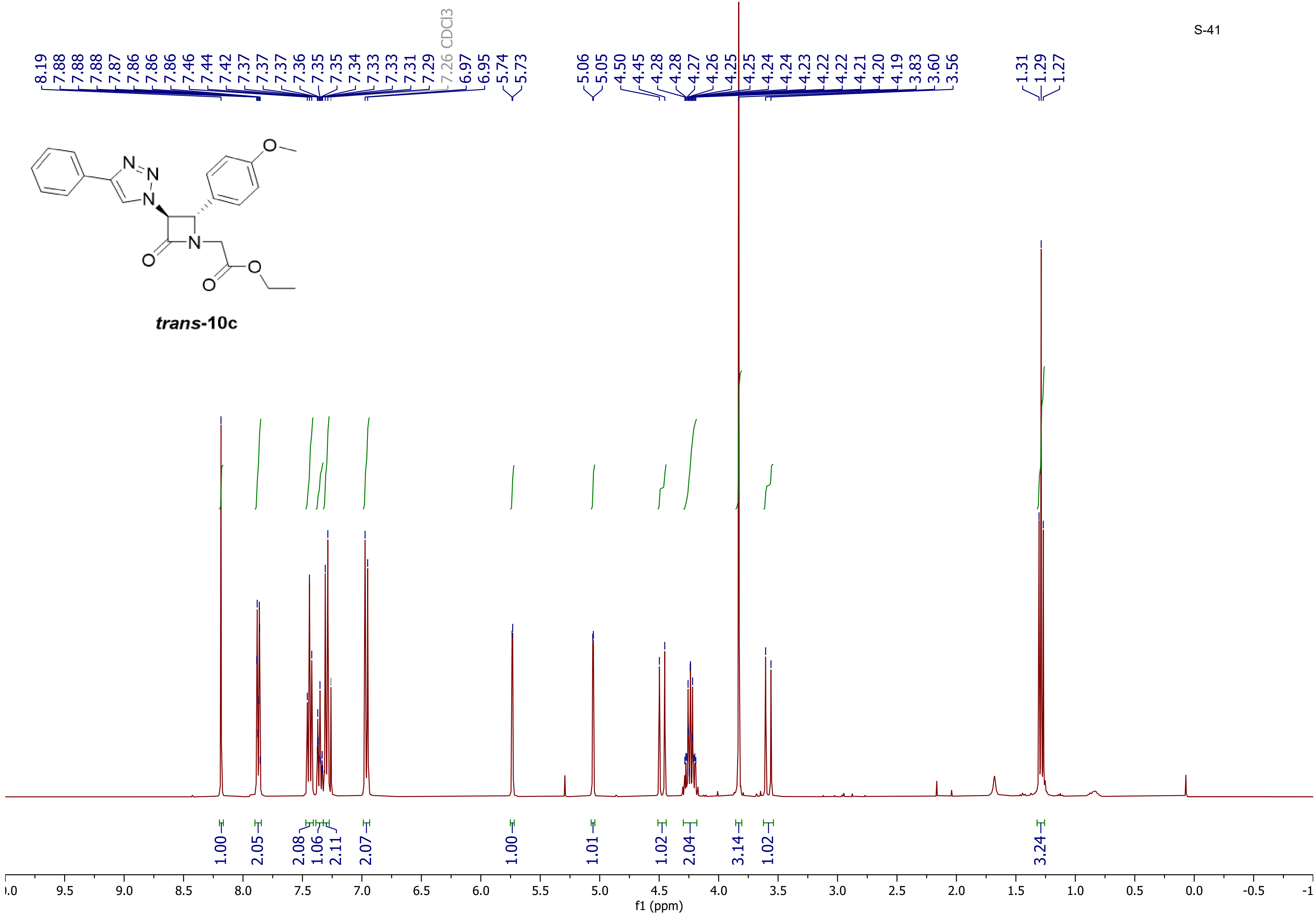
8.19
7.88
7.88
7.88
7.87
7.86
7.86
7.86
7.46
7.44
7.42
7.37
7.37
7.37
7.36
7.35
7.35
7.34
7.33
7.33
7.31
7.29
7.26 CDCl3
6.97
6.95
5.74
5.73
5.06
5.05
4.50
4.45
4.28
4.28
4.27
4.26
4.25
4.25
4.24
4.24
4.23
4.22
4.22
4.21
4.20
4.19
3.83
3.60
3.56
1.31
1.29
1.27
trans-10c
1.00
2.05
2.08
1.06
2.11
2.07
1.00
1.01
1.02
2.04
3.14
1.02
3.24
f1 (ppm)

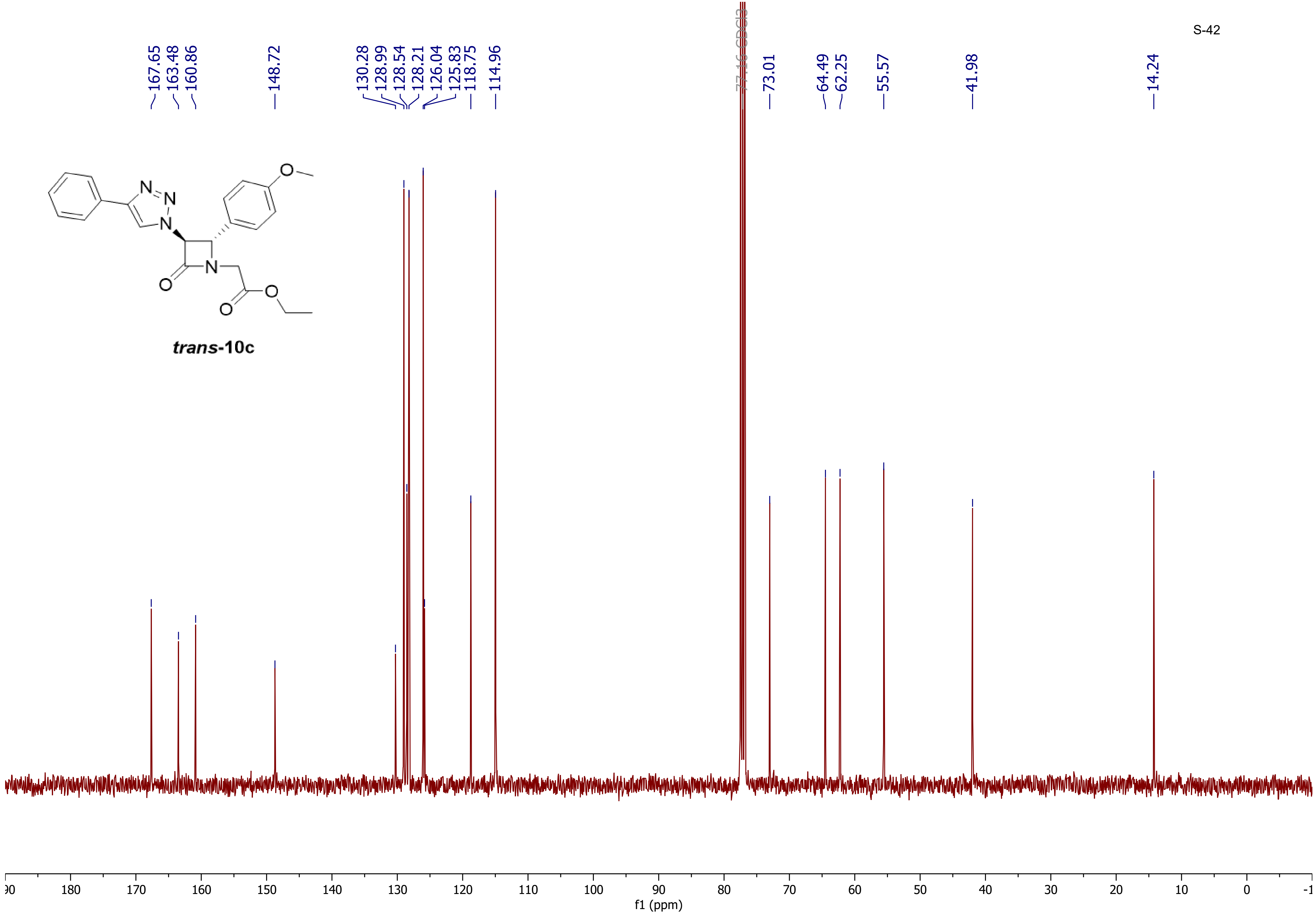
167.65
163.48
160.86
148.72
130.28
128.99
128.54
128.21
126.04
125.83
118.75
114.96
77.16 CDCl3
73.01
64.49
62.25
55.57
41.98
14.24
trans-10c
190
180
170
160
150
140
130
120
110
100
90
80
70
60
50
40
30
20
10
0
-10
f1 (ppm)

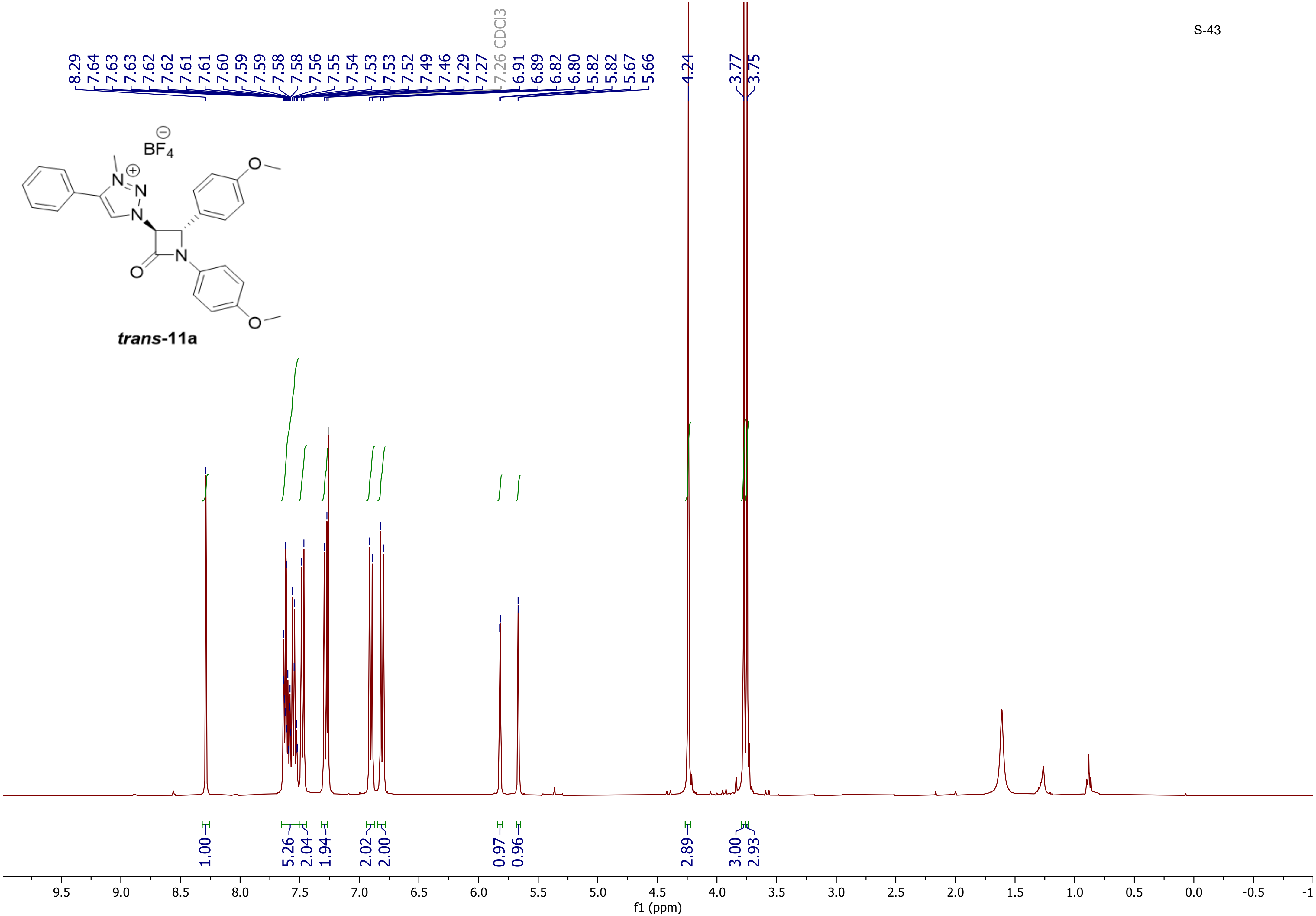
BF4
N
N
N
O
N
O
O
8.29
7.64
7.63
7.63
7.62
7.62
7.61
7.61
7.60
7.59
7.59
7.58
7.58
7.56
7.55
7.54
7.53
7.53
7.52
7.49
7.46
7.29
7.27
7.26 CDCl3
6.91
6.89
6.82
6.80
5.82
5.82
5.67
5.66
4.24
3.77
3.75
1.00
5.26
2.04
1.94
2.02
2.00
0.97
0.96
2.89
3.00
2.93
9.5
9.0
8.5
8.0
7.5
7.0
6.5
6.0
5.5
5.0
4.5
4.0
3.5
3.0
2.5
2.0
1.5
1.0
0.5
0.0
-0.5
-1
f1 (ppm)

***trans*-11a**

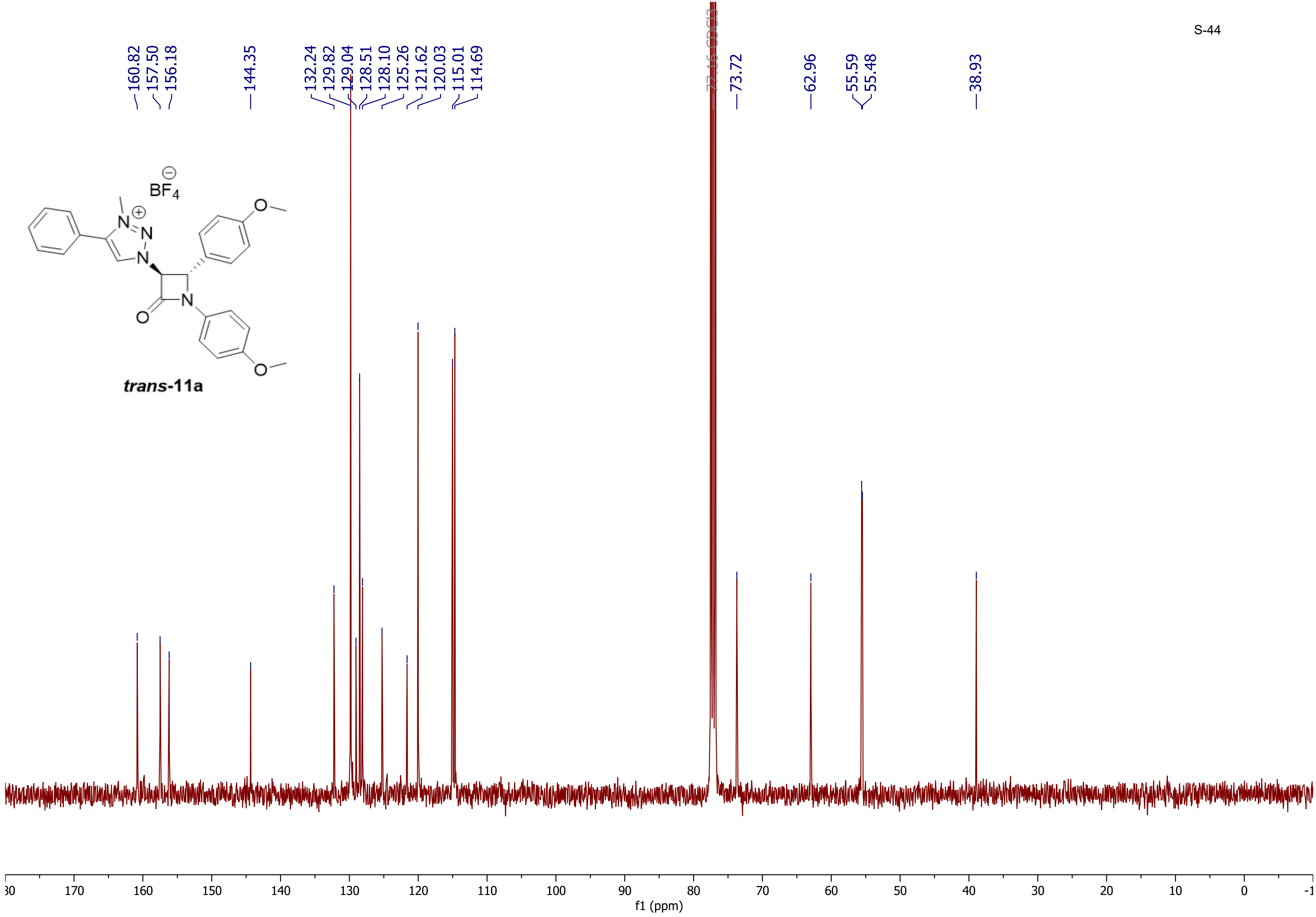
160.82
157.50
156.18
144.35
132.24
129.82
129.04
128.51
128.10
125.26
121.62
120.03
115.01
114.69
77.16 CDCl3
73.72
62.96
55.59
55.48
38.93
BF4
trans-11a
f1 (ppm)

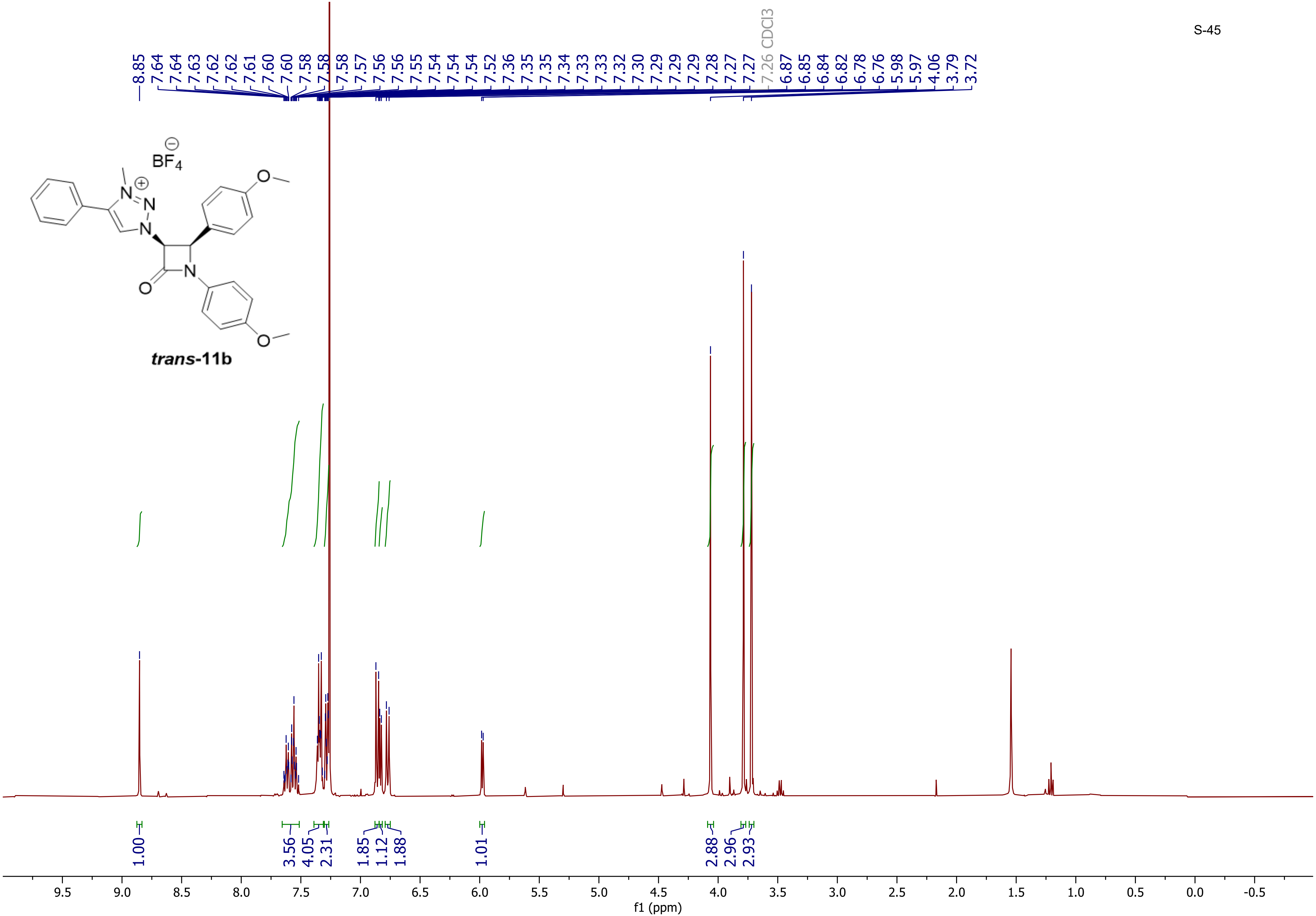
8.85 7.64 7.64 7.63 7.62 7.62 7.61 7.60 7.60 7.58 7.58 7.58 7.57 7.56 7.56 7.55 7.54 7.54 7.54 7.52 7.36 7.35 7.35 7.34 7.33 7.33 7.32 7.30 7.29 7.29 7.29 7.28 7.27 7.27 7.26 CDCl3 6.87 6.85 6.84 6.82 6.78 6.76 5.98 5.97 4.06 3.79 3.72
BF_4^-
1.00
3.56
4.05
2.31
1.85
1.12
1.88
1.01
2.88
2.96
2.93
9.5 9.0 8.5 8.0 7.5 7.0 6.5 6.0 5.5 5.0 4.5 4.0 3.5 3.0 2.5 2.0 1.5 1.0 0.5 0.0 -0.5
f1 (ppm)

***trans*-11b**

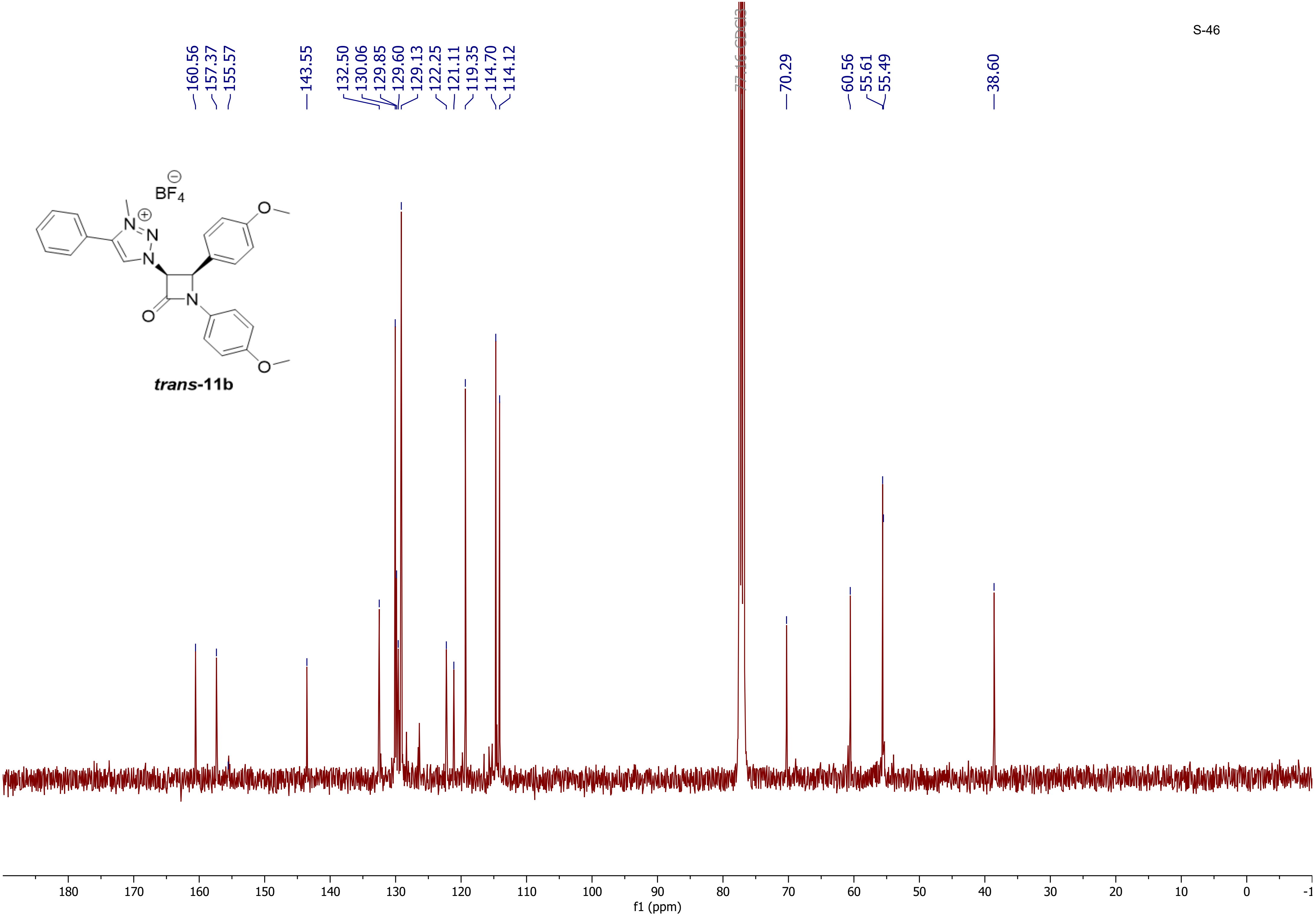

160.56
157.37
155.57
143.55
132.50
130.06
129.85
129.60
129.13
122.25
121.11
119.35
114.70
114.12
77.16 CDCl3
70.29
60.56
55.61
55.49
38.60
BF4
N
N
N
N
O
O
O
trans-11b
180 170 160 150 140 130 120 110 100 90 80 70 60 50 40 30 20 10 0 -1
f1 (ppm)

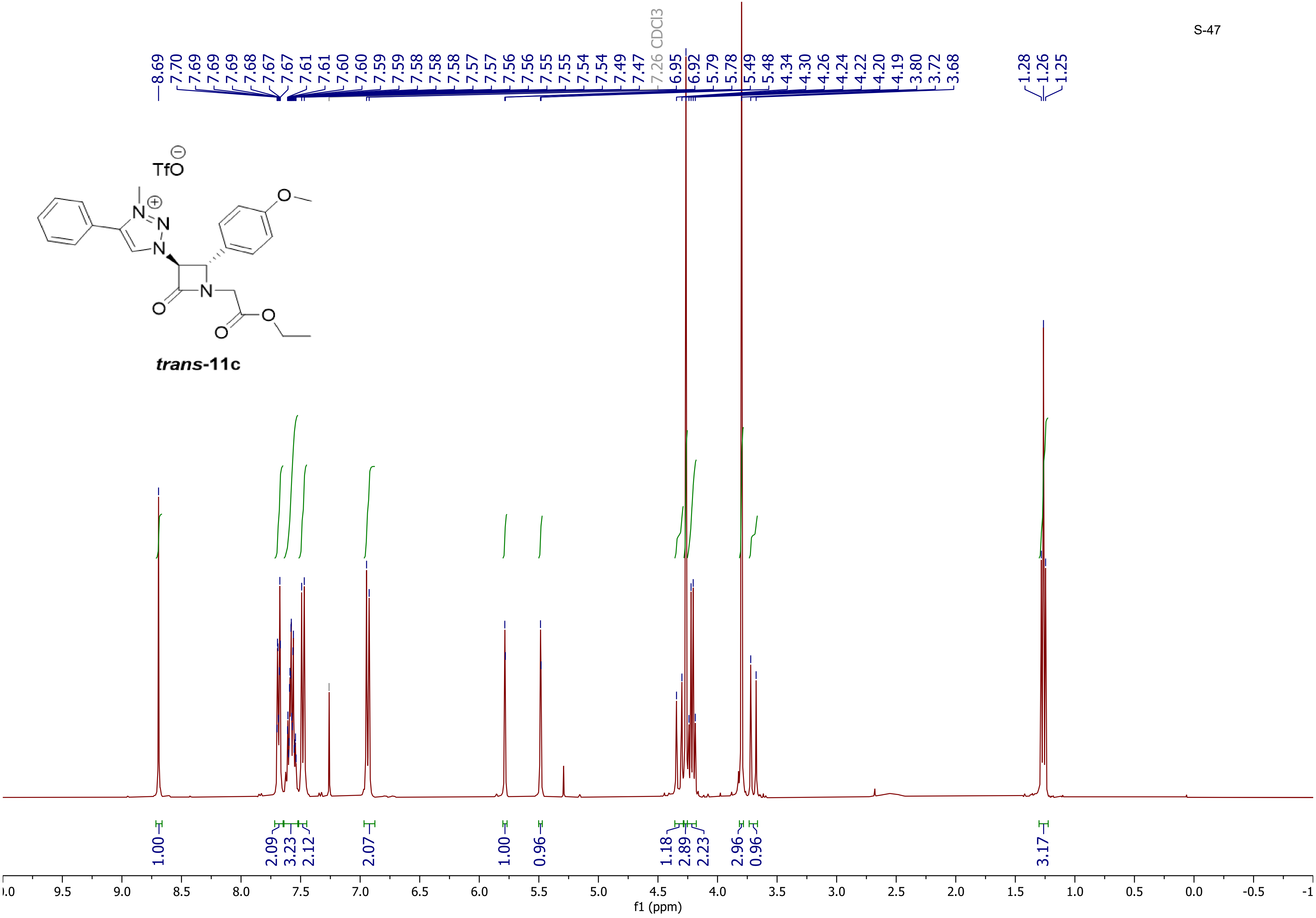
8.69
7.70
7.69
7.69
7.69
7.68
7.67
7.67
7.61
7.61
7.60
7.60
7.59
7.59
7.58
7.58
7.58
7.57
7.57
7.56
7.56
7.55
7.55
7.54
7.54
7.49
7.47
7.26 CDCl3
6.95
6.92
5.79
5.78
5.49
5.48
4.34
4.30
4.26
4.24
4.22
4.20
4.19
3.80
3.72
3.68
1.28
1.26
1.25
TfO
trans-11c
1.00
2.09
3.23
2.12
2.07
1.00
0.96
1.18
2.89
2.23
2.96
0.96
3.17
f1 (ppm)

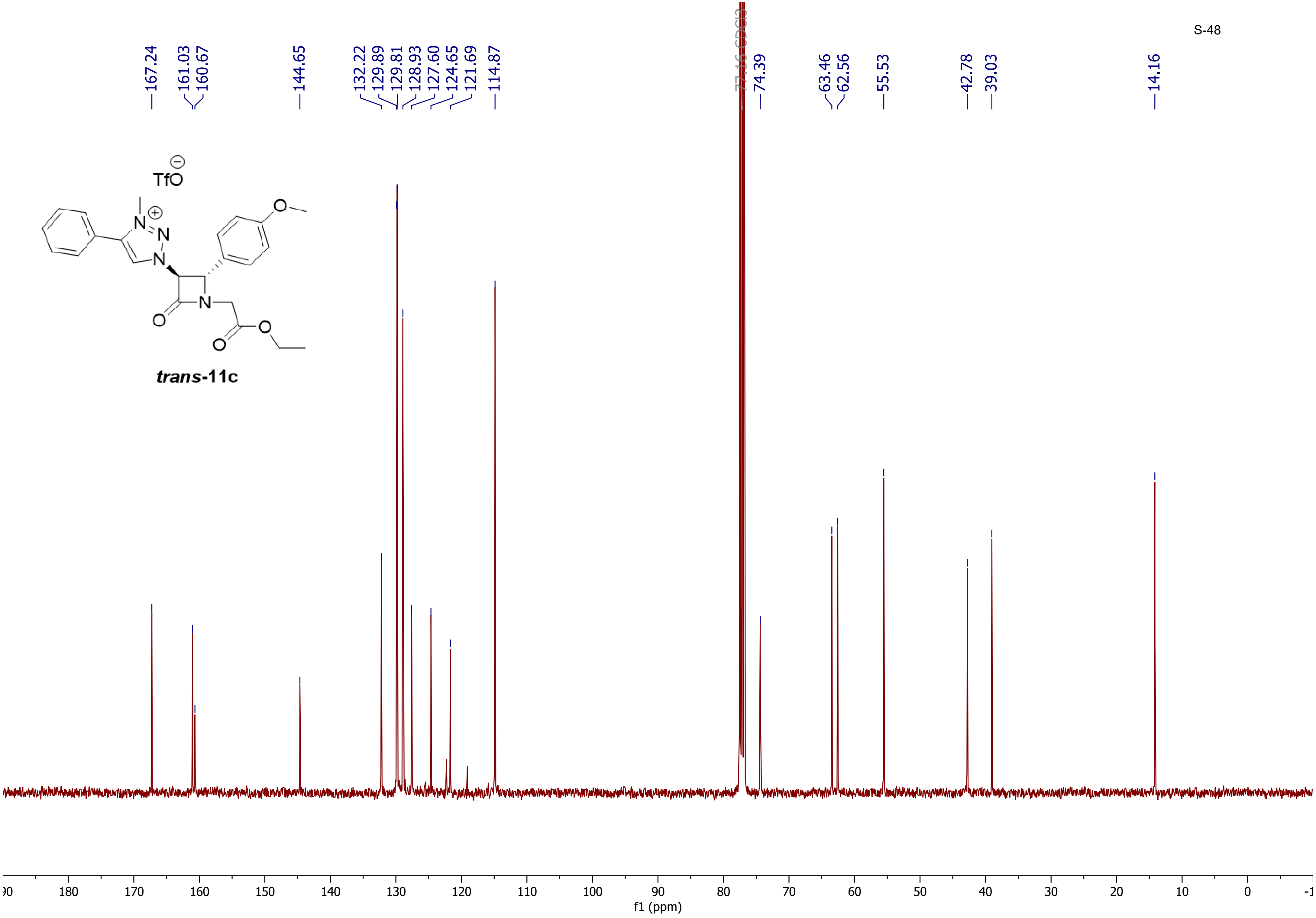

TfO
167.24
161.03
160.67
144.65
132.22
129.89
129.81
128.93
127.60
124.65
121.69
114.87
77.16 CDCl3
74.39
63.46
62.56
55.53
42.78
39.03
14.16
180 170 160 150 140 130 120 110 100 90 80 70 60 50 40 30 20 10 0
f1 (ppm)

***trans*-11c**

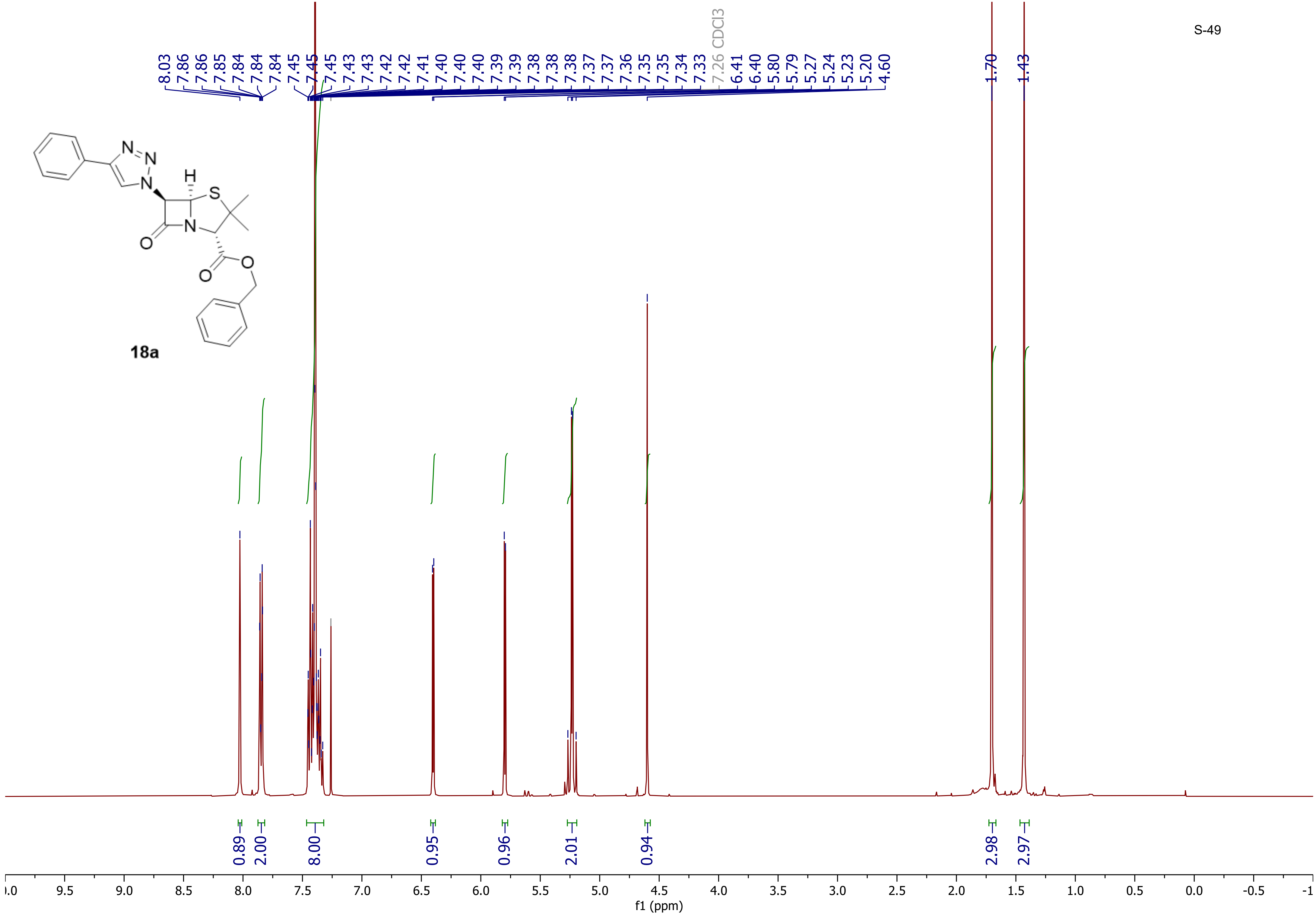
8.03 7.86 7.86 7.85 7.84 7.84 7.84 7.45 7.45 7.45 7.43 7.43 7.42 7.42 7.41 7.40 7.40 7.40 7.39 7.39 7.38 7.38 7.38 7.37 7.37 7.36 7.35 7.35 7.34 7.33 7.26 CDCl3 6.41 6.40 5.80 5.79 5.27 5.24 5.23 5.20 4.60
1.70 1.43
18a
0.89 2.00 8.00 0.95 0.96 2.01 0.94 2.98 2.97
f1 (ppm)

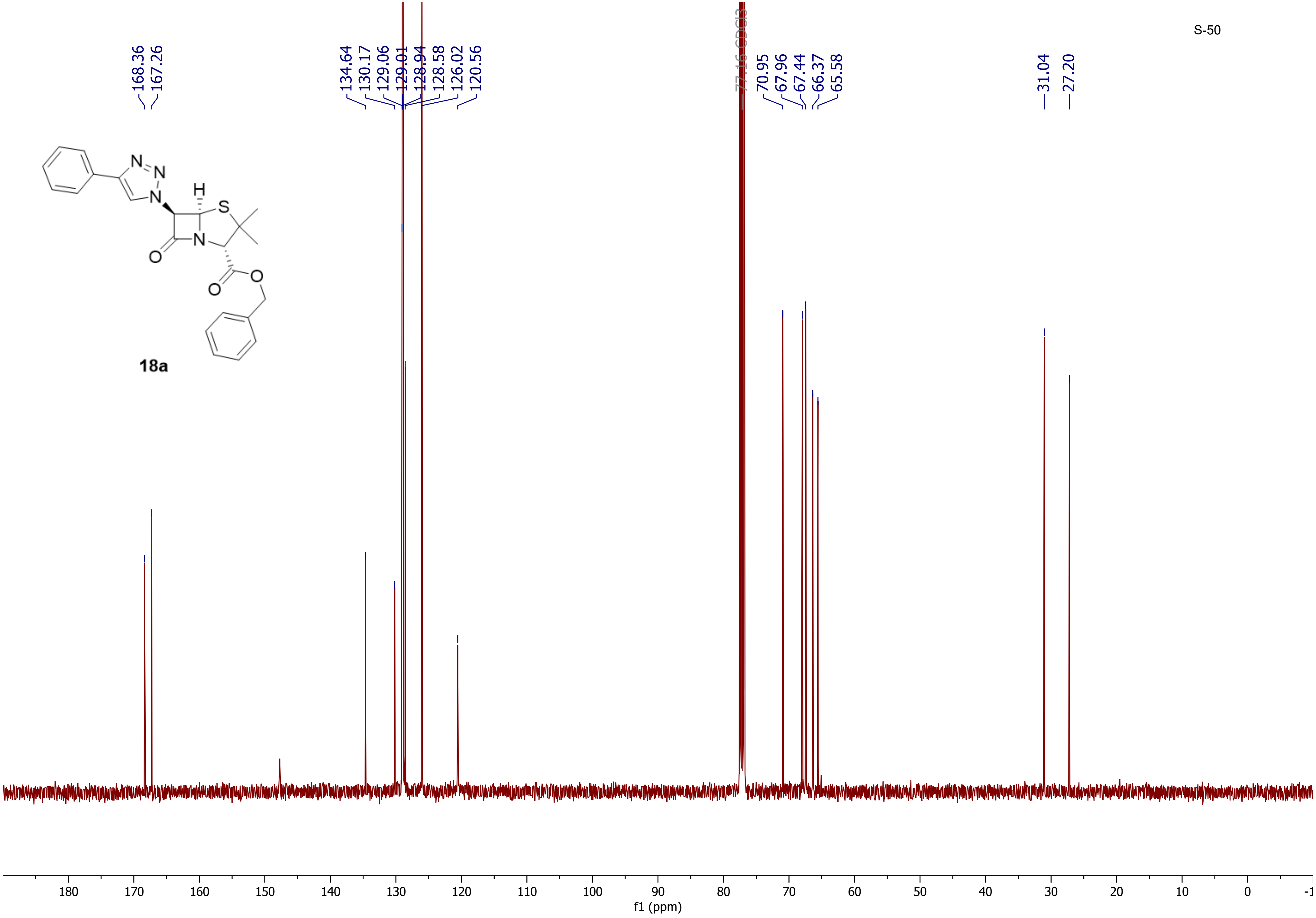
168.36
167.26
134.64
130.17
129.06
129.01
128.94
128.58
126.02
120.56
77.16 CDCl3
70.95
67.96
67.44
66.37
65.58
31.04
27.20
18a
180 170 160 150 140 130 120 110 100 90 80 70 60 50 40 30 20 10 0 -1
f1 (ppm)

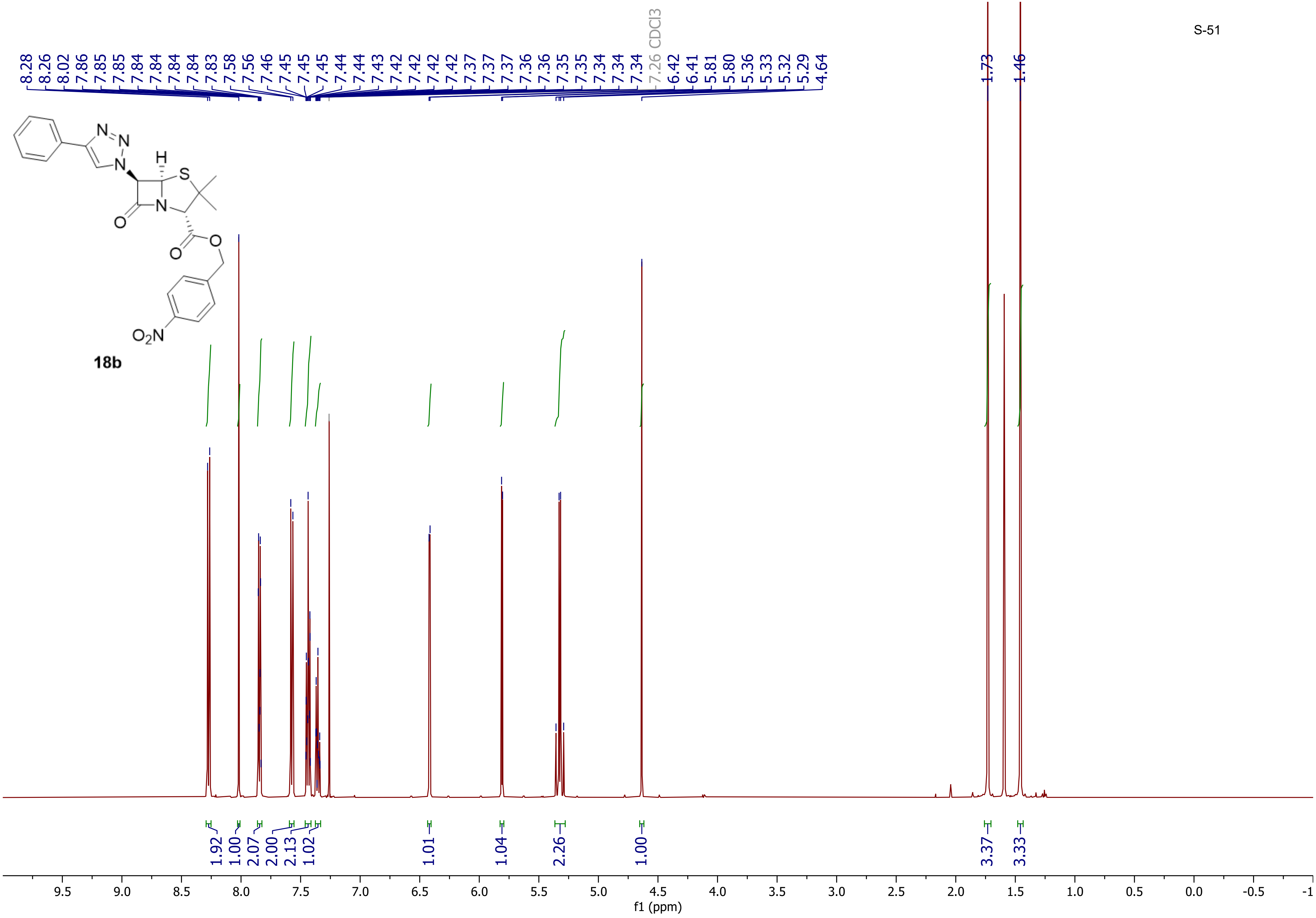
8.28 8.26 8.02 7.86 7.85 7.85 7.84 7.84 7.84 7.84 7.83 7.58 7.56 7.46 7.45 7.45 7.45 7.44 7.44 7.43 7.42 7.42 7.42 7.42 7.37 7.37 7.37 7.36 7.36 7.35 7.35 7.34 7.34 7.34 7.26 CDCl3 6.42 6.41 5.81 5.80 5.36 5.33 5.32 5.29 4.64
1.73
1.46
N
N
N
H
S
N
O
O
O
O_2N
18b
1.92 1.00 2.07 2.00 2.13 1.02
1.01
1.04
2.26
1.00
3.37
3.33
9.5 9.0 8.5 8.0 7.5 7.0 6.5 6.0 5.5 5.0 4.5 4.0 3.5 3.0 2.5 2.0 1.5 1.0 0.5 0.0 -0.5 -1
f1 (ppm)

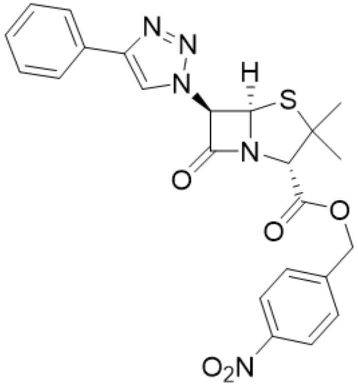

18b

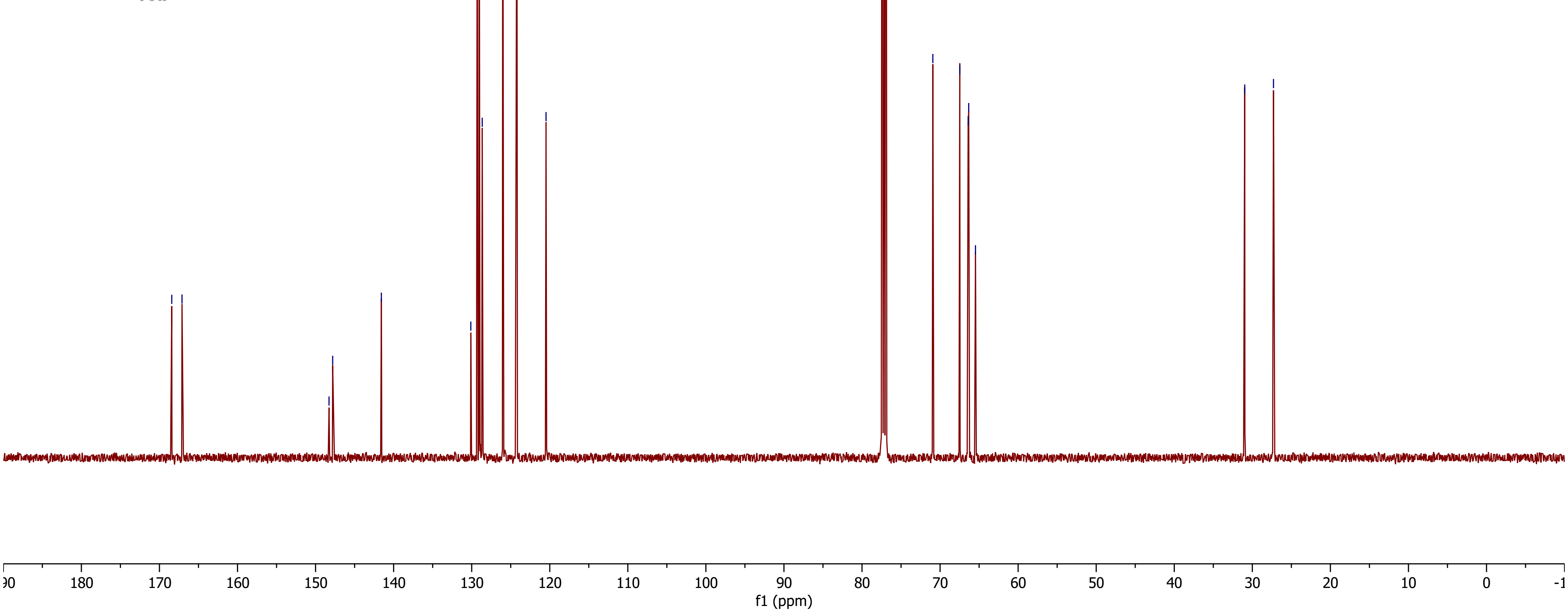
168.42
167.11
148.28
147.81
141.57
130.11
129.29
129.04
128.65
126.03
124.21
120.48
77.16 CDCl3
70.93
67.48
66.40
66.33
65.46
30.97
27.29
90
180
170
160
150
140
130
120
110
100
90
80
70
60
50
40
30
20
10
0
-1
f1 (ppm)

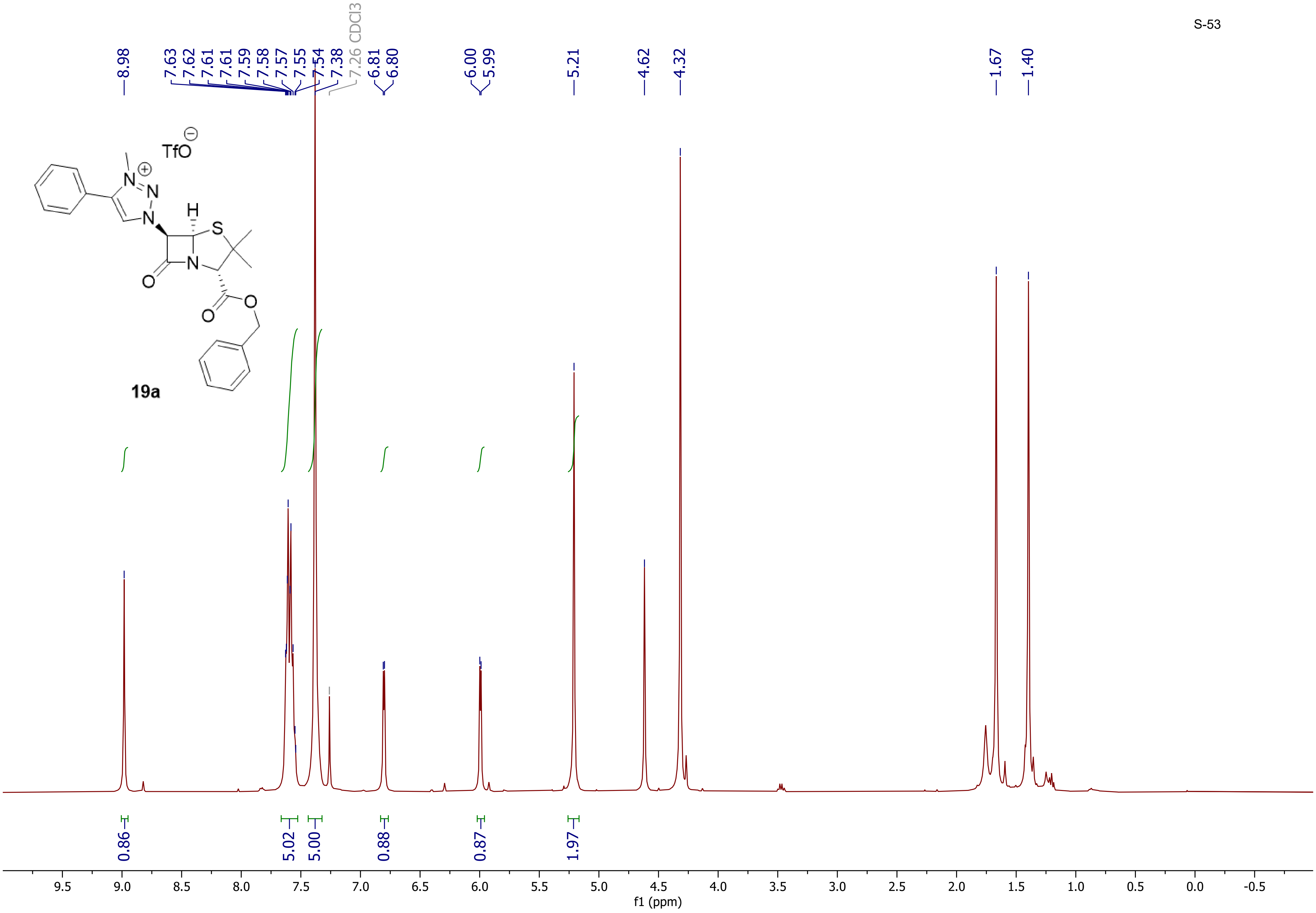
8.98
7.63
7.62
7.61
7.61
7.59
7.58
7.57
7.55
7.54
7.38
7.26 CDCl3
6.81
6.80
6.00
5.99
5.21
4.62
4.32
1.67
1.40
TfO
19a
0.86
5.02
5.00
0.88
0.87
1.97
9.5 9.0 8.5 8.0 7.5 7.0 6.5 6.0 5.5 5.0 4.5 4.0 3.5 3.0 2.5 2.0 1.5 1.0 0.5 0.0 -0.5
f1 (ppm)

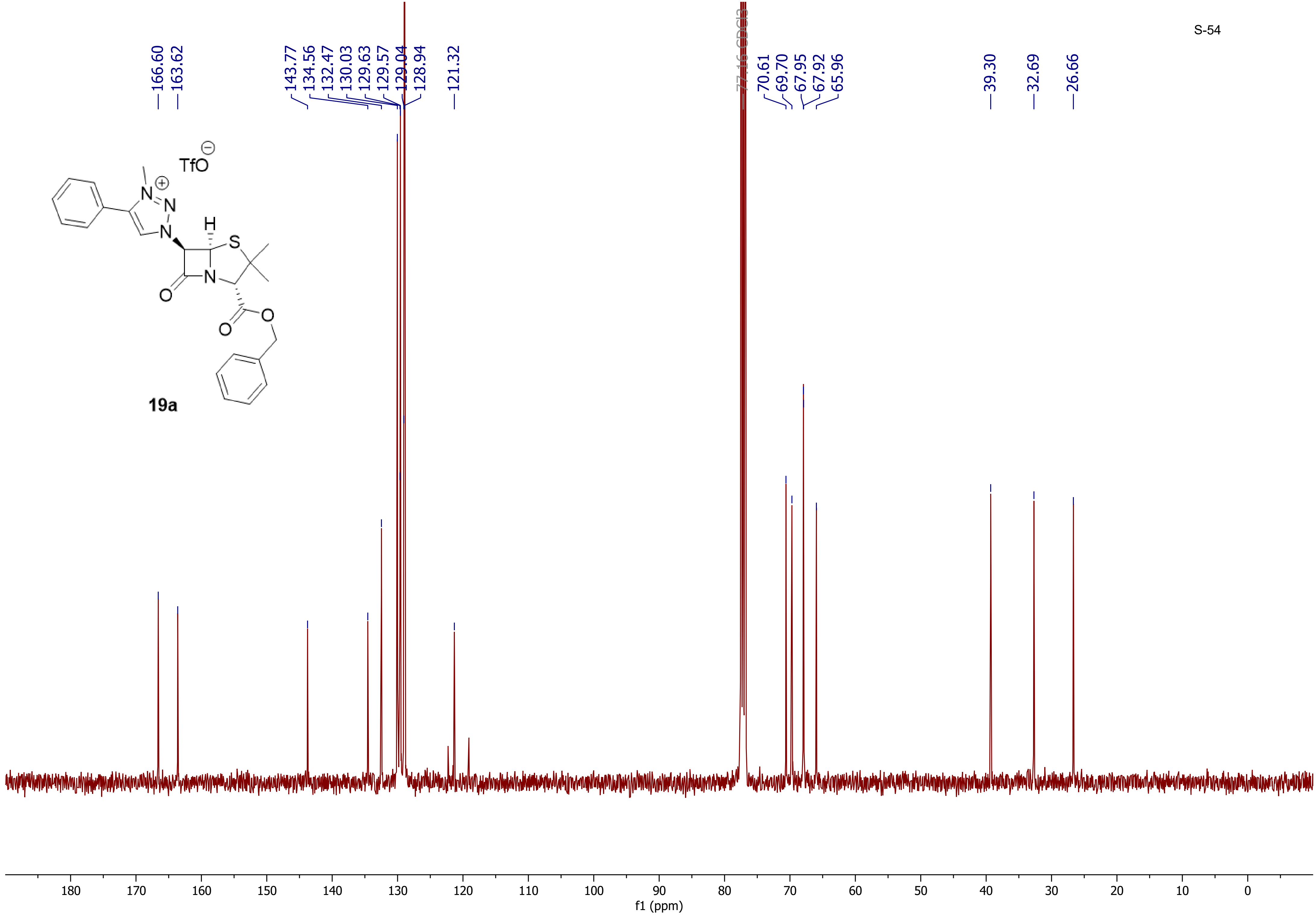
166.60
163.62
143.77
134.56
132.47
130.03
129.63
129.57
129.04
128.94
121.32
77.16 CDCl3
70.61
69.70
67.95
67.92
65.96
39.30
32.69
26.66
TfO
19a
180
170
160
150
140
130
120
110
100
90
80
70
60
50
40
30
20
10
0
f1 (ppm)

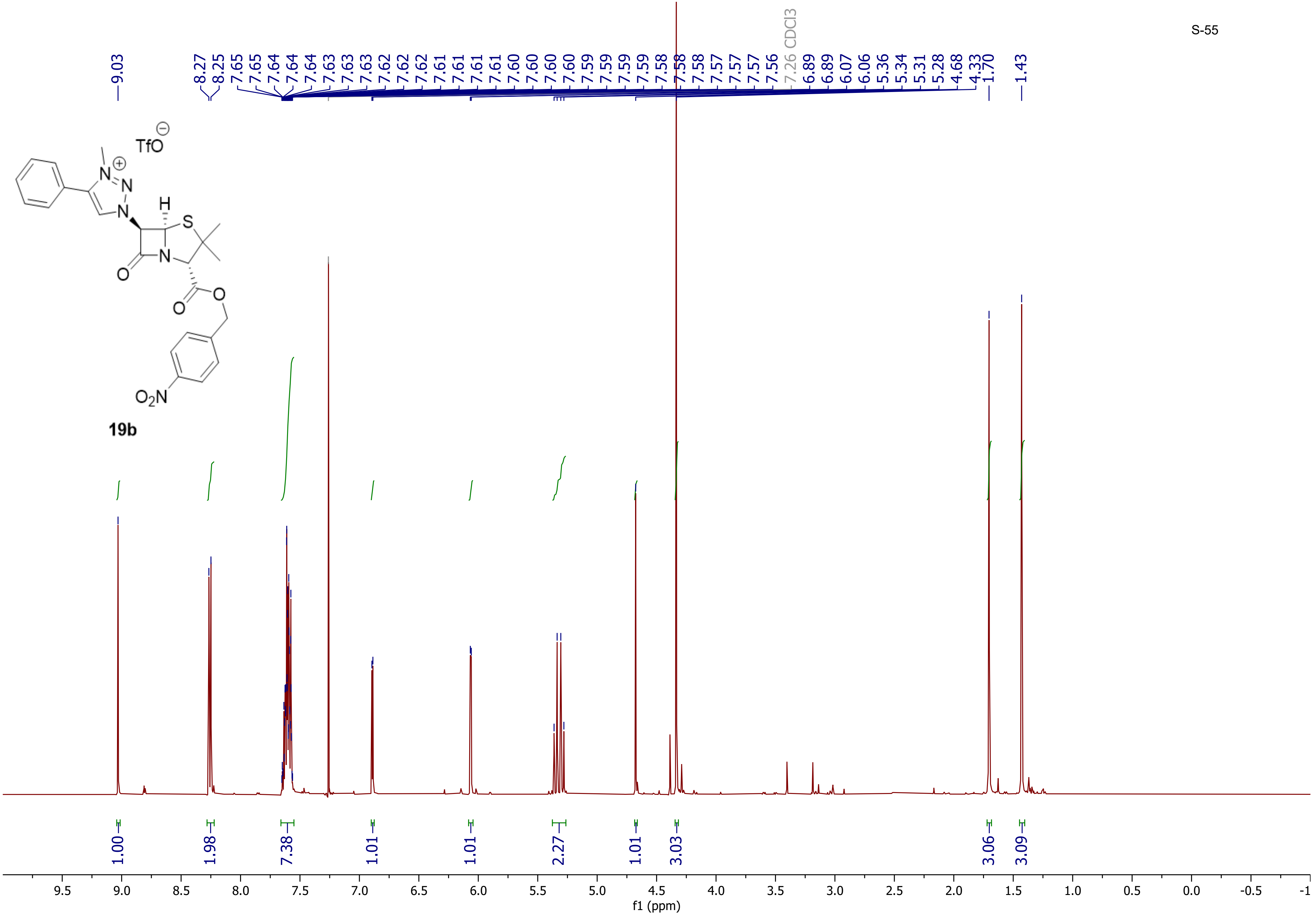

TfO
N
N
N
H
S
N
O
O
O
O2N
19b
9.03
8.27
8.25
7.65
7.65
7.64
7.64
7.64
7.63
7.63
7.63
7.62
7.62
7.62
7.61
7.61
7.61
7.61
7.60
7.60
7.60
7.60
7.59
7.59
7.59
7.59
7.58
7.58
7.58
7.57
7.57
7.57
7.56
7.26 CDCl3
6.89
6.89
6.07
6.06
5.36
5.34
5.31
5.28
4.68
4.33
1.70
1.43
1.00
1.98
7.38
1.01
1.01
2.27
1.01
3.03
3.06
3.09
9.5
9.0
8.5
8.0
7.5
7.0
6.5
6.0
5.5
5.0
4.5
4.0
3.5
3.0
2.5
2.0
1.5
1.0
0.5
0.0
-0.5
-1
f1 (ppm)

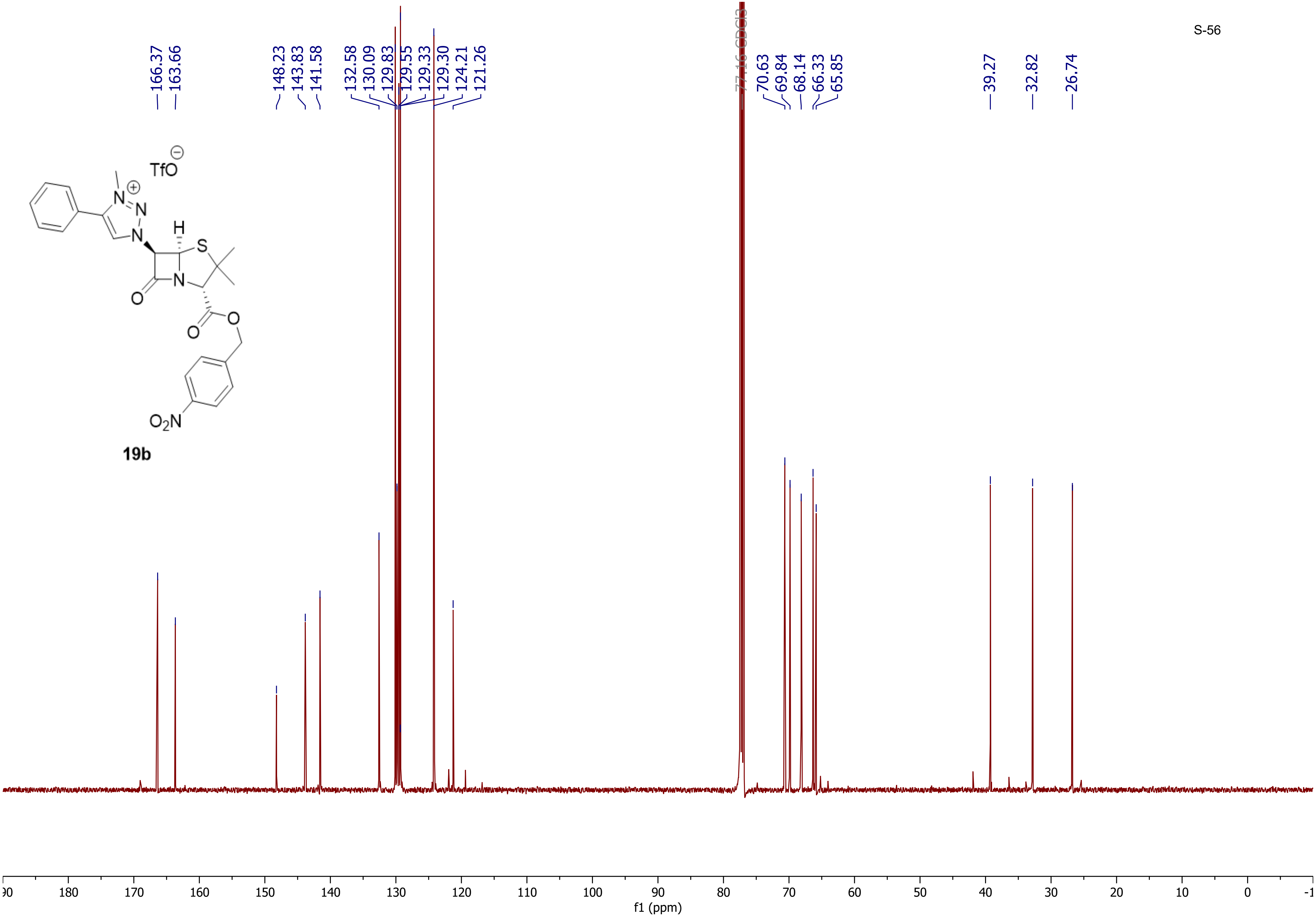
166.37
163.66
148.23
143.83
141.58
132.58
130.09
129.83
129.55
129.33
129.30
124.21
121.26
77.16 CDCl3
70.63
69.84
68.14
66.33
65.85
39.27
32.82
26.74
f1 (ppm)

19b

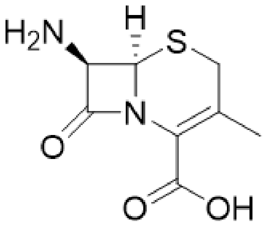

22

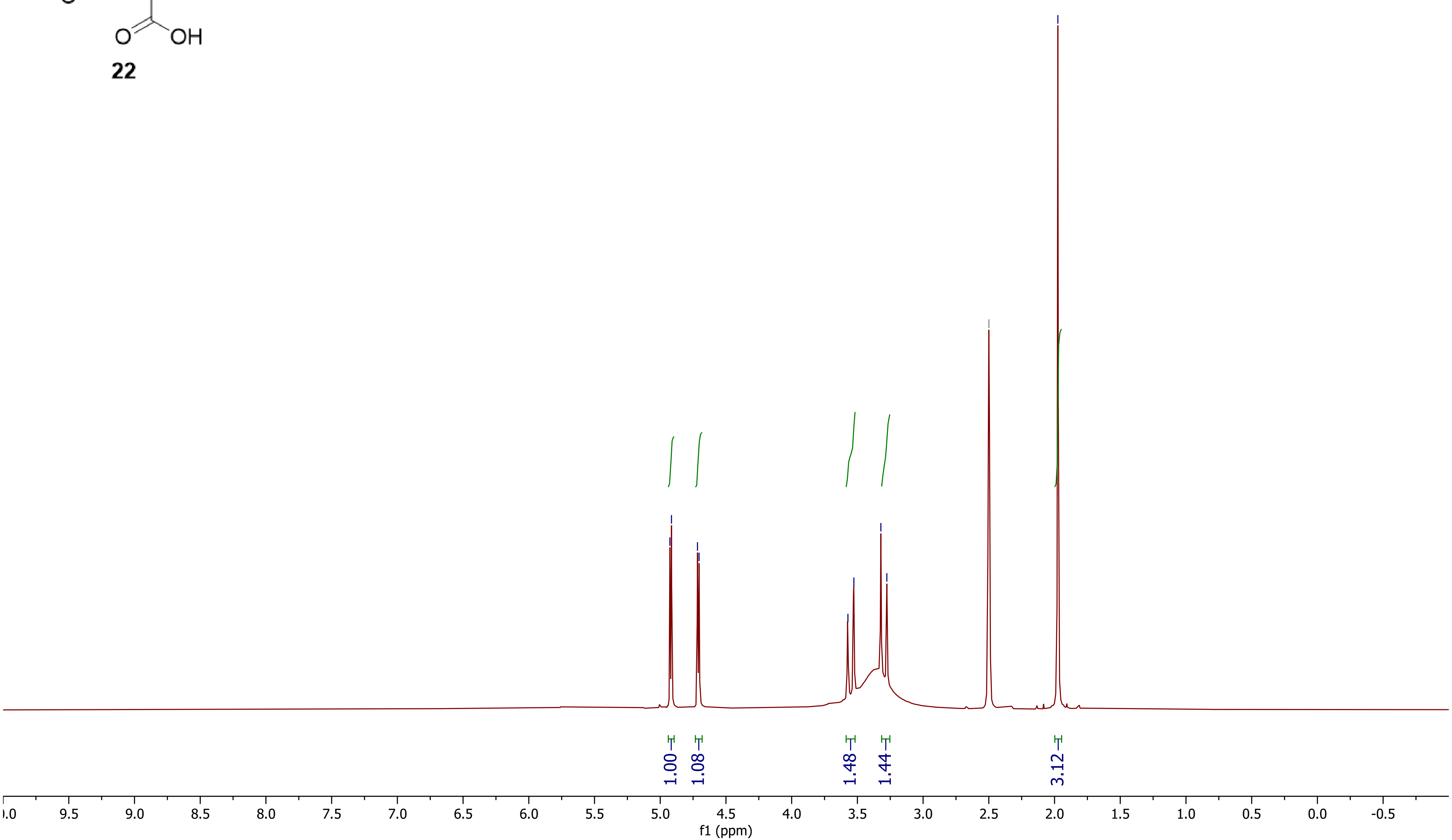
4.93
4.91
4.72
4.70
3.57
3.53
3.32
3.28
2.50 DMSO-d6
1.97
1.00
1.08
1.48
1.44
3.12
9.5
9.0
8.5
8.0
7.5
7.0
6.5
6.0
5.5
5.0
4.5
4.0
3.5
3.0
2.5
2.0
1.5
1.0
0.5
0.0
-0.5
f1 (ppm)

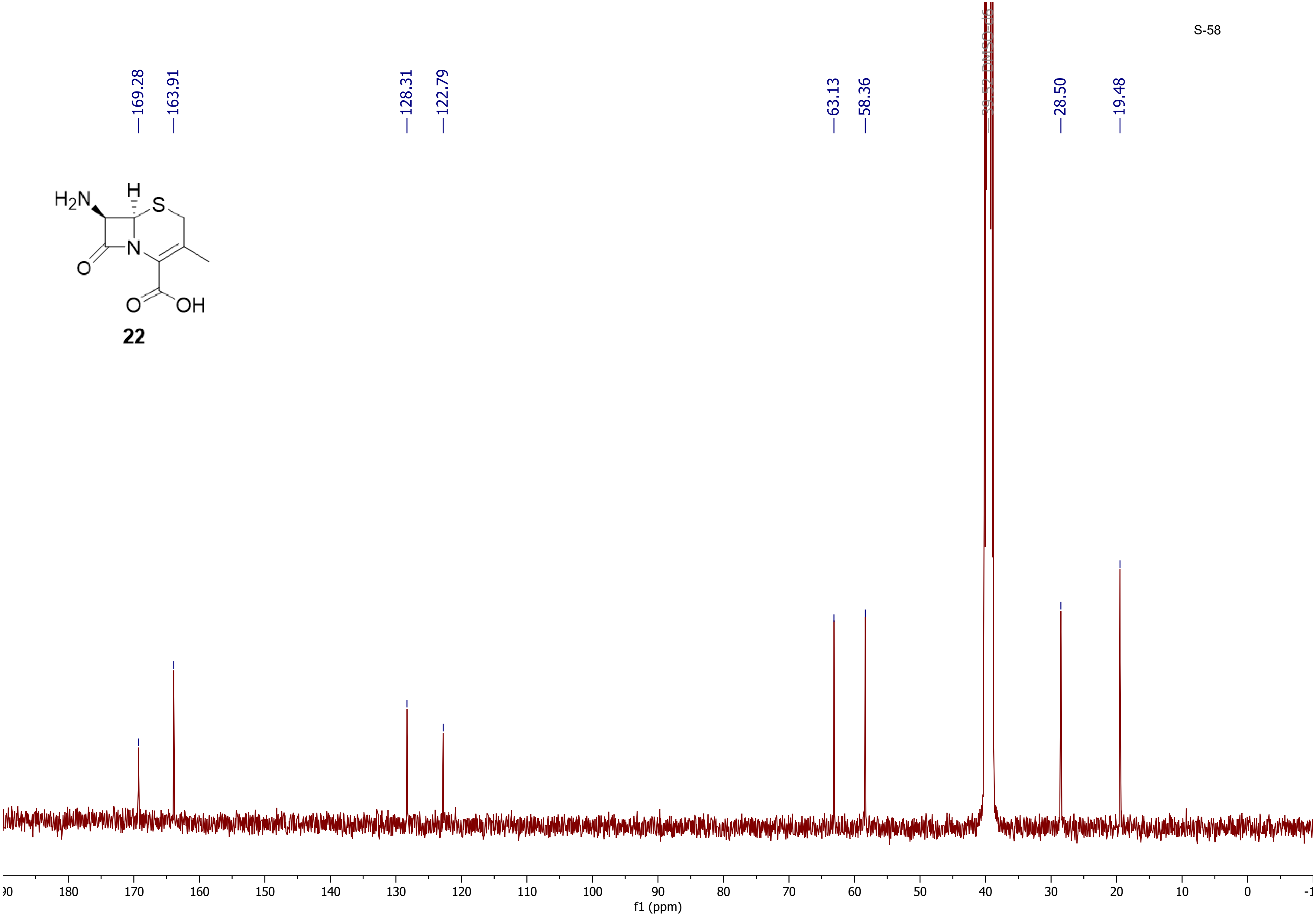
169.28
163.91
128.31
122.79
63.13
58.36
39.52 DMSO-d6
28.50
19.48
180
170
160
150
140
130
120
110
100
90
80
70
60
50
40
30
20
10
0
f1 (ppm)

22

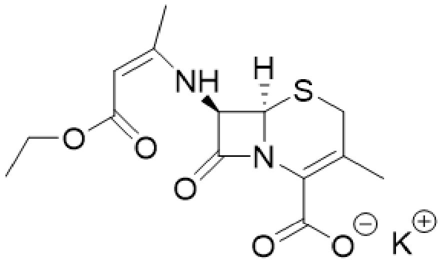
NH
H
S
O
O
N
O
O
O
K

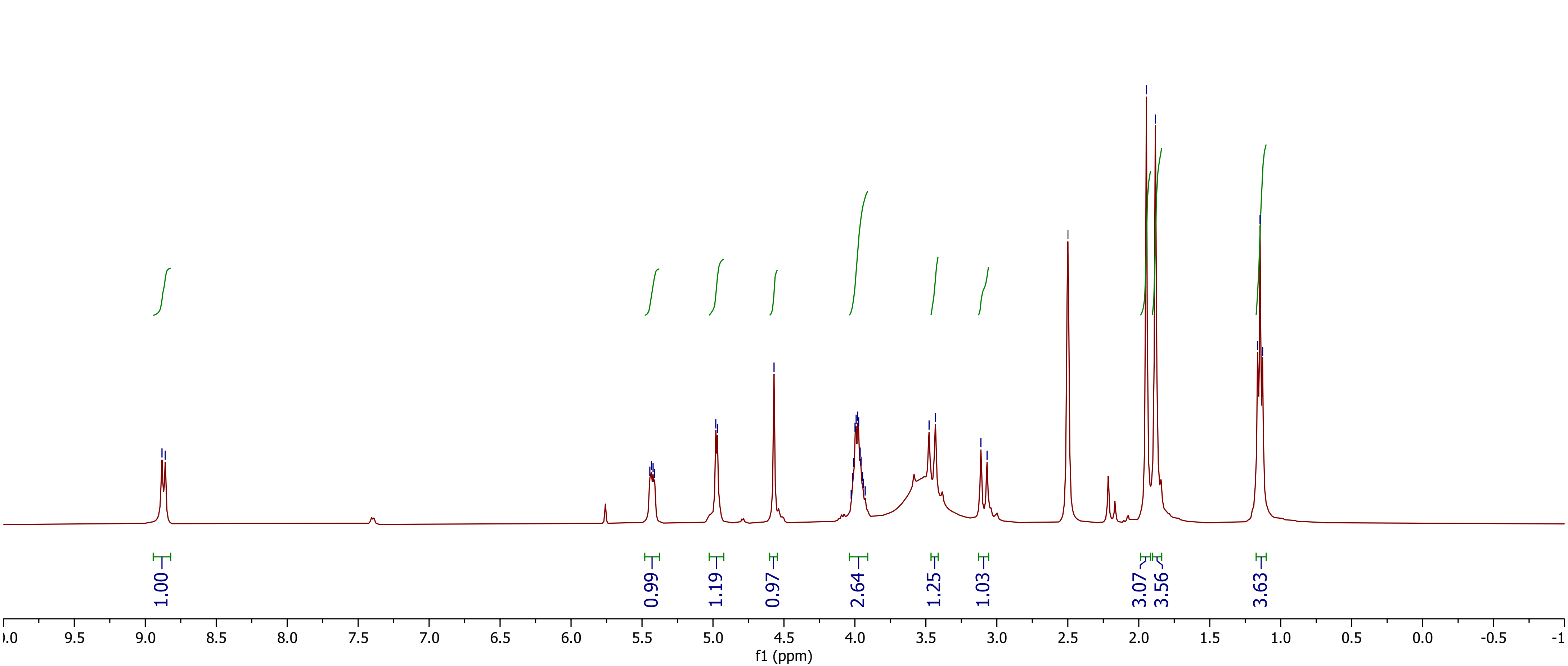
8.88
8.86
5.44
5.43
5.42
5.41
4.98
4.97
4.57
4.03
4.02
4.01
4.00
3.99
3.98
3.97
3.96
3.96
3.95
3.94
3.93
3.48
3.43
3.11
3.07
2.50 DMSO-d6
1.95
1.88
1.16
1.15
1.13
1.00
0.99
1.19
0.97
2.64
1.25
1.03
3.07
3.56
3.63
f1 (ppm)

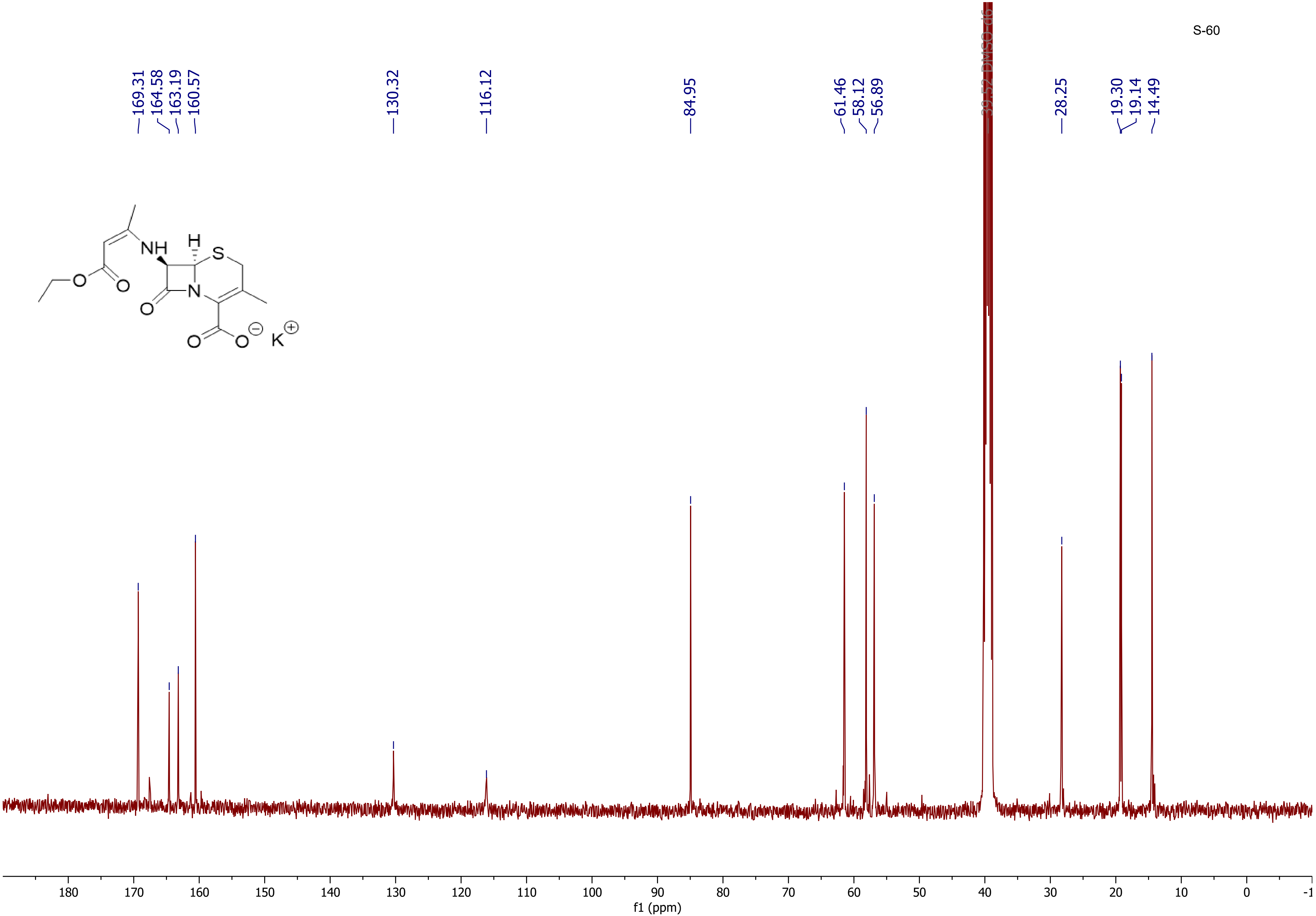
169.31
164.58
163.19
160.57
130.32
116.12
84.95
61.46
58.12
56.89
39.52 DMSO-d6
28.25
19.30
19.14
14.49
NH
H
S
N
O
K
180 170 160 150 140 130 120 110 100 90 80 70 60 50 40 30 20 10 0 -1
f1 (ppm)

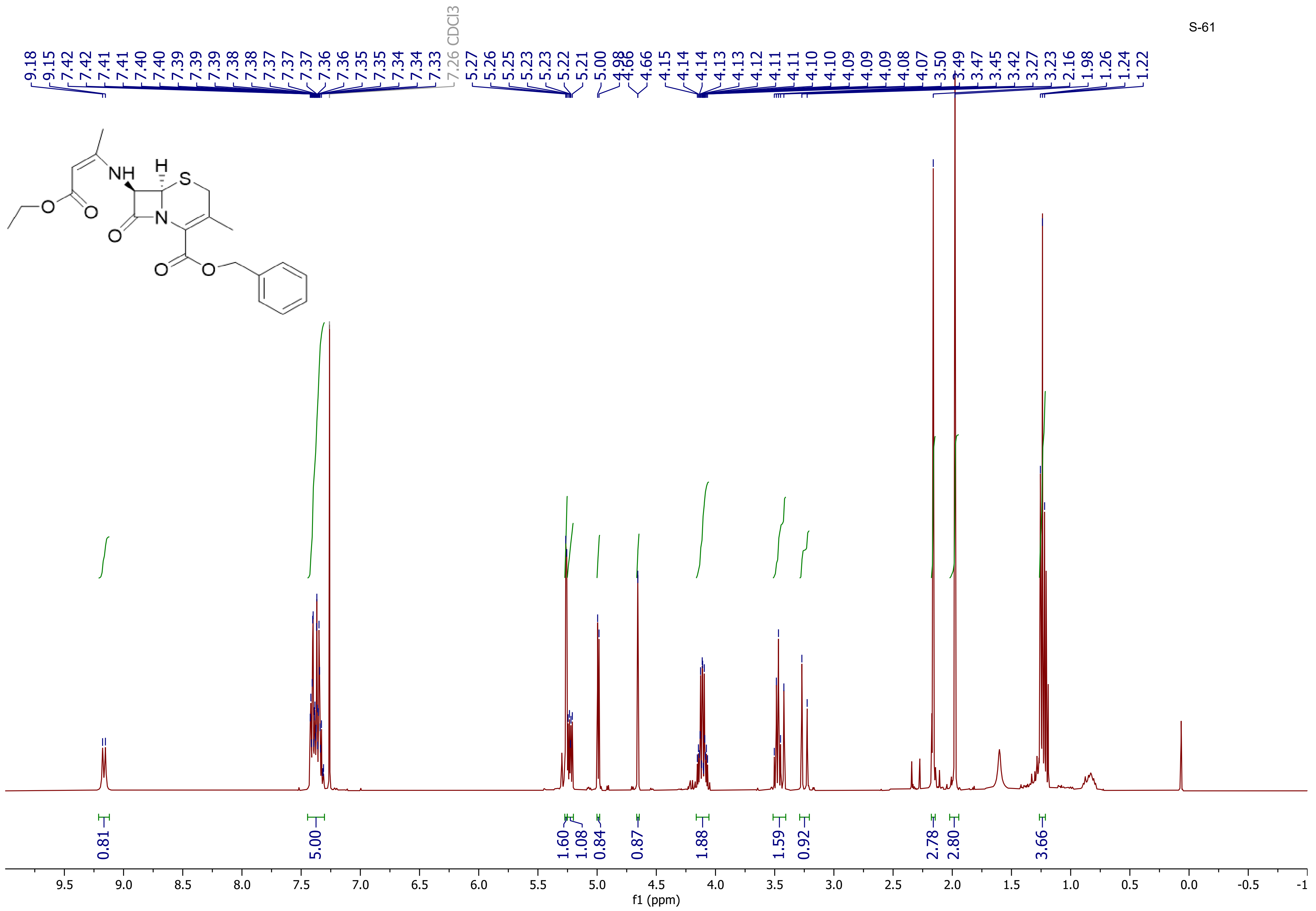
9.18
9.15
7.42
7.42
7.41
7.41
7.40
7.40
7.39
7.39
7.39
7.38
7.38
7.37
7.37
7.37
7.36
7.36
7.35
7.35
7.34
7.34
7.33
7.26 CDCl3
5.27
5.26
5.25
5.23
5.23
5.22
5.21
5.00
4.98
4.66
4.66
4.15
4.14
4.14
4.13
4.13
4.12
4.11
4.11
4.10
4.10
4.09
4.09
4.09
4.08
4.07
3.50
3.49
3.47
3.45
3.42
3.27
3.23
2.16
1.98
1.26
1.24
1.22
NH
H
S
O
N
O
O
O
O
O
0.81
5.00
1.60
1.08
0.84
0.87
1.88
1.59
0.92
2.78
2.80
3.66
9.5
9.0
8.5
8.0
7.5
7.0
6.5
6.0
5.5
5.0
4.5
4.0
3.5
3.0
2.5
2.0
1.5
1.0
0.5
0.0
-0.5
-1
f1 (ppm)

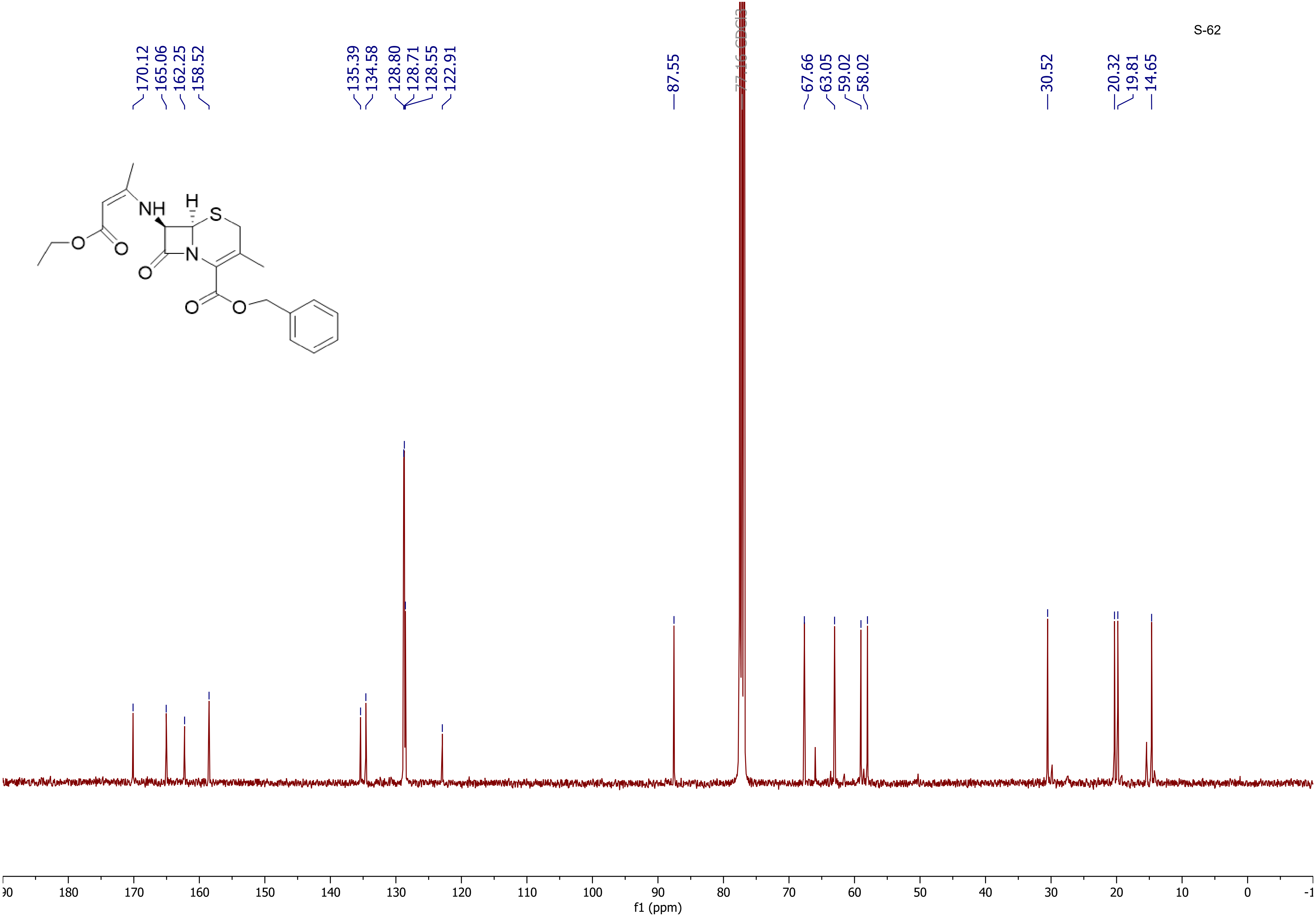
170.12
165.06
162.25
158.52
135.39
134.58
128.80
128.71
128.55
122.91
87.55
77.16 CDCl3
67.66
63.05
59.02
58.02
30.52
20.32
19.81
14.65
NH
H
S
O
O
N
O
O
O
90
180
170
160
150
140
130
120
110
100
90
80
70
60
50
40
30
20
10
0
-1
f1 (ppm)

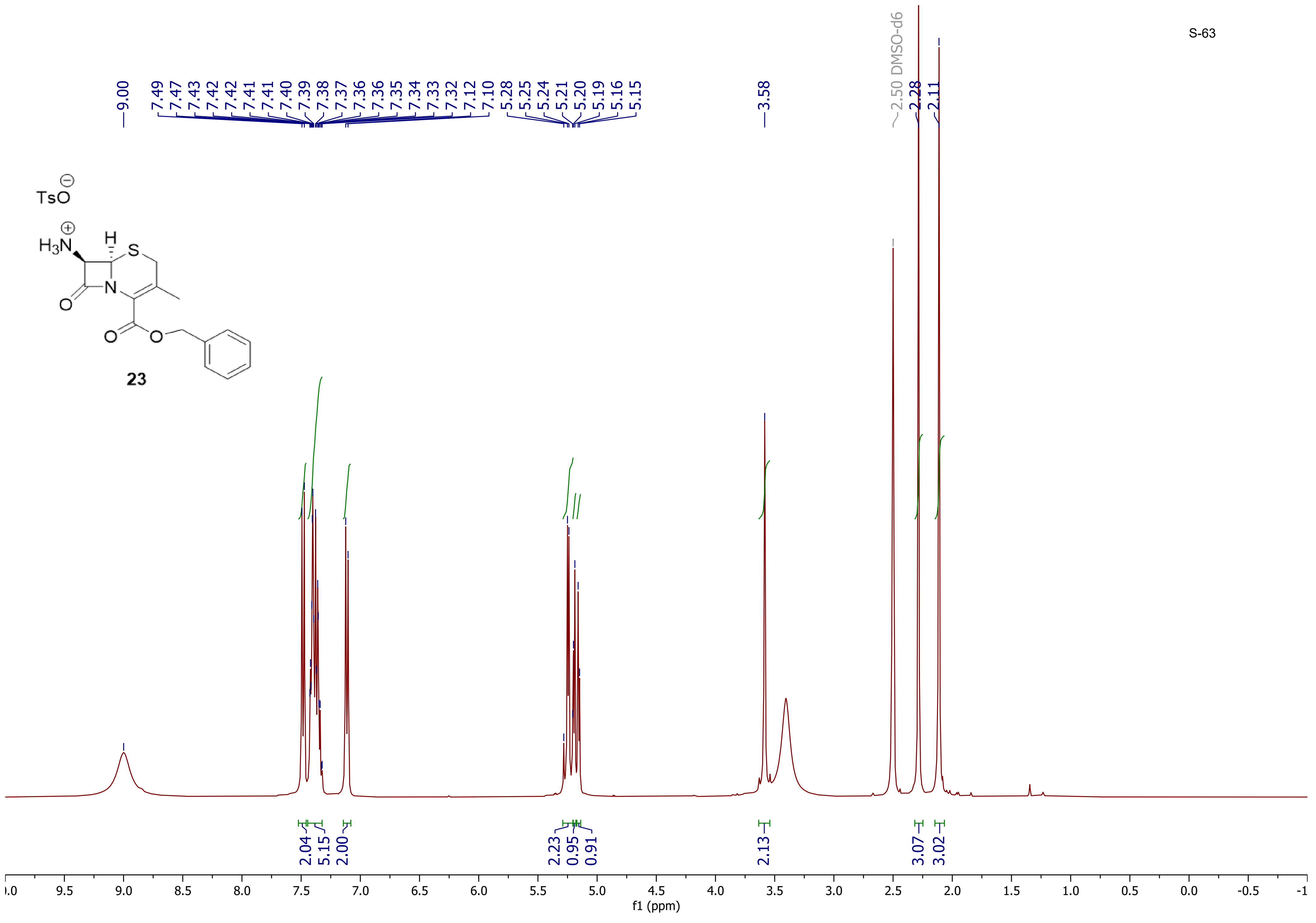
TsO
H3N
H
S
N
O
O
O
23
9.00
7.49
7.47
7.43
7.42
7.42
7.41
7.41
7.40
7.39
7.38
7.37
7.36
7.36
7.35
7.34
7.33
7.32
7.12
7.10
5.28
5.25
5.24
5.21
5.20
5.19
5.16
5.15
3.58
2.50 DMSO-d6
2.28
2.11
2.04
5.15
2.00
2.23
0.95
0.91
2.13
3.07
3.02
f1 (ppm)

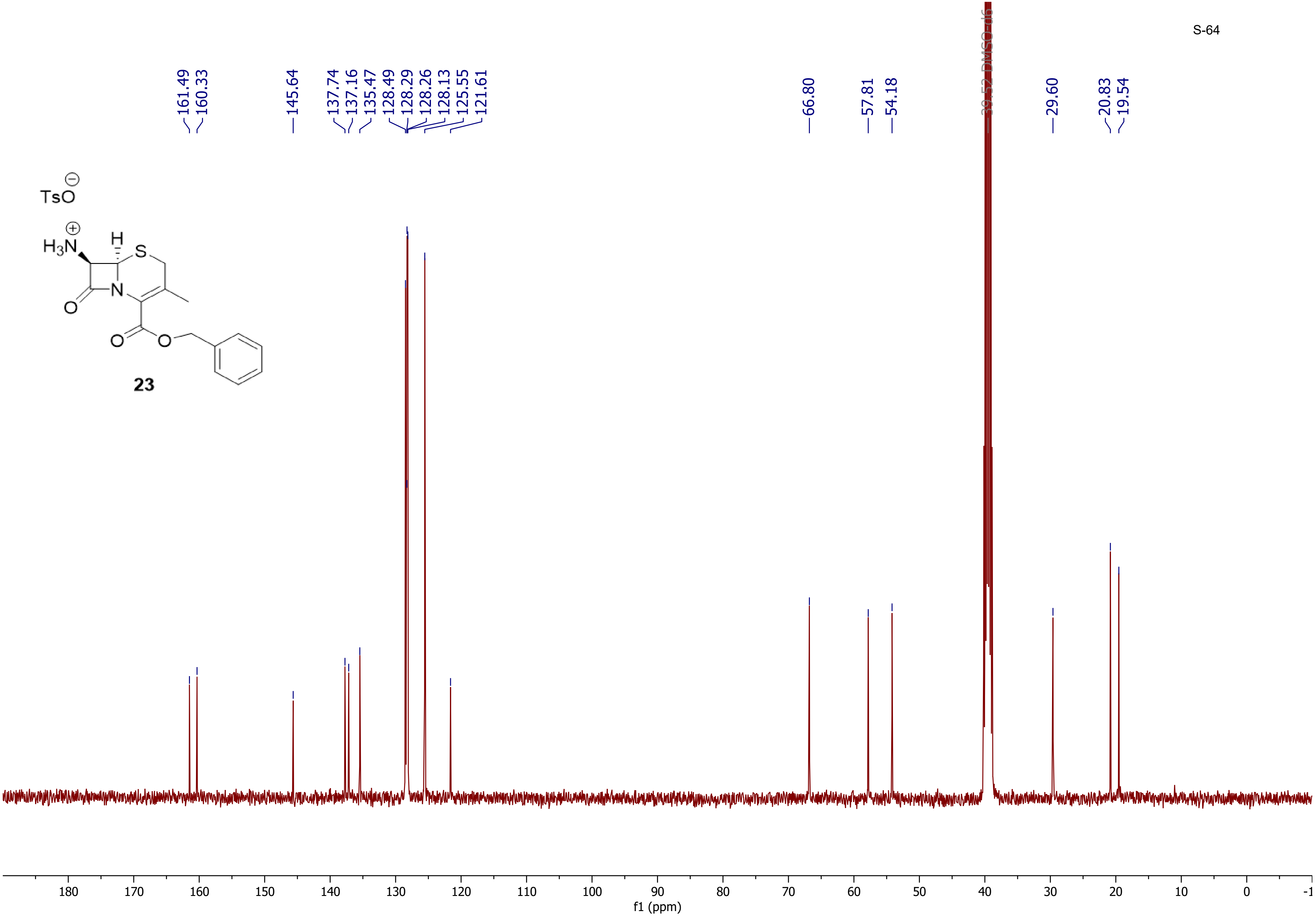
161.49
160.33
145.64
137.74
137.16
135.47
128.49
128.29
128.26
128.13
125.55
121.61
66.80
57.81
54.18
39.52 DMSO-d6
29.60
20.83
19.54
TsO
H3N
H
S
N
O
O
O
180 170 160 150 140 130 120 110 100 90 80 70 60 50 40 30 20 10 0 -1
f1 (ppm)

23

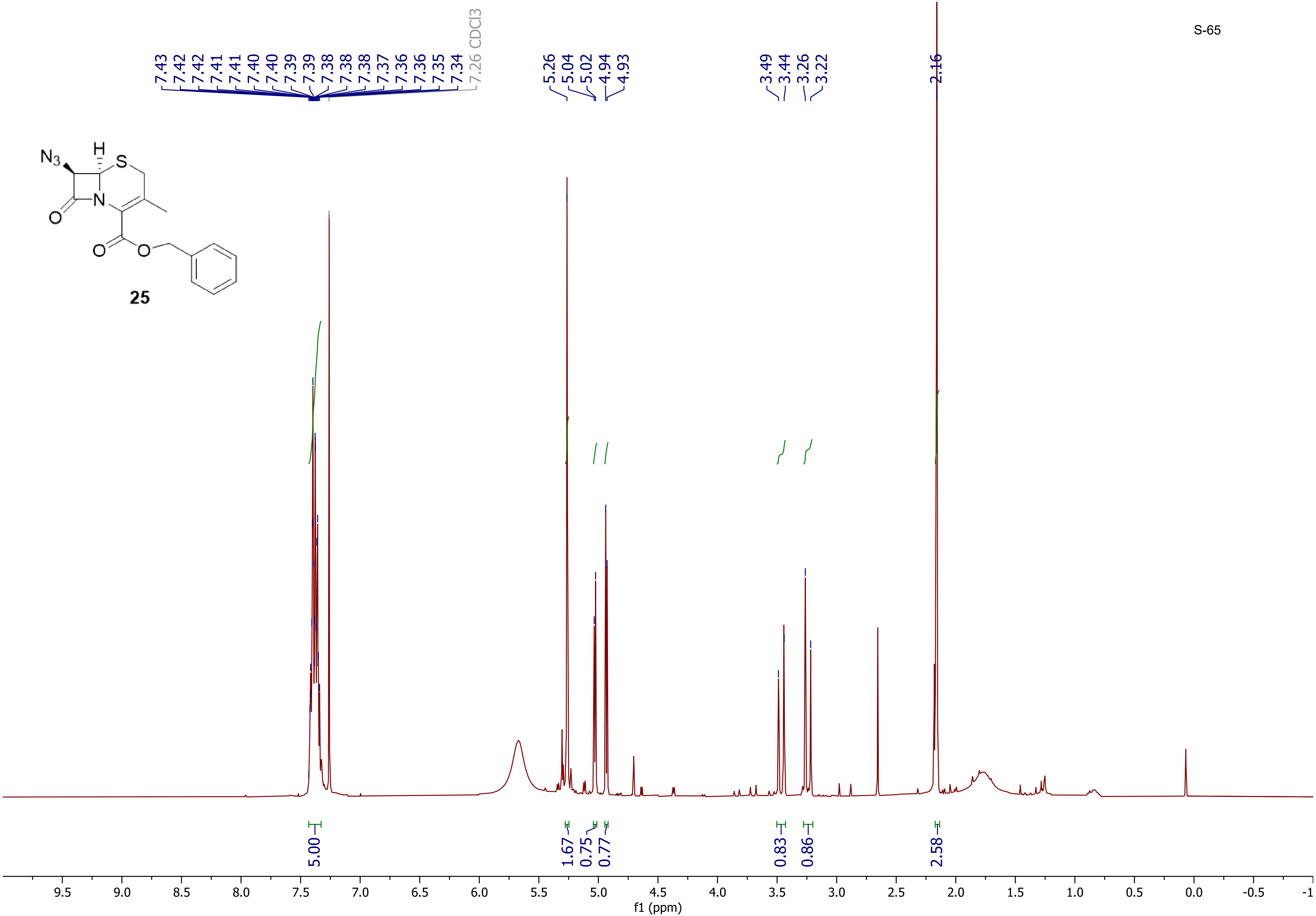
7.43
7.42
7.42
7.41
7.41
7.40
7.40
7.39
7.39
7.38
7.38
7.38
7.37
7.36
7.36
7.35
7.34
7.26 CDCl3
5.26
5.04
5.02
4.94
4.93
3.49
3.44
3.26
3.22
2.16
N3
H
S
N
O
O
O
25
5.00
1.67
0.75
0.77
0.83
0.86
2.58
9.5
9.0
8.5
8.0
7.5
7.0
6.5
6.0
5.5
5.0
4.5
4.0
3.5
3.0
2.5
2.0
1.5
1.0
0.5
0.0
-0.5
-1
f1 (ppm)

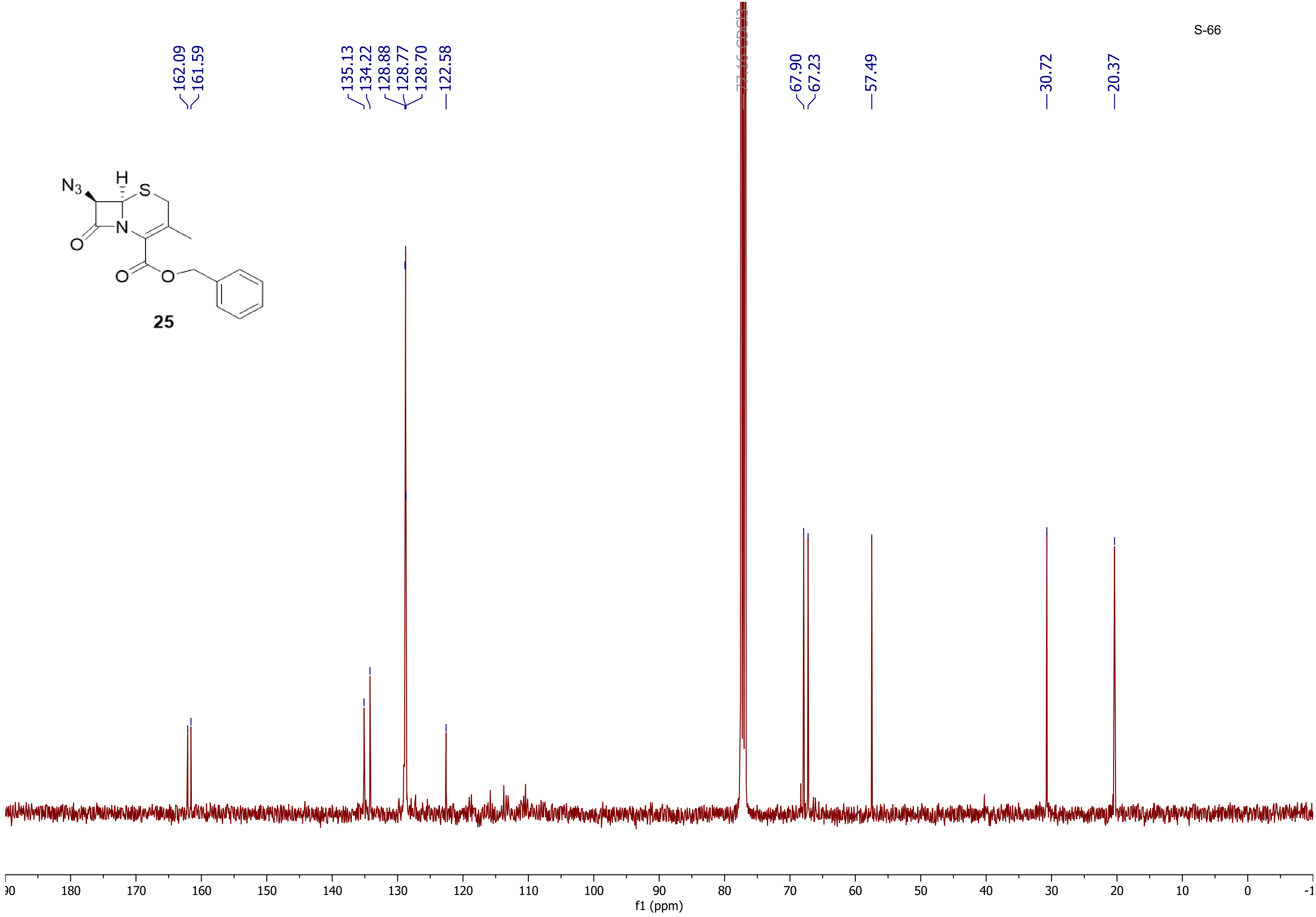
162.09
161.59
135.13
134.22
128.88
128.77
128.70
122.58
77.16 CDCl3
67.90
67.23
57.49
30.72
20.37
N3
H
S
N
O
O
O
25
90
180
170
160
150
140
130
120
110
100
90
80
70
60
50
40
30
20
10
0
-1
f1 (ppm)

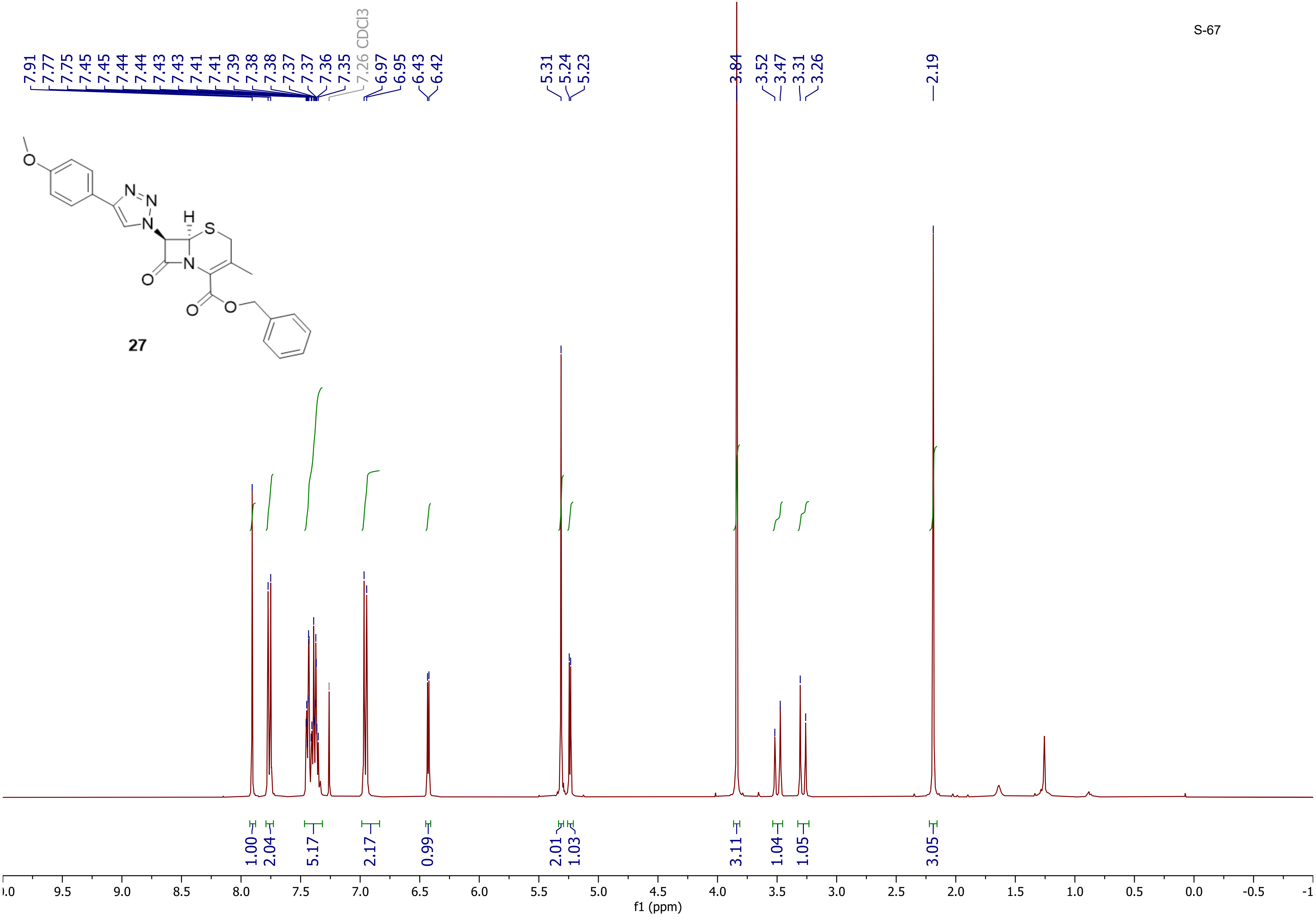
7.91 7.77 7.75 7.45 7.45 7.44 7.44 7.43 7.43 7.41 7.41 7.39 7.38 7.38 7.37 7.37 7.36 7.35 7.26 CDCl3 6.97 6.95 6.43 6.42
5.31 5.24 5.23
3.84 3.52 3.47 3.31 3.26
2.19
27
1.00 2.04 5.17 2.17 0.99 2.01 1.03 3.11 1.04 1.05 3.05
f1 (ppm)

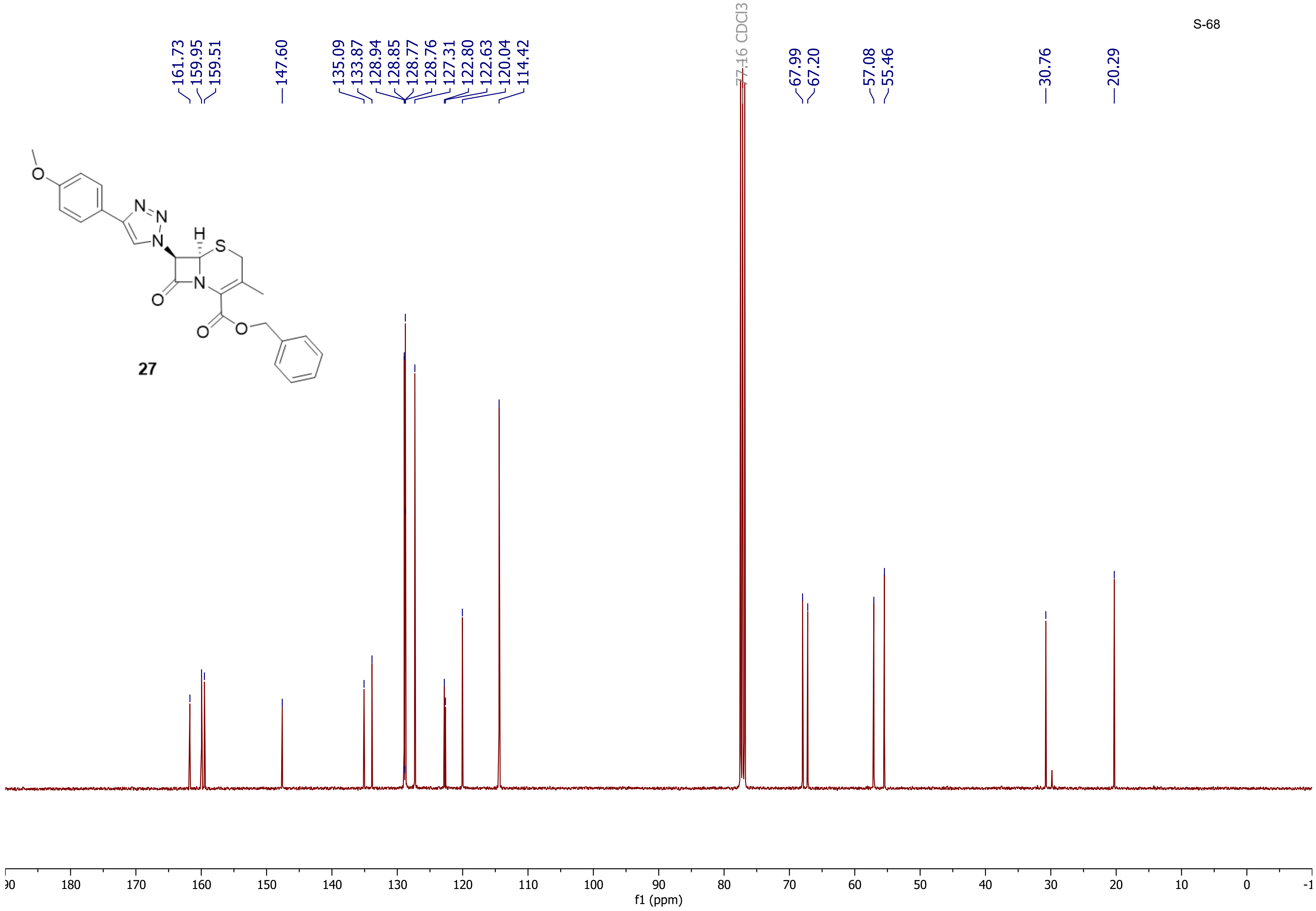
161.73
159.95
159.51
147.60
135.09
133.87
128.94
128.85
128.77
128.76
127.31
122.80
122.63
120.04
114.42
77.16 CDCl3
67.99
67.20
57.08
55.46
30.76
20.29
27
f1 (ppm)
180
170
160
150
140
130
120
110
100
90
80
70
60
50
40
30
20
10
0

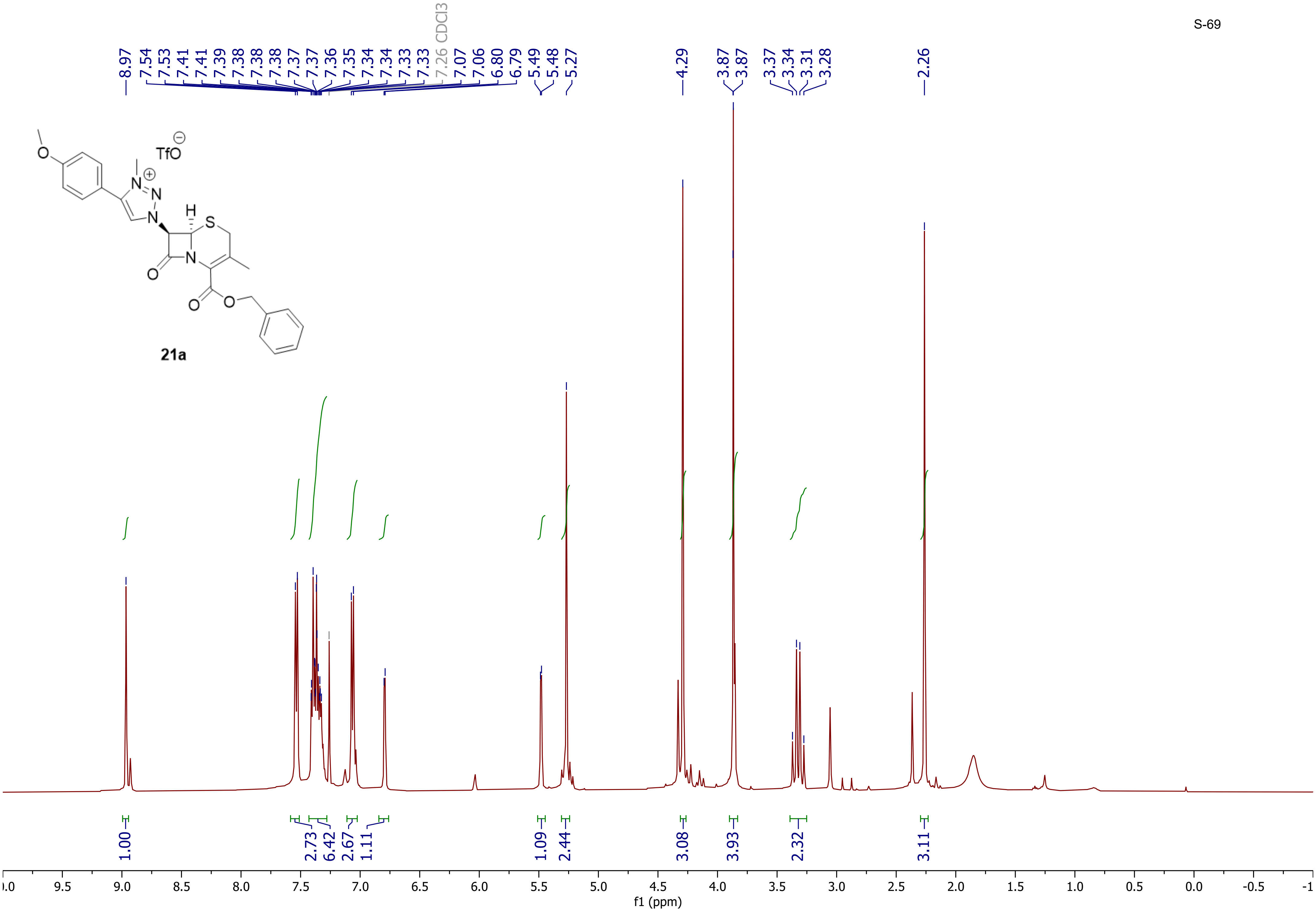

TfO
N
N
N
H
S
N
O
O
O
21a
8.97
7.54
7.53
7.41
7.41
7.39
7.38
7.38
7.38
7.37
7.37
7.36
7.35
7.34
7.34
7.33
7.33
7.26 CDCl3
7.07
7.06
6.80
6.79
5.49
5.48
5.27
4.29
3.87
3.87
3.37
3.34
3.31
3.28
2.26
1.00
2.73
6.42
2.67
1.11
1.09
2.44
3.08
3.93
2.32
3.11
f1 (ppm)

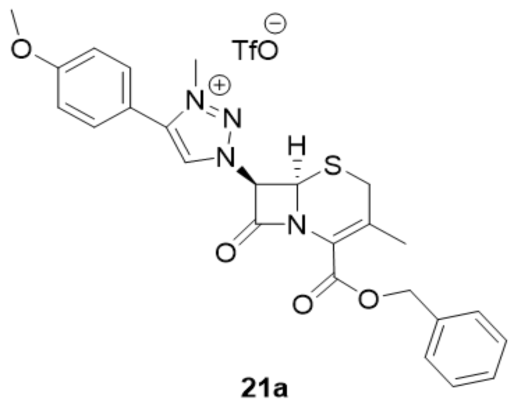
TfO
N
N
N
H
S
N
O
O
O
O

21a

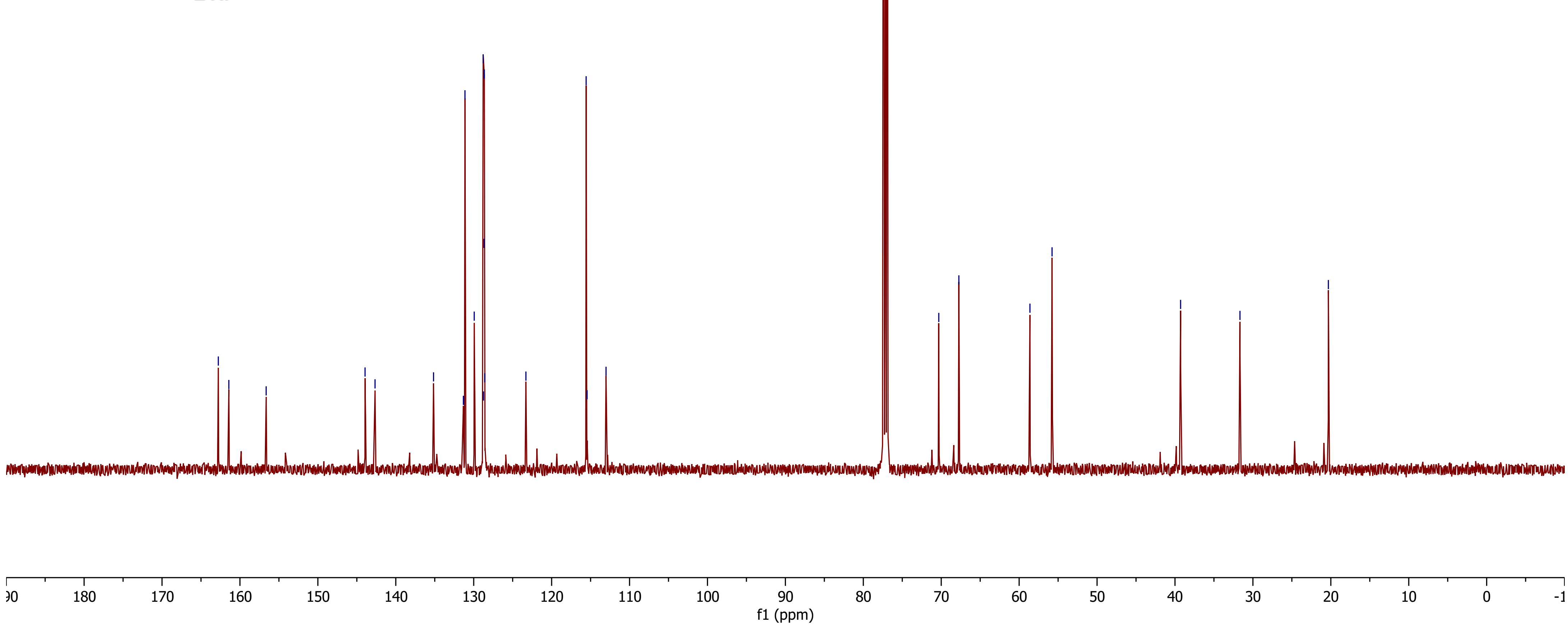
162.78
161.43
156.64
143.94
142.66
135.15
131.33
131.12
129.93
128.79
128.75
128.67
128.64
128.58
123.30
115.57
115.45
113.02
77.16 CDCl3
70.31
67.74
58.61
55.78
39.28
31.66
20.31
f1 (ppm)

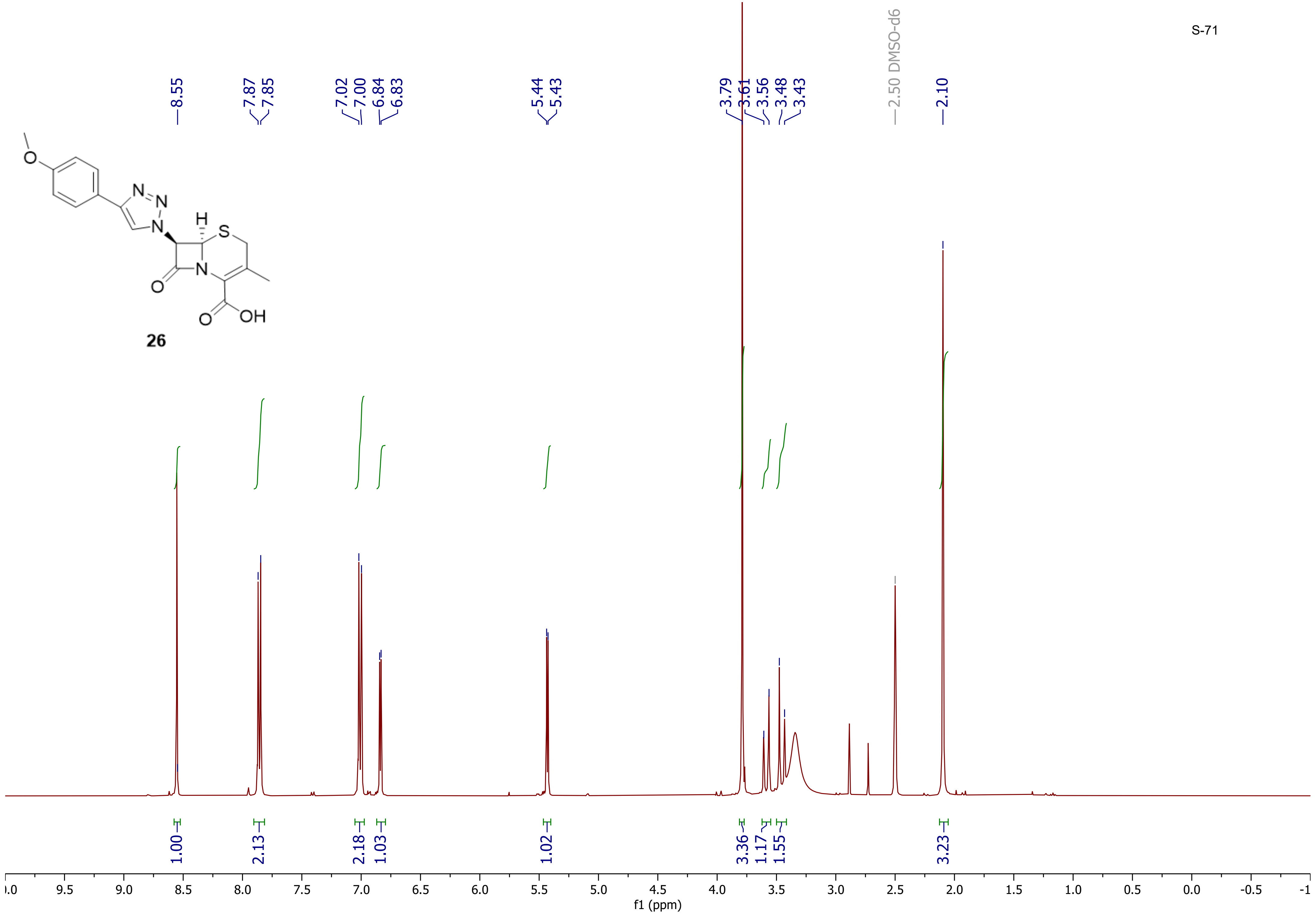
26
8.55
7.87
7.85
7.02
7.00
6.84
6.83
5.44
5.43
3.79
3.61
3.56
3.48
3.43
2.50 DMSO-d6
2.10
1.00
2.13
2.18
1.03
1.02
3.36
1.17
1.55
3.23
f1 (ppm)

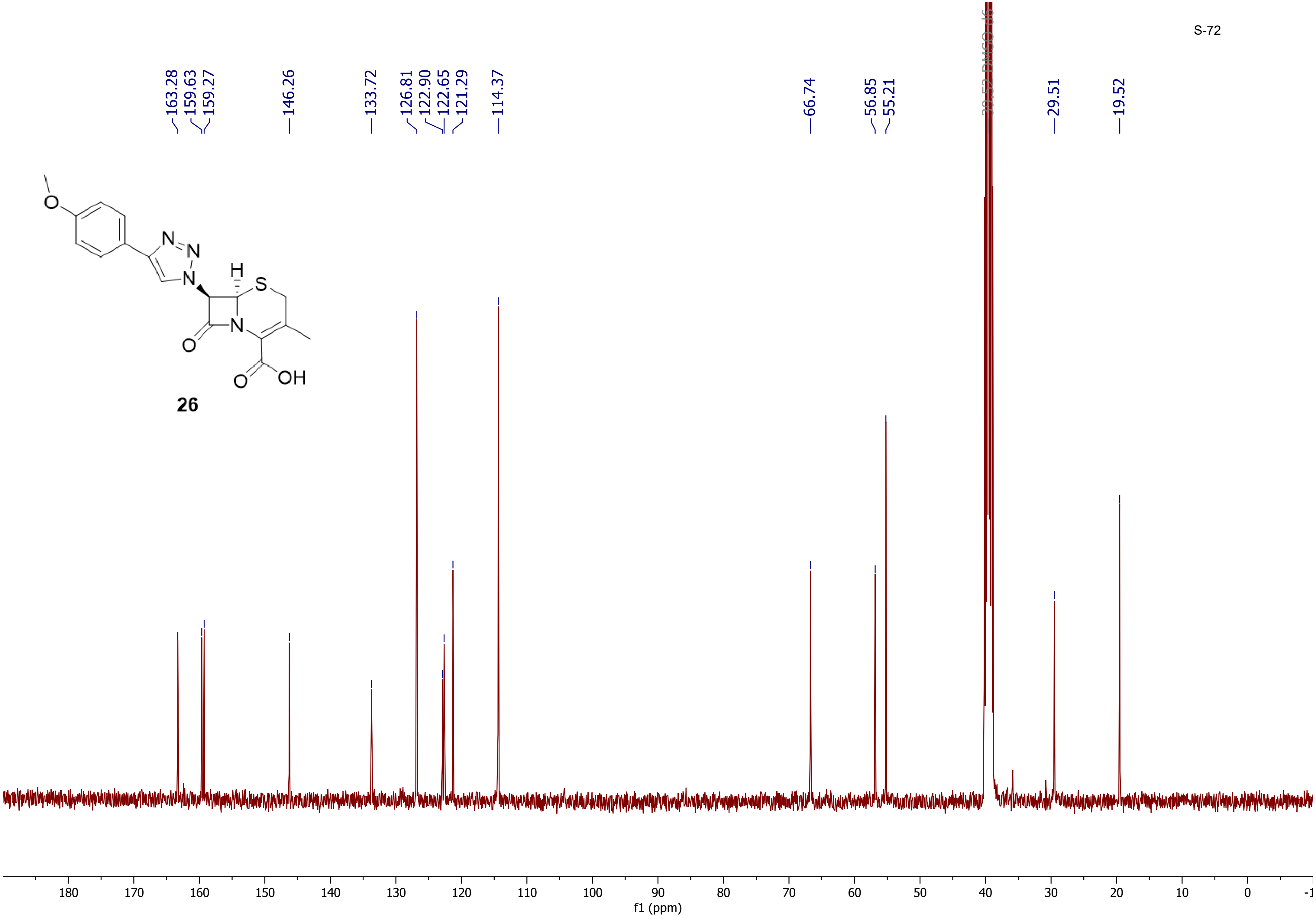
26
163.28
159.63
159.27
146.26
133.72
126.81
122.90
122.65
121.29
114.37
66.74
56.85
55.21
39.52 DMSO-d6
29.51
19.52
180
170
160
150
140
130
120
110
100
90
80
70
60
50
40
30
20
10
0
-1
f1 (ppm)

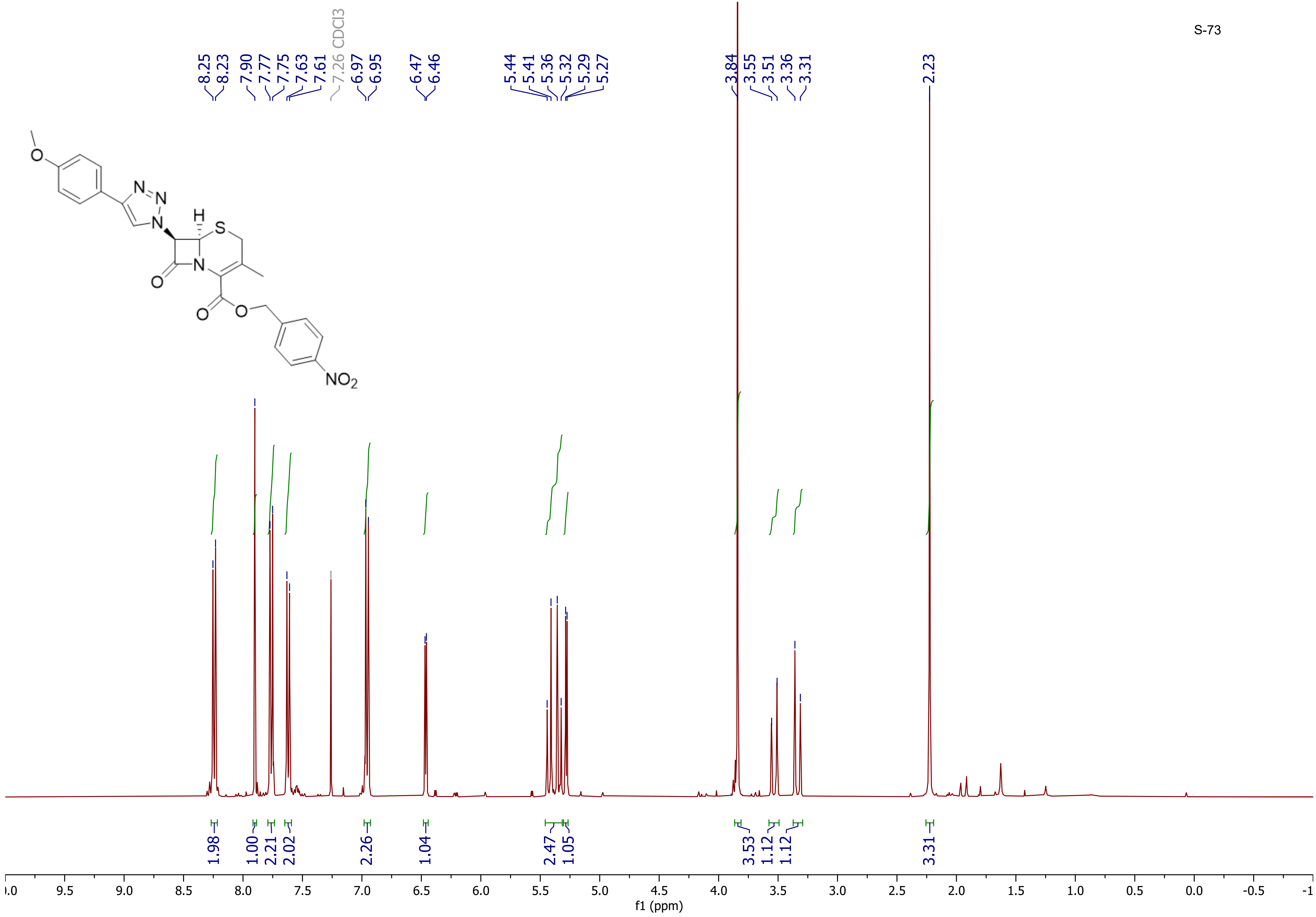
8.25
8.23
7.90
7.77
7.75
7.63
7.61
7.26 CDCl3
6.97
6.95
6.47
6.46
5.44
5.41
5.36
5.32
5.29
5.27
3.84
3.55
3.51
3.36
3.31
2.23
1.98
1.00
2.21
2.02
2.26
1.04
2.47
1.05
3.53
1.12
1.12
3.31
NO2
f1 (ppm)

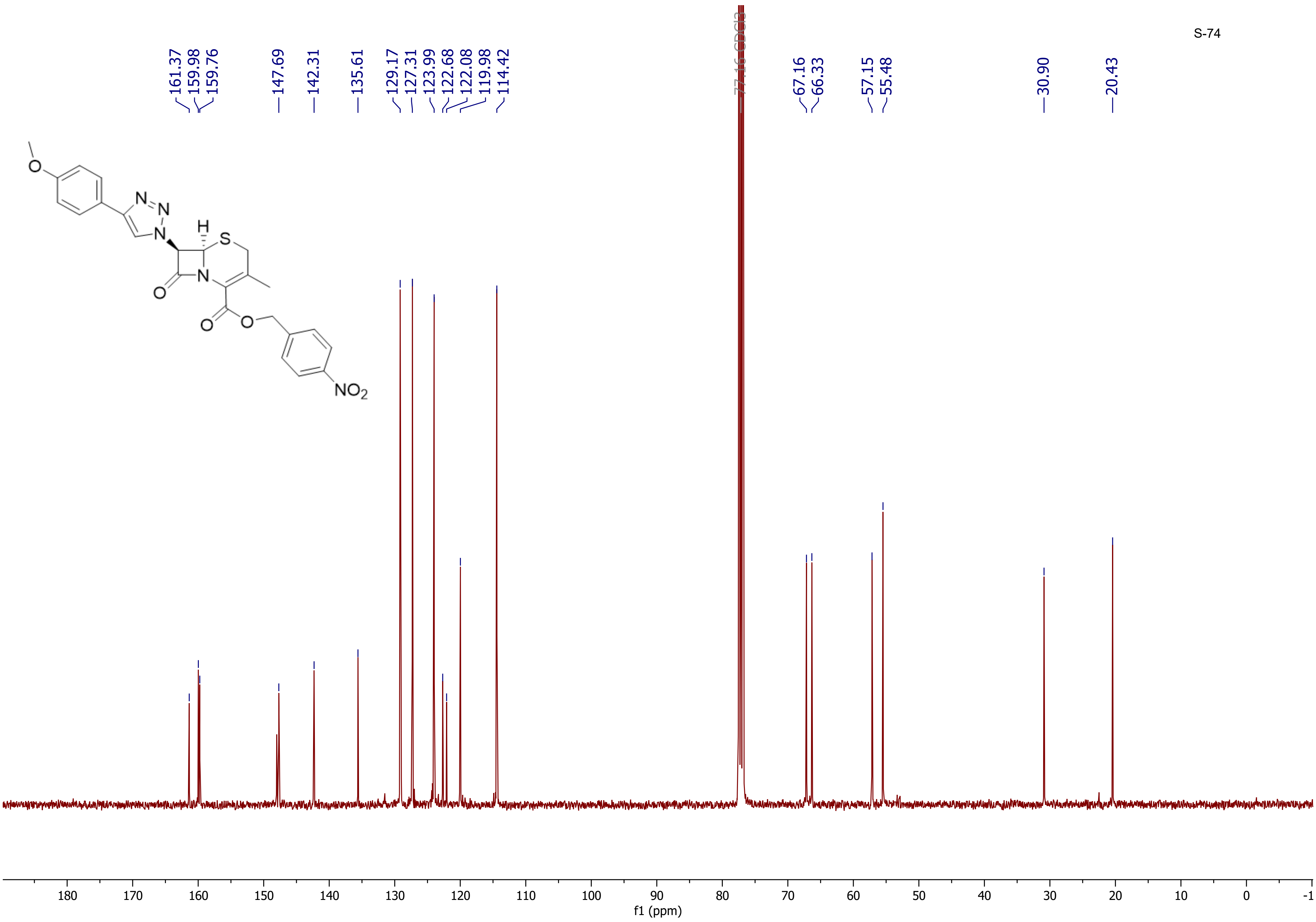

161.37
159.98
159.76
147.69
142.31
135.61
129.17
127.31
123.99
122.68
122.08
119.98
114.42
77.16 CDCl3
67.16
66.33
57.15
55.48
30.90
20.43
O
N
N
N
H
S
N
O
O
O
NO2
180
170
160
150
140
130
120
110
100
90
80
70
60
50
40
30
20
10
0
-1
f1 (ppm)

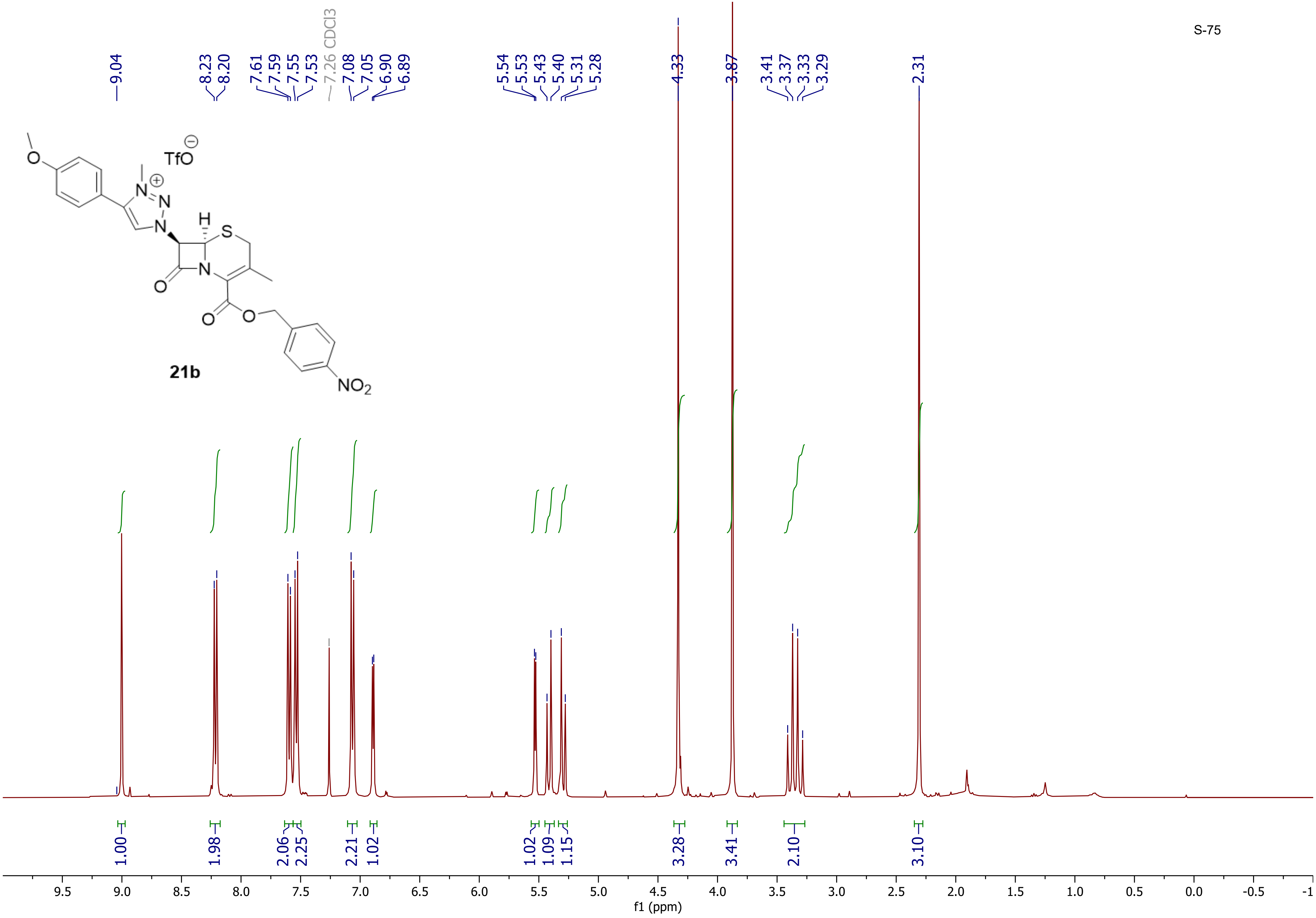
9.04
8.23
8.20
7.61
7.59
7.55
7.53
7.26 CDCl3
7.08
7.05
6.90
6.89
5.54
5.53
5.43
5.40
5.31
5.28
4.33
3.87
3.41
3.37
3.33
3.29
2.31
TfO
21b
NO2
1.00
1.98
2.06
2.25
2.21
1.02
1.02
1.09
1.15
3.28
3.41
2.10
3.10
f1 (ppm)

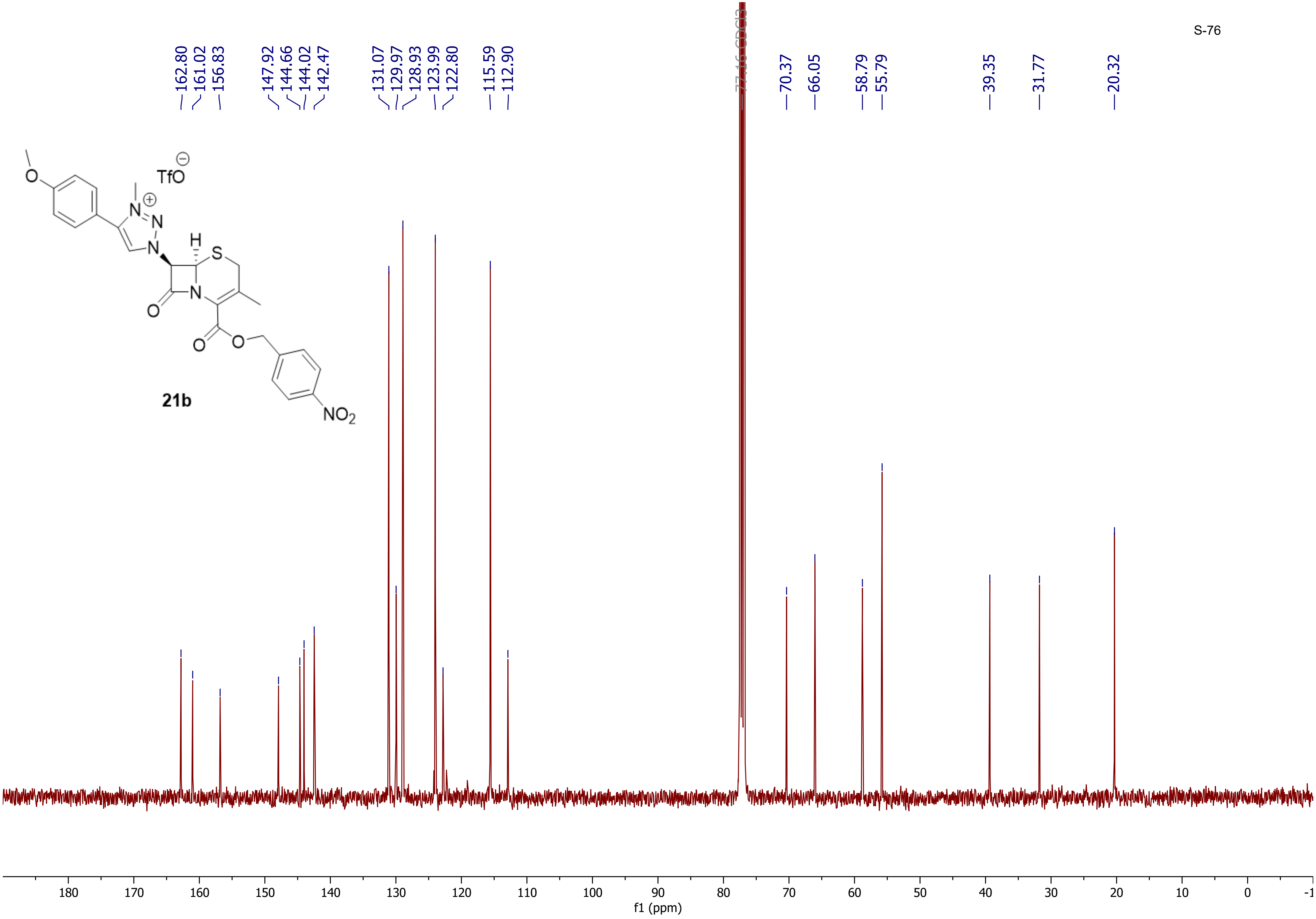
162.80
161.02
156.83
147.92
144.66
144.02
142.47
131.07
129.97
128.93
123.99
122.80
115.59
112.90
77.16 CDCl3
70.37
66.05
58.79
55.79
39.35
31.77
20.32
TfO
NO2
21b
f1 (ppm)
180
170
160
150
140
130
120
110
100
90
80
70
60
50
40
30
20
10
0
-1

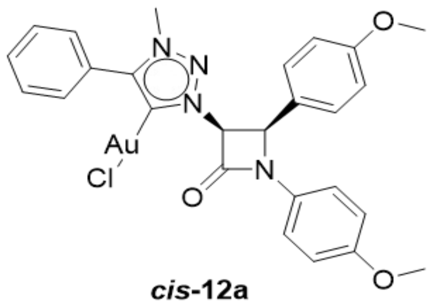
N
N
N
Au
Cl
O
N
O
O

***cis*-12a**

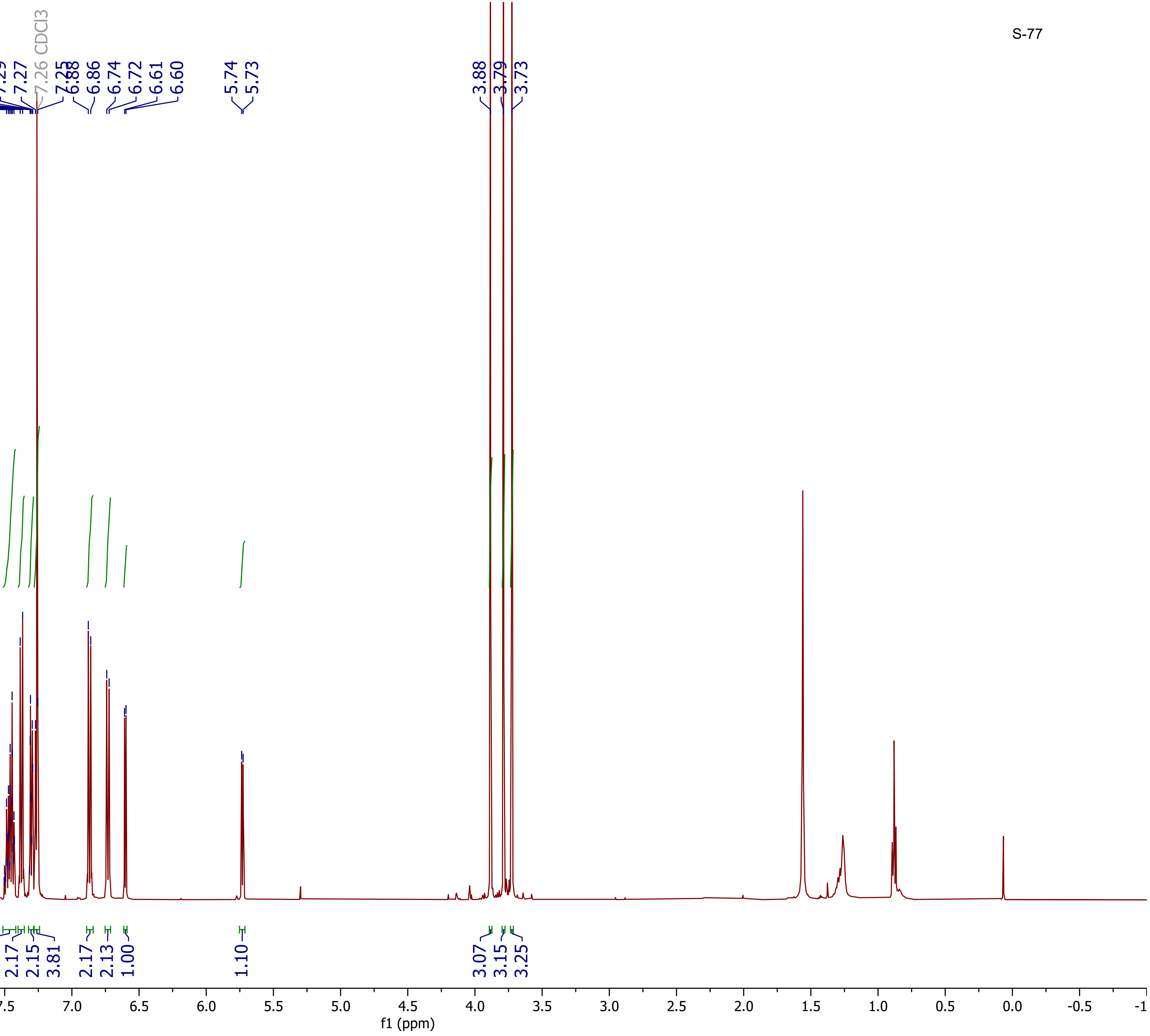
7.49
7.47
7.46
7.46
7.45
7.44
7.44
7.43
7.38
7.37
7.31
7.31
7.30
7.29
7.29
7.27
7.26 CDCl3
7.25
6.88
6.86
6.74
6.72
6.61
6.60
5.74
5.73
3.88
3.79
3.73
3.26
2.17
2.15
3.81
2.17
2.13
1.00
1.10
3.07
3.15
3.25
9.5
9.0
8.5
8.0
7.5
7.0
6.5
6.0
5.5
5.0
4.5
4.0
3.5
3.0
2.5
2.0
1.5
1.0
0.5
0.0
-0.5
-1
f1 (ppm)

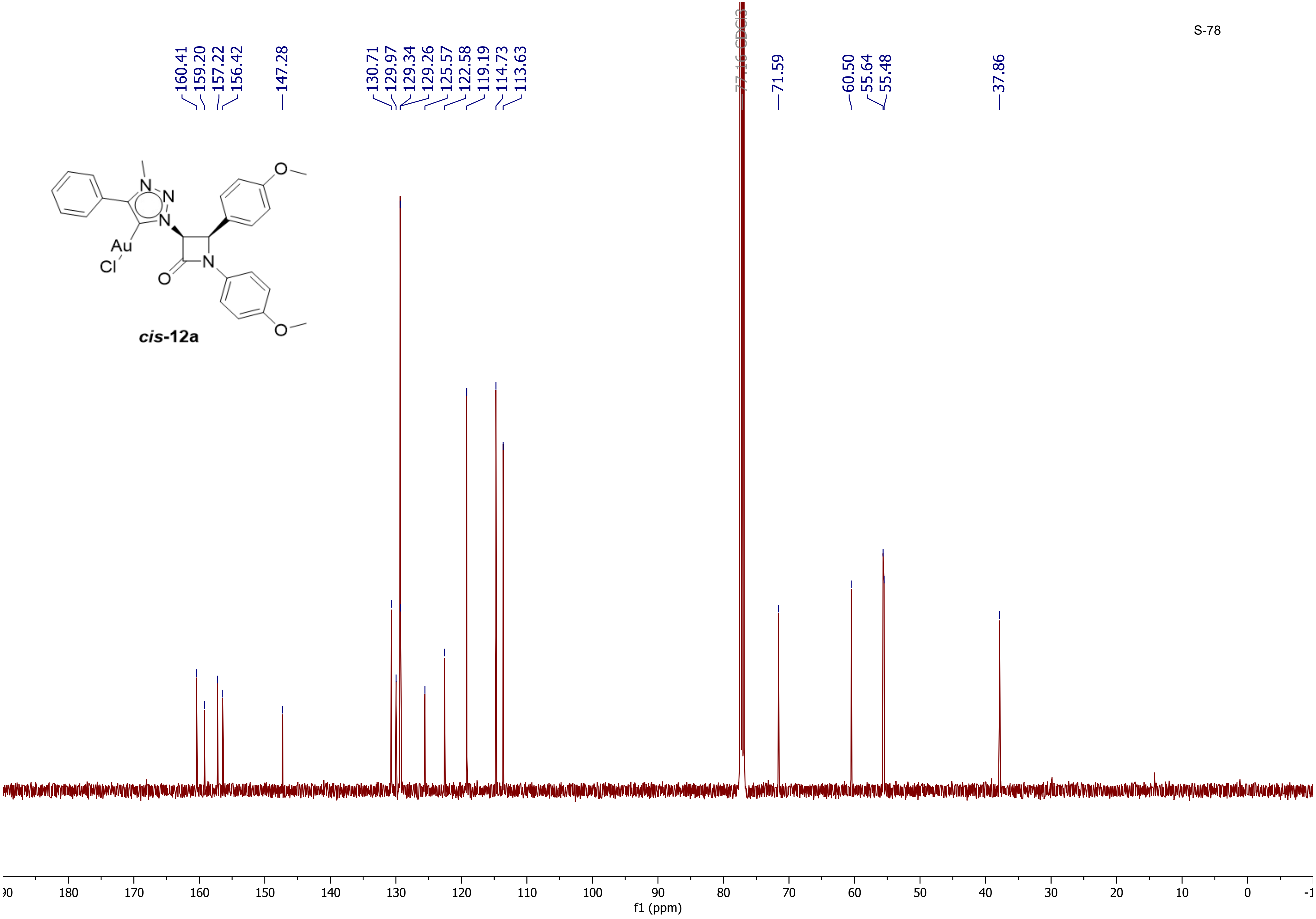
160.41
159.20
157.22
156.42
147.28
130.71
129.97
129.34
129.26
125.57
122.58
119.19
114.73
113.63
77.16 CDCl3
71.59
60.50
55.64
55.48
37.86
N
N
N
Au
Cl
O
N
O
O
cis-12a
190
180
170
160
150
140
130
120
110
100
90
80
70
60
50
40
30
20
10
0
-10
f1 (ppm)

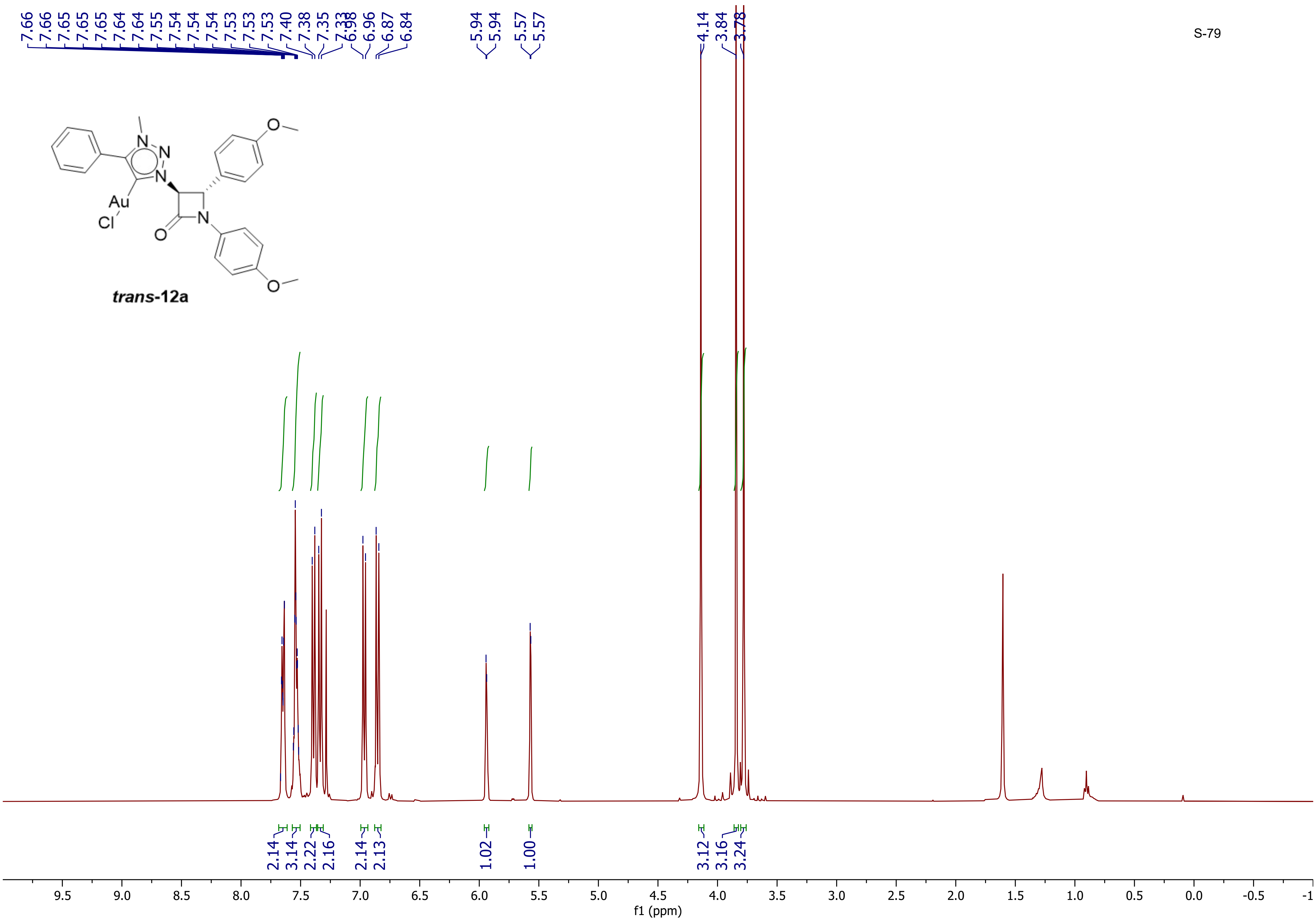
7.66
7.66
7.65
7.65
7.65
7.64
7.64
7.55
7.54
7.54
7.54
7.54
7.53
7.53
7.53
7.40
7.38
7.35
7.33
6.98
6.96
6.87
6.84
5.94
5.94
5.57
5.57
4.14
3.84
3.78
N
N
N
O
O
Au
Cl
N
O
trans-**12a**
2.14
3.14
2.22
2.16
2.14
2.13
1.02
1.00
3.12
3.16
3.24
9.5
9.0
8.5
8.0
7.5
7.0
6.5
6.0
5.5
5.0
4.5
4.0
3.5
3.0
2.5
2.0
1.5
1.0
0.5
0.0
-0.5
-1
f1 (ppm)

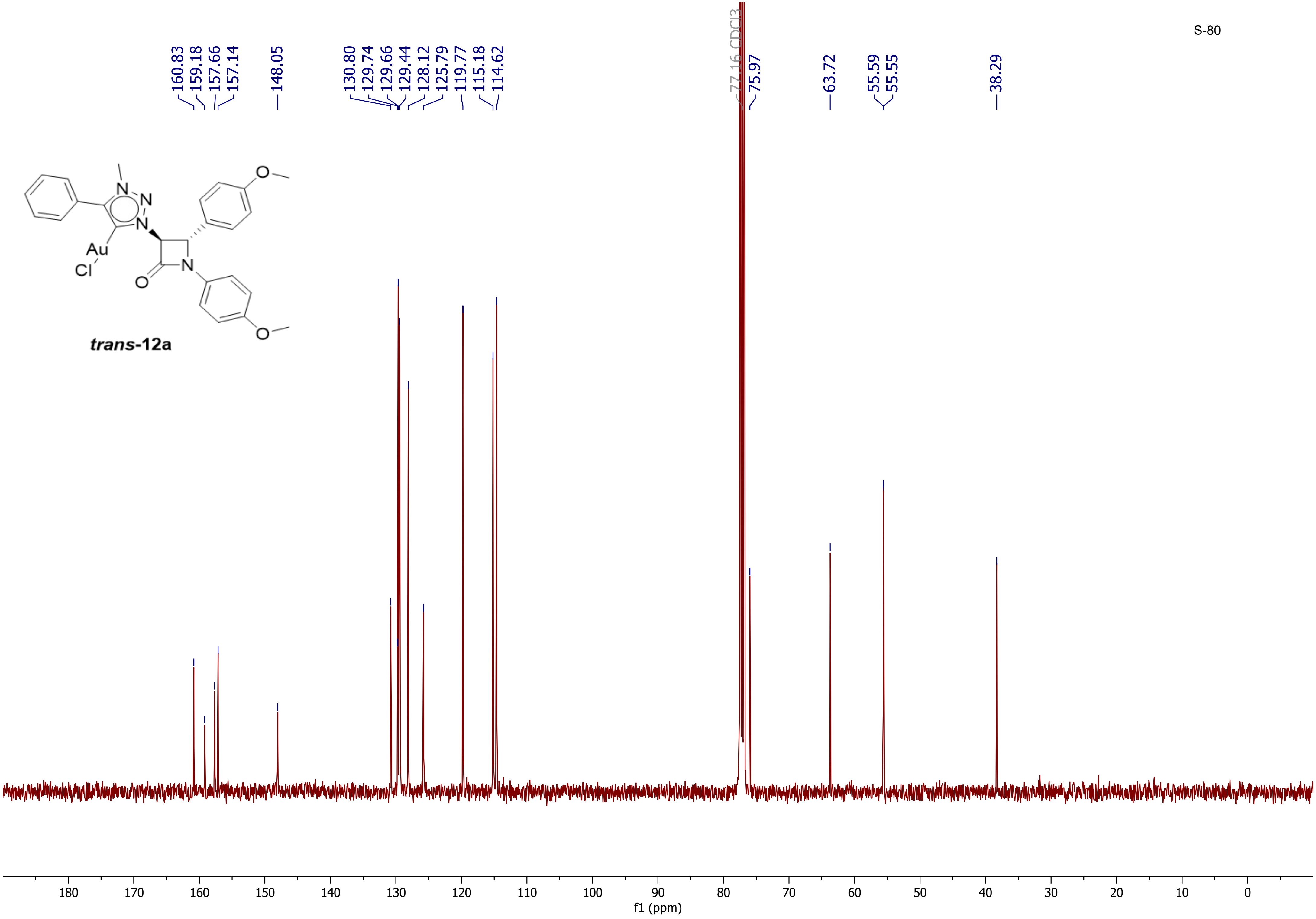

160.83
159.18
157.66
157.14
148.05
130.80
129.74
129.66
129.44
128.12
125.79
119.77
115.18
114.62
77.16 CDCl3
75.97
63.72
55.59
55.55
38.29
N
N
N
Au
Cl
O
N
O
O
180
170
160
150
140
130
120
110
100
90
80
70
60
50
40
30
20
10
0
f1 (ppm)

trans-**12a**

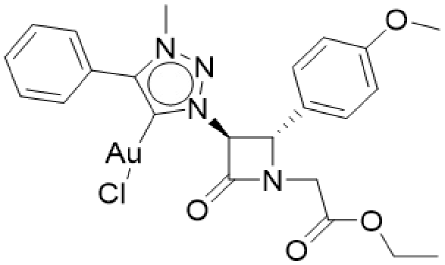

N
N
N
O
Au
Cl
N
O
O
O

***trans*-12c**

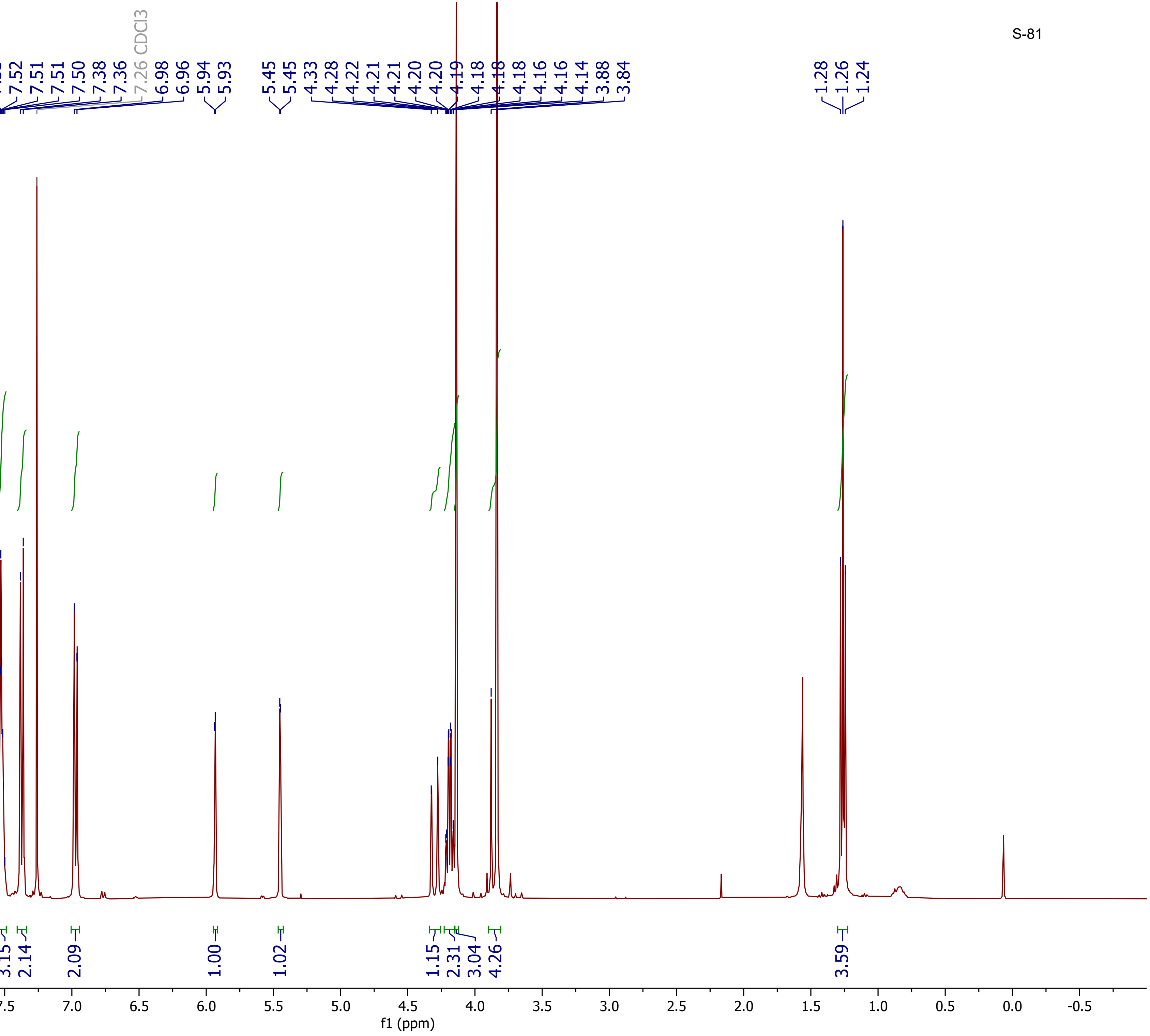

7.64
7.64
7.63
7.63
7.63
7.62
7.62
7.62
7.61
7.61
7.60
7.55
7.54
7.53
7.53
7.52
7.51
7.51
7.50
7.38
7.36
7.26 CDCl3
6.98
6.96
5.94
5.93
5.45
5.45
4.33
4.28
4.22
4.21
4.21
4.20
4.20
4.19
4.18
4.18
4.18
4.16
4.16
4.14
3.88
3.84
1.28
1.26
1.24
2.17
3.15
2.14
2.09
1.00
1.02
1.15
2.31
3.04
4.26
3.59
9.5
9.0
8.5
8.0
7.5
7.0
6.5
6.0
5.5
5.0
4.5
4.0
3.5
3.0
2.5
2.0
1.5
1.0
0.5
0.0
-0.5
f1 (ppm)

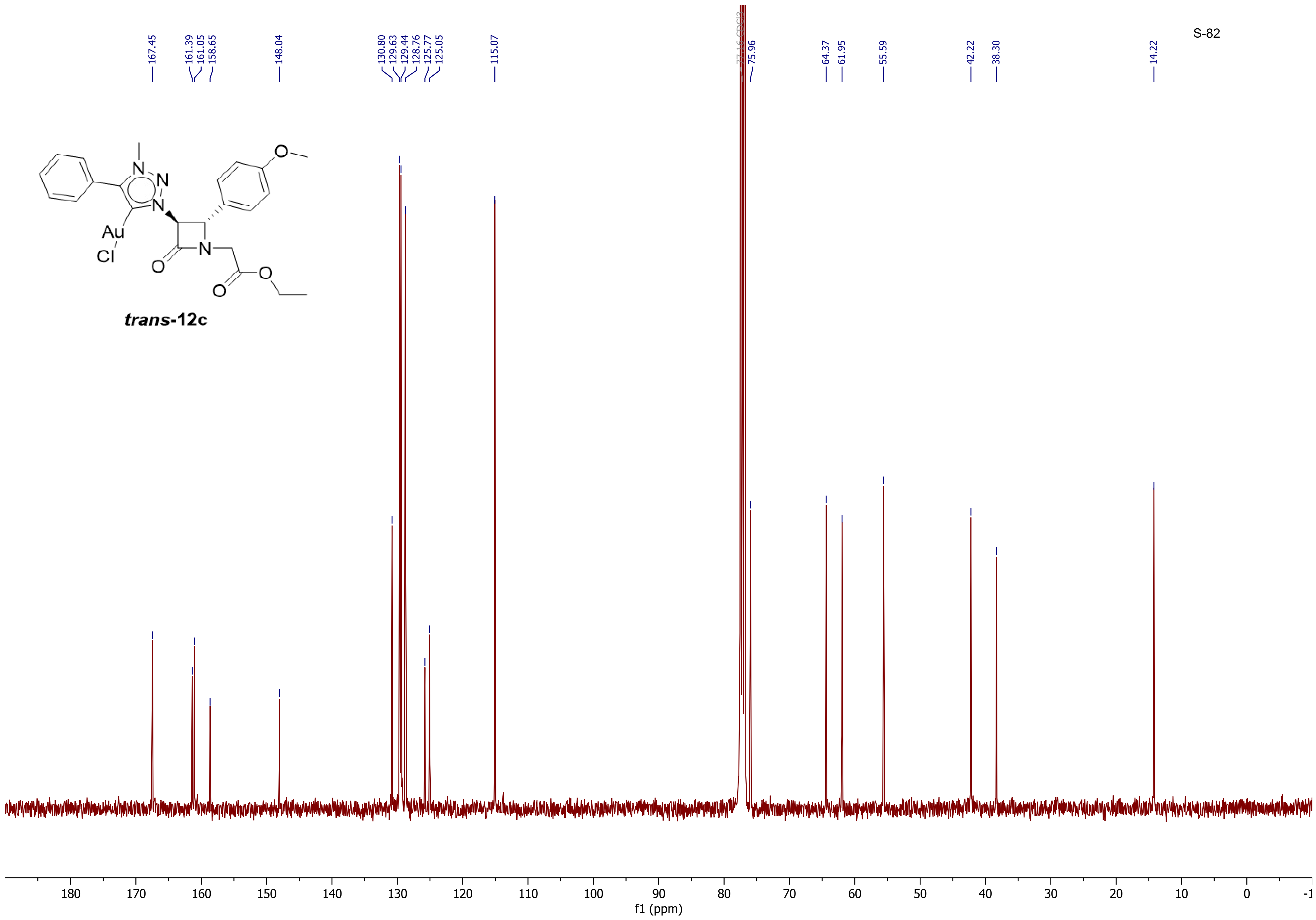
167.45
161.39
161.05
158.65
148.04
130.80
129.63
129.44
128.76
125.77
125.05
115.07
77.16 CDCl3
75.96
64.37
61.95
55.59
42.22
38.30
14.22
Au
Cl
N
N
N
O
O
N
O
O
trans-12c
180 170 160 150 140 130 120 110 100 90 80 70 60 50 40 30 20 10 0 -1
f1 (ppm)

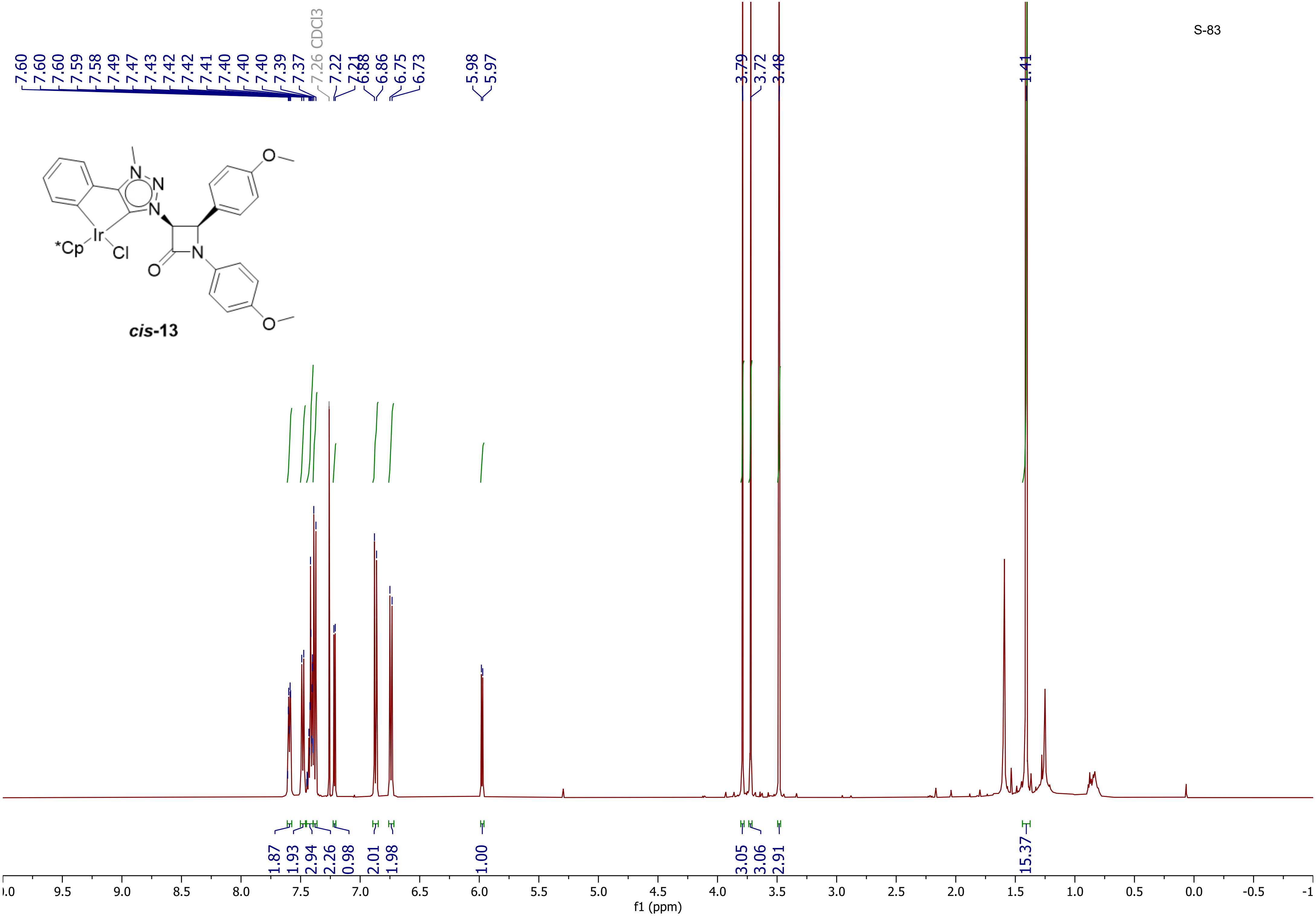
7.60
7.60
7.60
7.59
7.58
7.49
7.47
7.43
7.42
7.42
7.41
7.40
7.40
7.40
7.39
7.37
7.26 CDCl3
7.22
7.21
6.88
6.86
6.75
6.73
5.98
5.97
3.79
3.72
3.48
1.41
*Cp
Ir
Cl
N
N
N
N
O
O
O
cis-13
1.87
1.93
2.94
2.26
0.98
2.01
1.98
1.00
3.05
3.06
2.91
15.37
f1 (ppm)

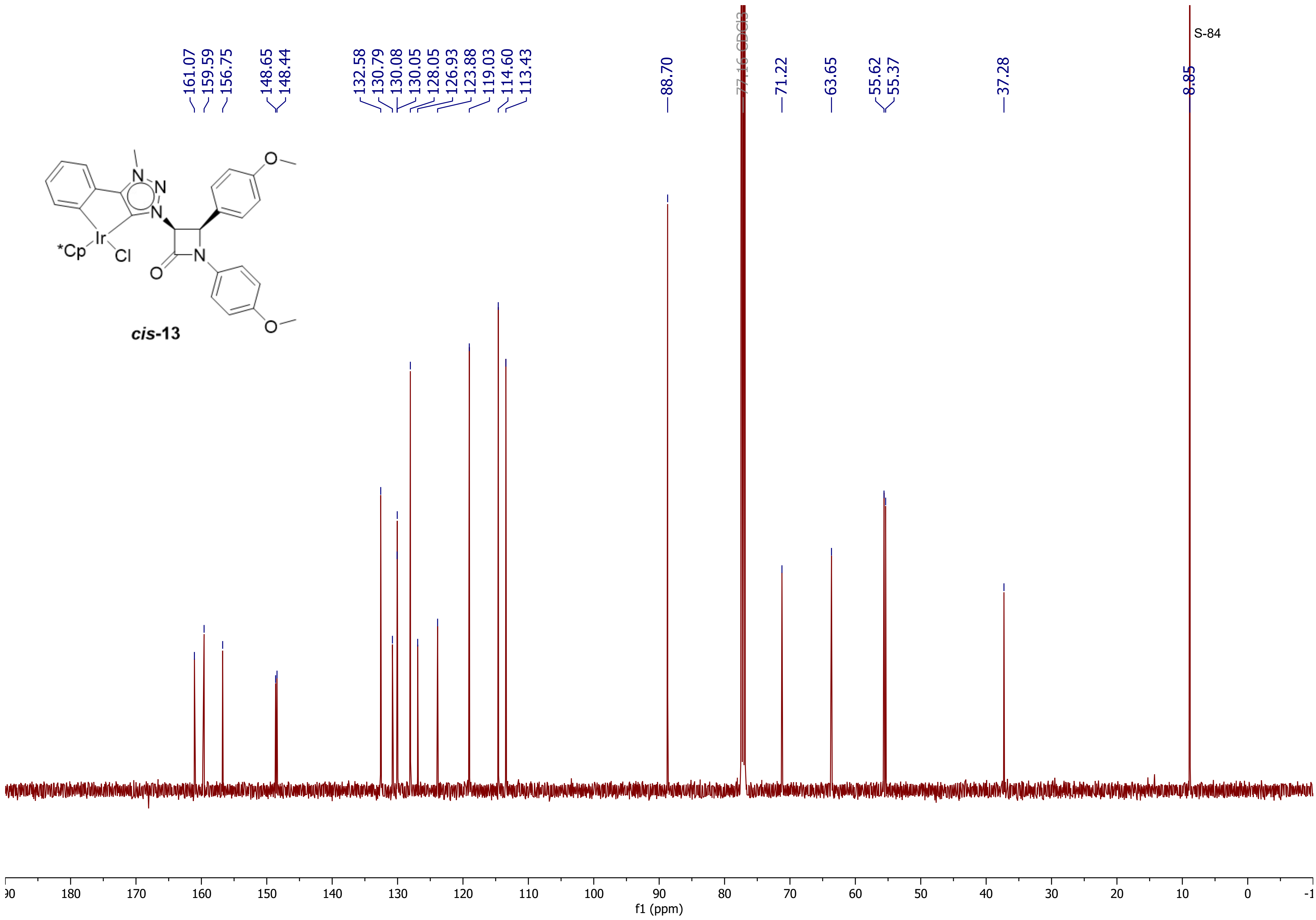
N
N
N
Ir
*Cp
Cl
O
N
O
O
cis-**13**
161.07
159.59
156.75
148.65
148.44
132.58
130.79
130.08
130.05
128.05
126.93
123.88
119.03
114.60
113.43
88.70
77.16 CDCl3
71.22
63.65
55.62
55.37
37.28
8.85
f1 (ppm)

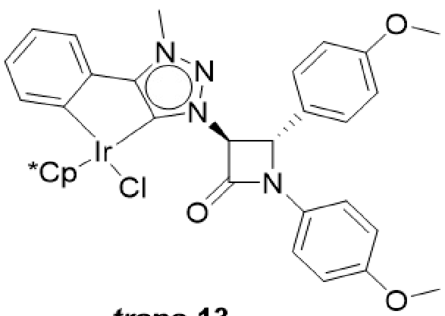

***trans*-13**

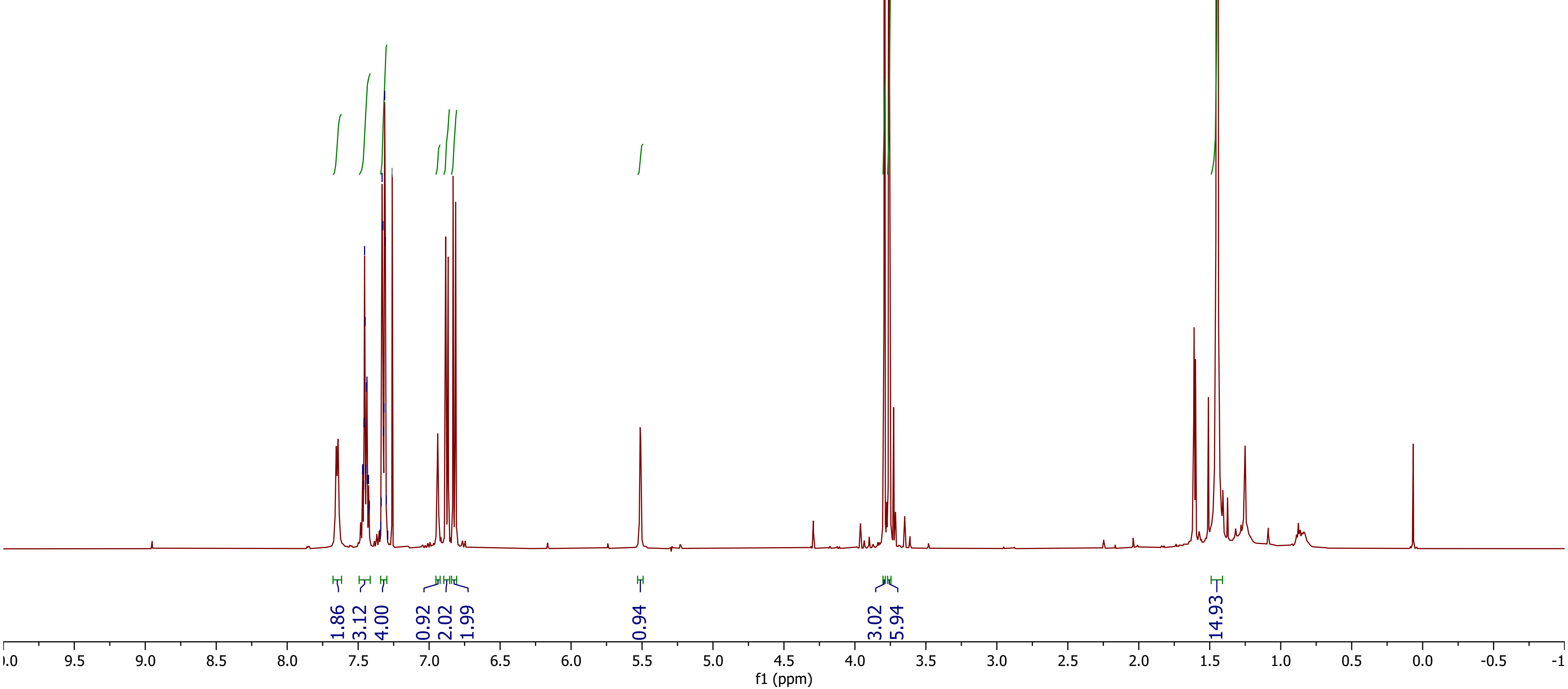
7.47
7.47
7.46
7.46
7.45
7.45
7.44
7.44
7.44
7.44
7.43
7.42
7.42
7.35
7.34
7.34
7.33
7.33
7.32
7.32
7.31
7.31
7.30
7.29
7.26 CDCl3
3.79
3.76
1.45
1.86
3.12
4.00
0.92
2.02
1.99
0.94
3.02
5.94
14.93
f1 (ppm)

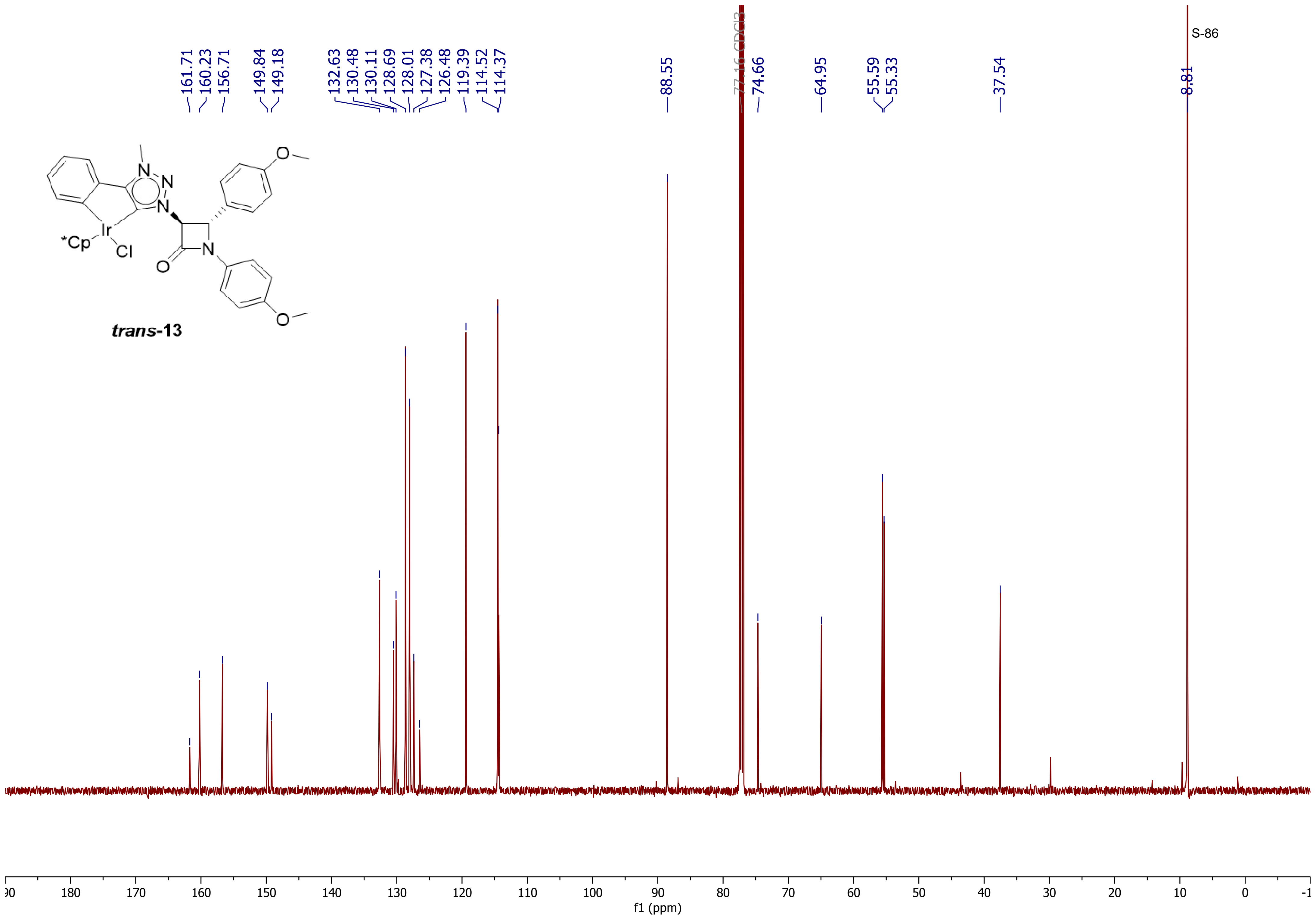
161.71
160.23
156.71
149.84
149.18
132.63
130.48
130.11
128.69
128.01
127.38
126.48
119.39
114.52
114.37
88.55
77.16 CDCl3
74.66
64.95
55.59
55.33
37.54
8.81
*Cp
Ir
Cl
N
N
N
N
O
O
O
trans-13
190
180
170
160
150
140
130
120
110
100
90
80
70
60
50
40
30
20
10
0
-10
f1 (ppm)

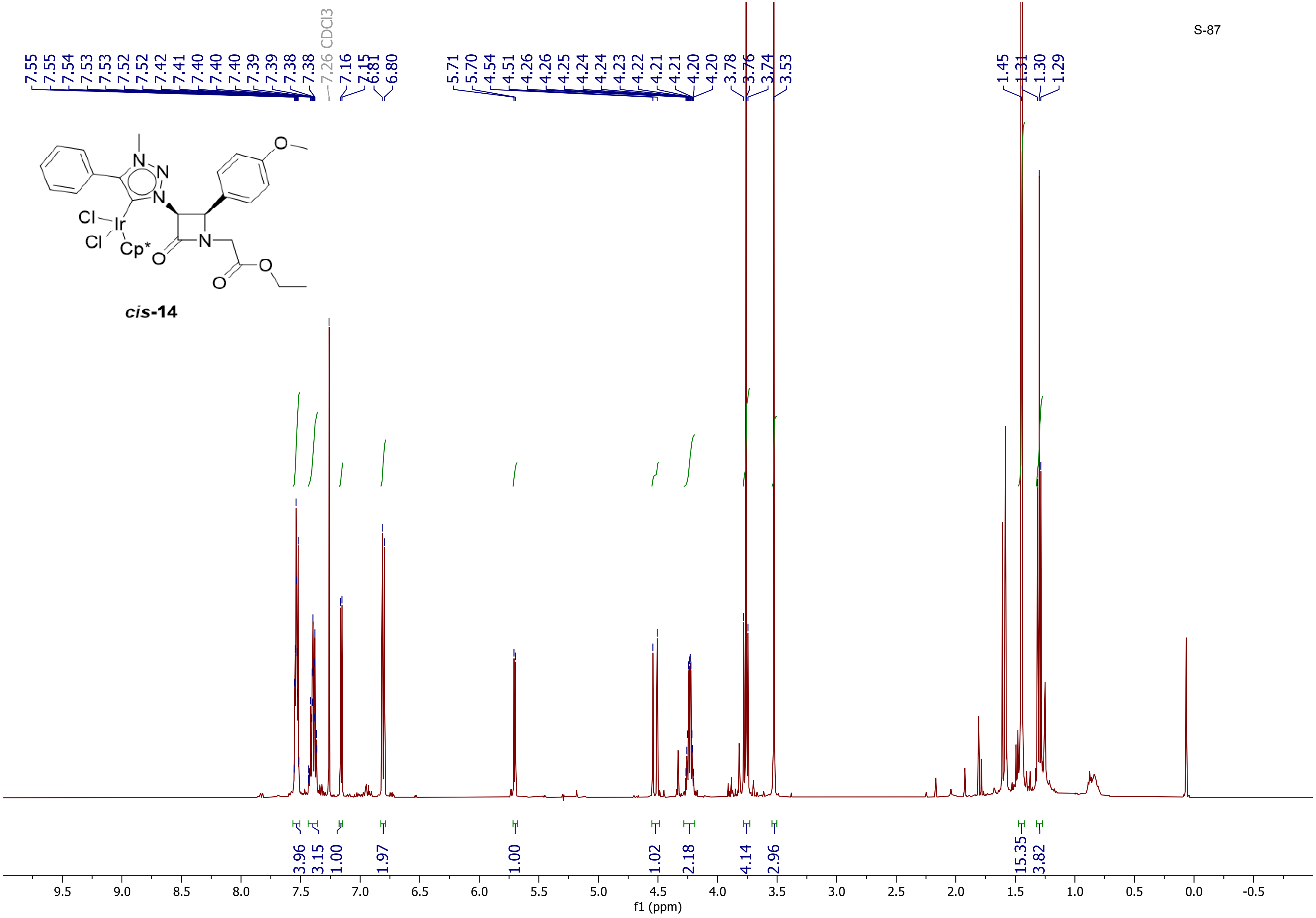
7.55 7.55 7.54 7.53 7.53 7.52 7.52 7.42 7.41 7.40 7.40 7.40 7.39 7.39 7.38 7.38 7.26 CDCl3 7.16 7.15 6.81 6.80
5.71 5.70 4.54 4.51 4.26 4.26 4.25 4.24 4.24 4.23 4.22 4.21 4.21 4.20 4.20 3.78 3.76 3.74 3.53
1.45 1.31 1.30 1.29
Cl
Ir
Cl
Cp*
N
N
N
O
N
O
O
O
cis-14
3.96 3.15 1.00 1.97 1.00 1.02 2.18 4.14 2.96 15.35 3.82
9.5 9.0 8.5 8.0 7.5 7.0 6.5 6.0 5.5 5.0 4.5 4.0 3.5 3.0 2.5 2.0 1.5 1.0 0.5 0.0 -0.5
f1 (ppm)

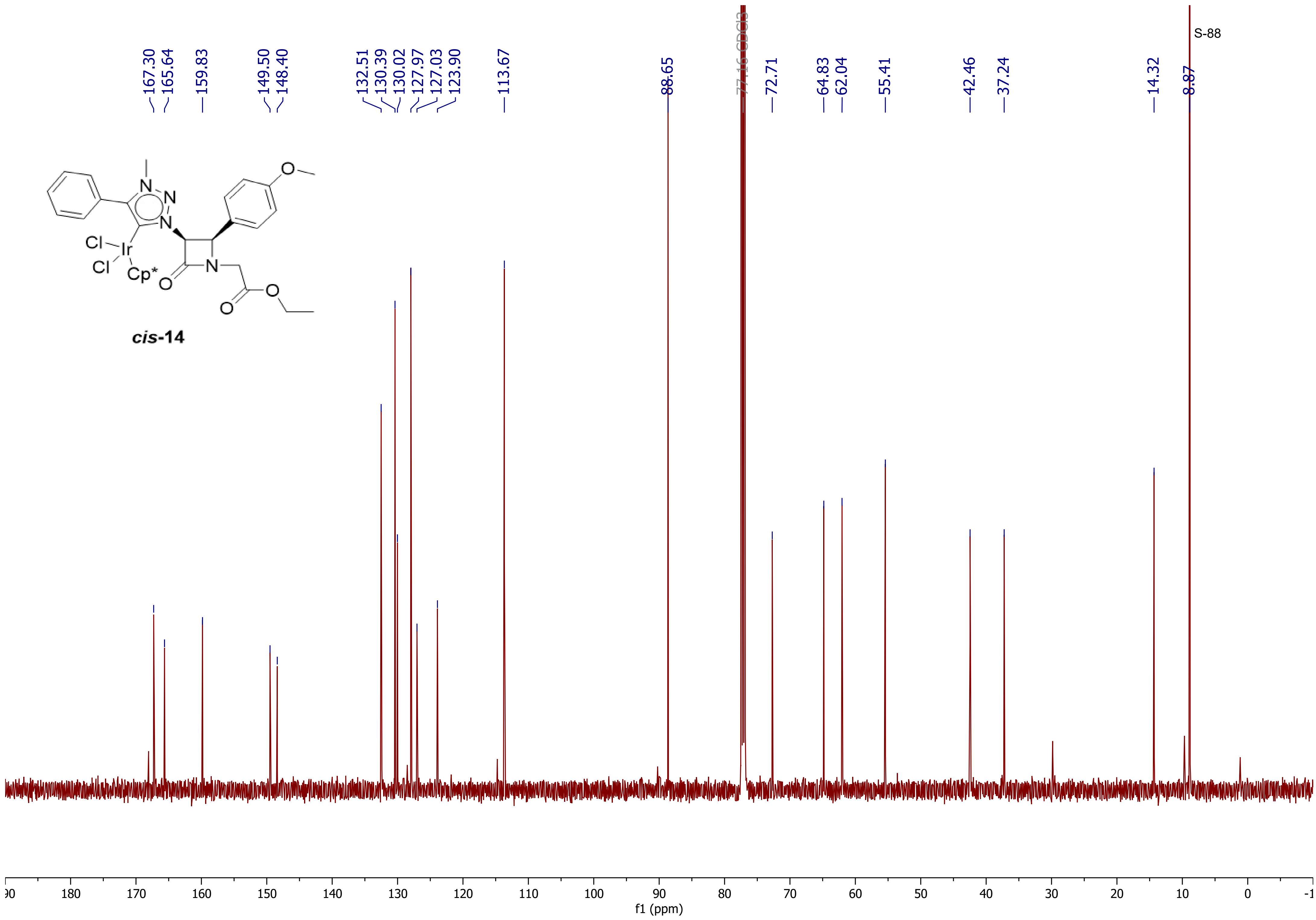
167.30
165.64
159.83
149.50
148.40
132.51
130.39
130.02
127.97
127.03
123.90
113.67
88.65
77.16 CDCl3
72.71
64.83
62.04
55.41
42.46
37.24
14.32
8.87
Cl
Ir
Cl
Cp*
N
N
N
O
N
O
O
O
O
cis-14
190
180
170
160
150
140
130
120
110
100
90
80
70
60
50
40
30
20
10
0
-10
f1 (ppm)

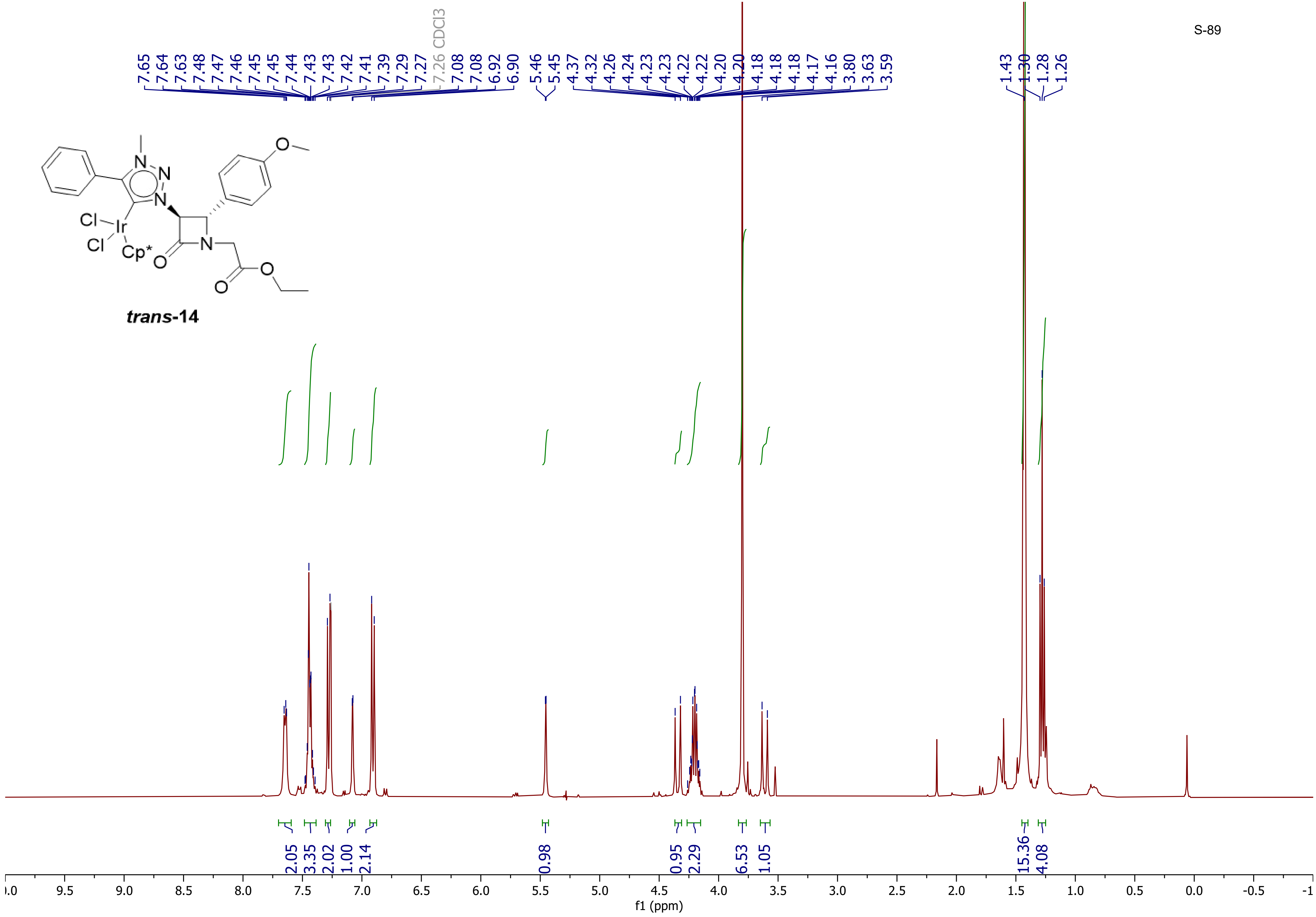
7.65 7.64 7.63 7.48 7.47 7.46 7.45 7.45 7.44 7.43 7.43 7.42 7.41 7.39 7.29 7.27 7.26 CDCl3 7.08 7.08 7.08 6.92 6.90 5.46 5.45 4.37 4.32 4.26 4.24 4.23 4.23 4.22 4.22 4.20 4.20 4.18 4.18 4.18 4.17 4.16 3.80 3.63 3.59 1.43 1.30 1.28 1.26
Cl
Cl
Ir
Cp*
N
N
N
O
N
O
O
O
trans-14
2.05 3.35 2.02 1.00 2.14 0.98 0.95 2.29 6.53 1.05 15.36 4.08
10.0 9.5 9.0 8.5 8.0 7.5 7.0 6.5 6.0 5.5 5.0 4.5 4.0 3.5 3.0 2.5 2.0 1.5 1.0 0.5 0.0 -0.5 -1
f1 (ppm)

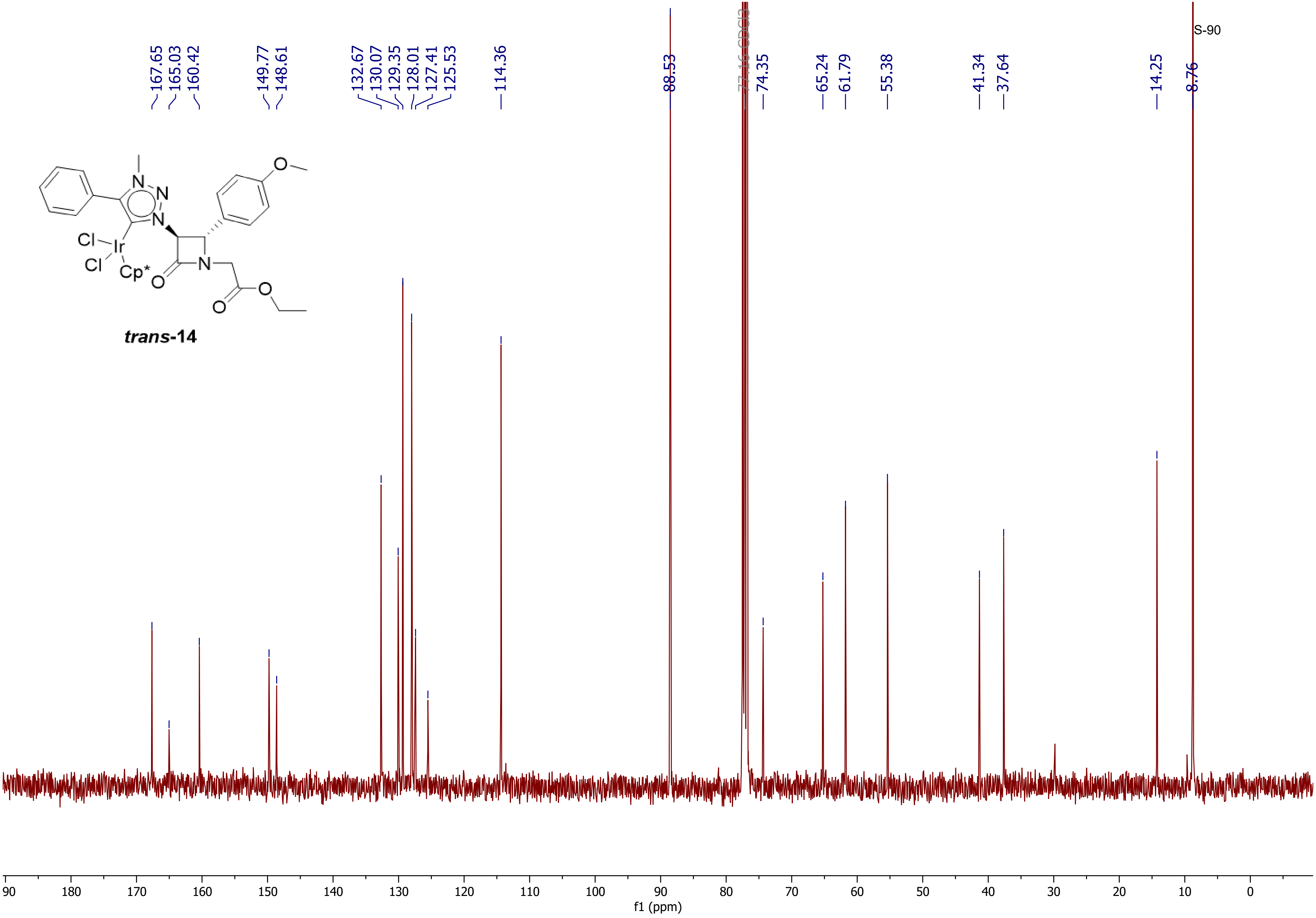

167.65
165.03
160.42
149.77
148.61
132.67
130.07
129.35
128.01
127.41
125.53
114.36
88.53
77.16 CDCl3
74.35
65.24
61.79
55.38
41.34
37.64
14.25
8.76
Cl
Cl
Ir
Cp*
N
N
N
O
N
O
O
O
190 180 170 160 150 140 130 120 110 100 90 80 70 60 50 40 30 20 10 0
f1 (ppm)

***trans*-14**

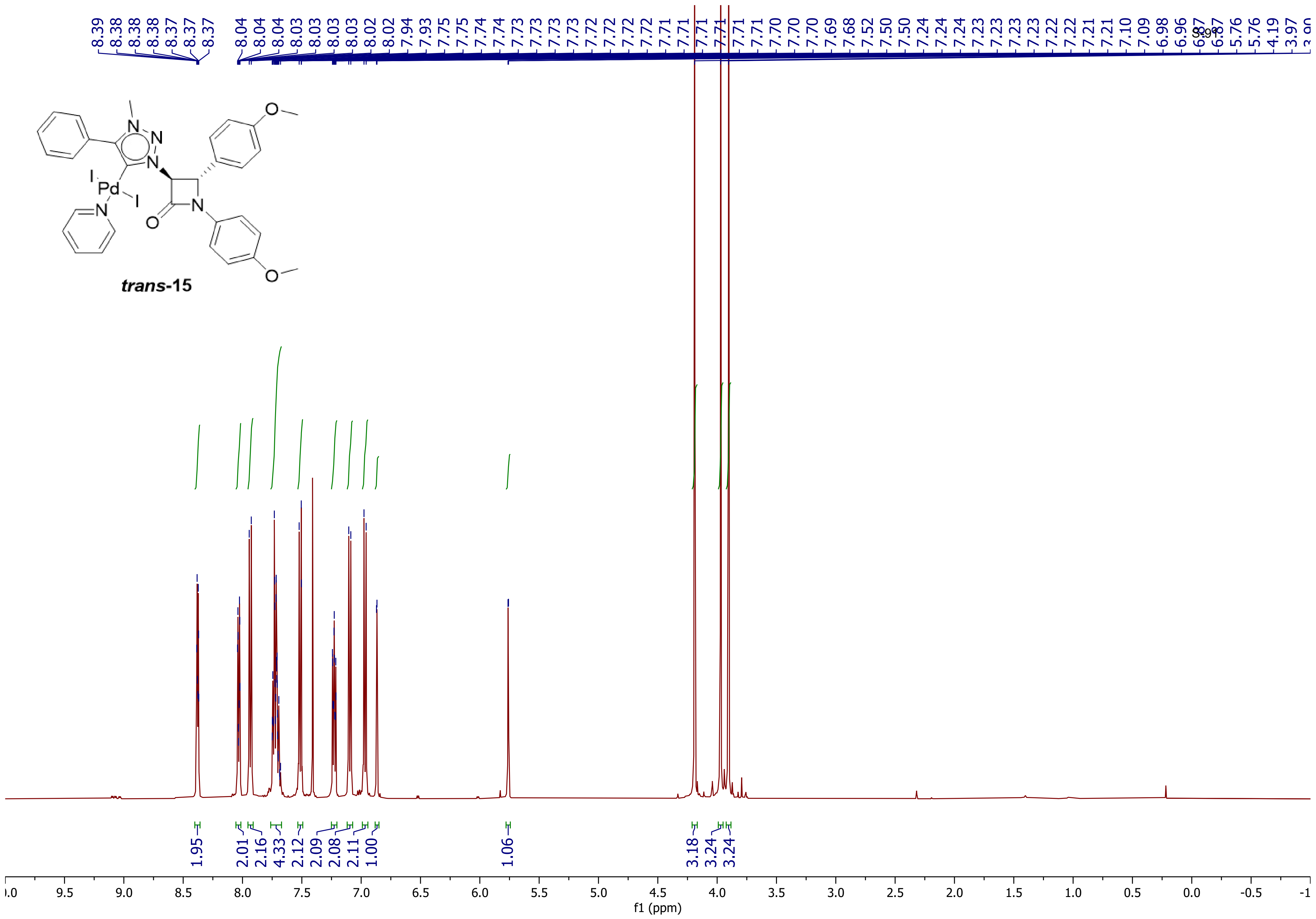

8.39 8.38 8.38 8.38 8.37 8.37 8.37 8.04 8.04 8.04 8.03 8.03 8.03 8.03 8.02 8.02 7.94 7.93 7.75 7.75 7.74 7.74 7.73 7.73 7.73 7.73 7.72 7.72 7.72 7.71 7.71 7.71 7.71 7.71 7.71 7.70 7.70 7.70 7.69 7.68 7.52 7.50 7.50 7.24 7.24 7.24 7.23 7.23 7.23 7.23 7.22 7.22 7.21 7.21 7.10 7.09 6.98 6.96 6.87 5.76 5.76 4.19 3.97
trans-15
1.95 2.01 2.16 4.33 2.12 2.09 2.08 2.11 1.00 1.06 3.18 3.24 3.24
9.5 9.0 8.5 8.0 7.5 7.0 6.5 6.0 5.5 5.0 4.5 4.0 3.5 3.0 2.5 2.0 1.5 1.0 0.5 0.0 -0.5
f1 (ppm)

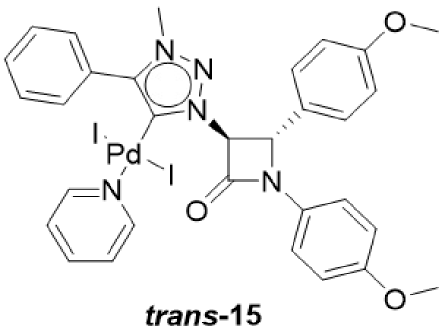
N
N
N
I
Pd
I
N
O
N
O
O

trans-**15**

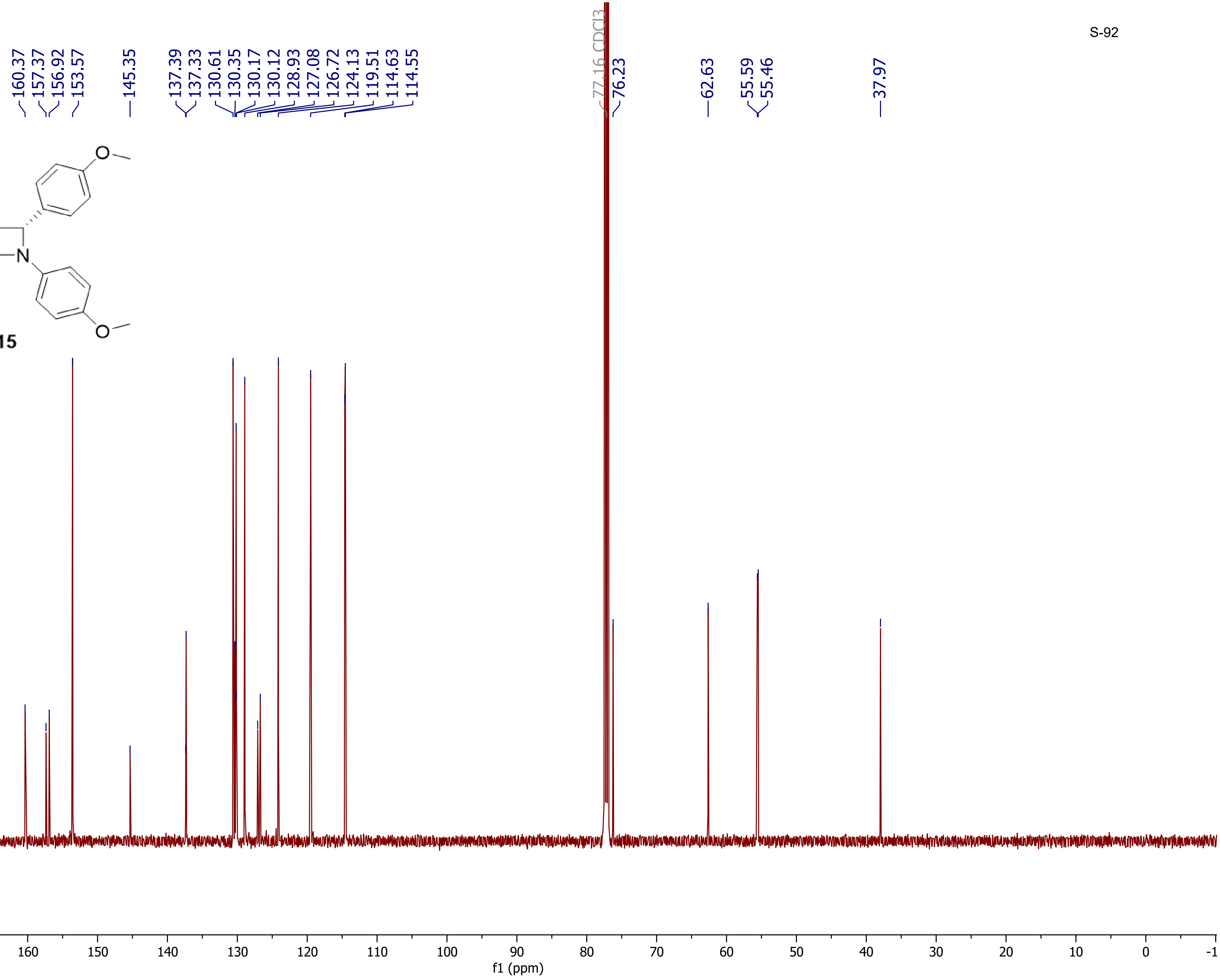
160.37
157.37
156.92
153.57
145.35
137.39
137.33
130.61
130.35
130.17
130.12
128.93
127.08
126.72
124.13
119.51
114.63
114.55
77.16 CDCl3
76.23
62.63
55.59
55.46
37.97
180
170
160
150
140
130
120
110
100
90
80
70
60
50
40
30
20
10
0
-1
f1 (ppm)

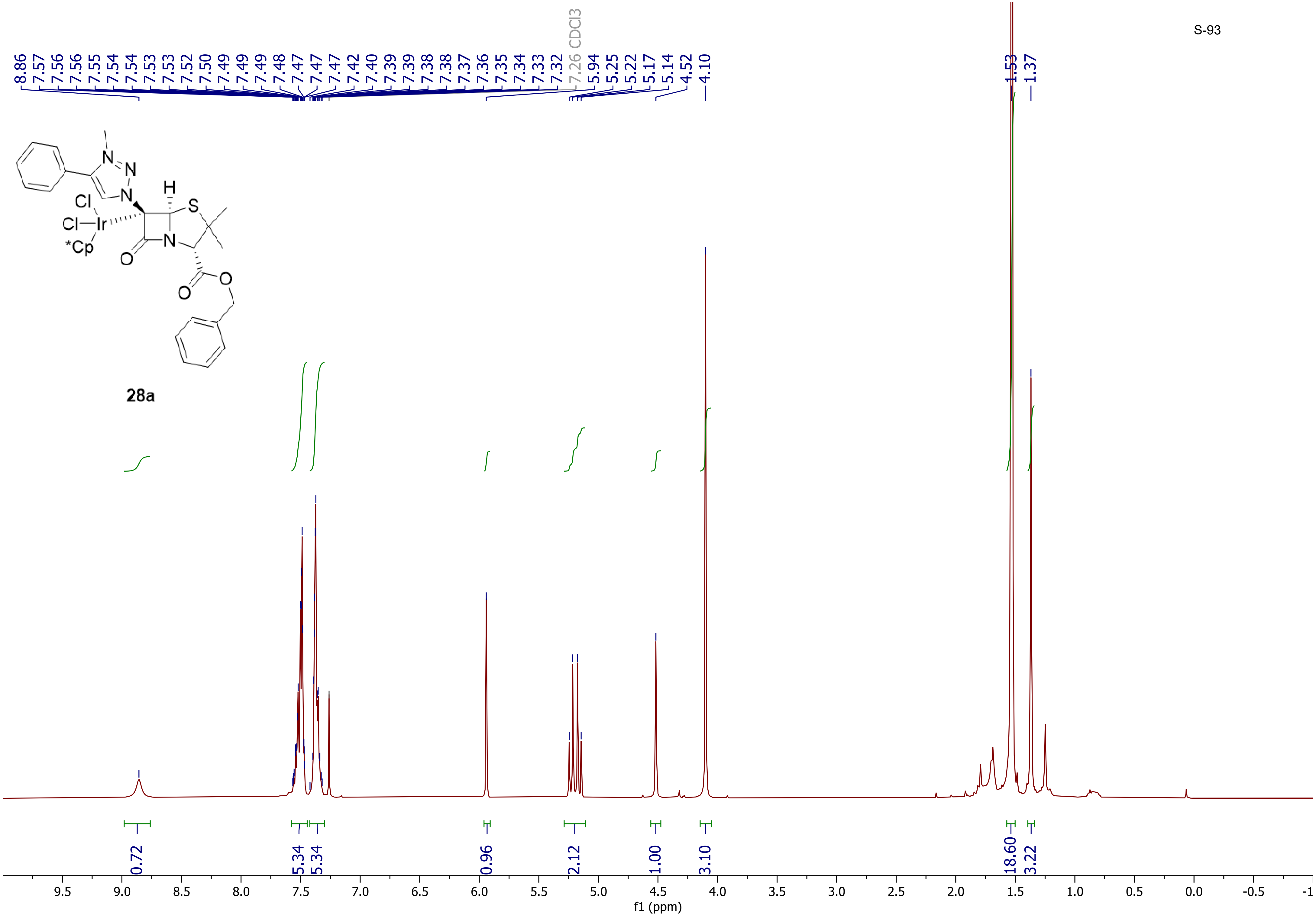
8.86
7.57
7.56
7.56
7.55
7.54
7.54
7.53
7.53
7.52
7.50
7.49
7.49
7.49
7.48
7.47
7.47
7.47
7.42
7.40
7.39
7.39
7.38
7.38
7.37
7.36
7.35
7.34
7.33
7.32
7.26 CDCl3
5.94
5.25
5.22
5.17
5.14
4.52
4.10
1.53
1.37
Cl
Cl—Ir
*Cp
N
N
N
H
S
N
O
O
O
28a
0.72
5.34
5.34
0.96
2.12
1.00
3.10
18.60
3.22
9.5
9.0
8.5
8.0
7.5
7.0
6.5
6.0
5.5
5.0
4.5
4.0
3.5
3.0
2.5
2.0
1.5
1.0
0.5
0.0
-0.5
-1
f1 (ppm)

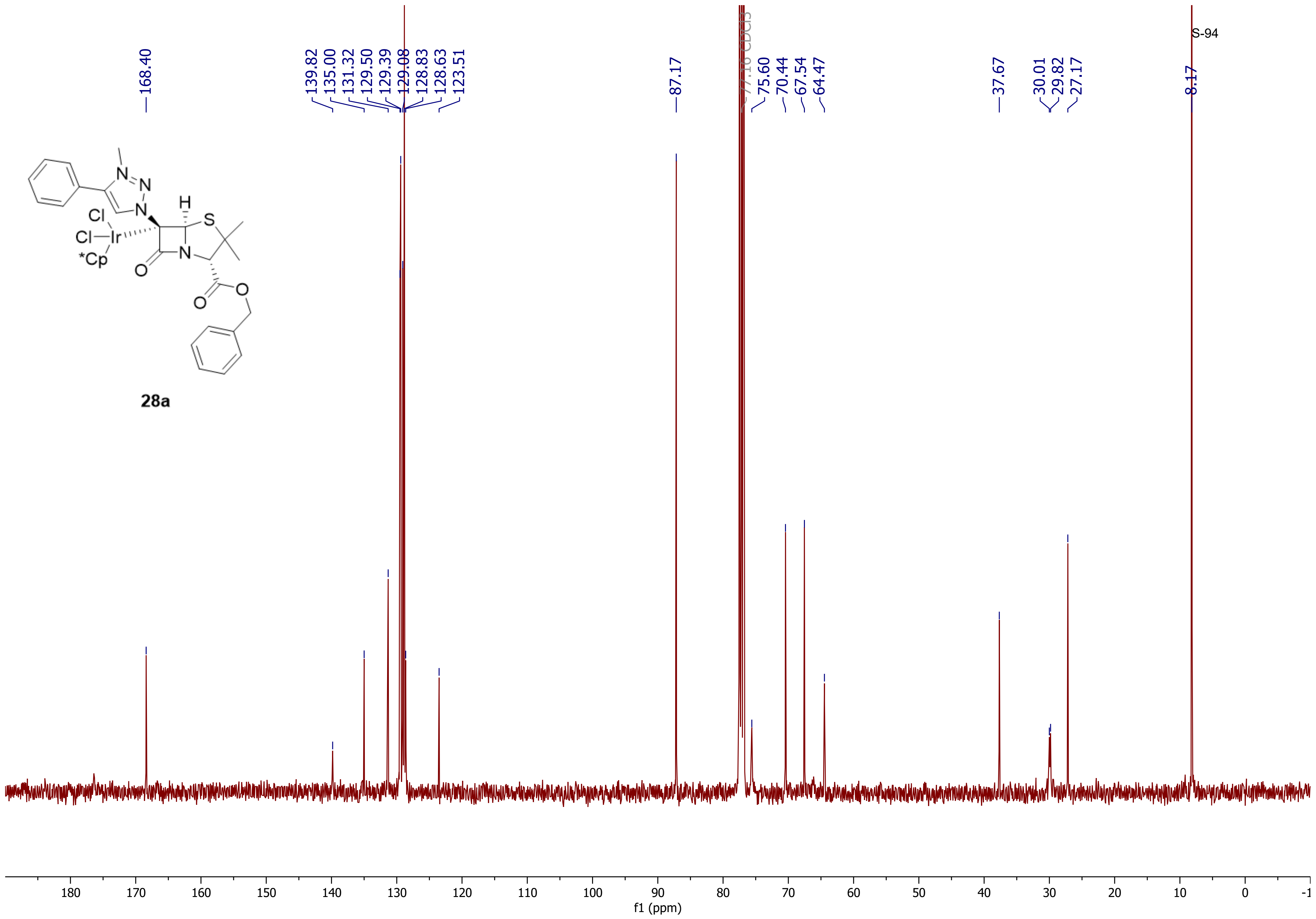
168.40
139.82
135.00
131.32
129.50
129.39
129.08
128.83
128.63
123.51
87.17
77.16 CDCl3
75.60
70.44
67.54
64.47
37.67
30.01
29.82
27.17
8.17
Cl
Cl
Ir
*Cp
N
N
N
H
S
N
O
O
O
28a
180 170 160 150 140 130 120 110 100 90 80 70 60 50 40 30 20 10 0 -1
f1 (ppm)

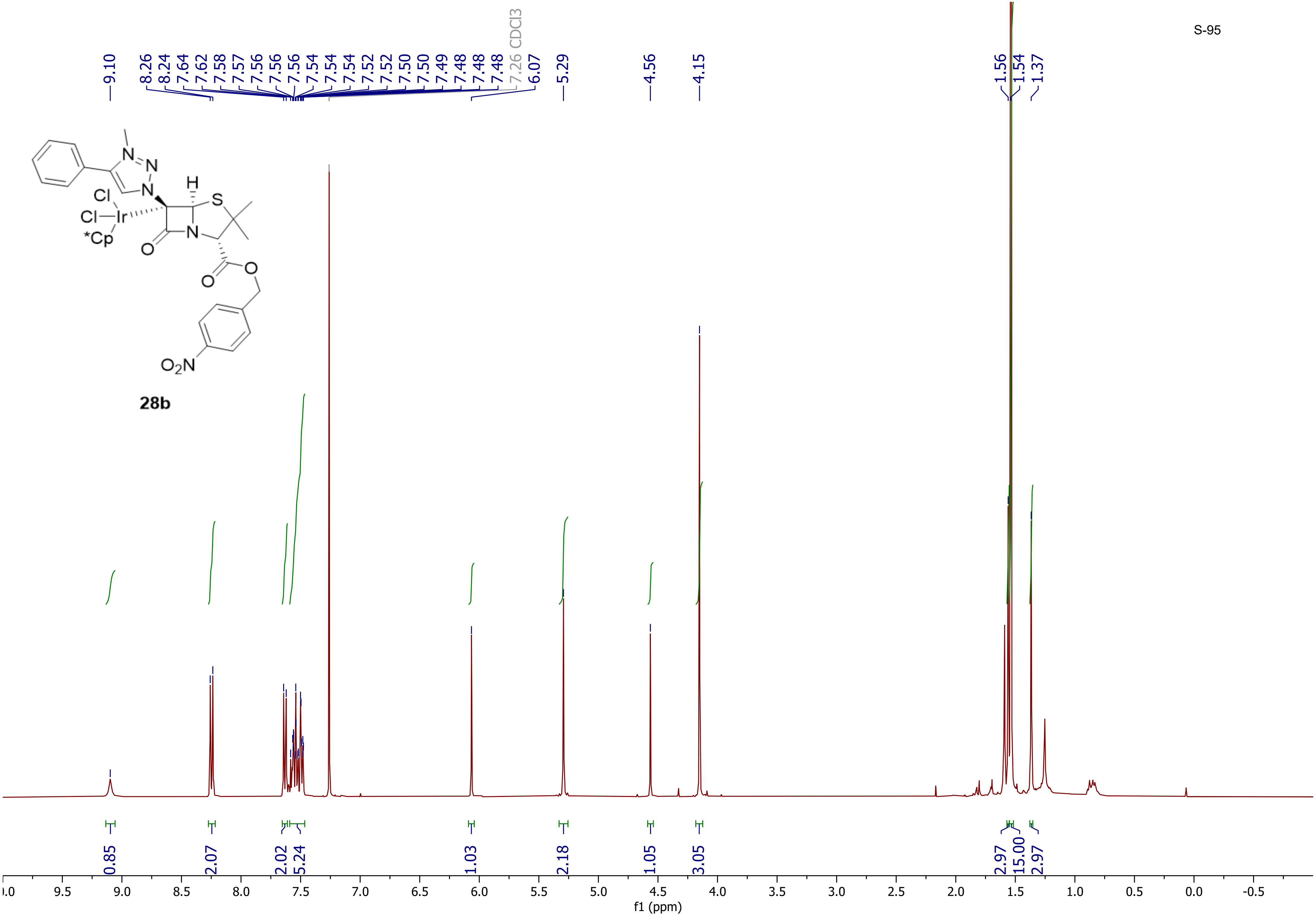
9.10
8.26
8.24
7.64
7.62
7.58
7.57
7.56
7.56
7.56
7.54
7.54
7.54
7.52
7.52
7.50
7.50
7.49
7.48
7.48
7.48
7.26 CDCl3
6.07
5.29
4.56
4.15
1.56
1.54
1.37
0.85
2.07
2.02
5.24
1.03
2.18
1.05
3.05
2.97
15.00
2.97
f1 (ppm)

28b

MML829B-13c
STANDARD PROTON PARAMETERS

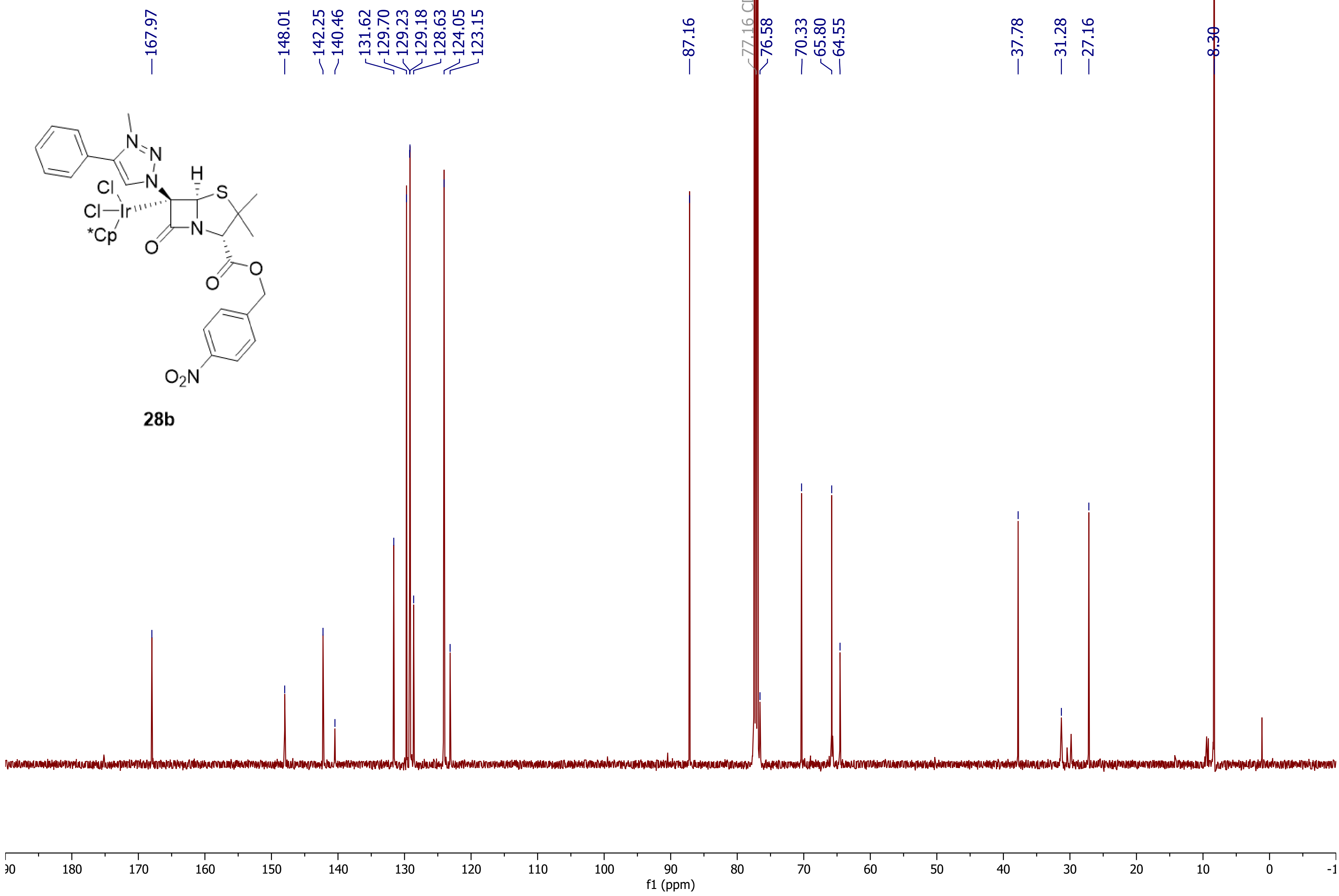

28b

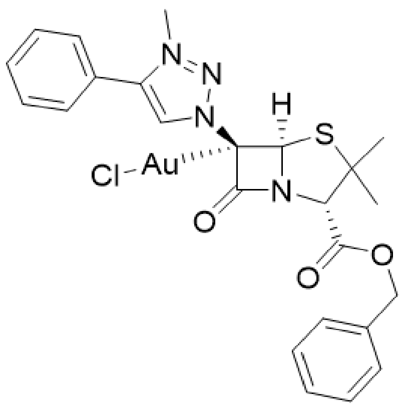

Cl
Au
N
N
N
H
S
N
O
O
O

30

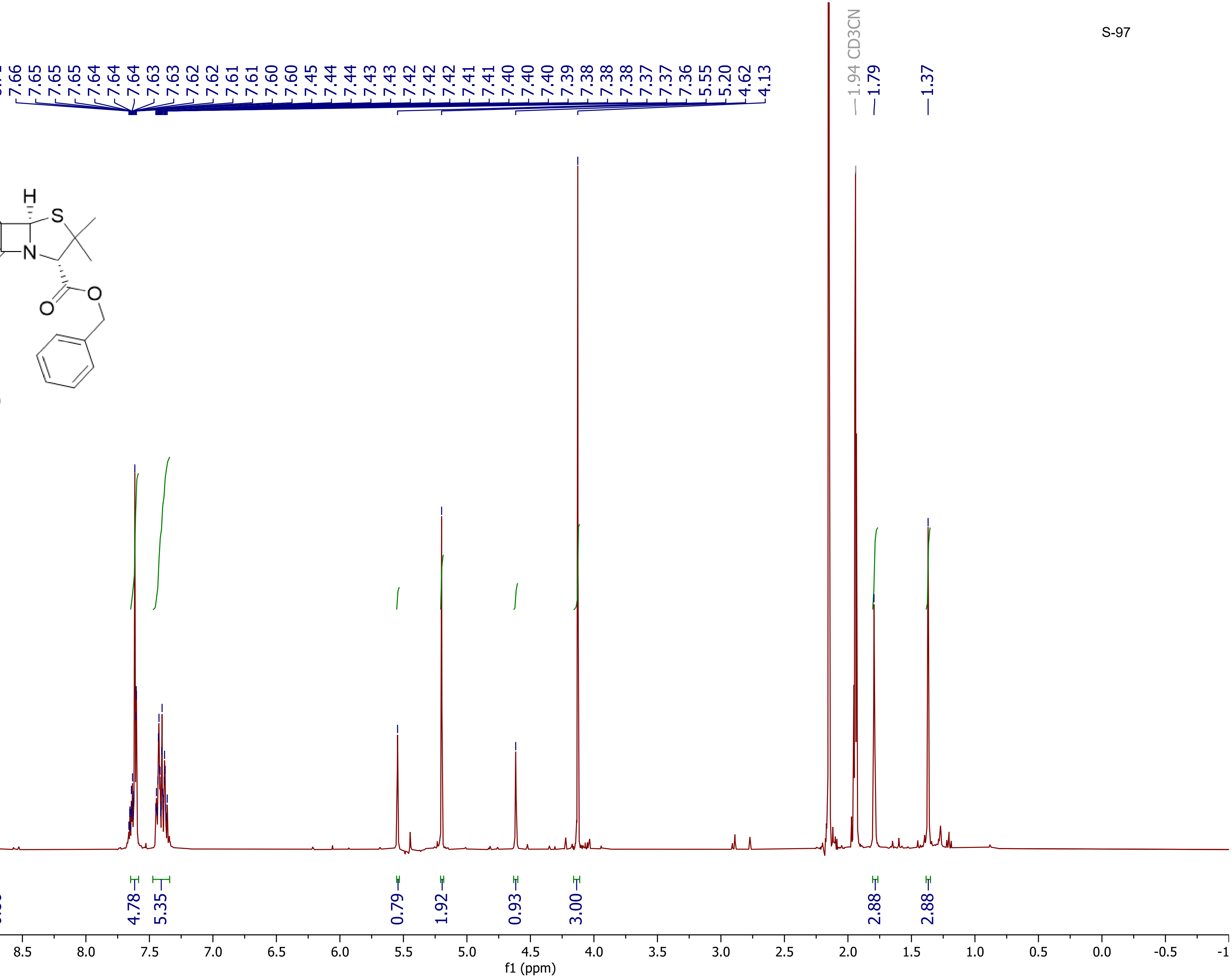

8.71
7.66
7.65
7.65
7.65
7.64
7.64
7.64
7.63
7.63
7.62
7.62
7.61
7.61
7.60
7.60
7.45
7.44
7.44
7.43
7.43
7.42
7.42
7.42
7.41
7.41
7.40
7.40
7.40
7.39
7.38
7.38
7.38
7.37
7.37
7.36
5.55
5.20
4.62
4.13
1.94 CD3CN
1.79
1.37
0.86
4.78
5.35
0.79
1.92
0.93
3.00
2.88
2.88
f1 (ppm)

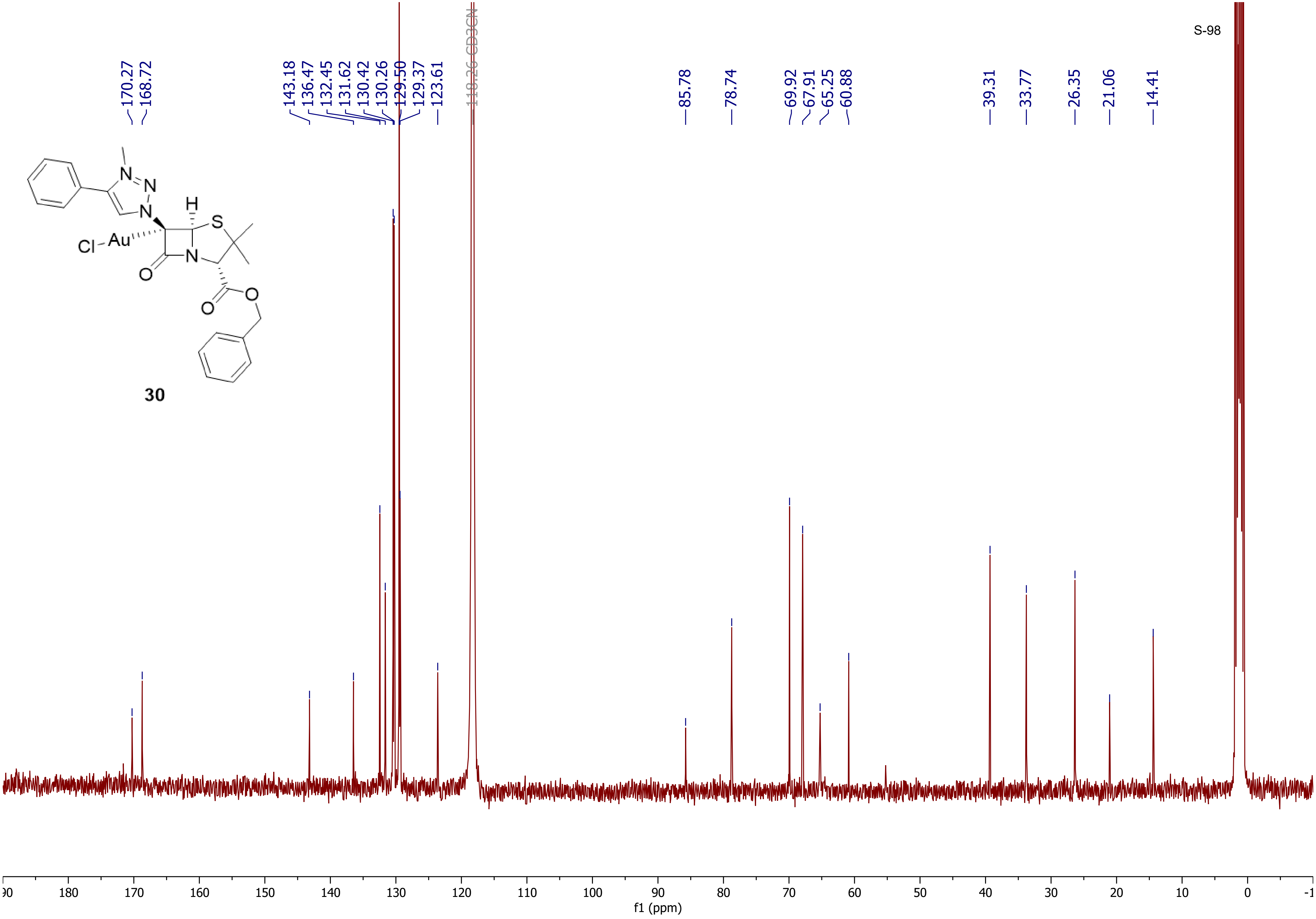
170.27
168.72
143.18
136.47
132.45
131.62
130.42
130.26
129.50
129.37
123.61
118.26 CD3CN
85.78
78.74
69.92
67.91
65.25
60.88
39.31
33.77
26.35
21.06
14.41
f1 (ppm)
30

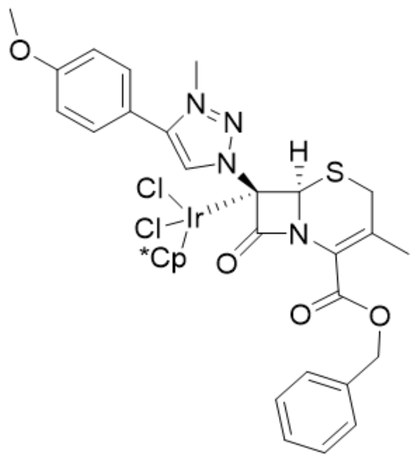

29a

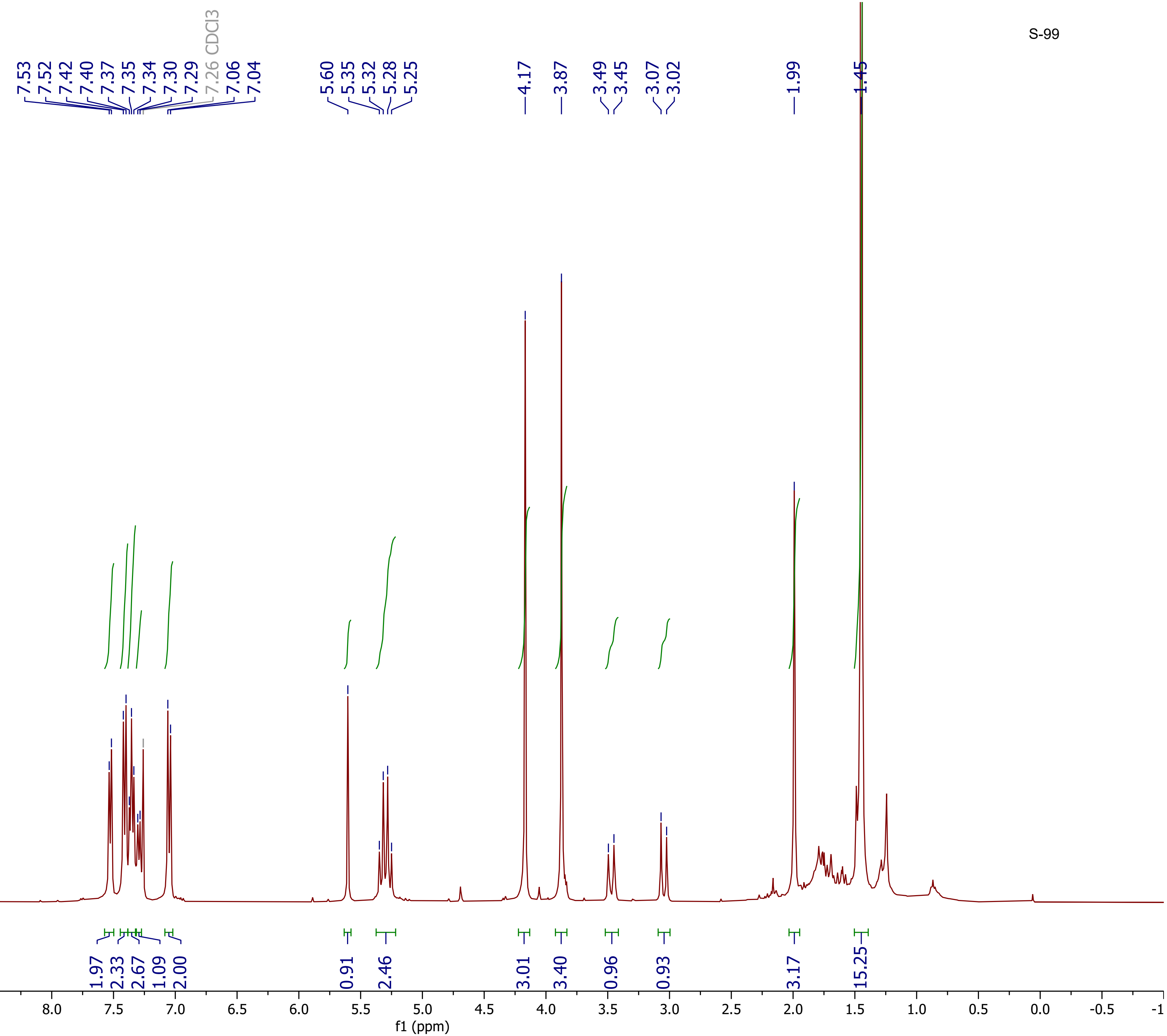

9.46
7.53
7.52
7.42
7.40
7.37
7.35
7.34
7.30
7.29
7.26 CDCl3
7.06
7.04
5.60
5.35
5.32
5.28
5.25
4.17
3.87
3.49
3.45
3.07
3.02
1.99
1.45
0.89
1.97
2.33
2.67
1.09
2.00
0.91
2.46
3.01
3.40
0.96
0.93
3.17
15.25
f1 (ppm)

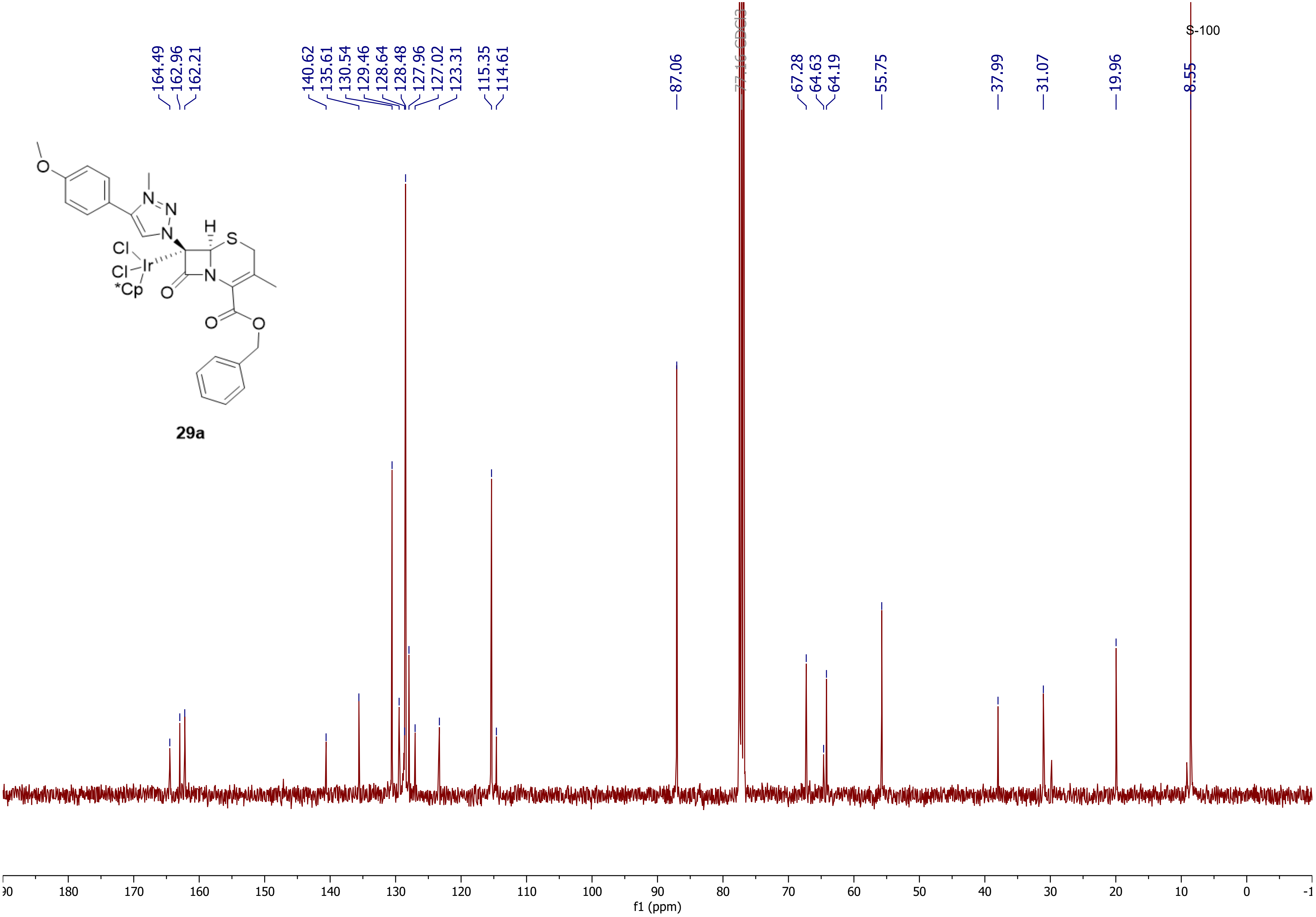
164.49
162.96
162.21
140.62
135.61
130.54
129.46
128.64
128.48
127.96
127.02
123.31
115.35
114.61
87.06
77.16 CDCl3
67.28
64.63
64.19
55.75
37.99
31.07
19.96
8.55
29a
f1 (ppm)
190 180 170 160 150 140 130 120 110 100 90 80 70 60 50 40 30 20 10 0 -10

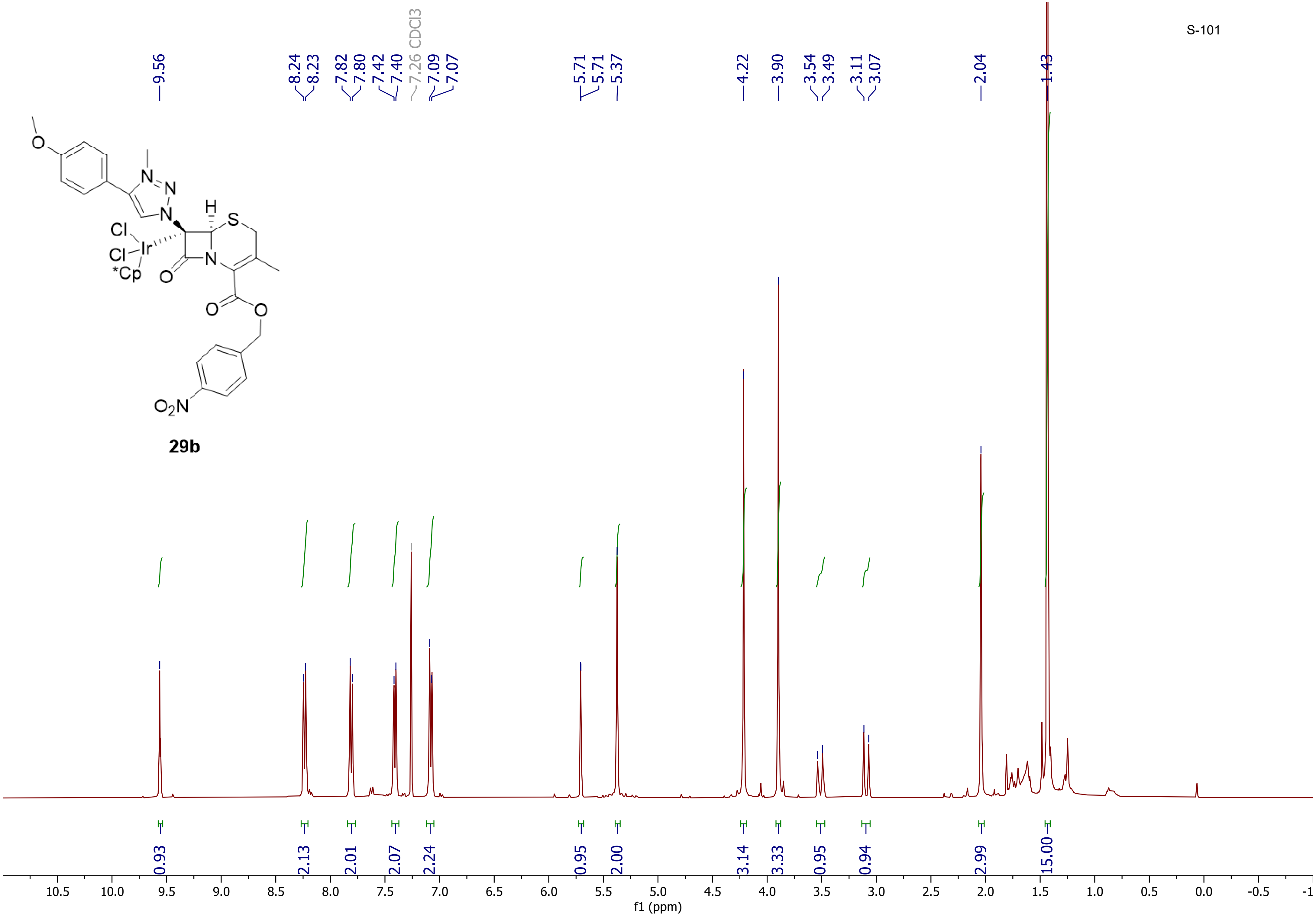

9.56
8.24
8.23
7.82
7.80
7.42
7.40
7.26 CDCl3
7.09
7.07
5.71
5.71
5.37
4.22
3.90
3.54
3.49
3.11
3.07
2.04
1.43
O
N
N
N
H
S
Cl
Ir
Cl
*Cp
O
N
O
O
O_2N
29b
0.93
2.13
2.01
2.07
2.24
0.95
2.00
3.14
3.33
0.95
0.94
2.99
15.00
10.5 10.0 9.5 9.0 8.5 8.0 7.5 7.0 6.5 6.0 5.5 5.0 4.5 4.0 3.5 3.0 2.5 2.0 1.5 1.0 0.5 0.0 -0.5 -1
f1 (ppm)

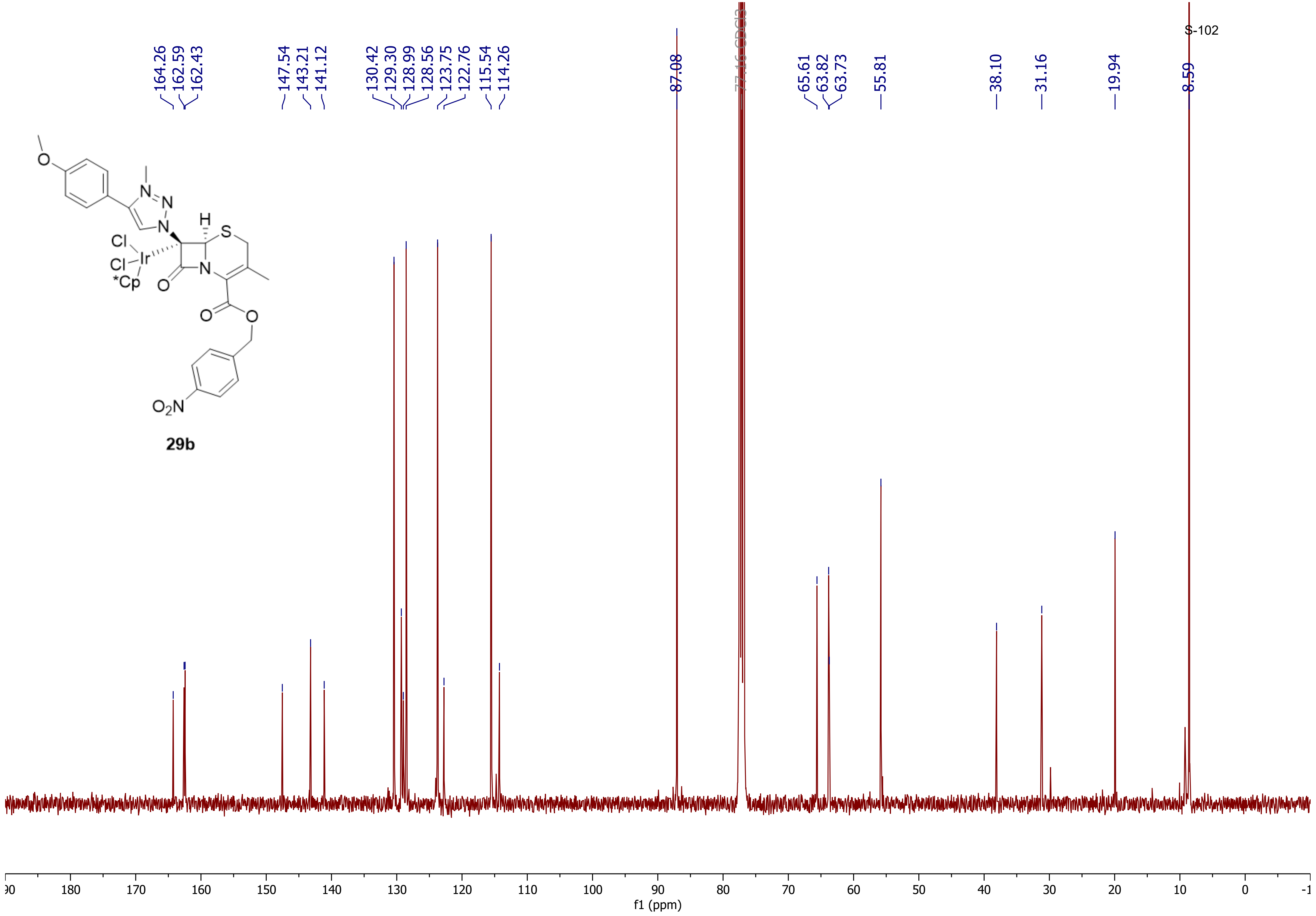
164.26
162.59
162.43
147.54
143.21
141.12
130.42
129.30
128.99
128.56
123.75
122.76
115.54
114.26
87.08
77.16 CDCl3
65.61
63.82
63.73
55.81
38.10
31.16
19.94
8.59
Cl
Cl
Ir
*Cp
N
N
N
O
H
S
N
O
O
O
O₂N
29b
f1 (ppm)

Figure S1.

Variable time experiments. Solvent $CDCl_3$

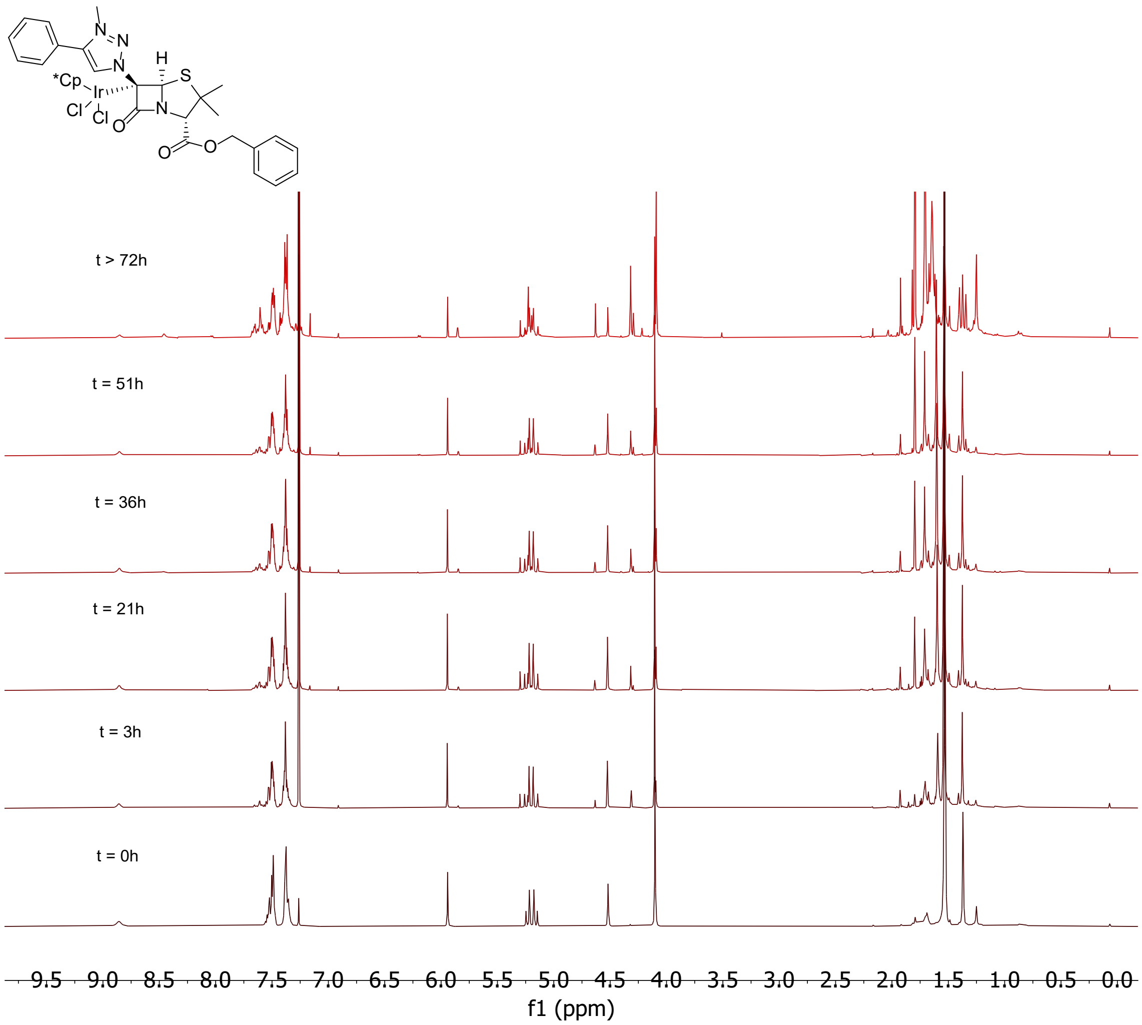

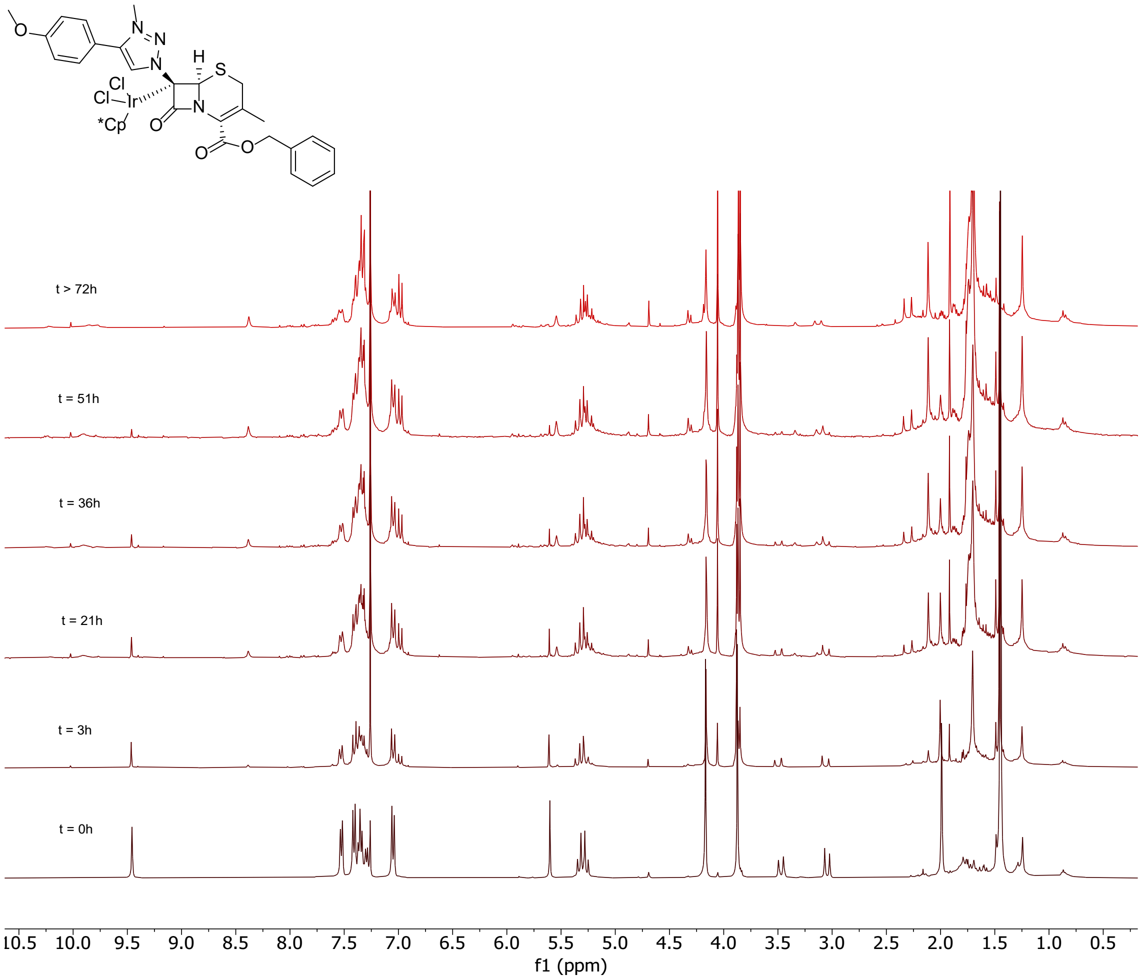

O
N
N
N
H
S
Cl
Cl
Ir
*Cp
O
N
O
O
t > 72h
t = 51h
t = 36h
t = 21h
t = 3h
t = 0h
10.5 10.0 9.5 9.0 8.5 8.0 7.5 7.0 6.5 6.0 5.5 5.0 4.5 4.0 3.5 3.0 2.5 2.0 1.5 1.0 0.5
f1 (ppm)

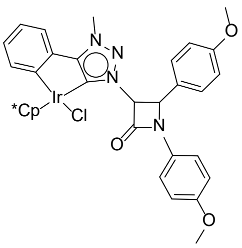
N
N
N
Ir
*Cp
Cl
O
N
O
O

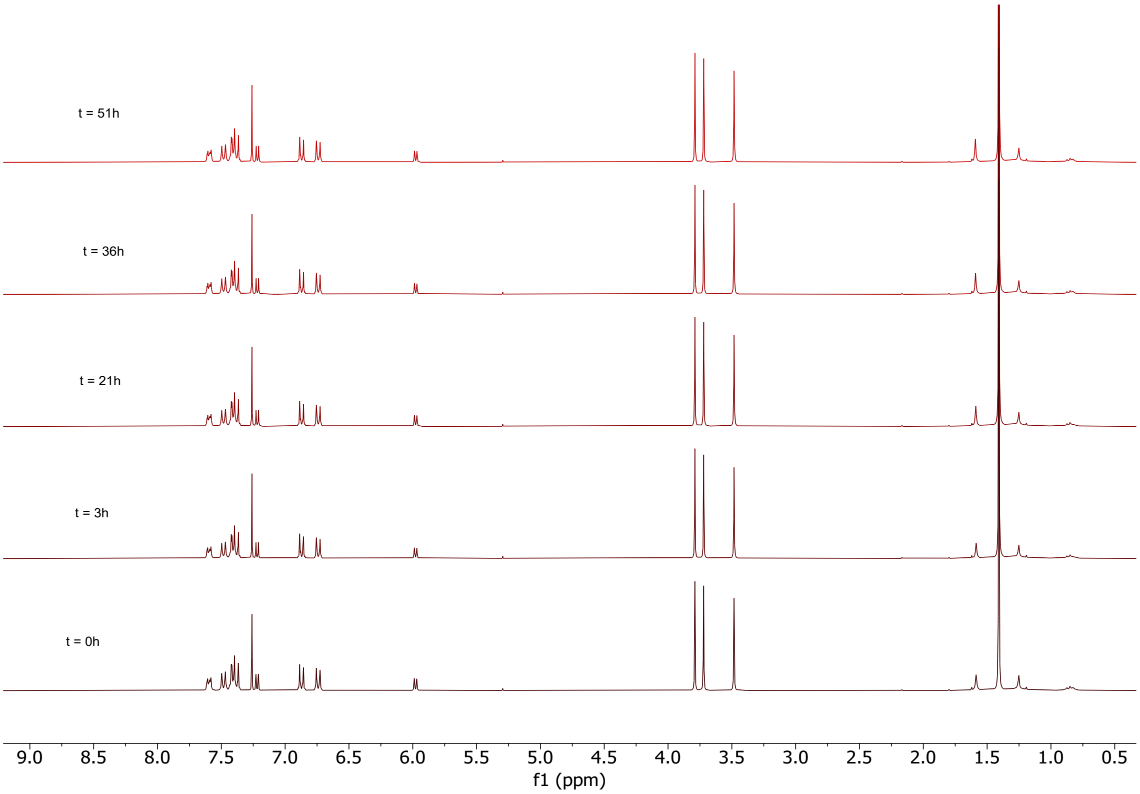
t = 51h
t = 36h
t = 21h
t = 3h
t = 0h
9.0
8.5
8.0
7.5
7.0
6.5
6.0
5.5
5.0
4.5
4.0
3.5
3.0
2.5
2.0
1.5
1.0
0.5
f1 (ppm)

Variable time experiments. Solvent DMSO-d_6/D_2O (4:1 v/v).

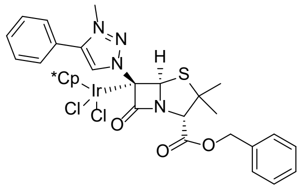

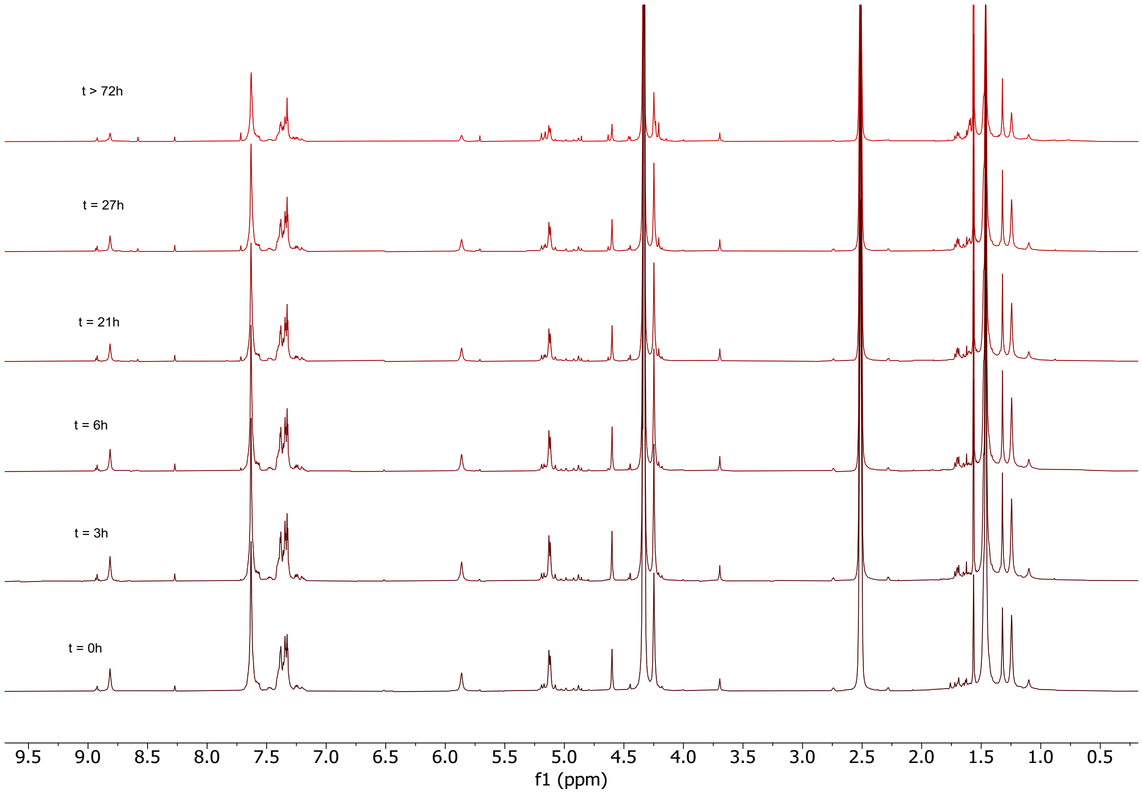

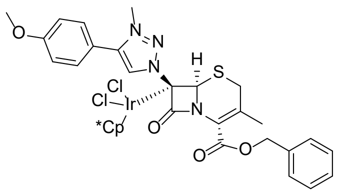

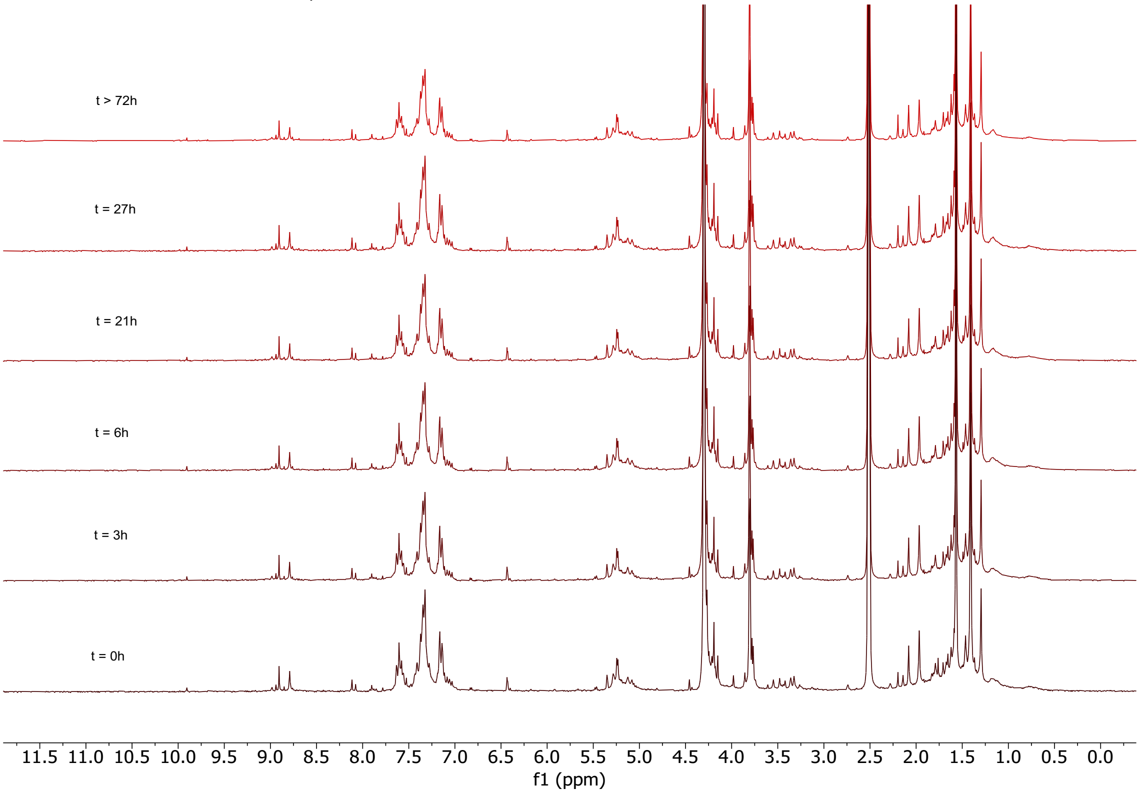
t > 72h
t = 27h
t = 21h
t = 6h
t = 3h
t = 0h
11.5 11.0 10.5 10.0 9.5 9.0 8.5 8.0 7.5 7.0 6.5 6.0 5.5 5.0 4.5 4.0 3.5 3.0 2.5 2.0 1.5 1.0 0.5 0.0
f1 (ppm)

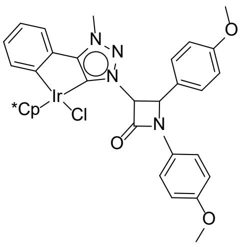
N
N
N
*Cp
Ir
Cl
O
N
O
O

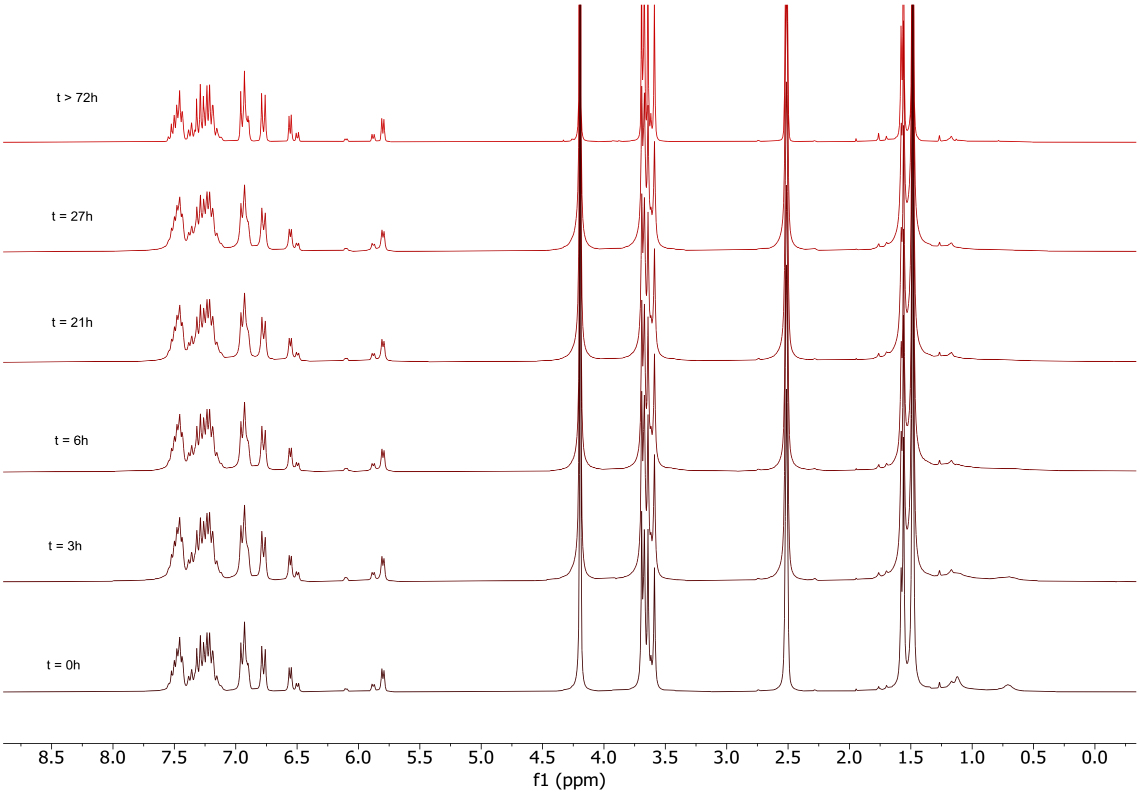
t > 72h
t = 27h
t = 21h
t = 6h
t = 3h
t = 0h
8.5
8.0
7.5
7.0
6.5
6.0
5.5
5.0
4.5
4.0
3.5
3.0
2.5
2.0
1.5
1.0
0.5
0.0
f1 (ppm)
